# Supplementary figures and images for: Inositol polyphosphate multikinase physically binds to the SWI/SNF complex and modulates BRG1 occupancy in mouse embryonic stem cells (part 1 of 2)
Source: eLife. 2022 May 12;11:e73523. doi: 10.7554/eLife.73523 (PMC9098221; doi:10.7554/eLife.73523)

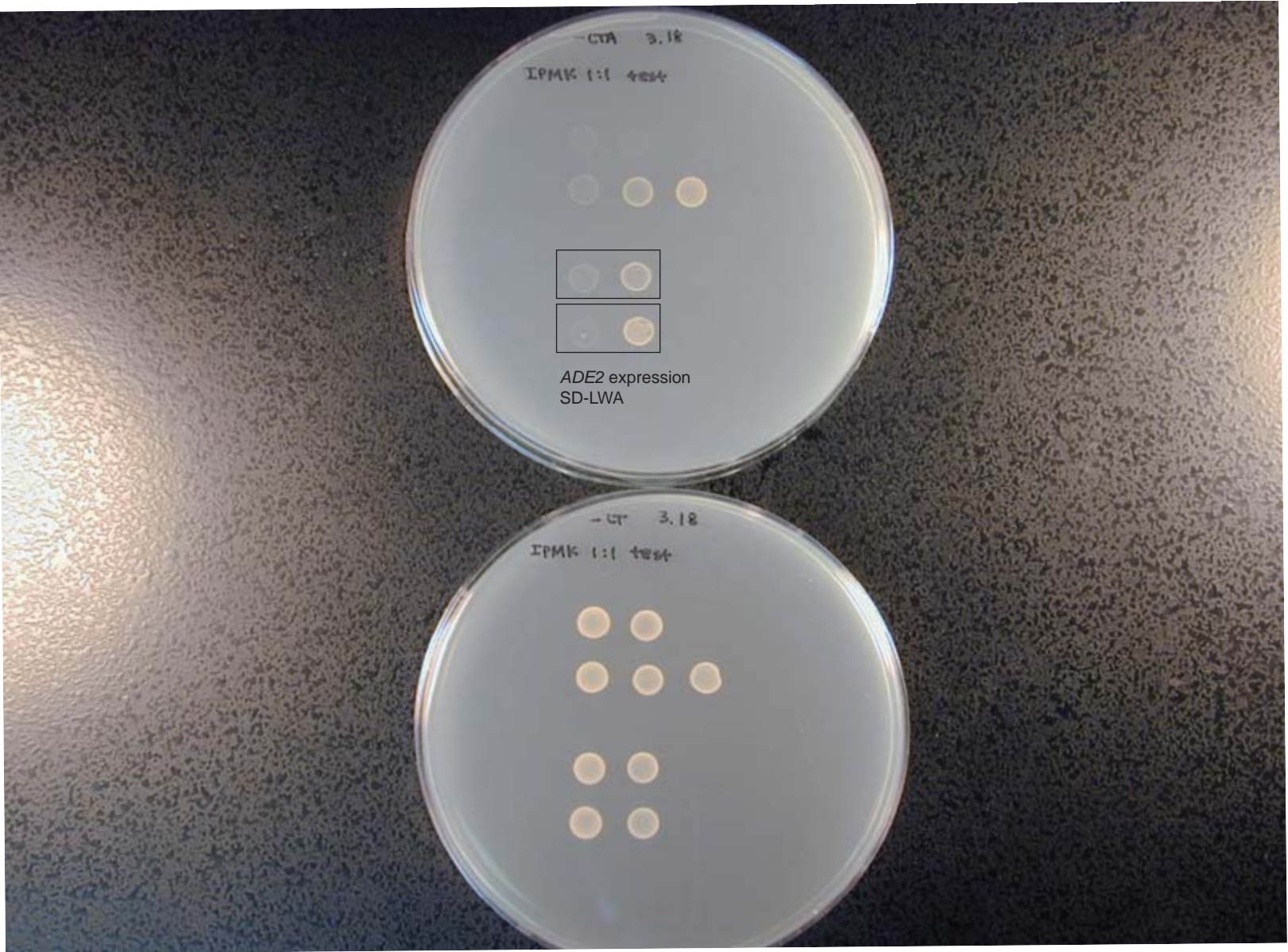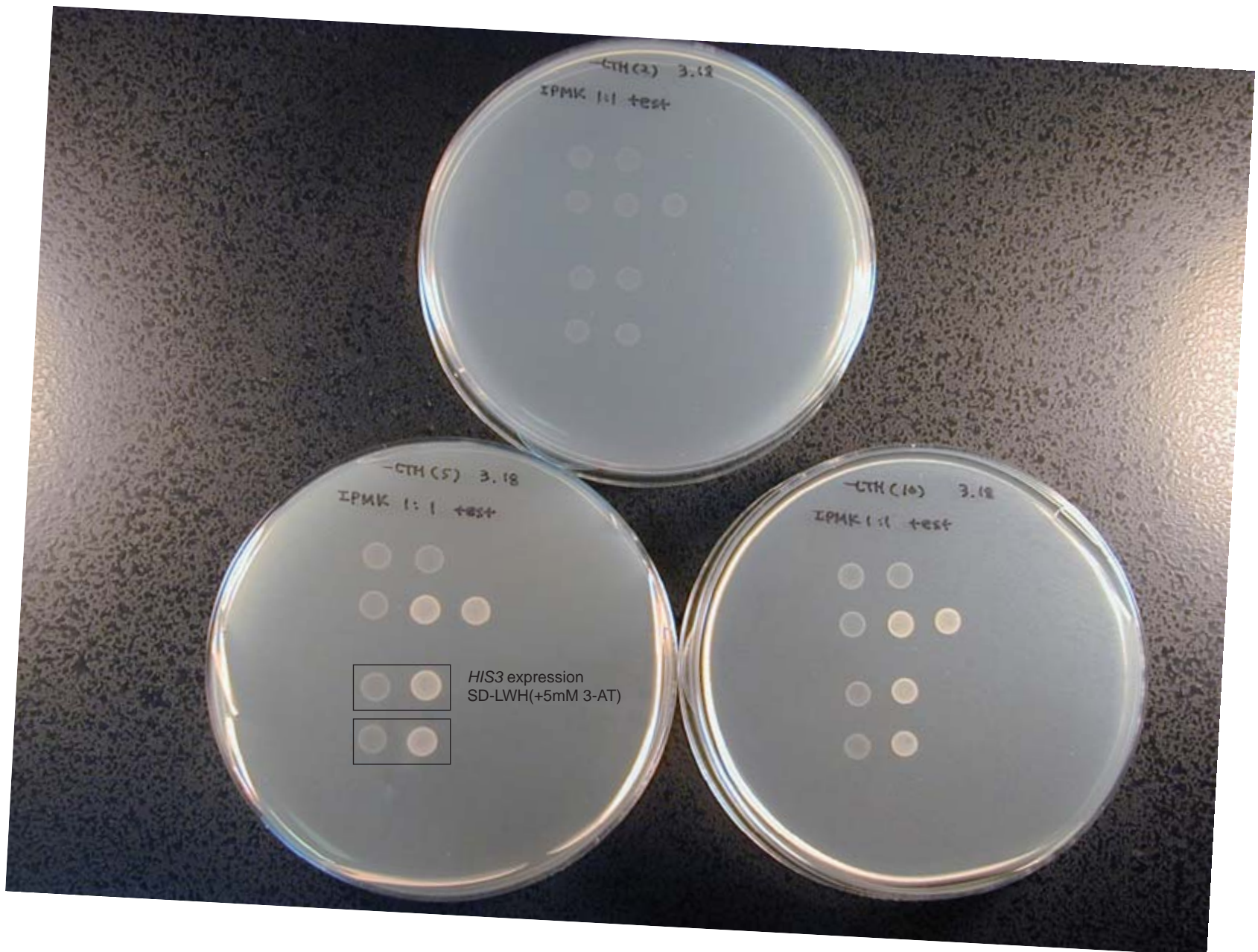

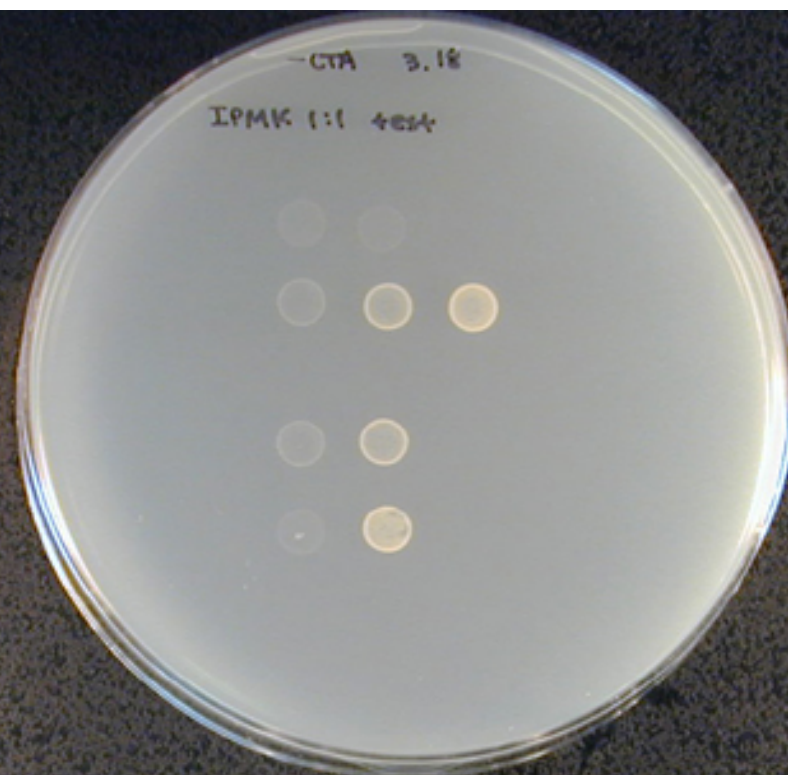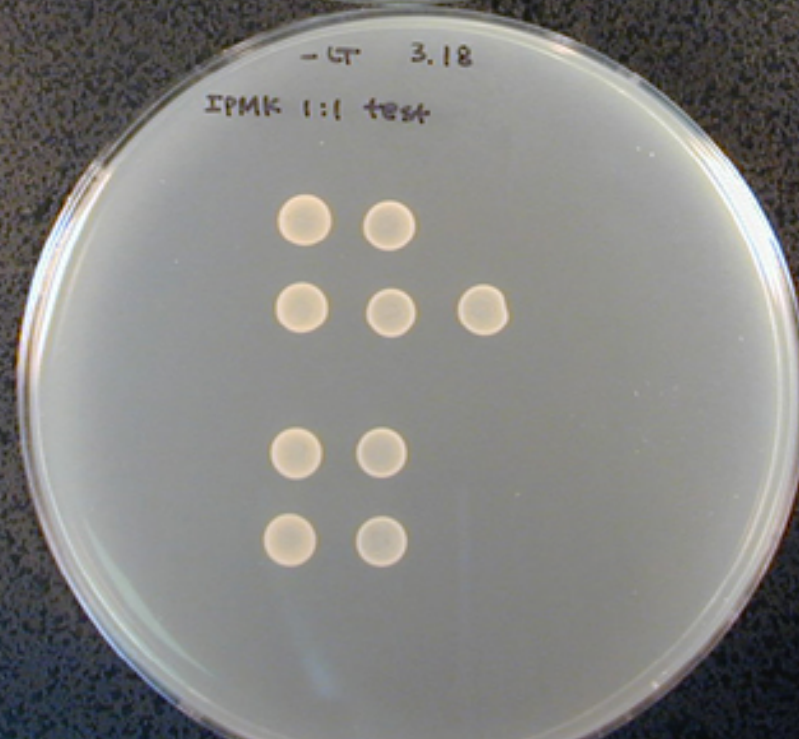

-CTH(2) 3.18  
IPMK 1:1 test

-CTH(5) 3.18  
IPMK 1:1 test

-CTH(10) 3.18  
IPMK 1:1 test

Supplement: Figure 1—source data 1. [file elife-73523-fig1-data1.pdf]

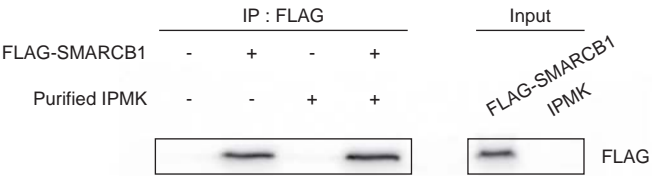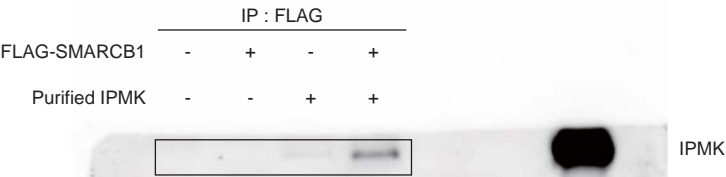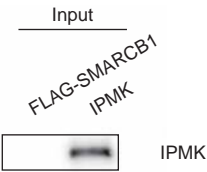

Supplement: Figure 2—source data 1. [file elife-73523-fig2-data1.zip › Figure 2A.pdf]

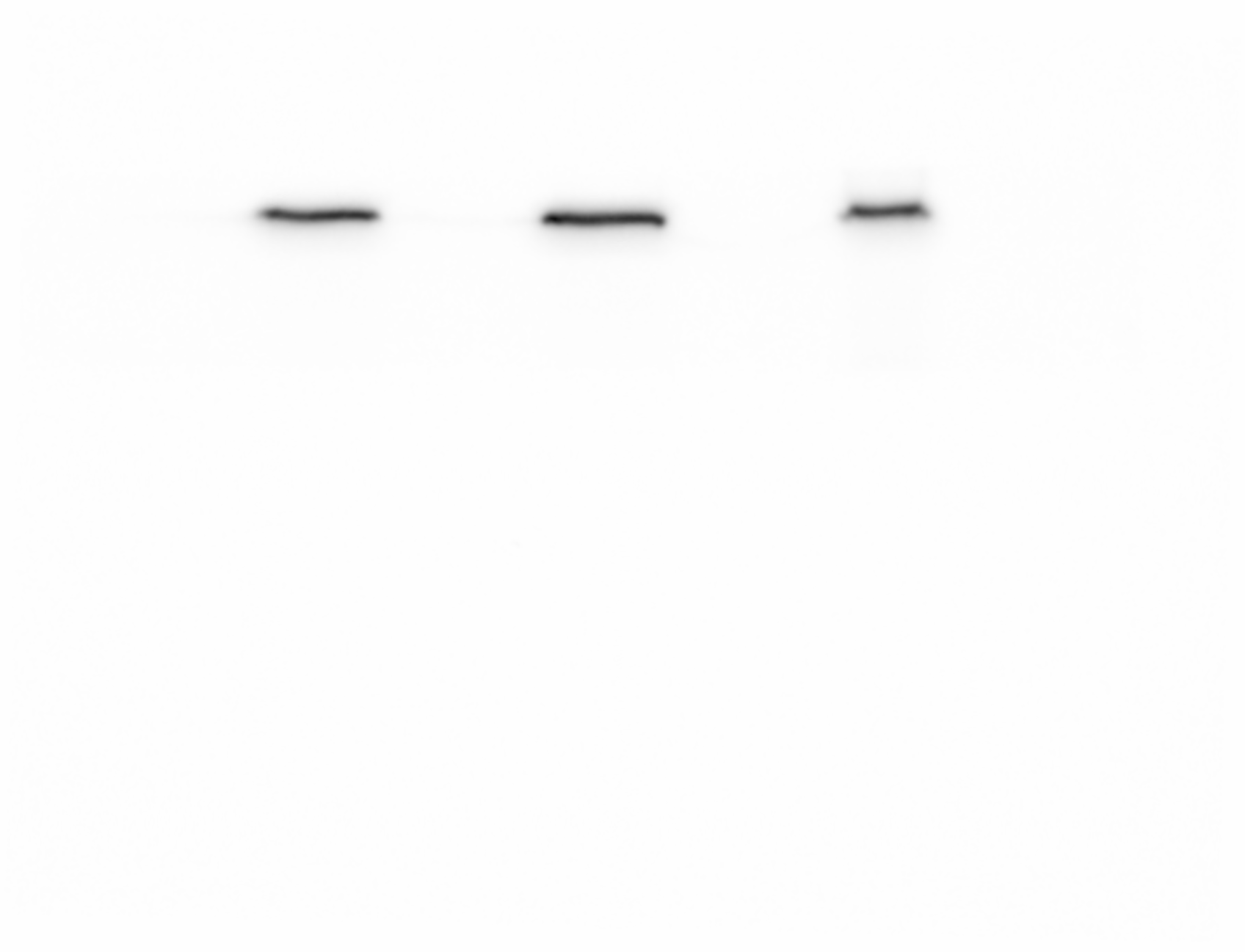

Supplement: Figure 2—source data 1. [file elife-73523-fig2-data1.zip › raw blot/IP and Input_ anti-FLAG.tif]

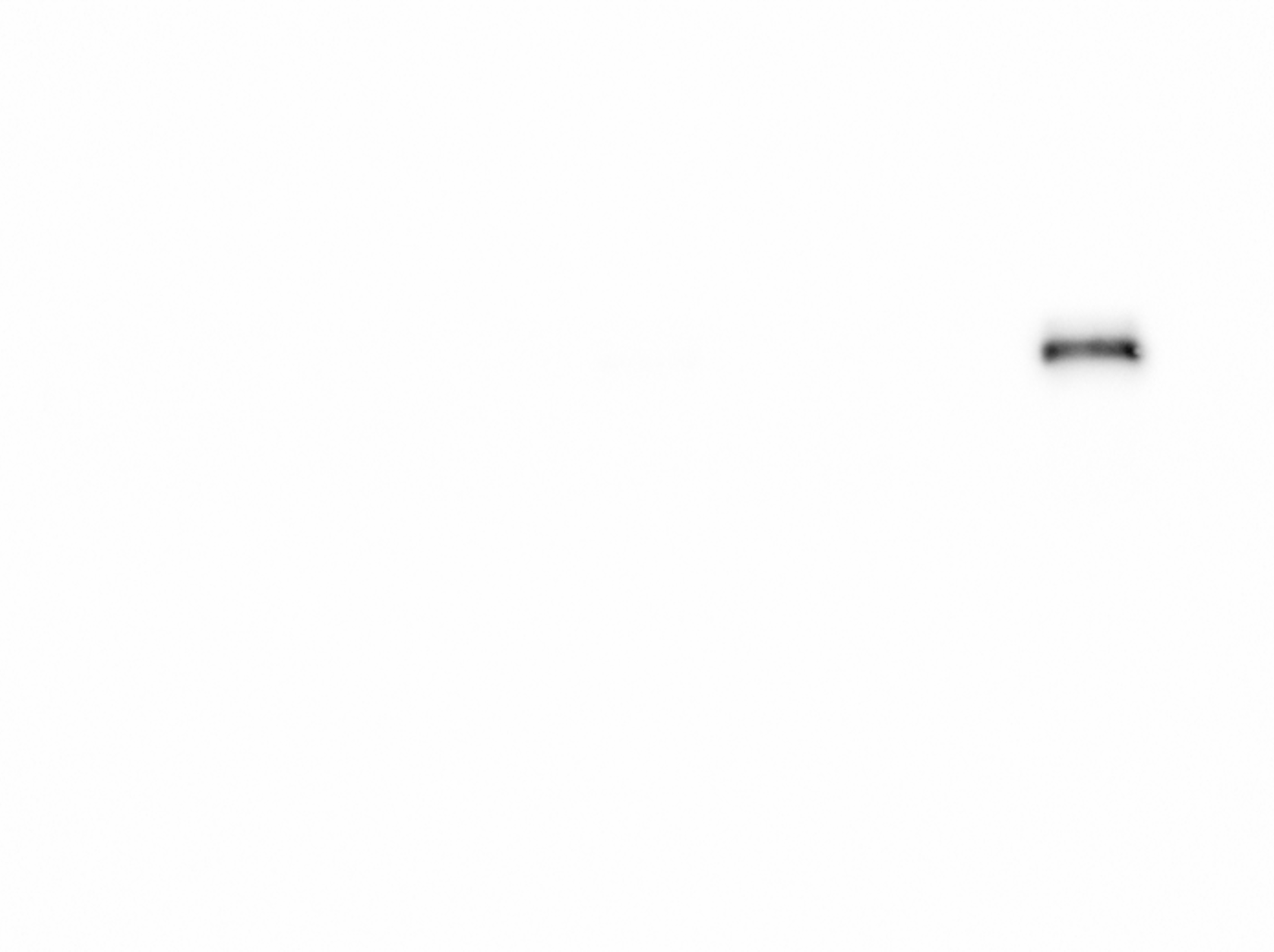

Supplement: Figure 2—source data 1. [file elife-73523-fig2-data1.zip › raw blot/Input_ anti-IPMK.tif]

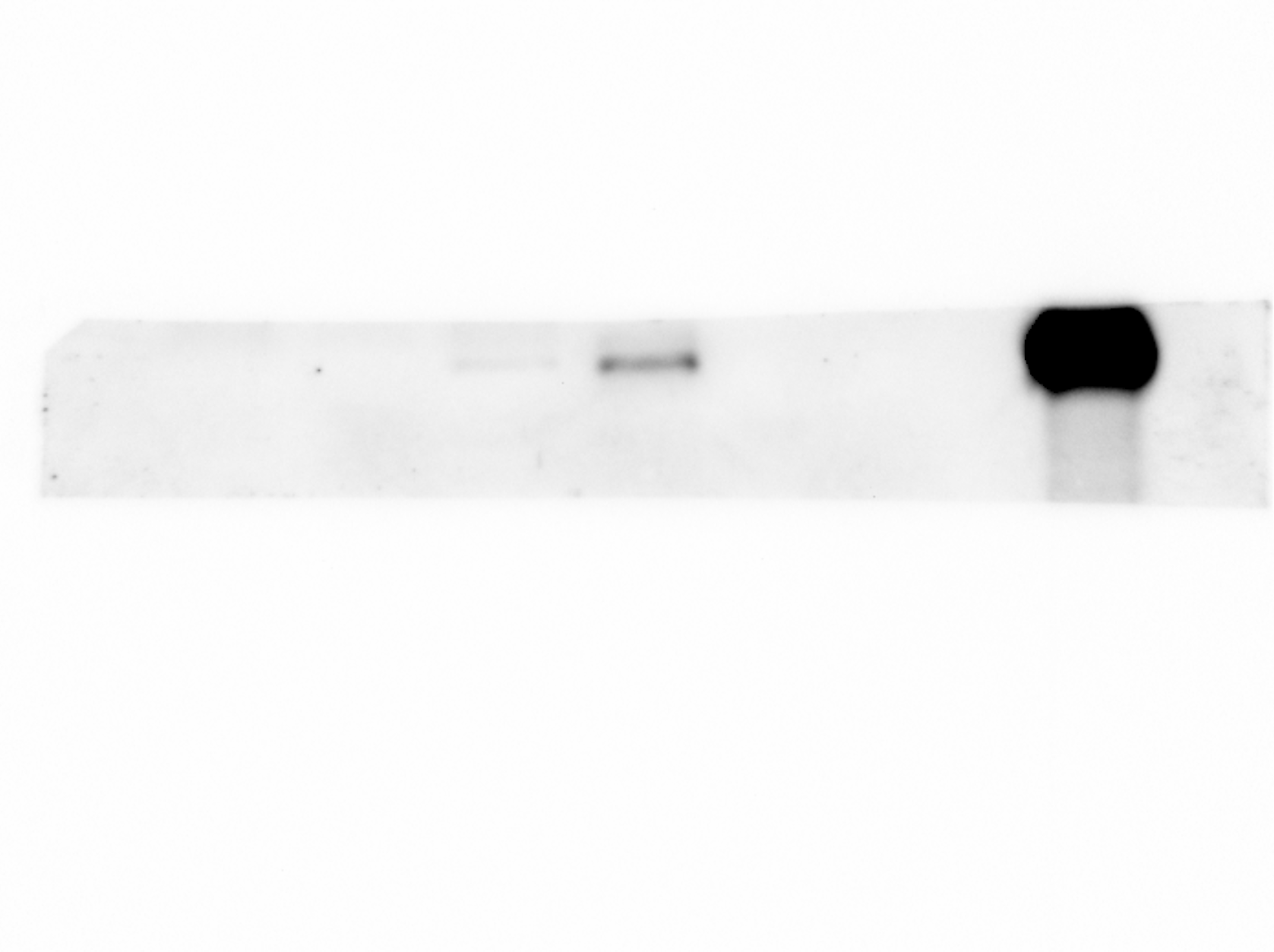

Supplement: Figure 2—source data 1. [file elife-73523-fig2-data1.zip › raw blot/IP_ anti-IPMK.tif]

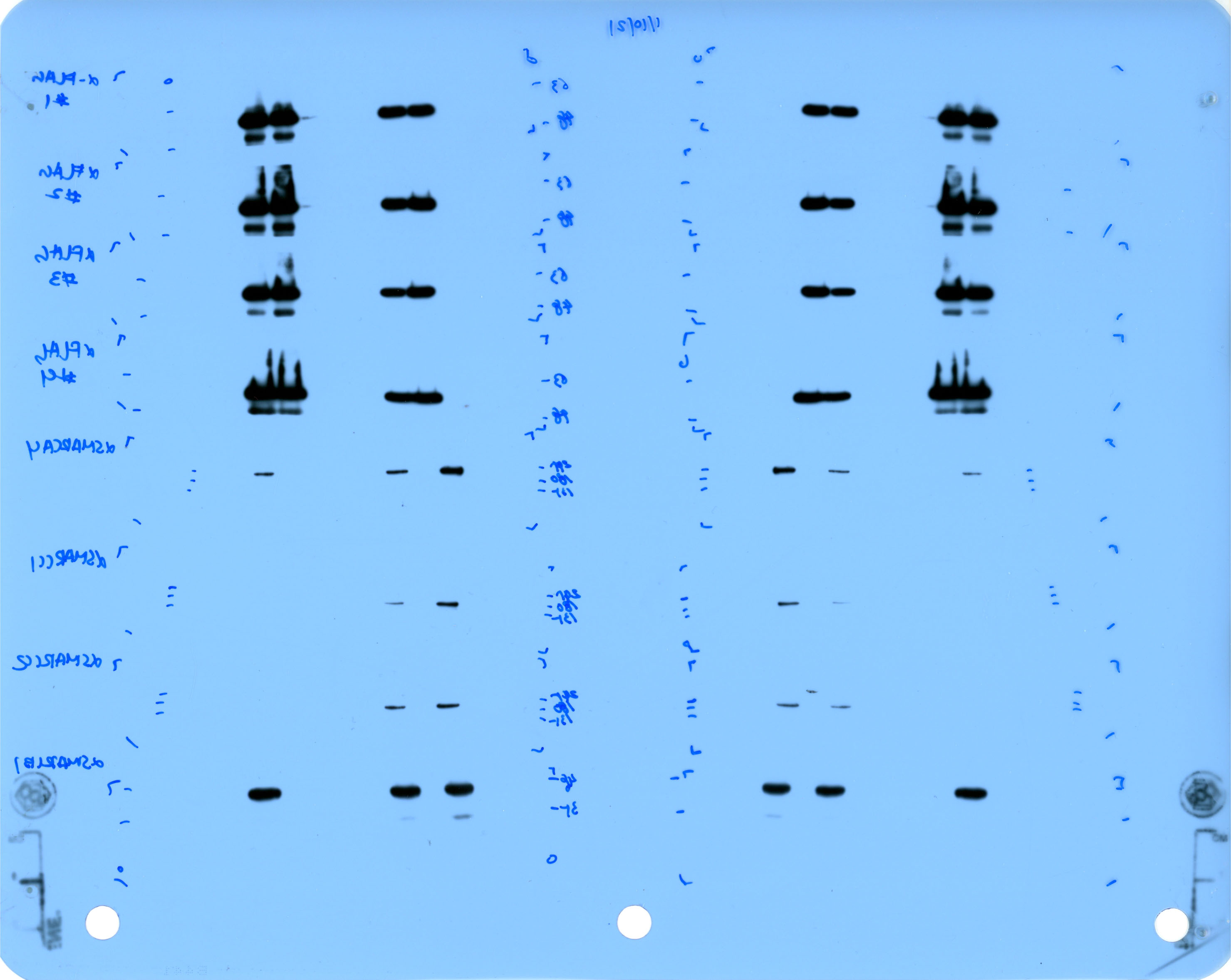

Supplement: Figure 2—source data 2. [file elife-73523-fig2-data2.zip › Raw blots/IPMK SMARCC2/FLAG.jpg]

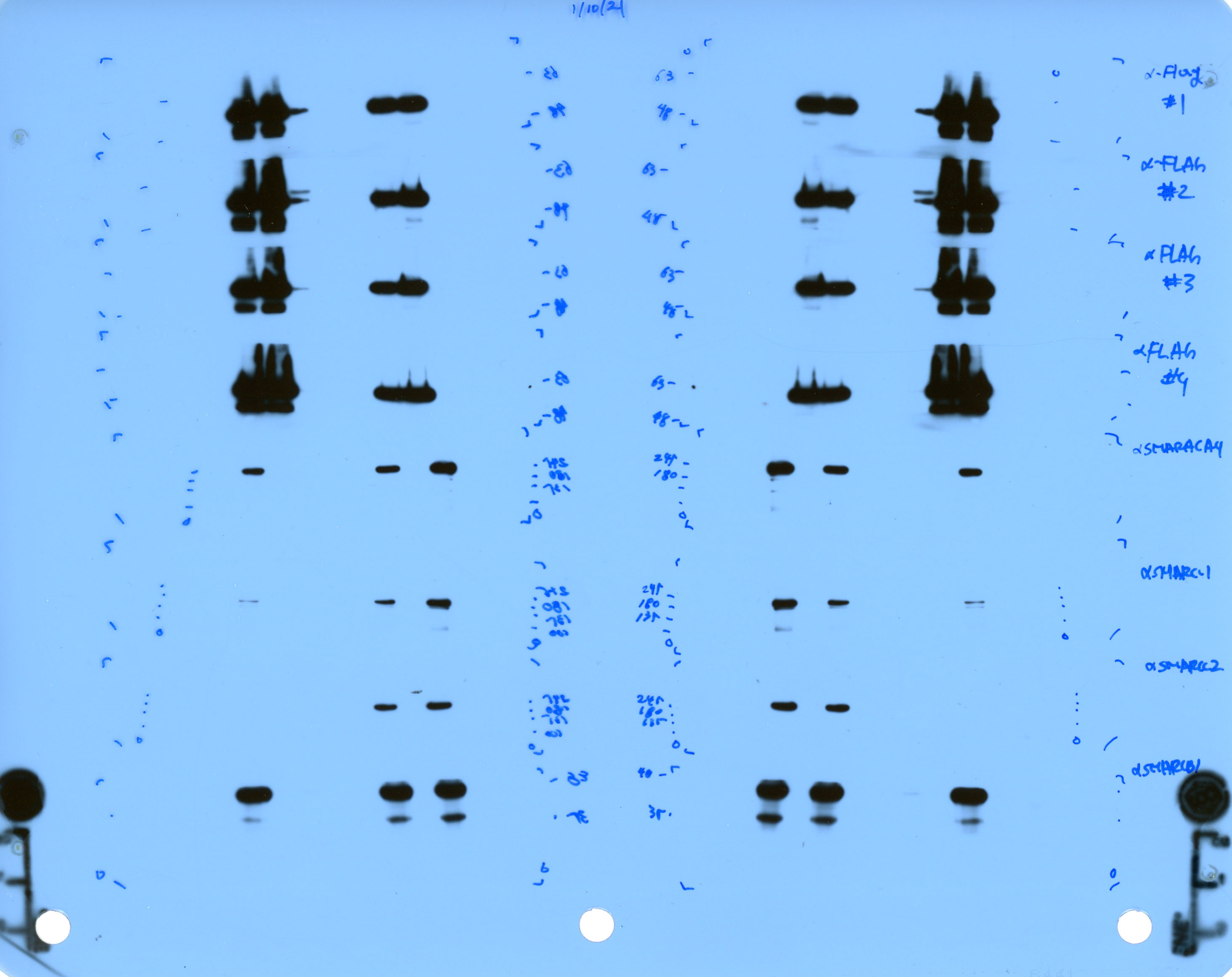

Supplement: Figure 2—source data 2. [file elife-73523-fig2-data2.zip › Raw blots/IPMK SMARCC2/SMARCC2.jpg]

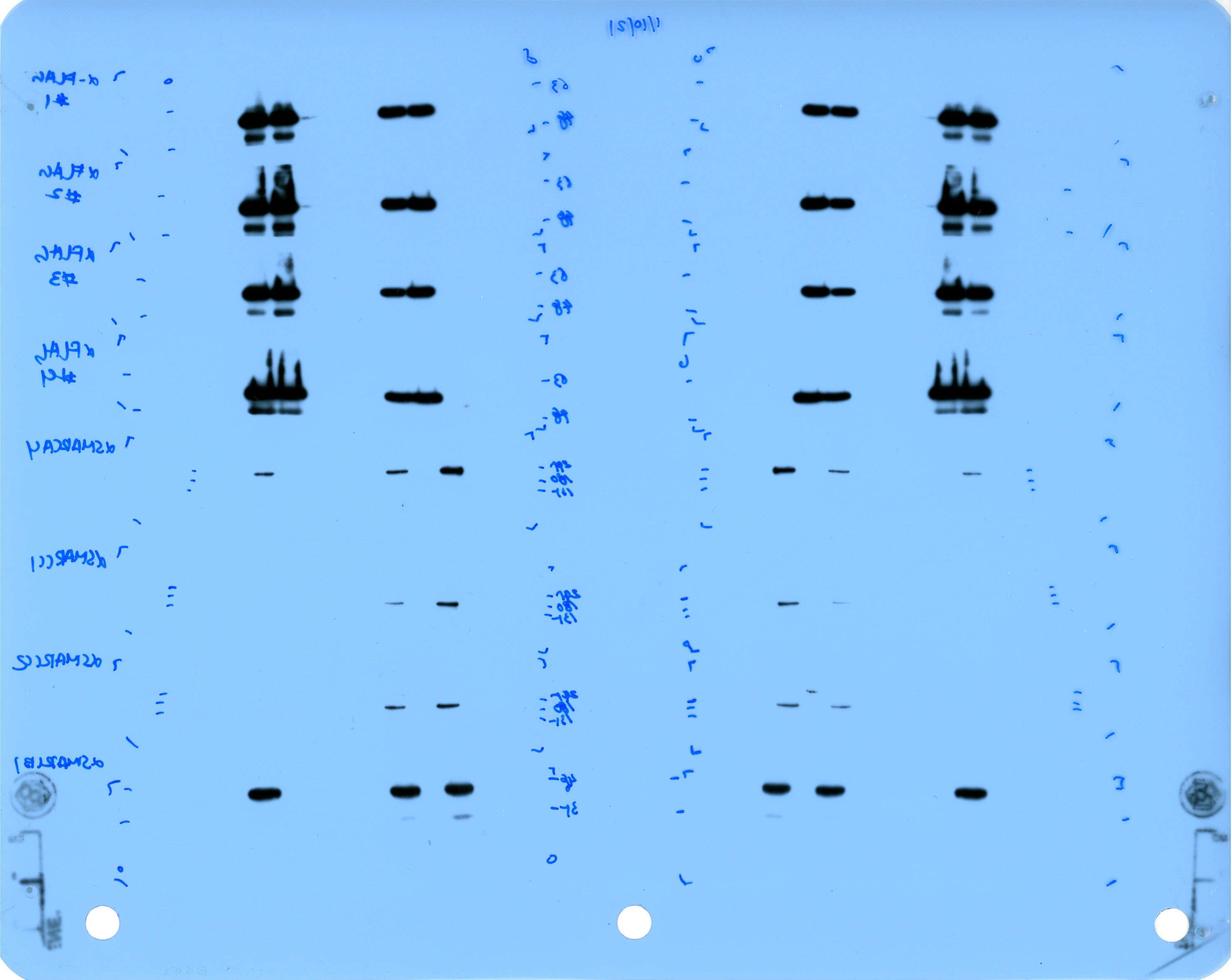

Supplement: Figure 2—source data 2. [file elife-73523-fig2-data2.zip › Raw blots/IPMK SMARCB1/SMARCB1_and_FLAG.jpg]

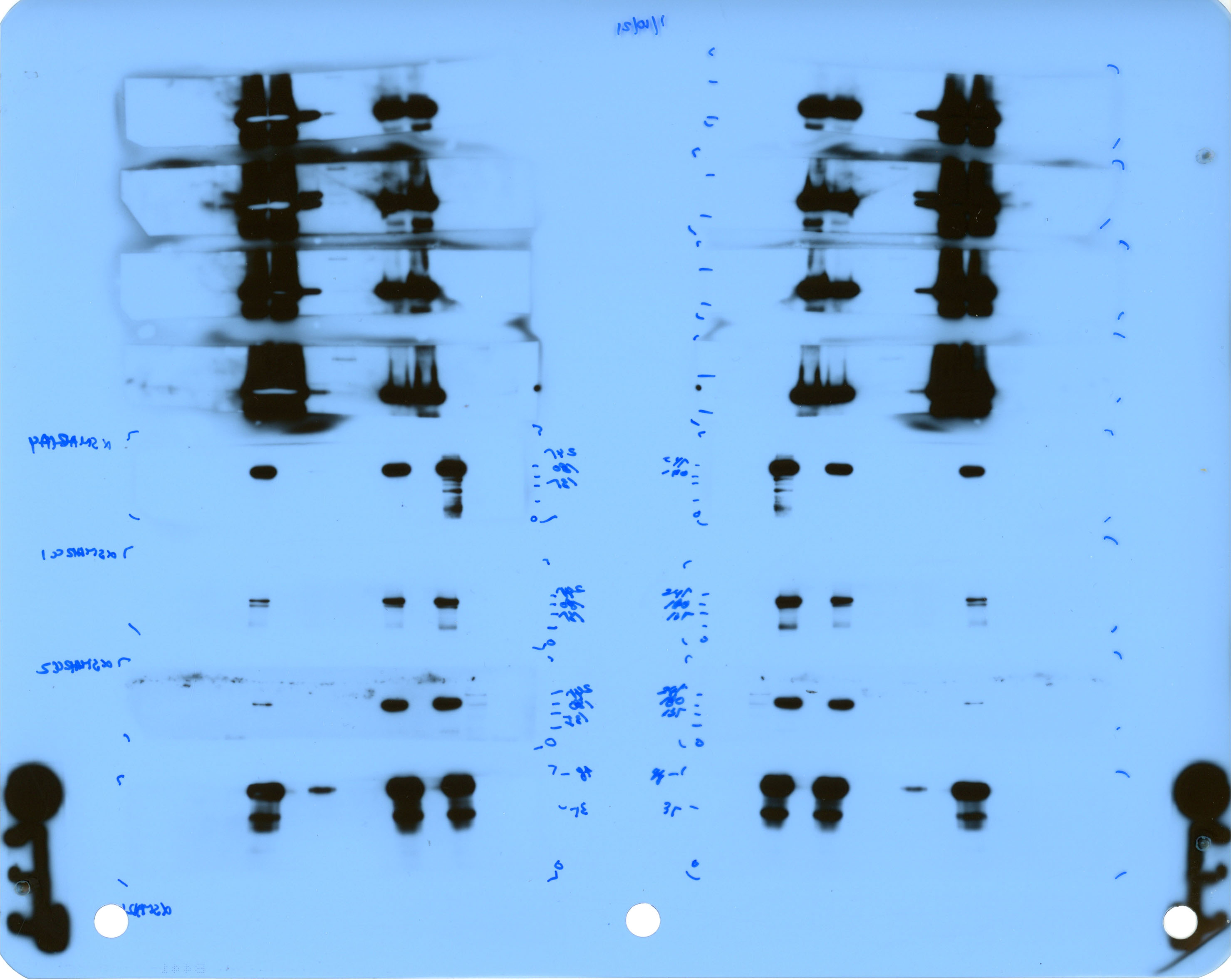

Supplement: Figure 2—source data 2. [file elife-73523-fig2-data2.zip › Raw blots/IPMK SMARCC1/SMARCC1.jpg]

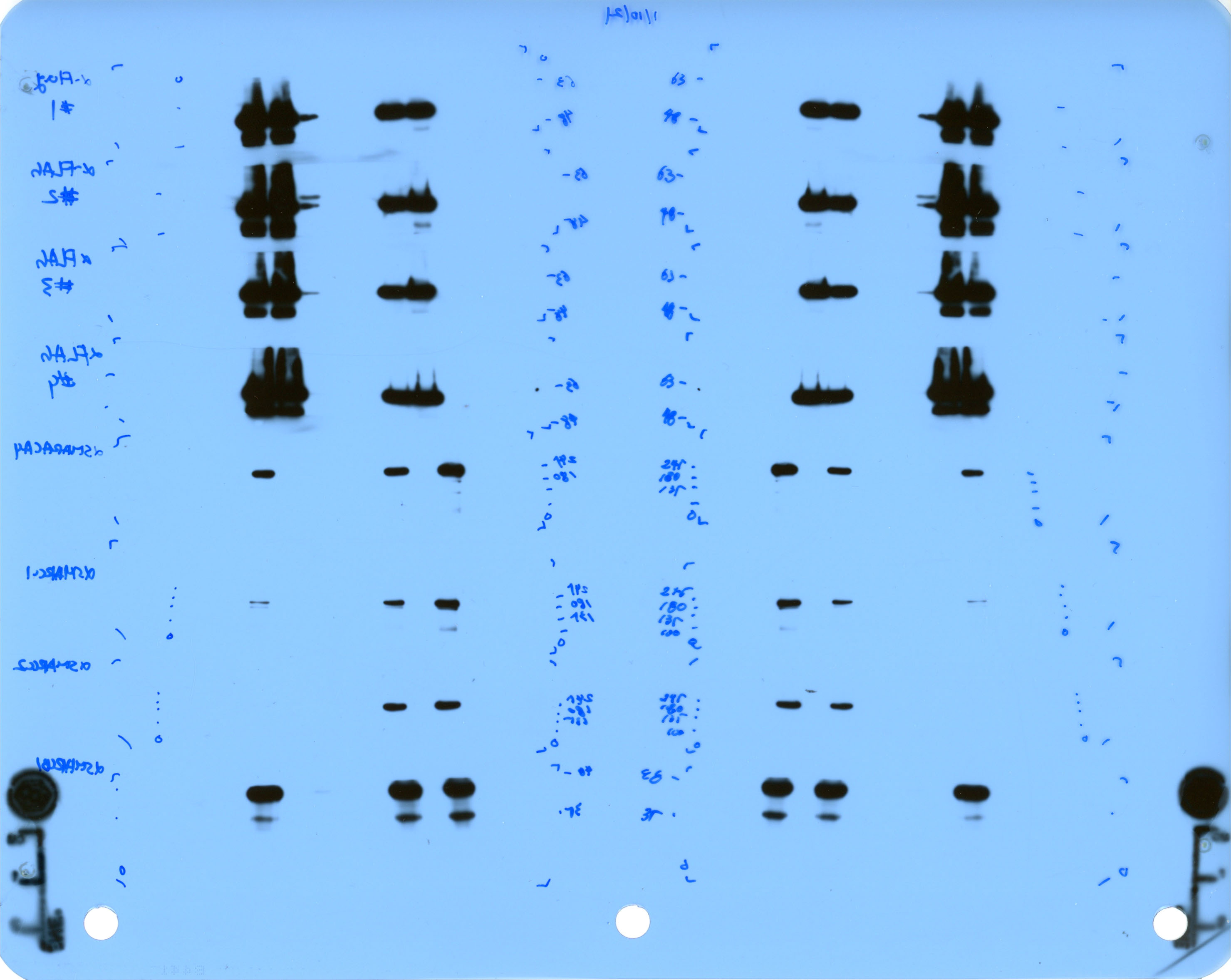

Supplement: Figure 2—source data 2. [file elife-73523-fig2-data2.zip › Raw blots/IPMK SMARCA4/SMARCA4.jpg]

Figure 2E  
Top Left

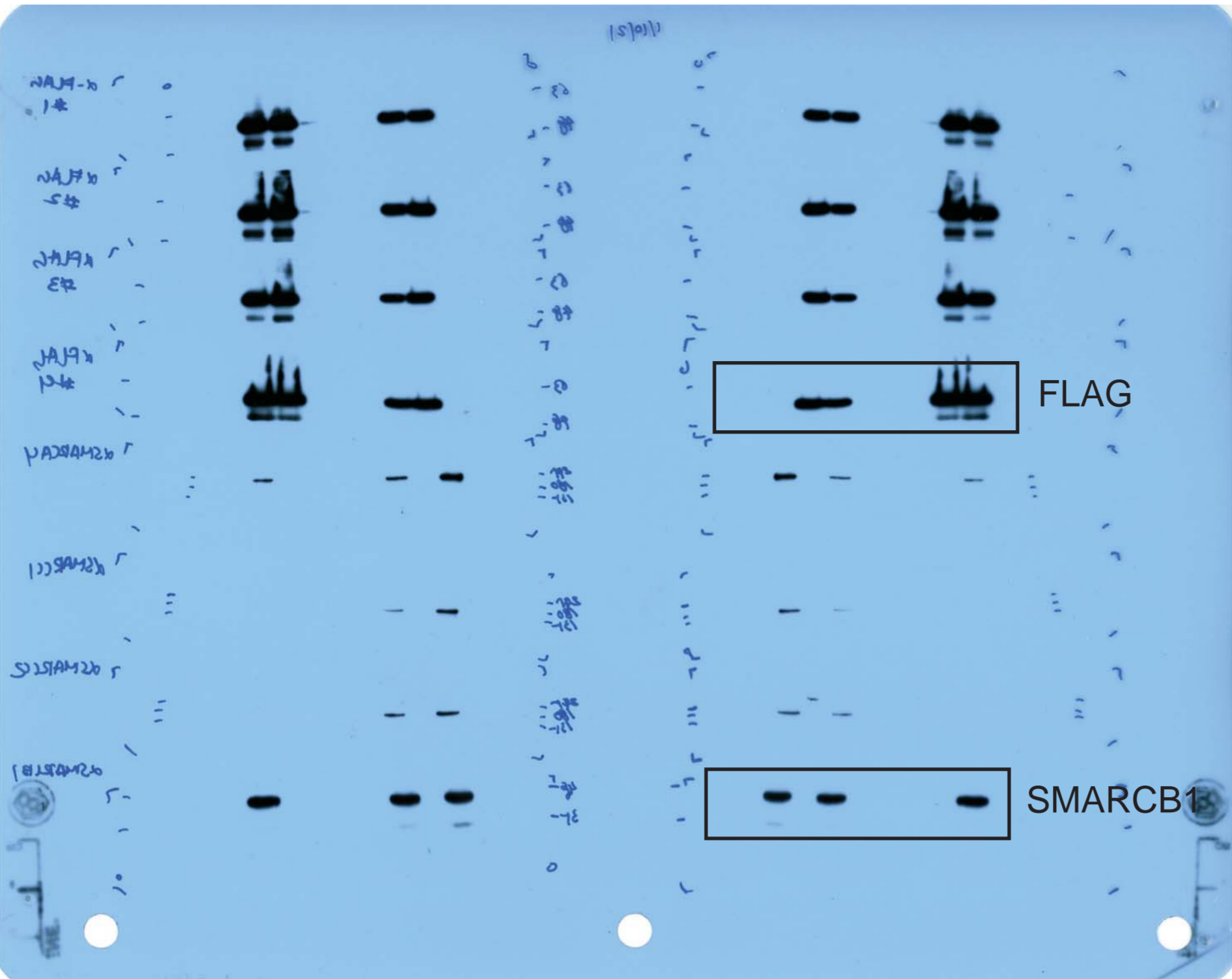

Figure 2E  
Top Right

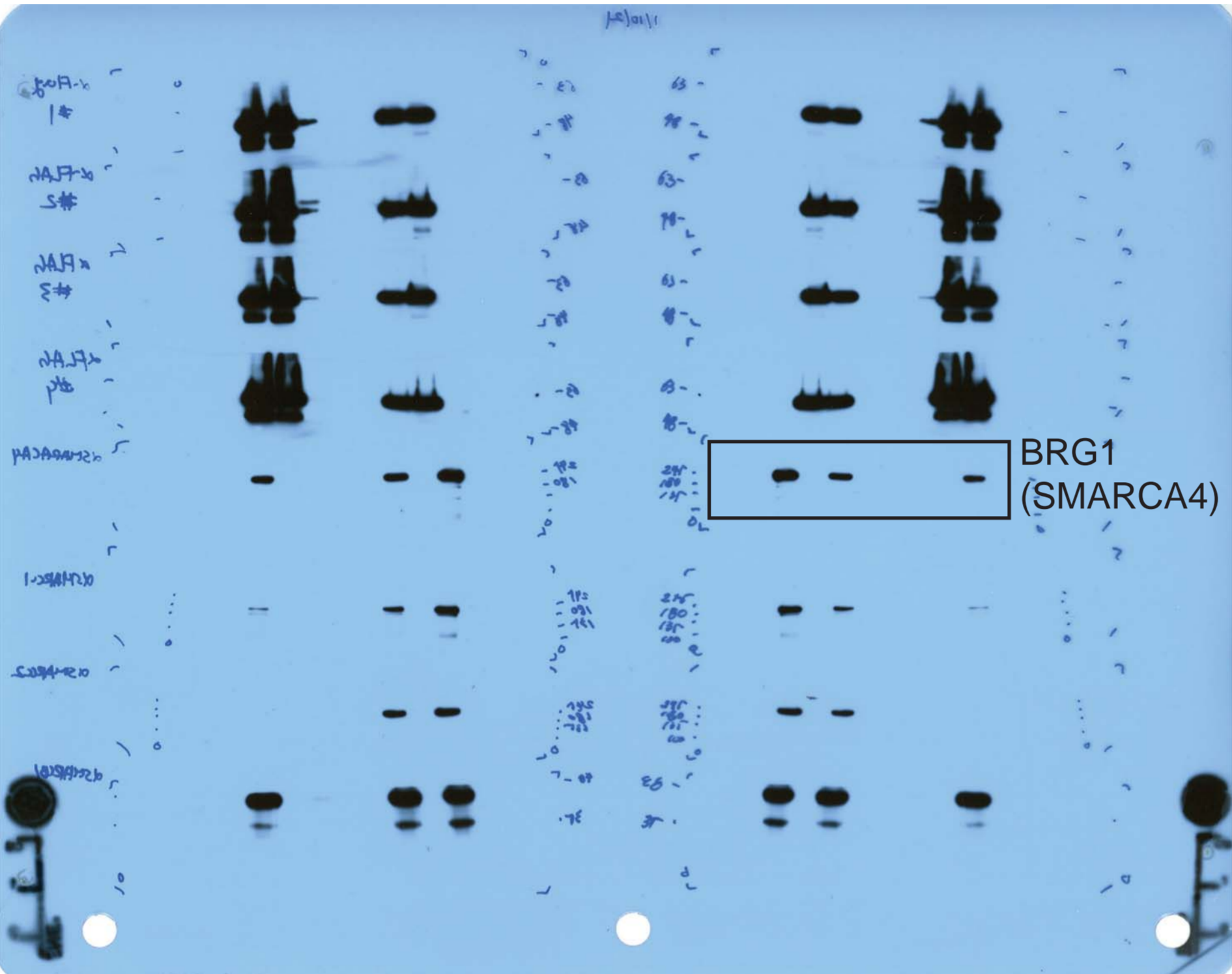

Figure 2E  
Bottom Left

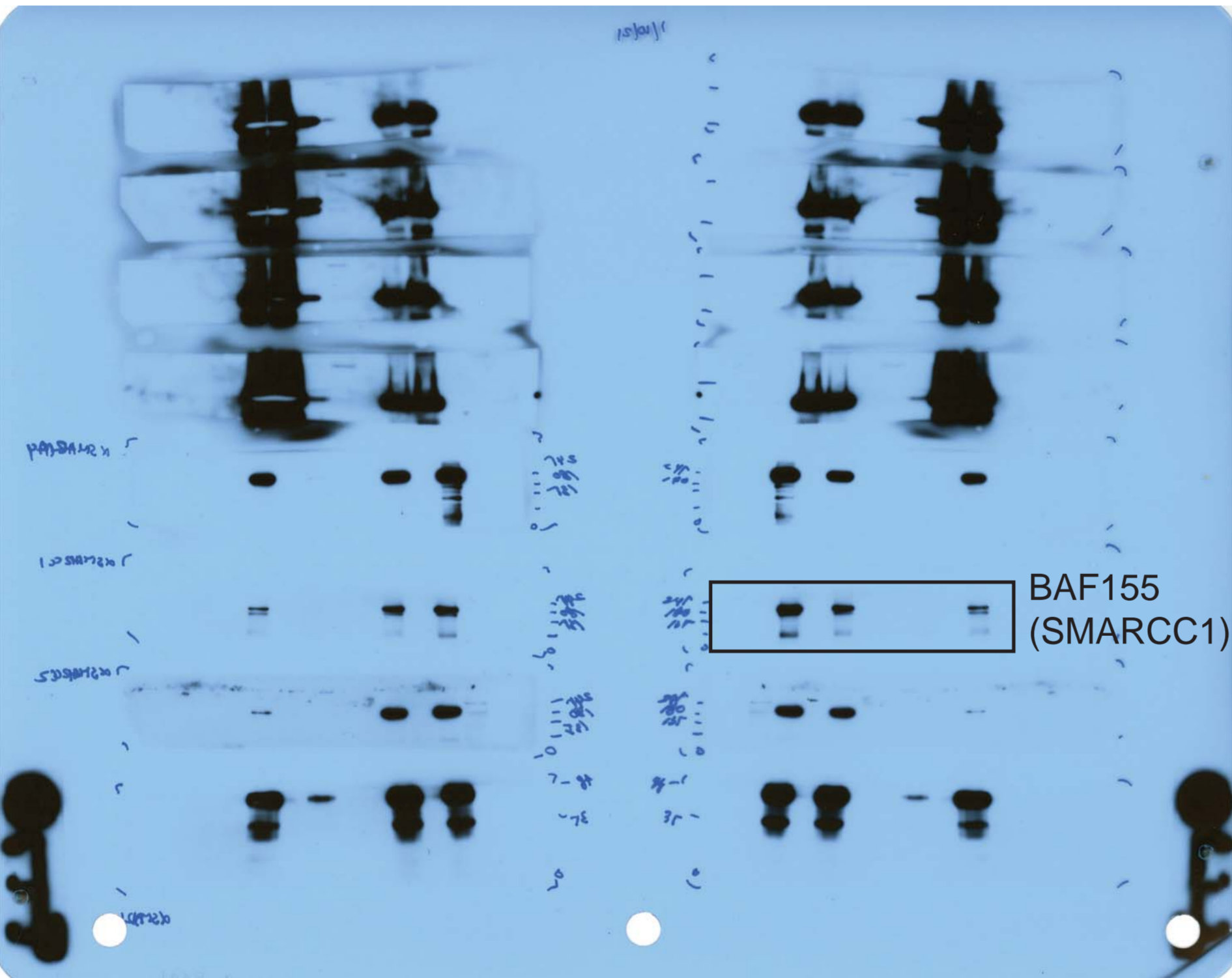

Figure 2E  
Bottom Right

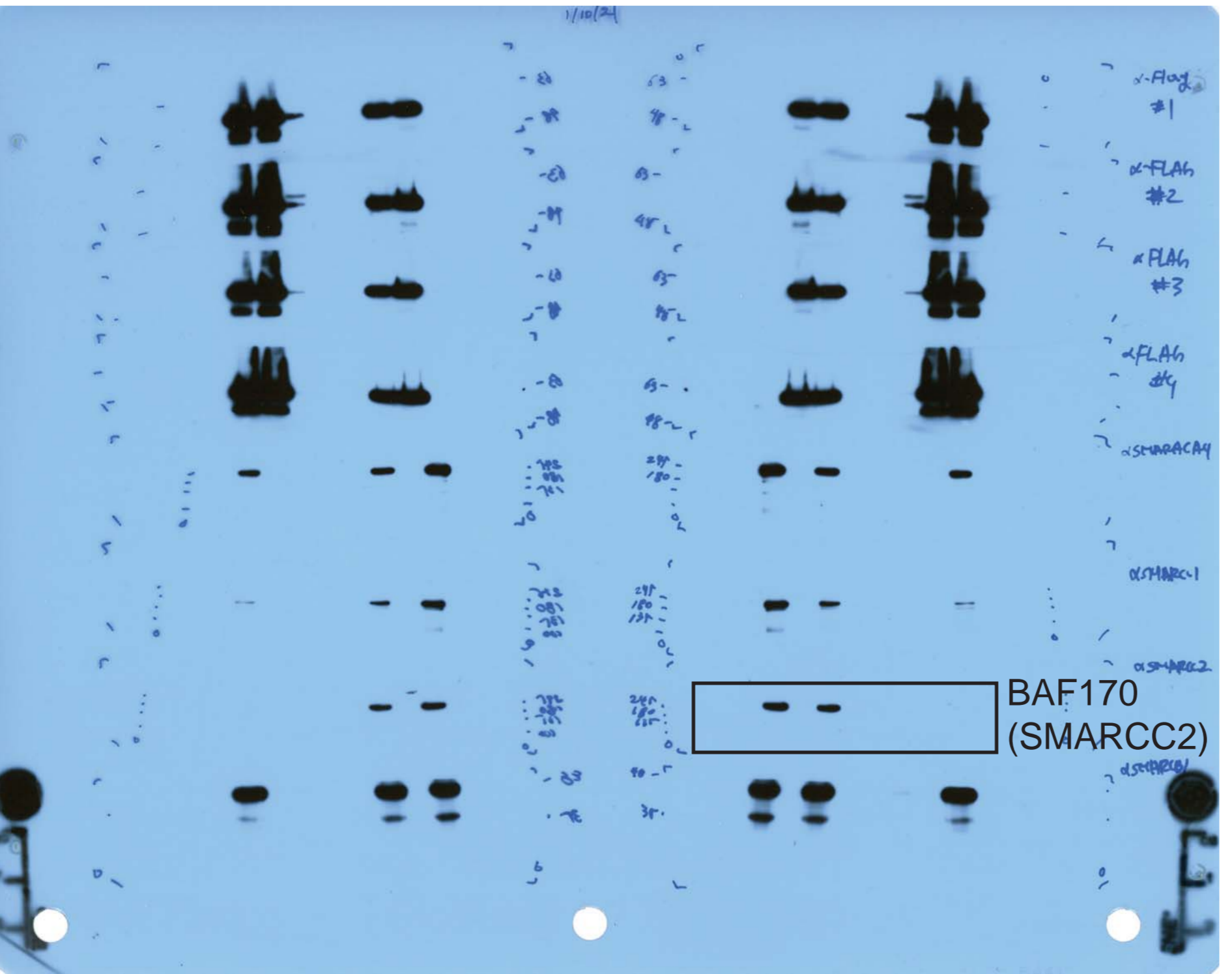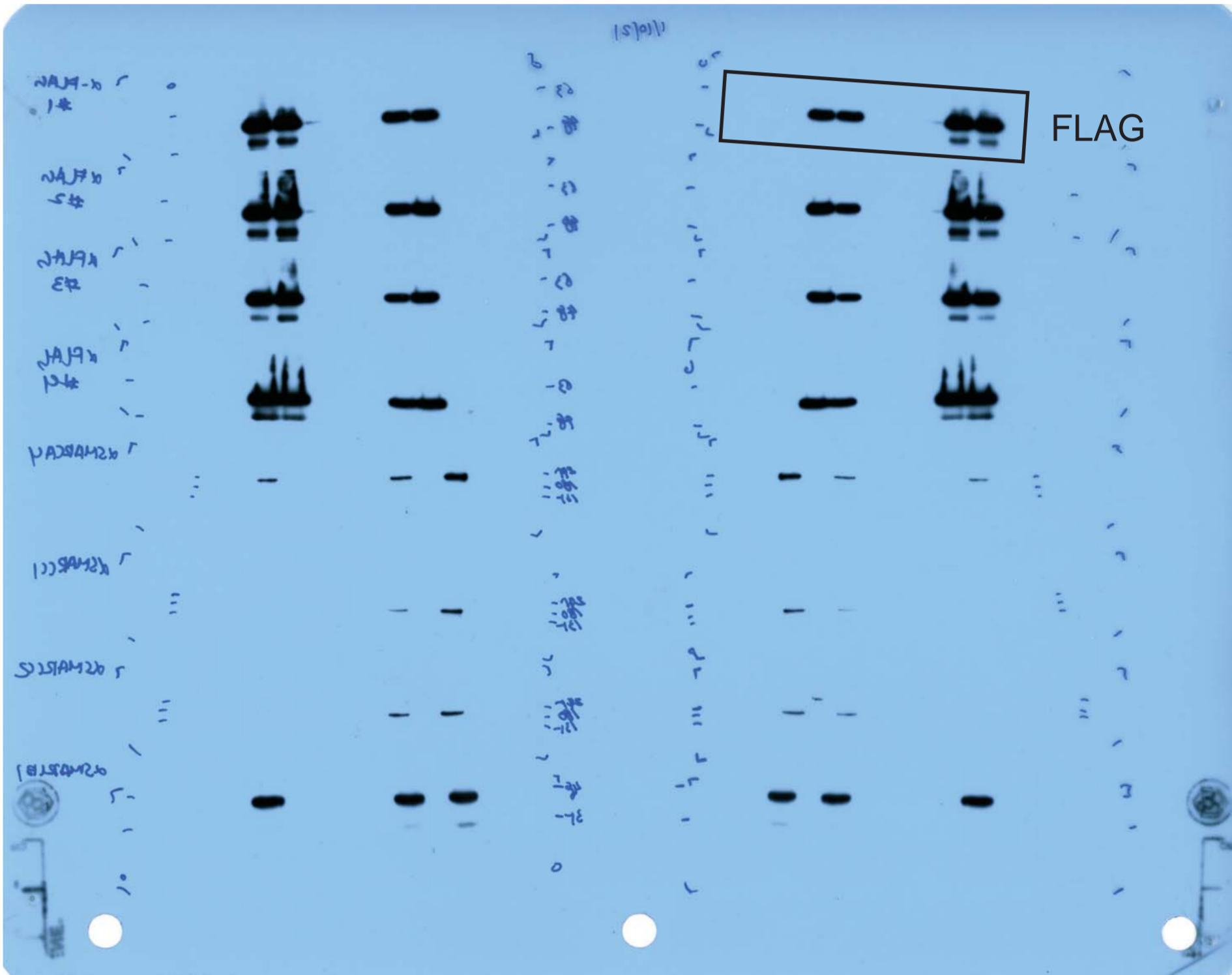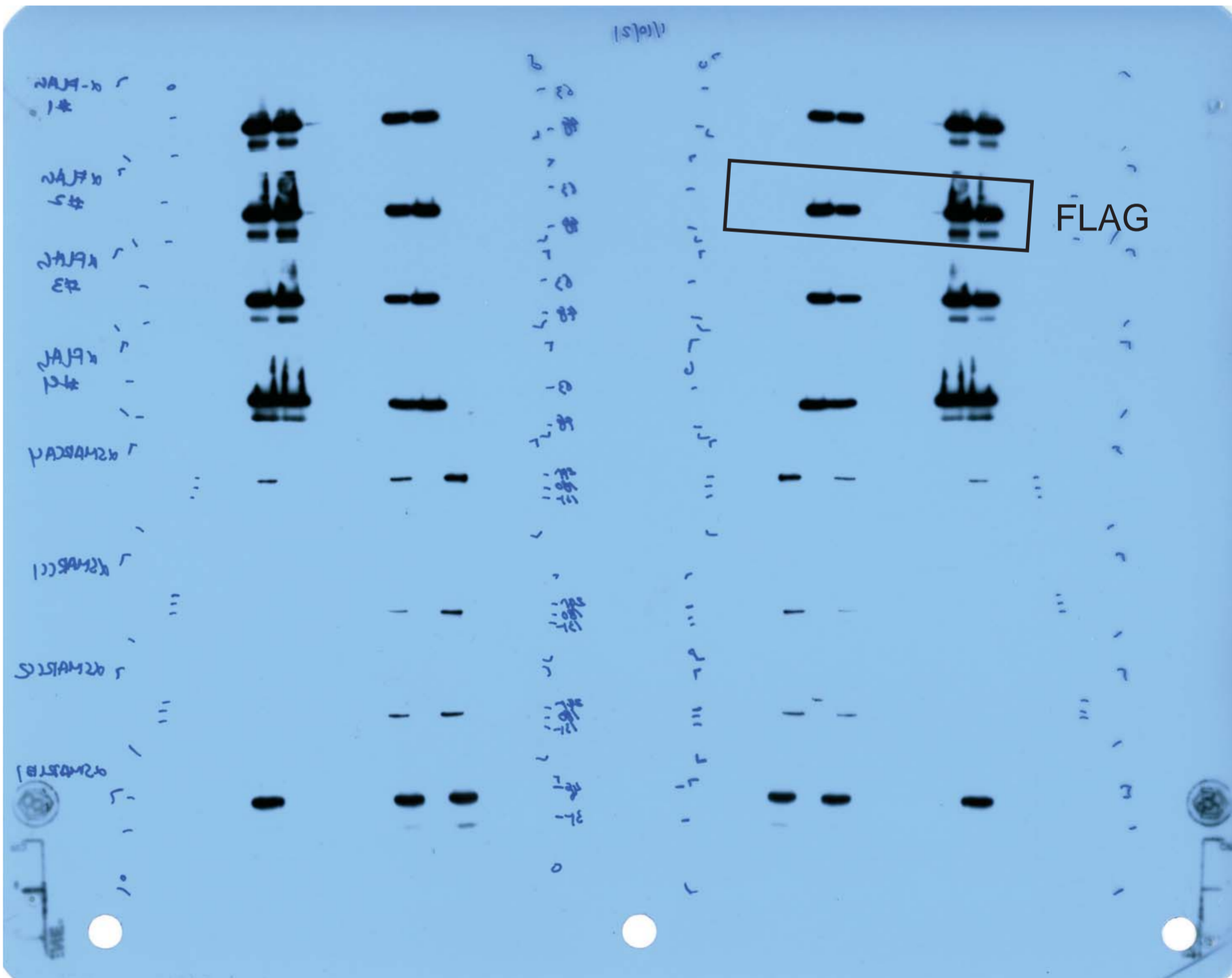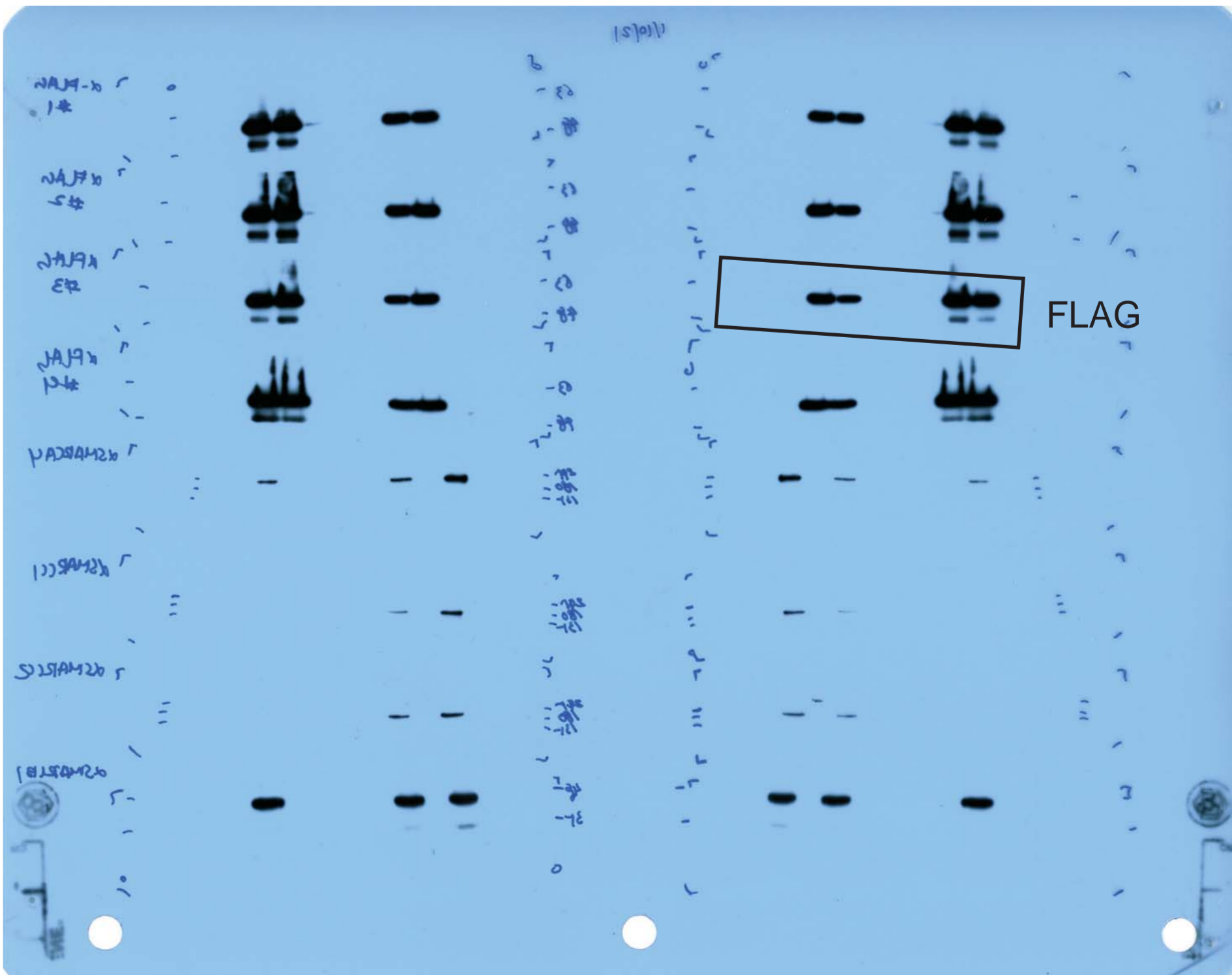

Supplement: Figure 2—source data 2. [file elife-73523-fig2-data2.zip › Labelled raw blots.pdf]

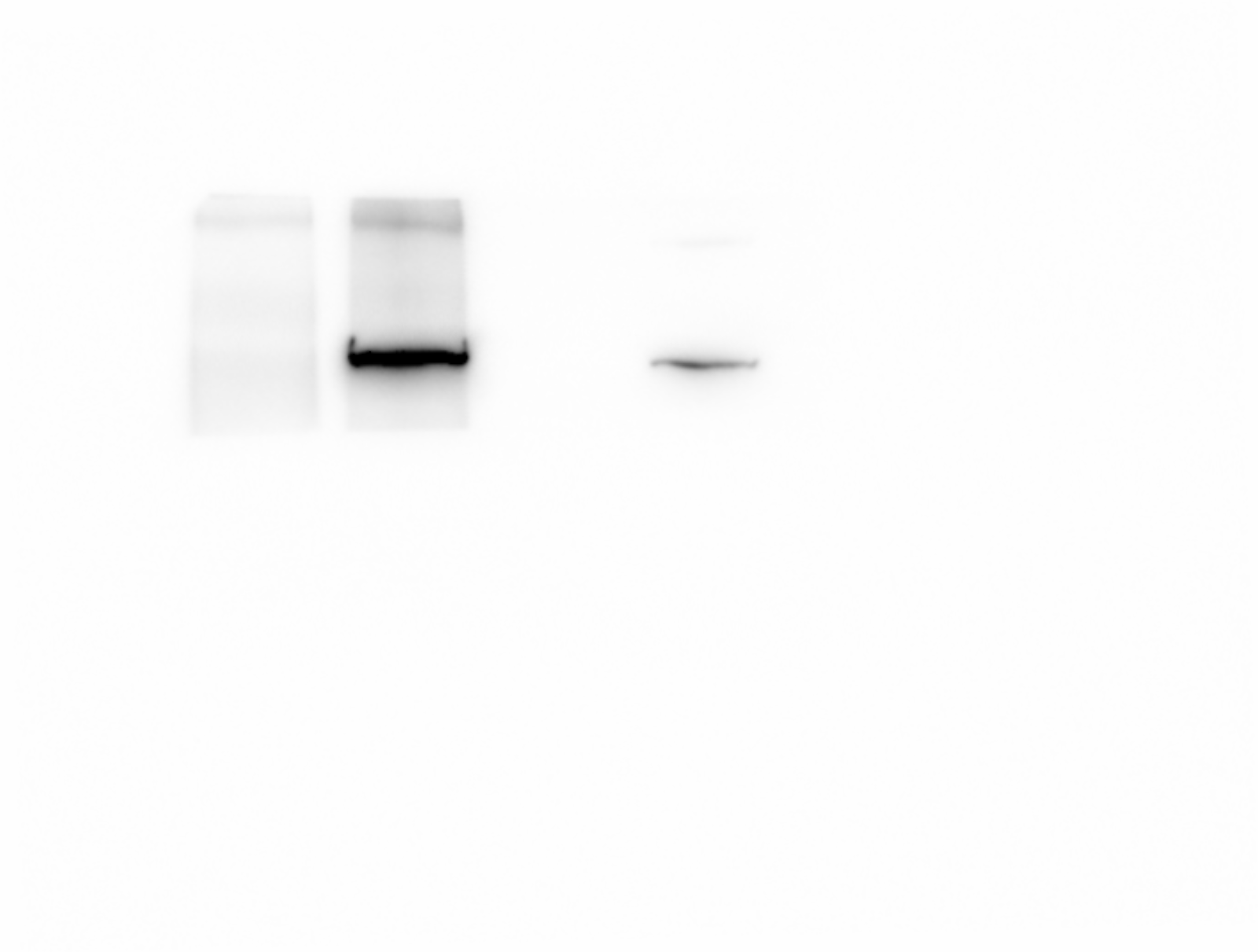

Supplement: Figure 2—source data 3. [file elife-73523-fig2-data3.zip › Raw blots/Input_ anti-BAF155.tif]

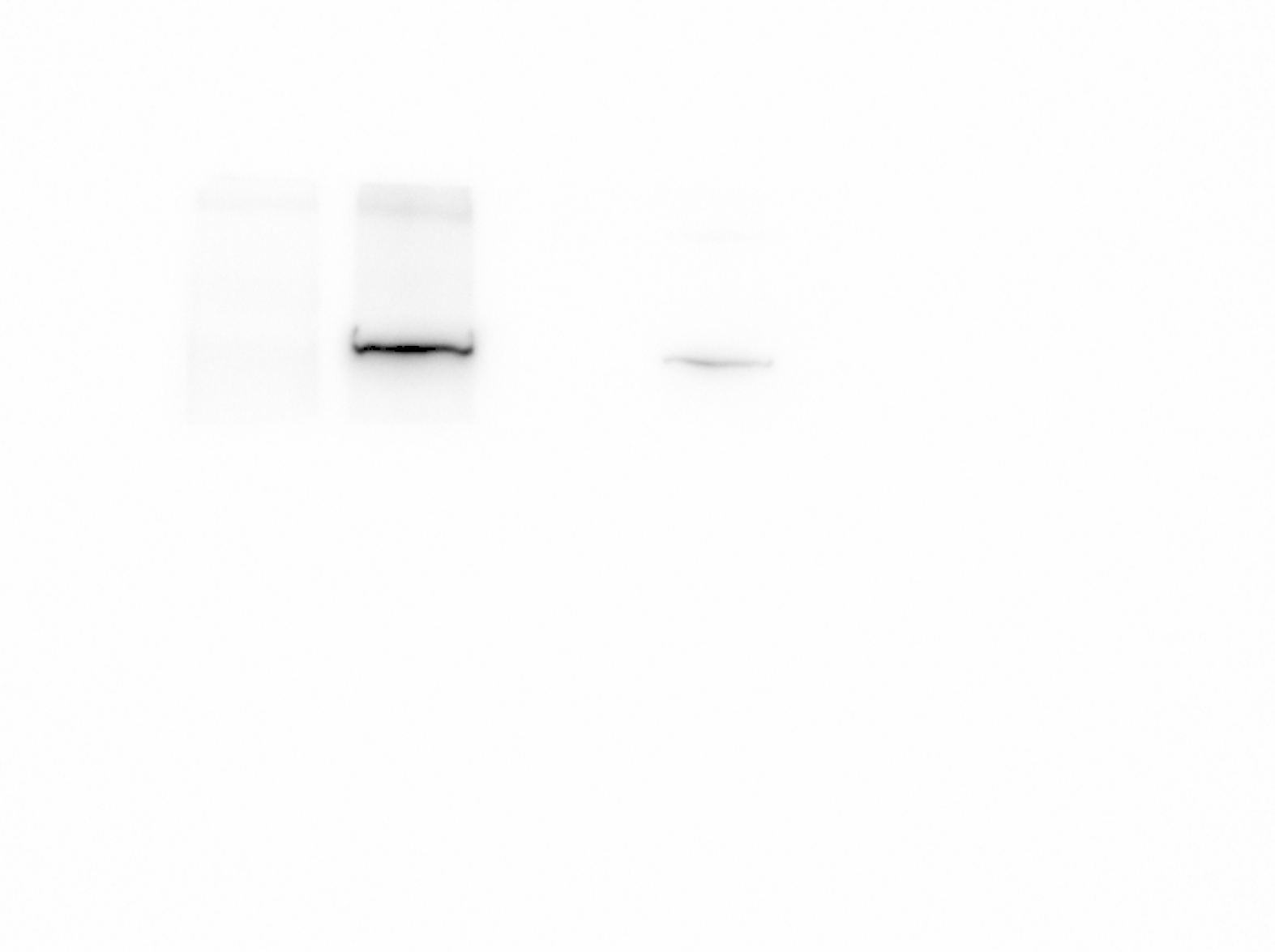

Supplement: Figure 2—source data 3. [file elife-73523-fig2-data3.zip › Raw blots/IP_ anti-BAF155.tif]

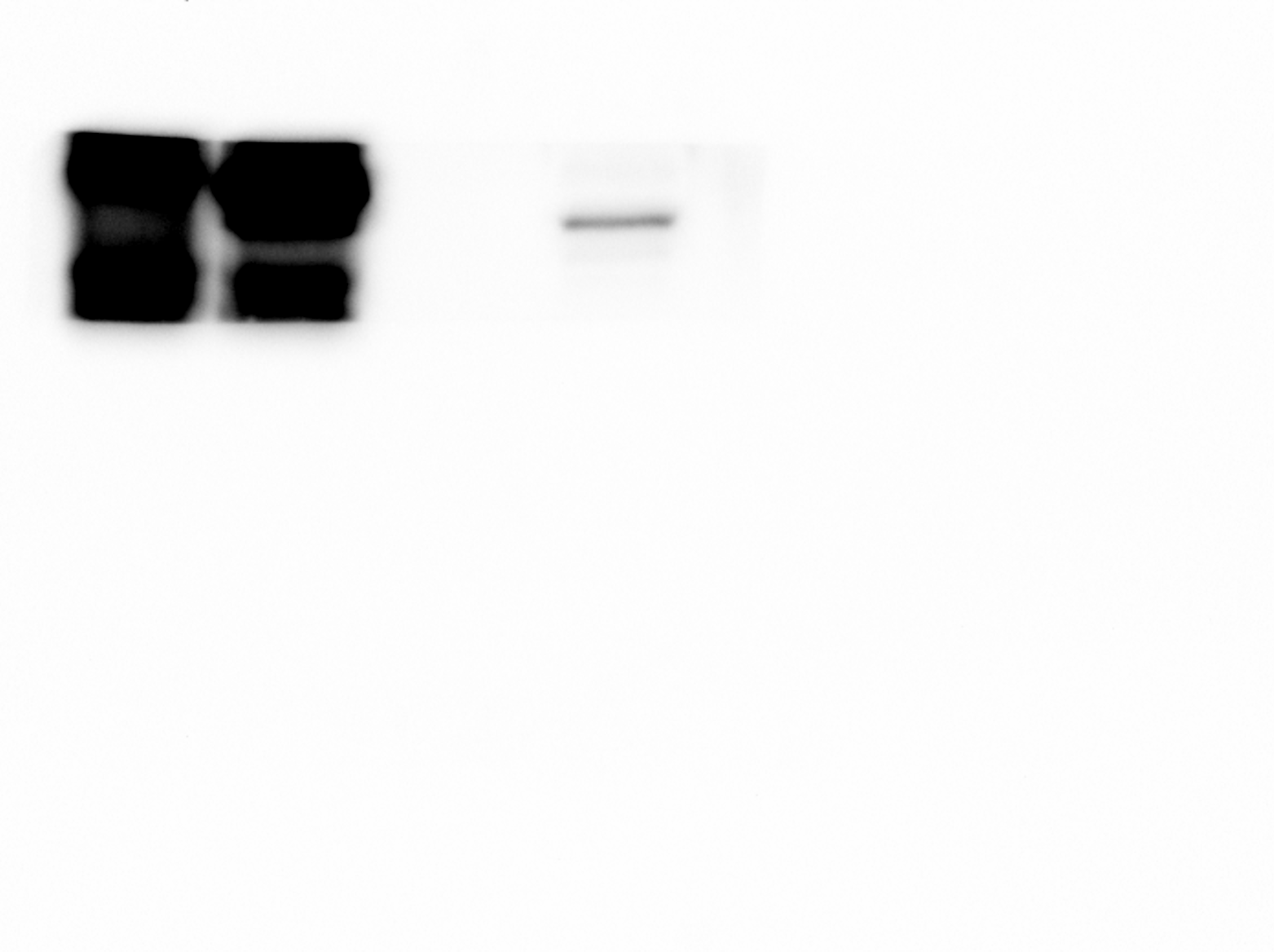

Supplement: Figure 2—source data 3. [file elife-73523-fig2-data3.zip › Raw blots/Input_ anti-IPMK.tif]

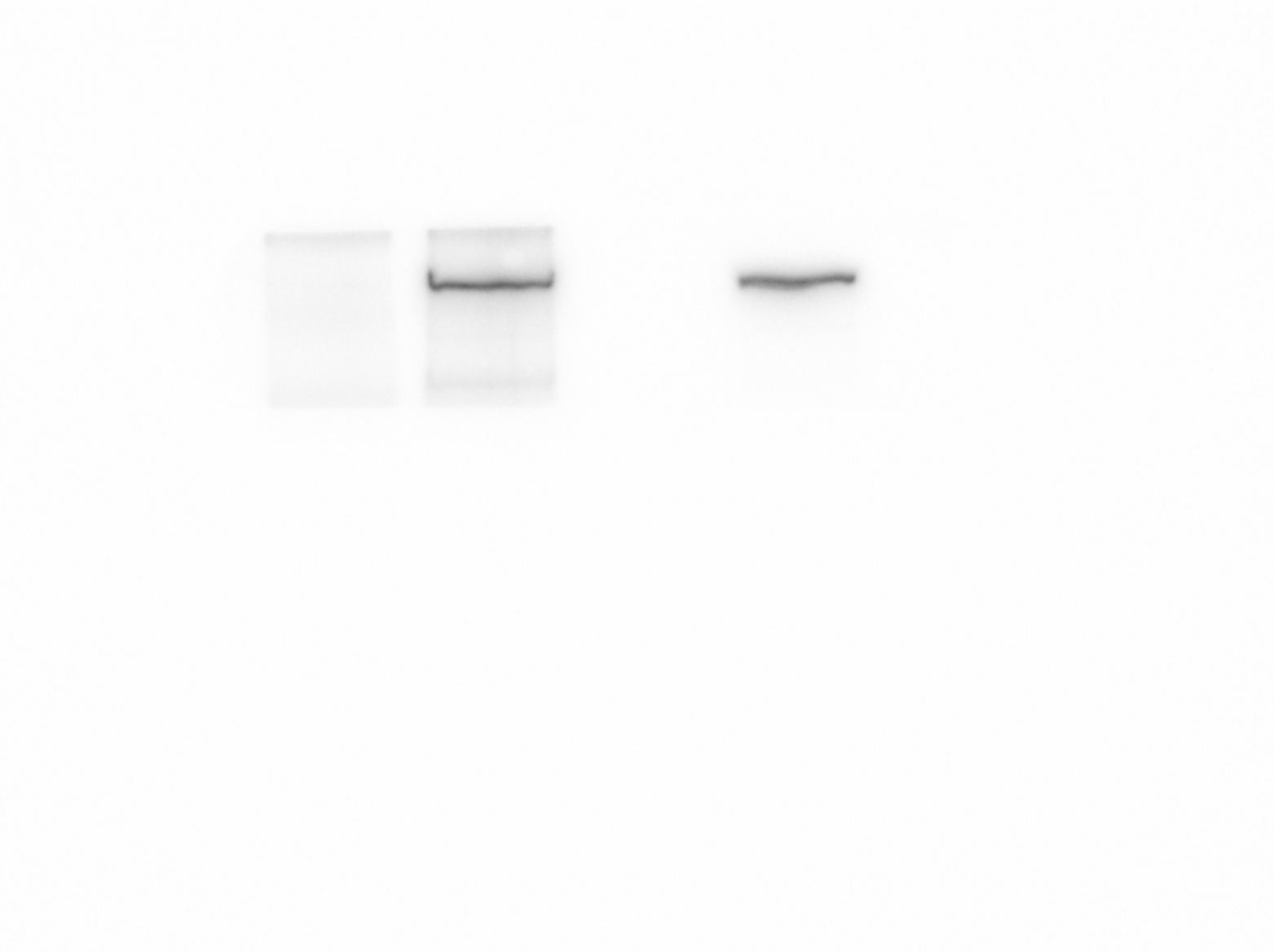

Supplement: Figure 2—source data 3. [file elife-73523-fig2-data3.zip › Raw blots/anti-BRG1.tif]

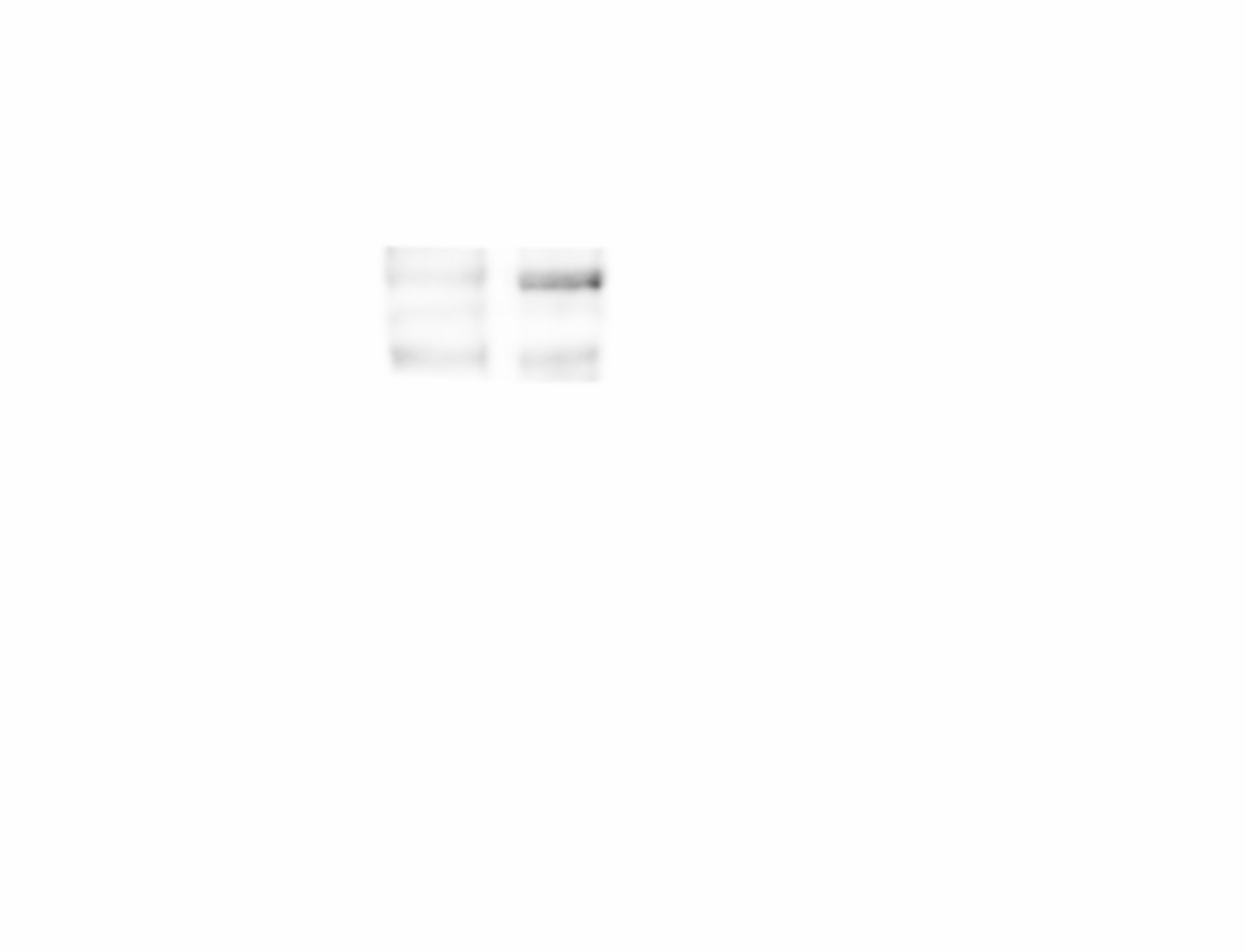

Supplement: Figure 2—source data 3. [file elife-73523-fig2-data3.zip › Raw blots/IP_ anti-SMARCB1.tif]

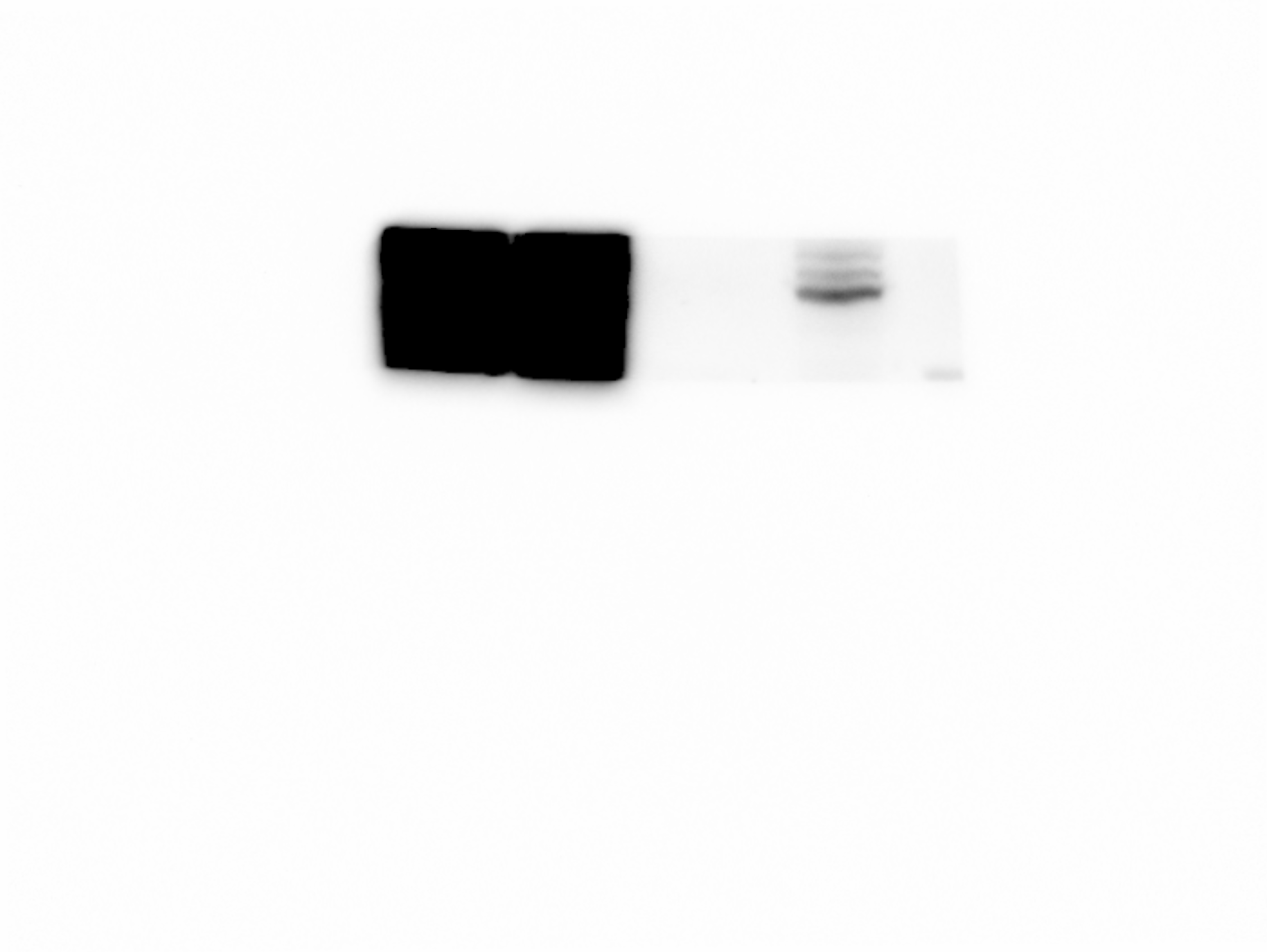

Supplement: Figure 2—source data 3. [file elife-73523-fig2-data3.zip › Raw blots/Input_ anti-SMARCB1.tif]

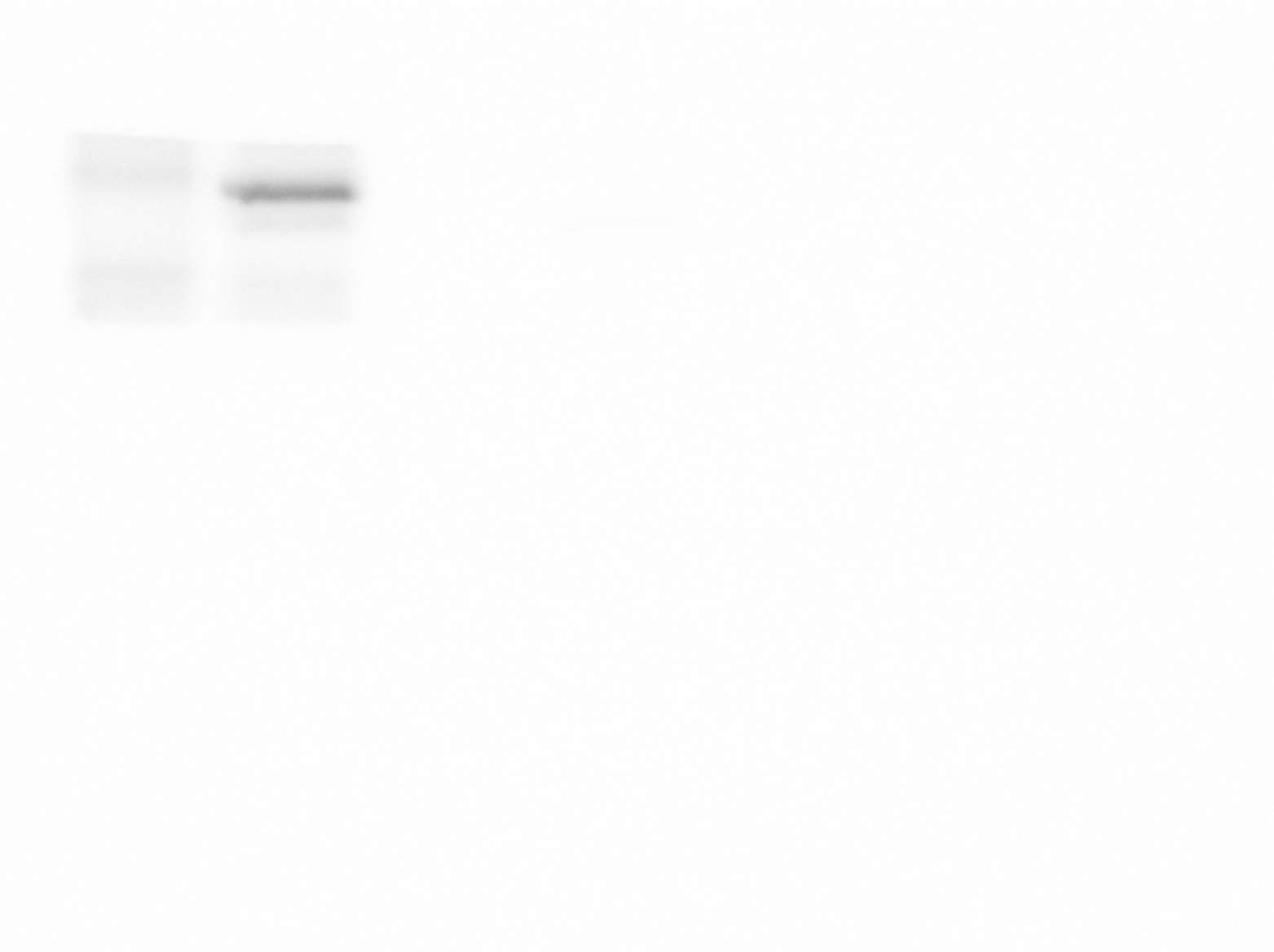

Supplement: Figure 2—source data 3. [file elife-73523-fig2-data3.zip › Raw blots/IP_ anti-IPMK.tif]

IP : IgG IPMK

Input

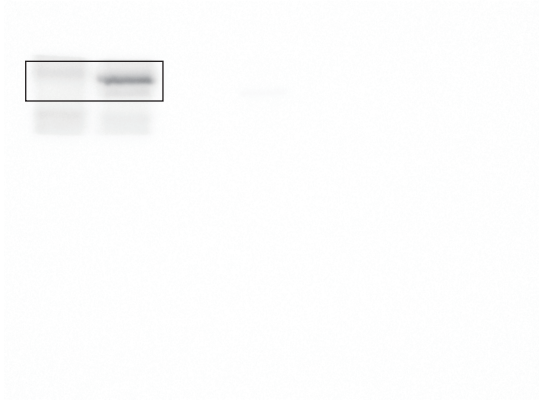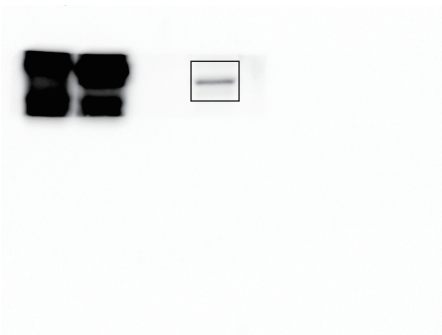

IPMK

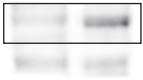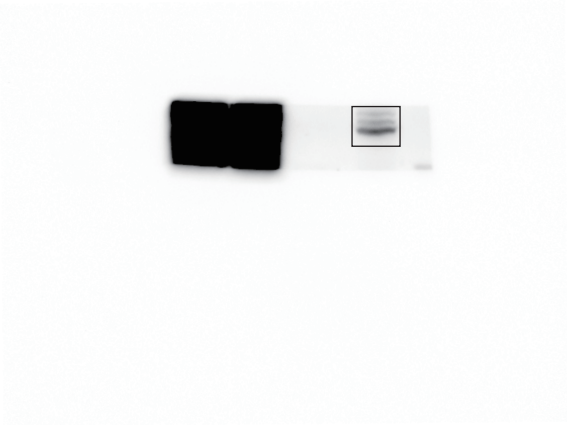

SMARCB1

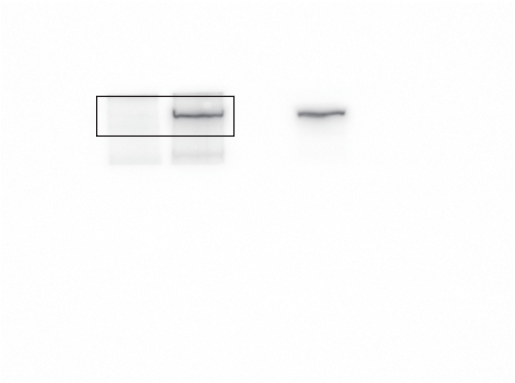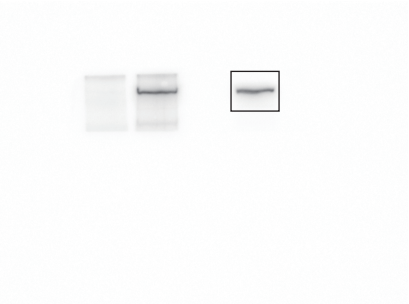

BRG1

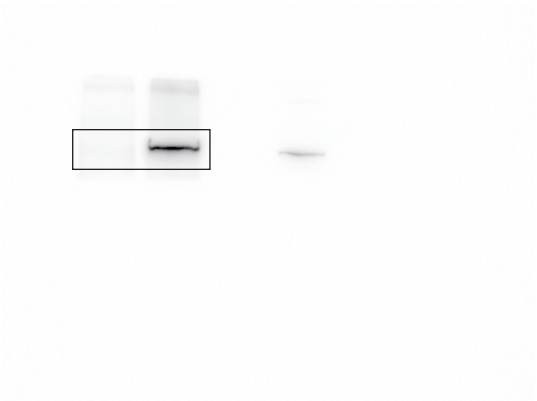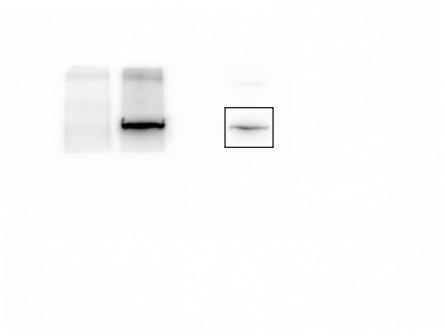

BAF155

Supplement: Figure 2—source data 3. [file elife-73523-fig2-data3.zip › Labelled blots.pdf]

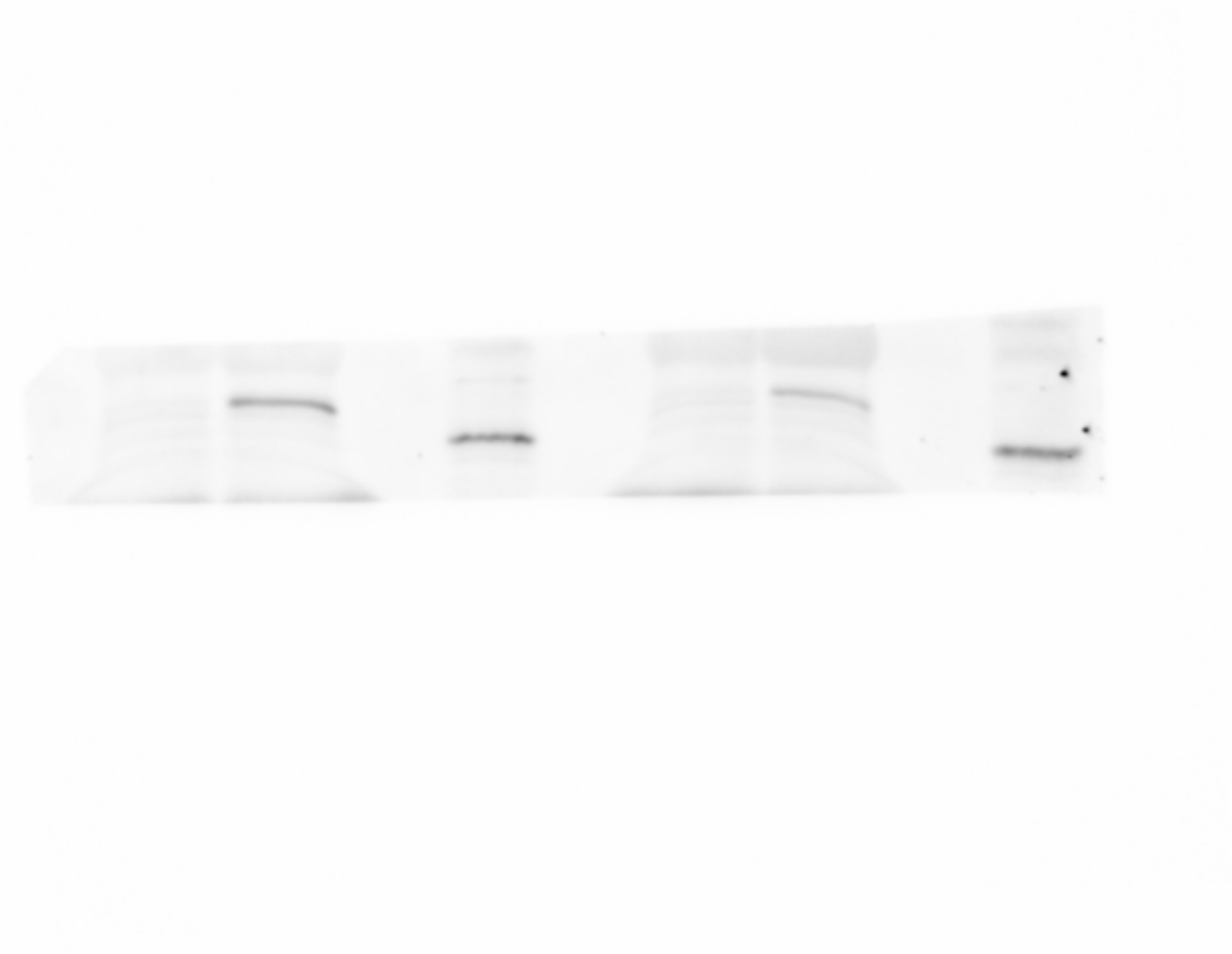

Supplement: Figure 2—source data 4. [file elife-73523-fig2-data4.zip › Raw blots/anti-IPMK.tif]

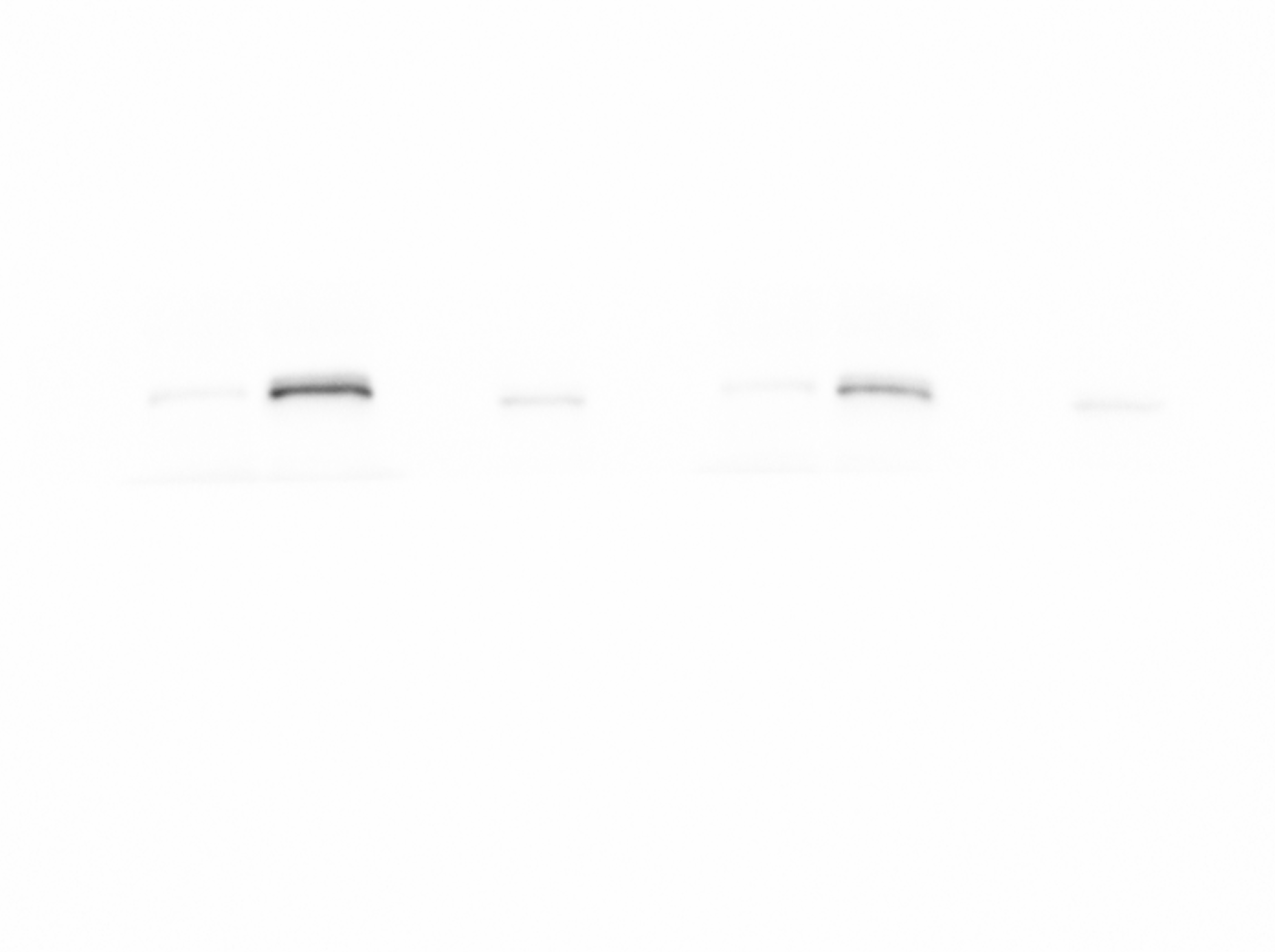

Supplement: Figure 2—source data 4. [file elife-73523-fig2-data4.zip › Raw blots/anti-SMARCB1.tif]

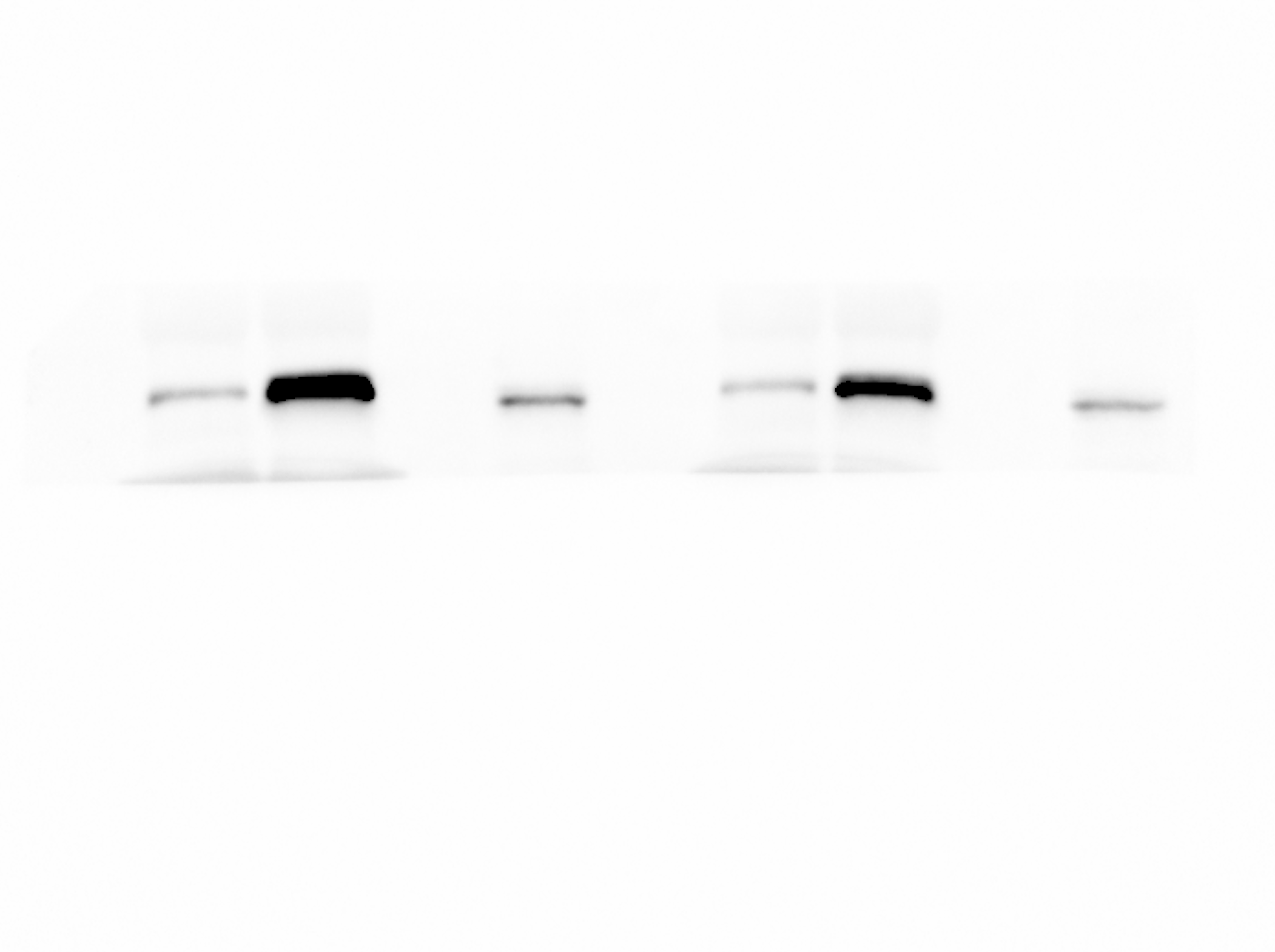

Supplement: Figure 2—source data 4. [file elife-73523-fig2-data4.zip › Raw blots/Input_ anti-SMARCB1.tif]

D

IP : IgG SMARCB1

Input

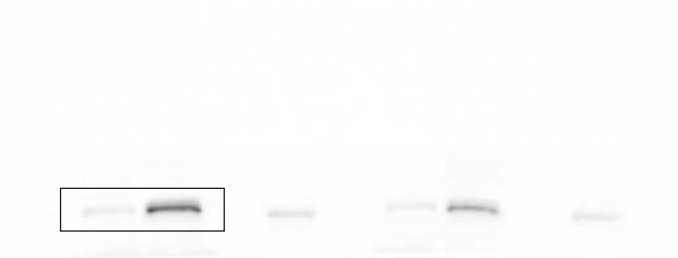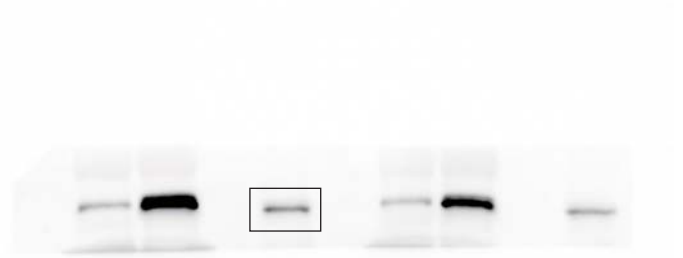

SMARCB1

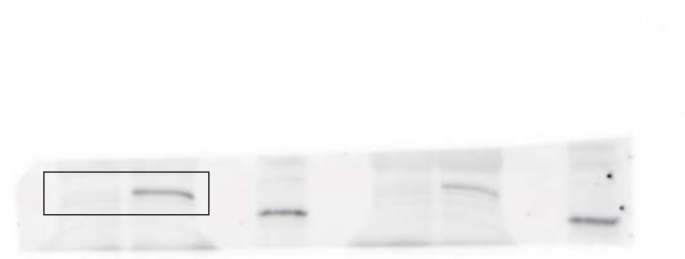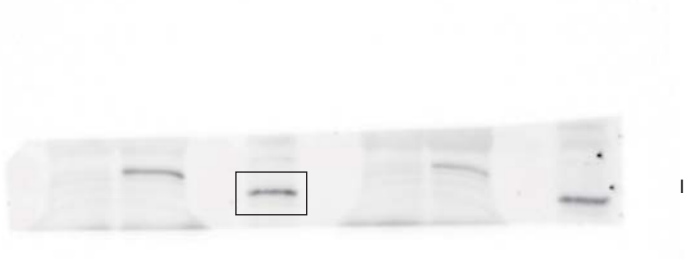

IPMK

Supplement: Figure 2—source data 4. [file elife-73523-fig2-data4.zip › Labelled blots.pdf]

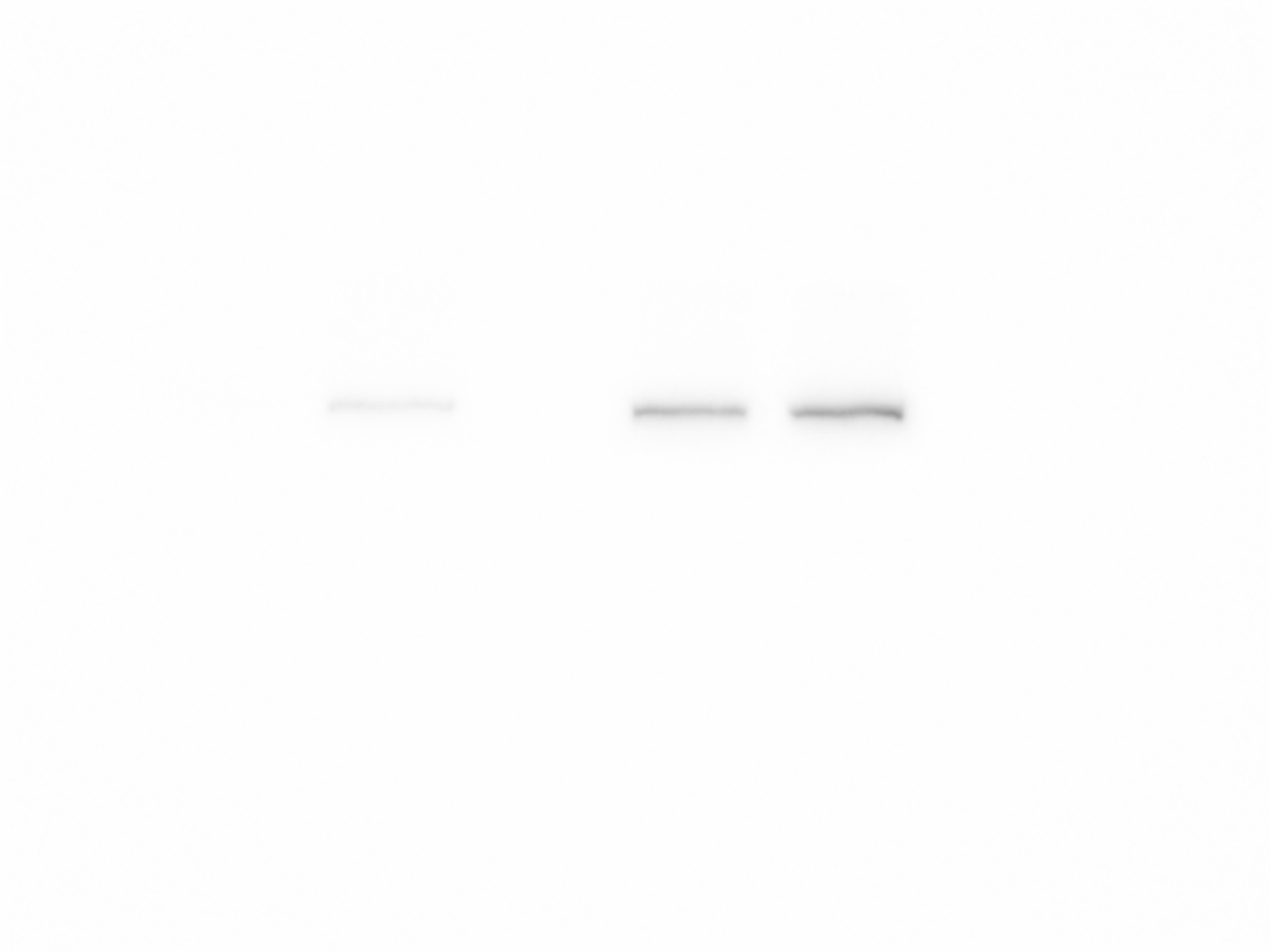

Supplement: Figure 2—source data 5. [file elife-73523-fig2-data5.zip › Raw blots/Input_ anti-BRG1.tif]

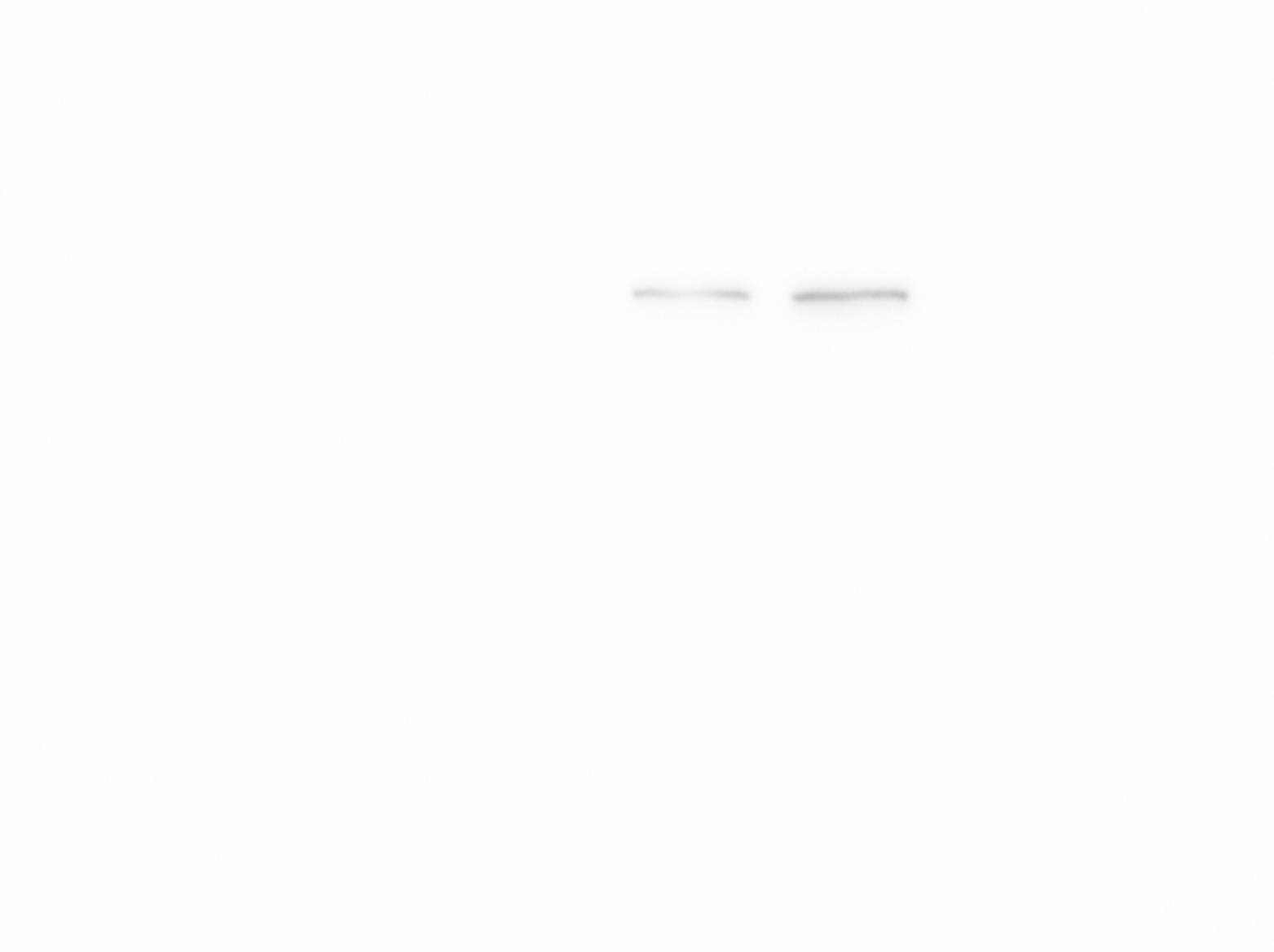

Supplement: Figure 2—source data 5. [file elife-73523-fig2-data5.zip › Raw blots/Input_ anti-BAF155.tif]

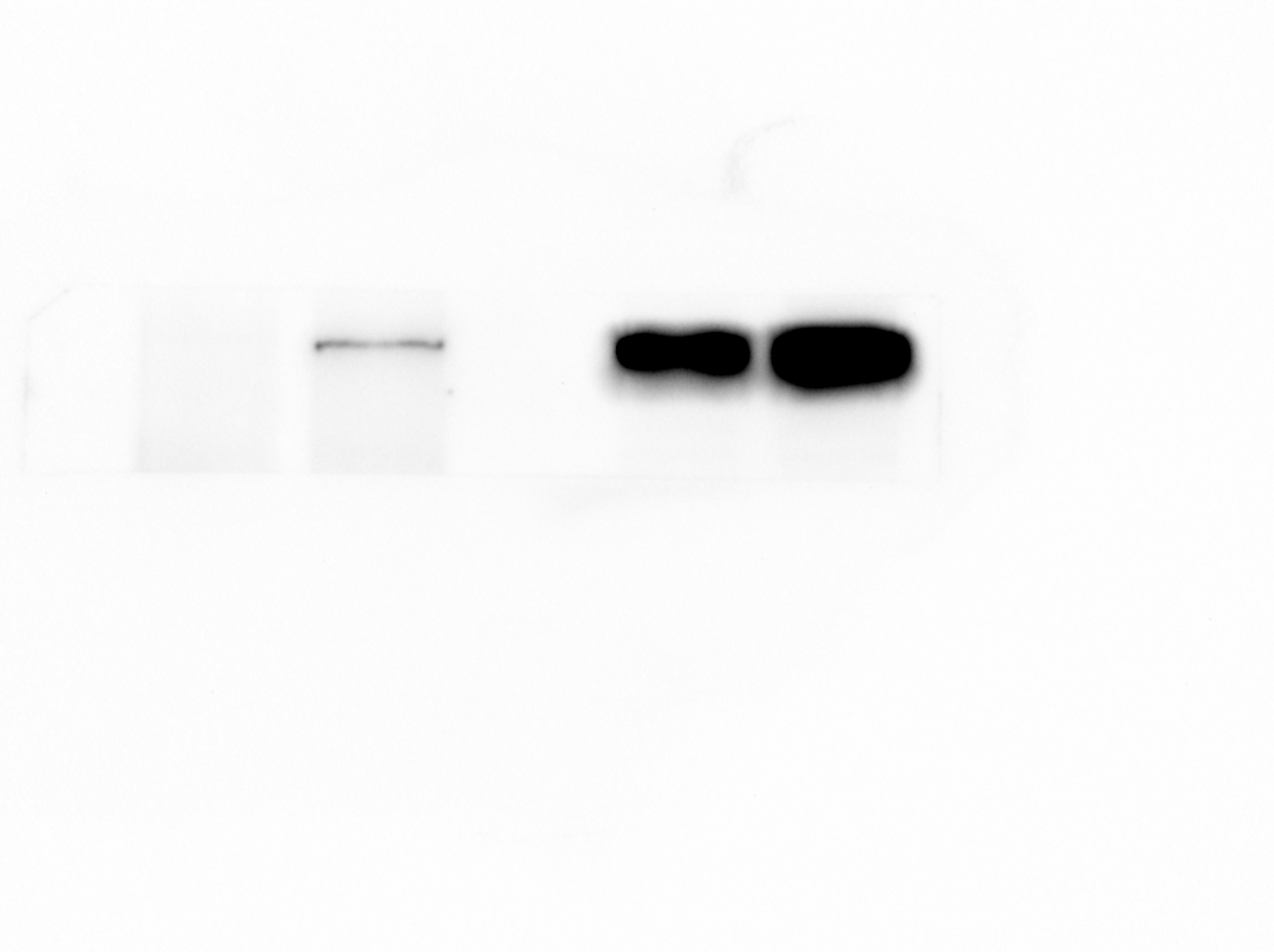

Supplement: Figure 2—source data 5. [file elife-73523-fig2-data5.zip › Raw blots/IP_ anti-BAF155.tif]

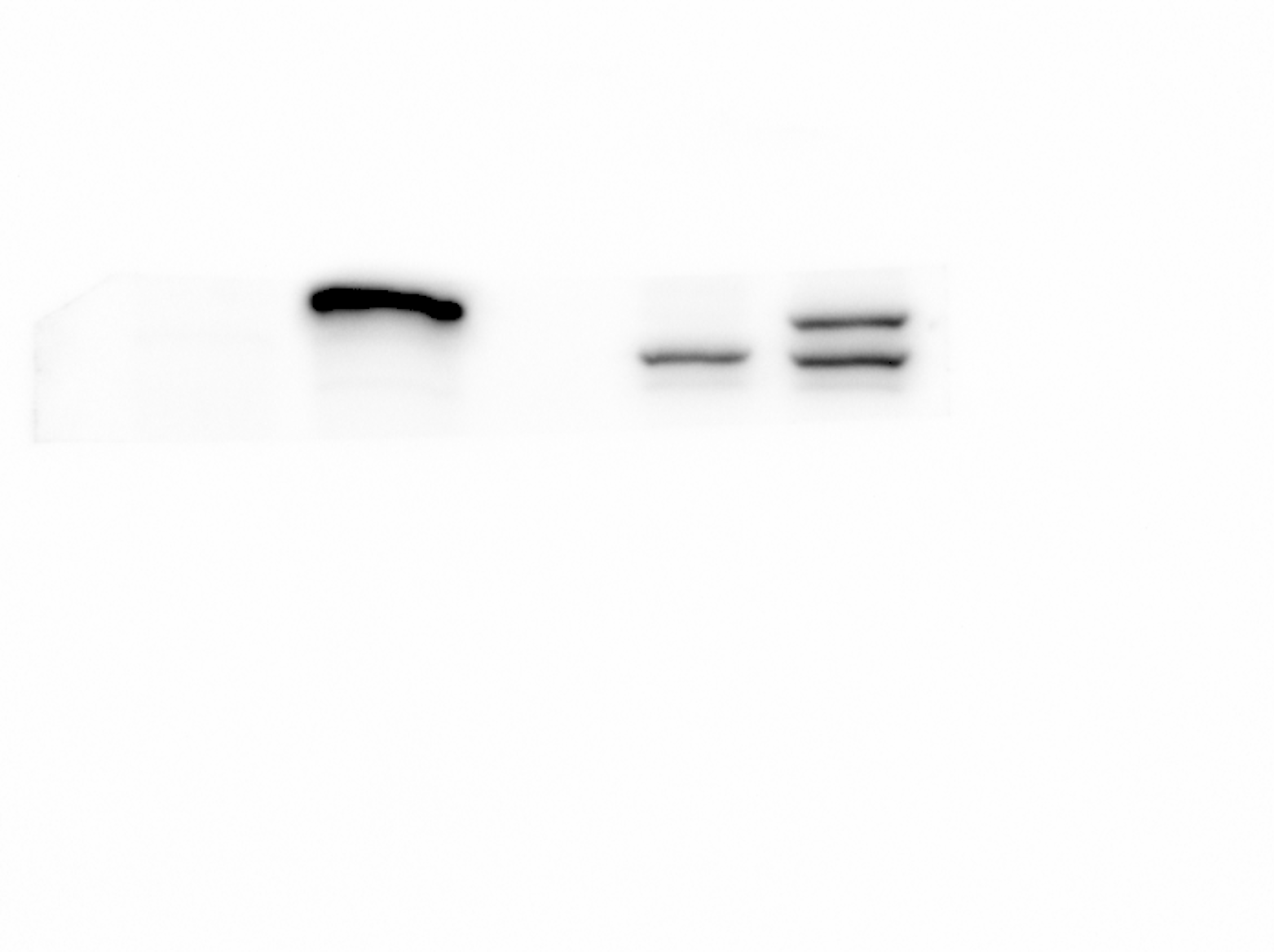

Supplement: Figure 2—source data 5. [file elife-73523-fig2-data5.zip › Raw blots/Input_ anti-IPMK.tif]

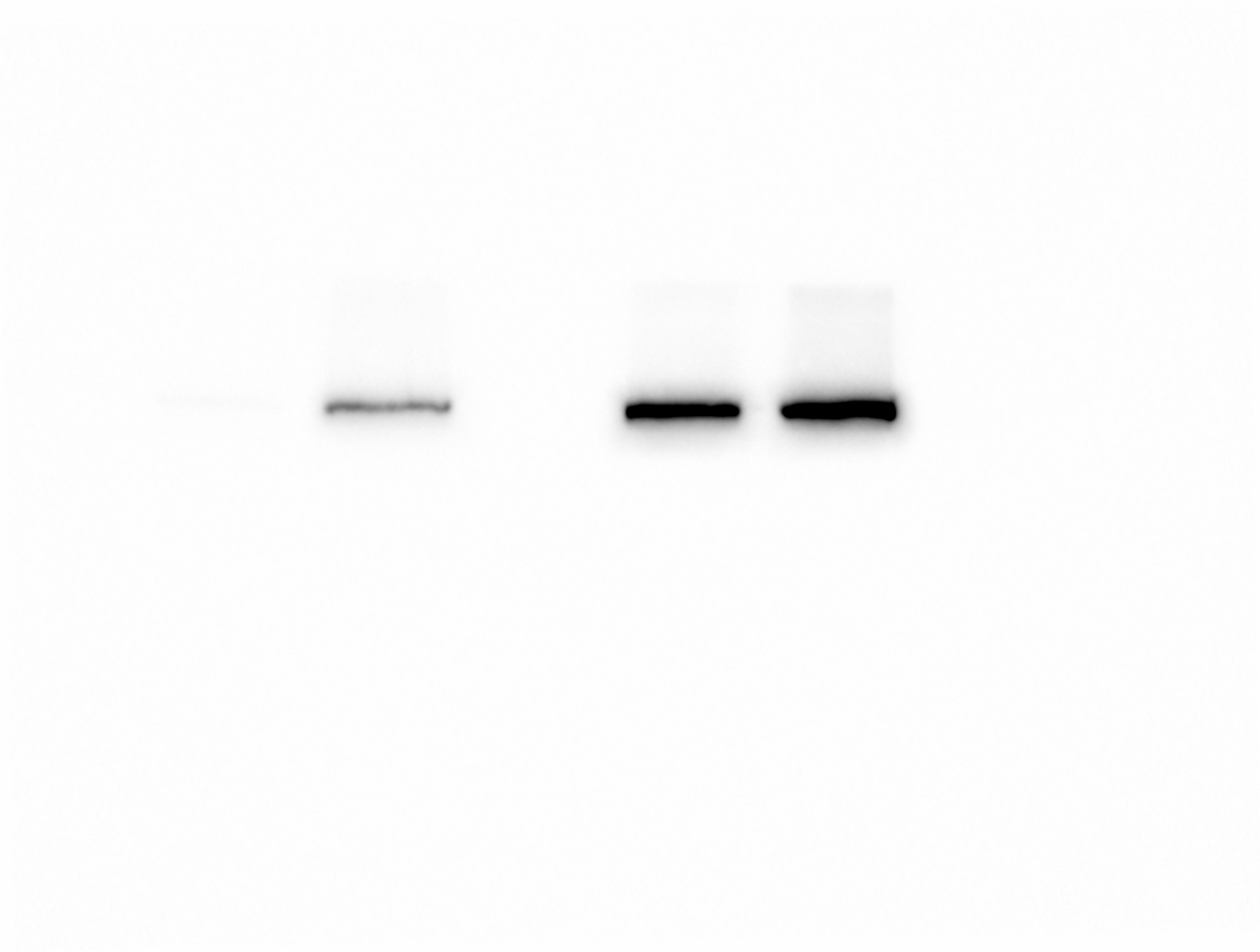

Supplement: Figure 2—source data 5. [file elife-73523-fig2-data5.zip › Raw blots/IP_ anti-BRG1.tif]

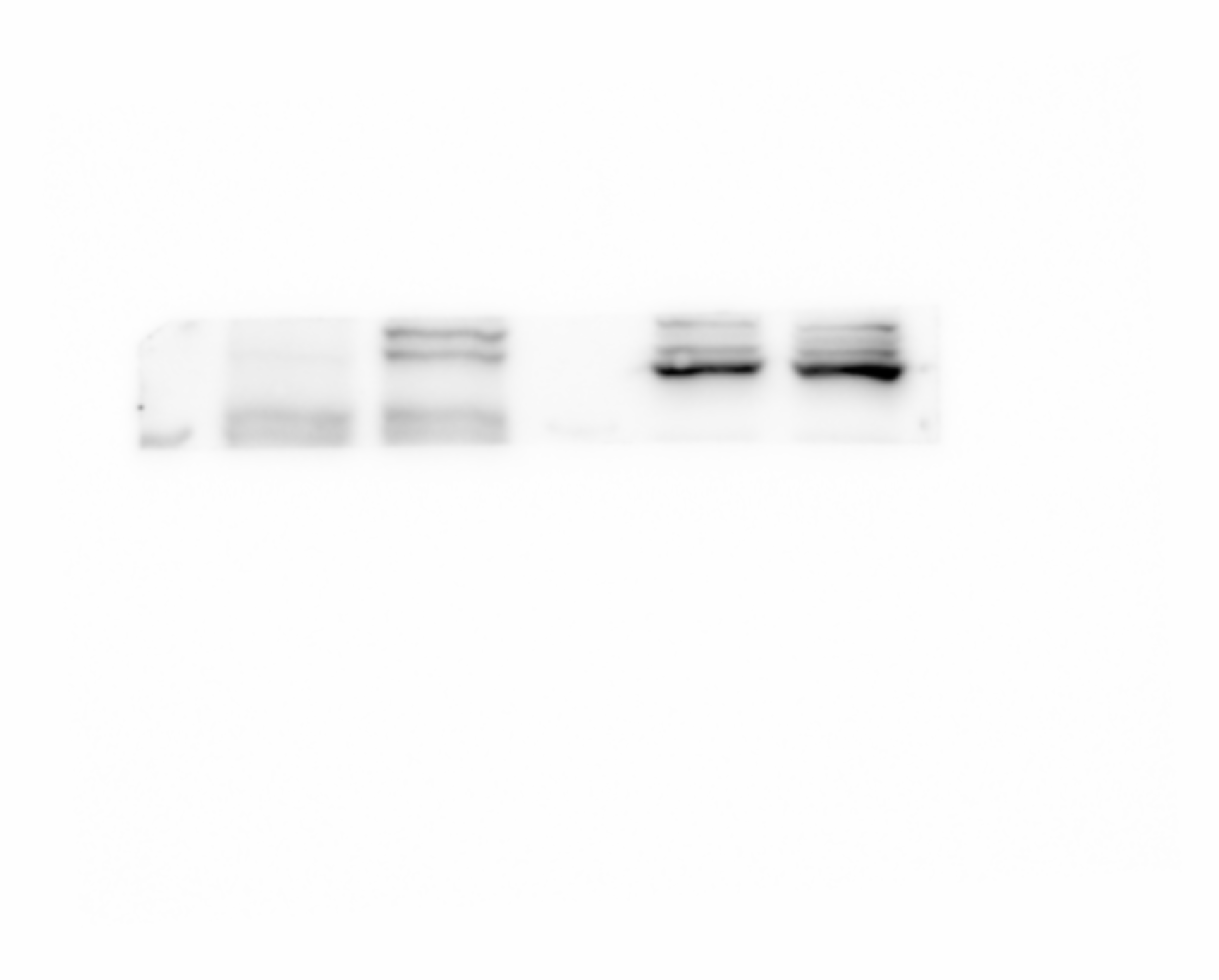

Supplement: Figure 2—source data 5. [file elife-73523-fig2-data5.zip › Raw blots/IP_ anti-SMARCB1.tif]

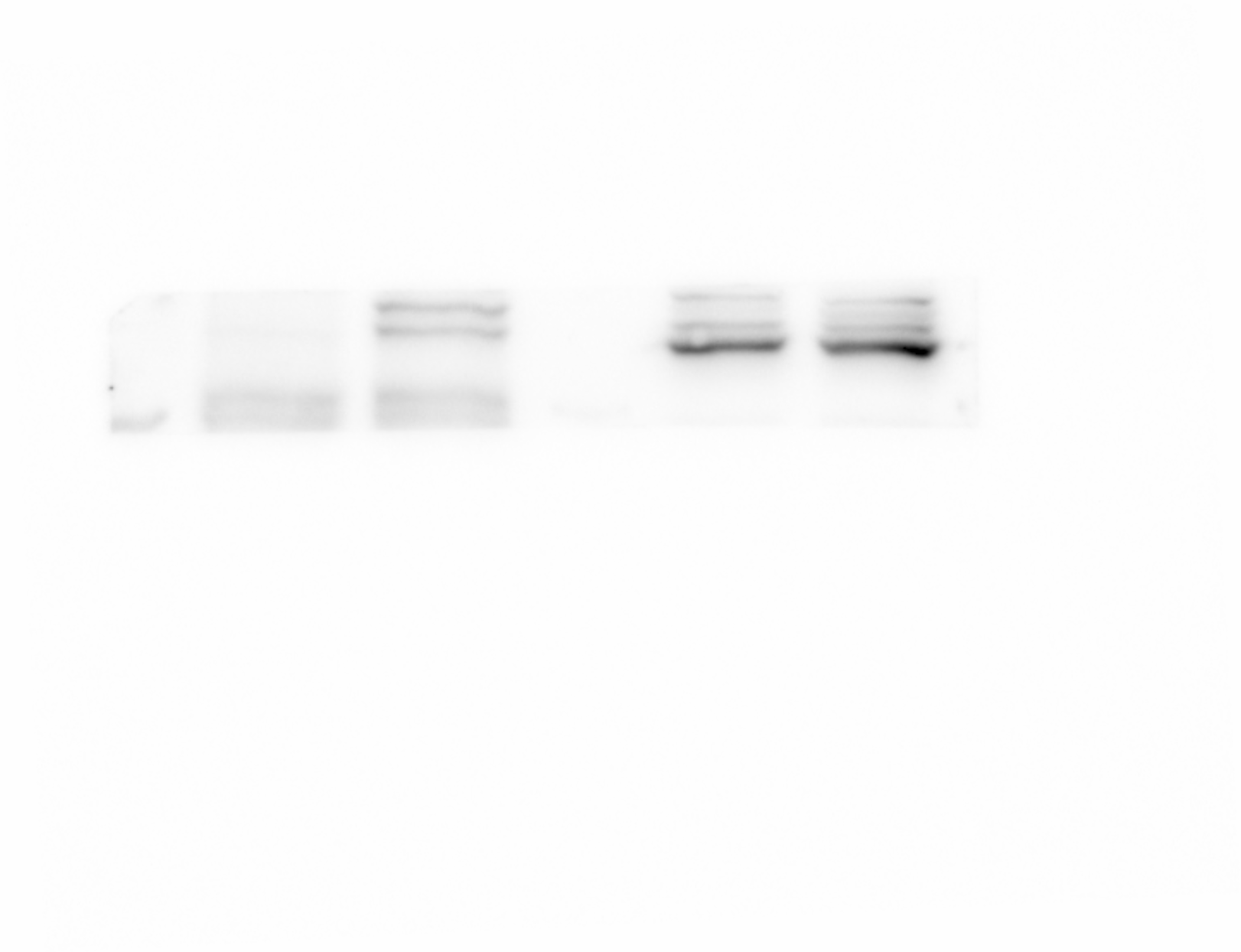

Supplement: Figure 2—source data 5. [file elife-73523-fig2-data5.zip › Raw blots/Input_ anti-SMARCB1.tif]

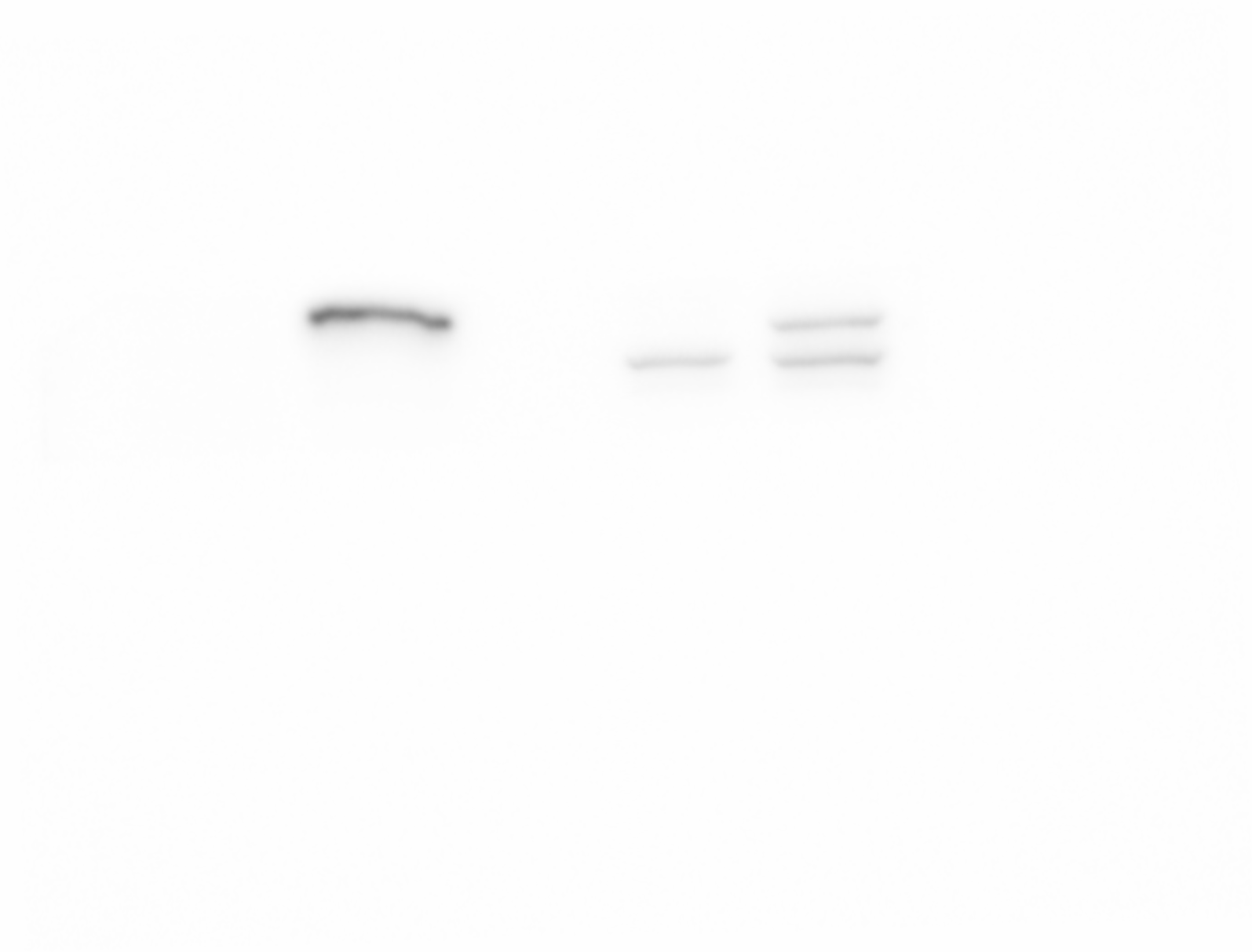

Supplement: Figure 2—source data 5. [file elife-73523-fig2-data5.zip › Raw blots/IP_ anti-IPMK.tif]

**E**

IP : FLAG

FLAG

FLAG-IPMK

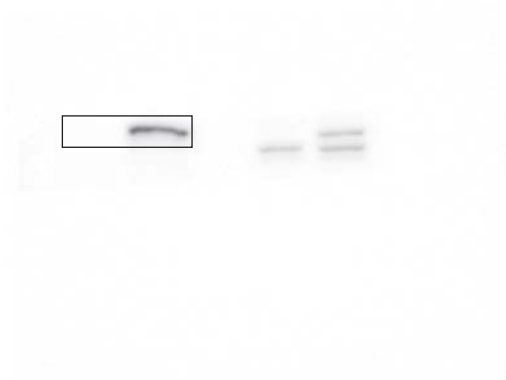

Input

FLAG

FLAG-IPMK

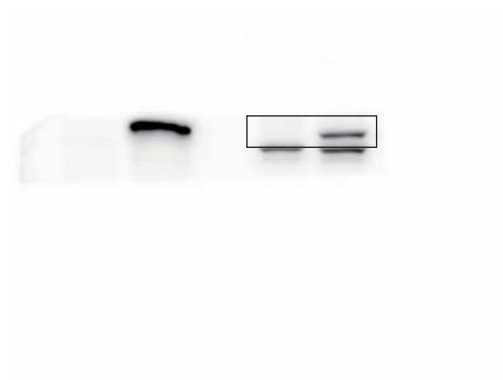

IPMK

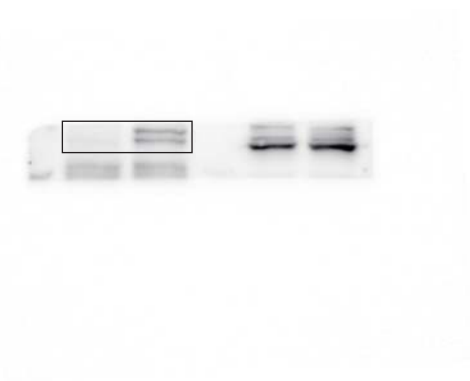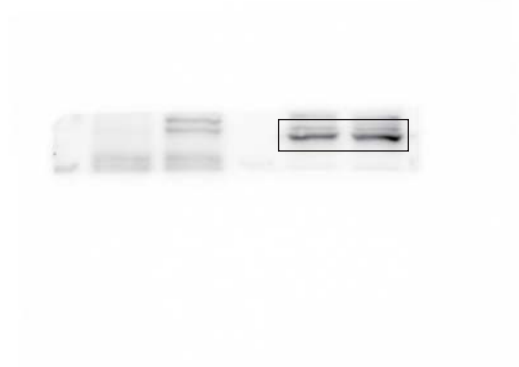

SMARCB1

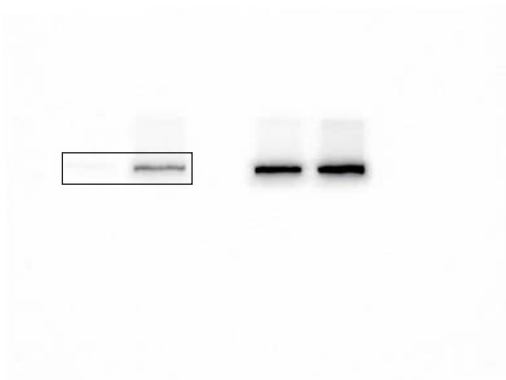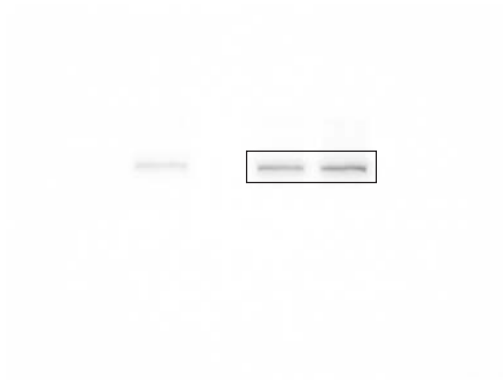

BRG1

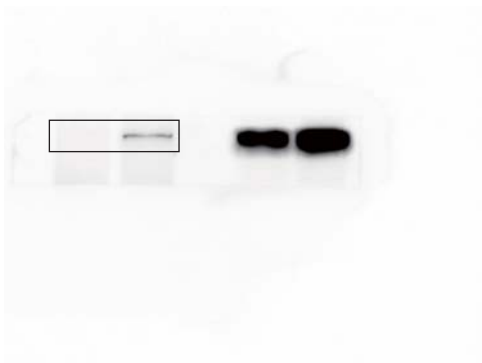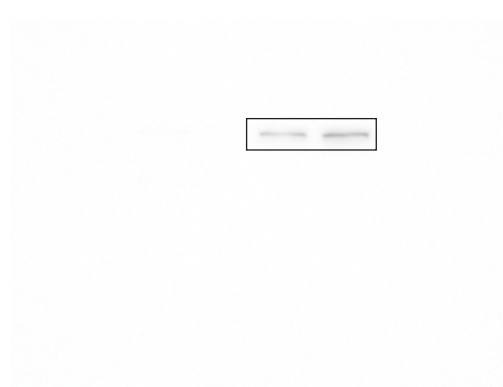

BAF155

Supplement: Figure 2—source data 5. [file elife-73523-fig2-data5.zip › Labelled blots.pdf]

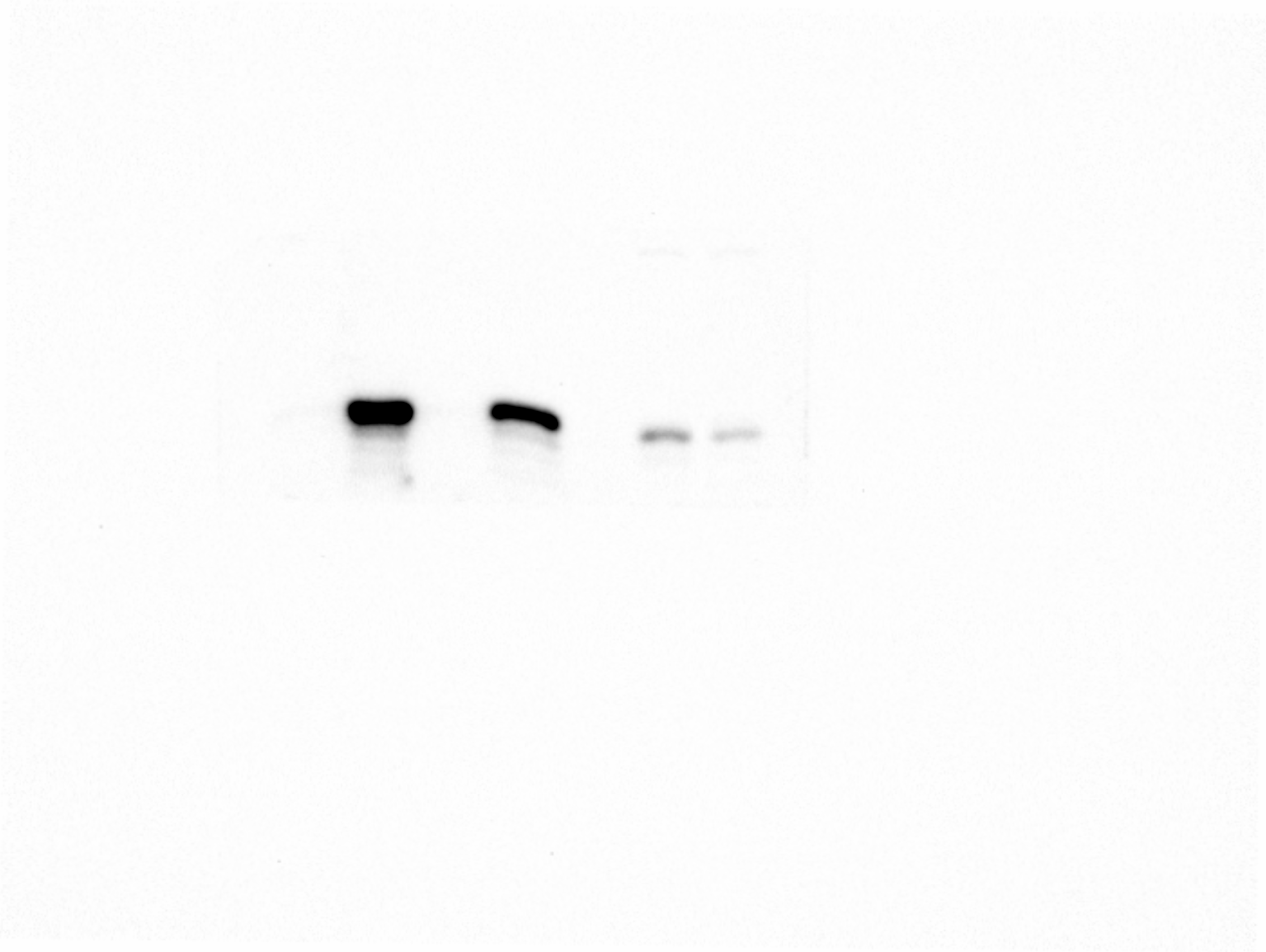

Supplement: Figure 2—source data 6. [file elife-73523-fig2-data6.zip › Raw blots/Input_ anti-IPMK.tif]

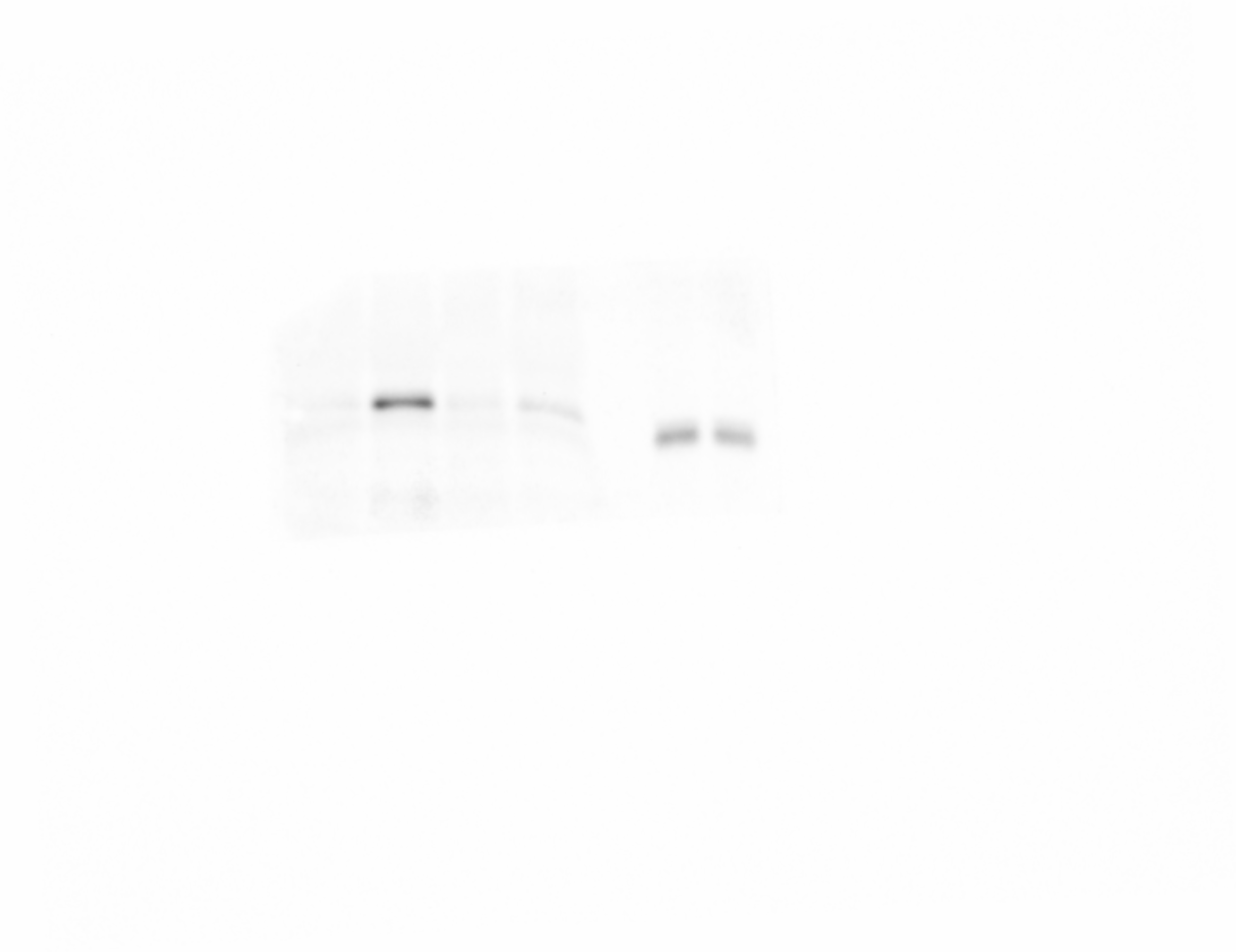

Supplement: Figure 2—source data 6. [file elife-73523-fig2-data6.zip › Raw blots/IP_ anti-SMARCB1.tif]

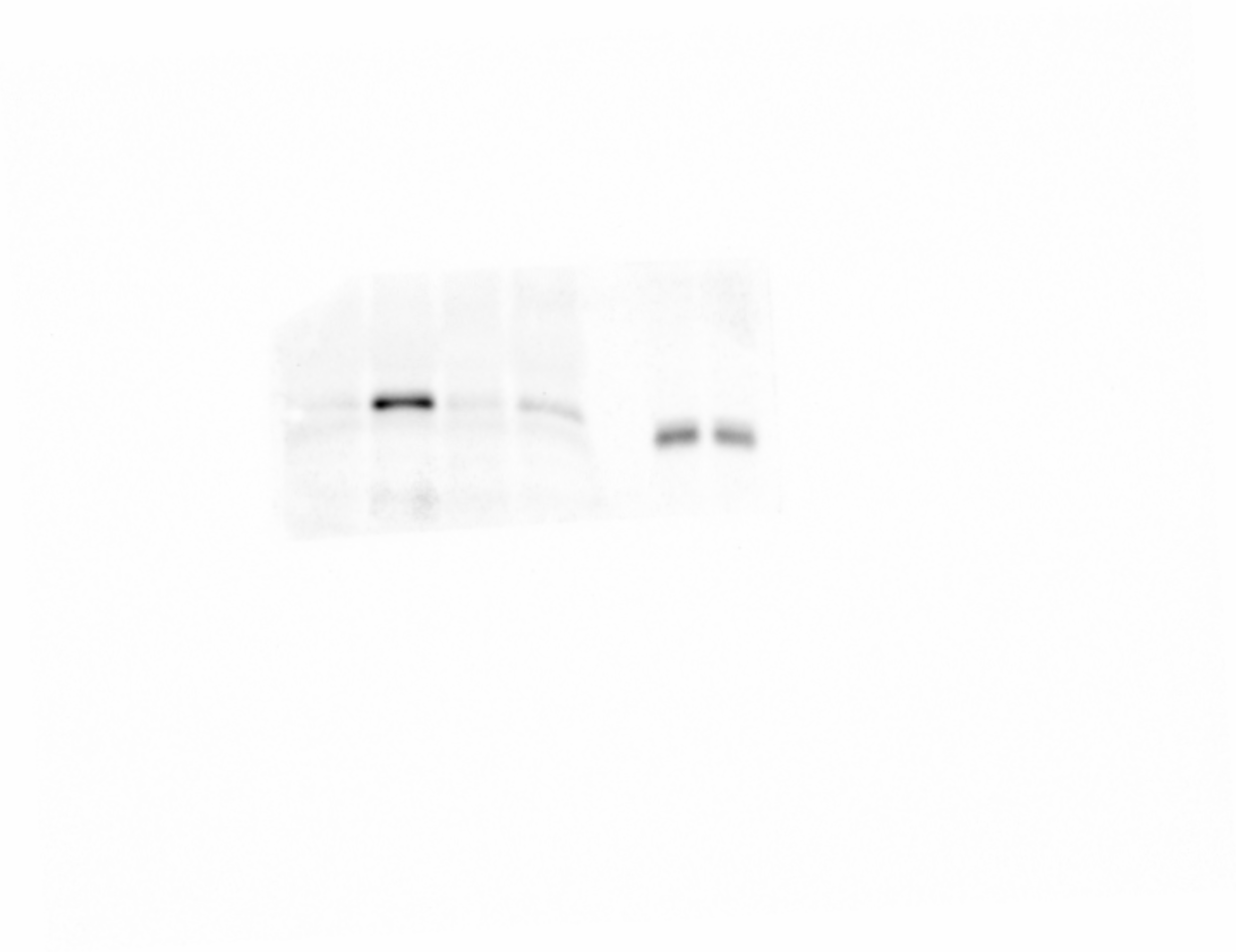

Supplement: Figure 2—source data 6. [file elife-73523-fig2-data6.zip › Raw blots/Input_ anti-SMARCB1.tif]

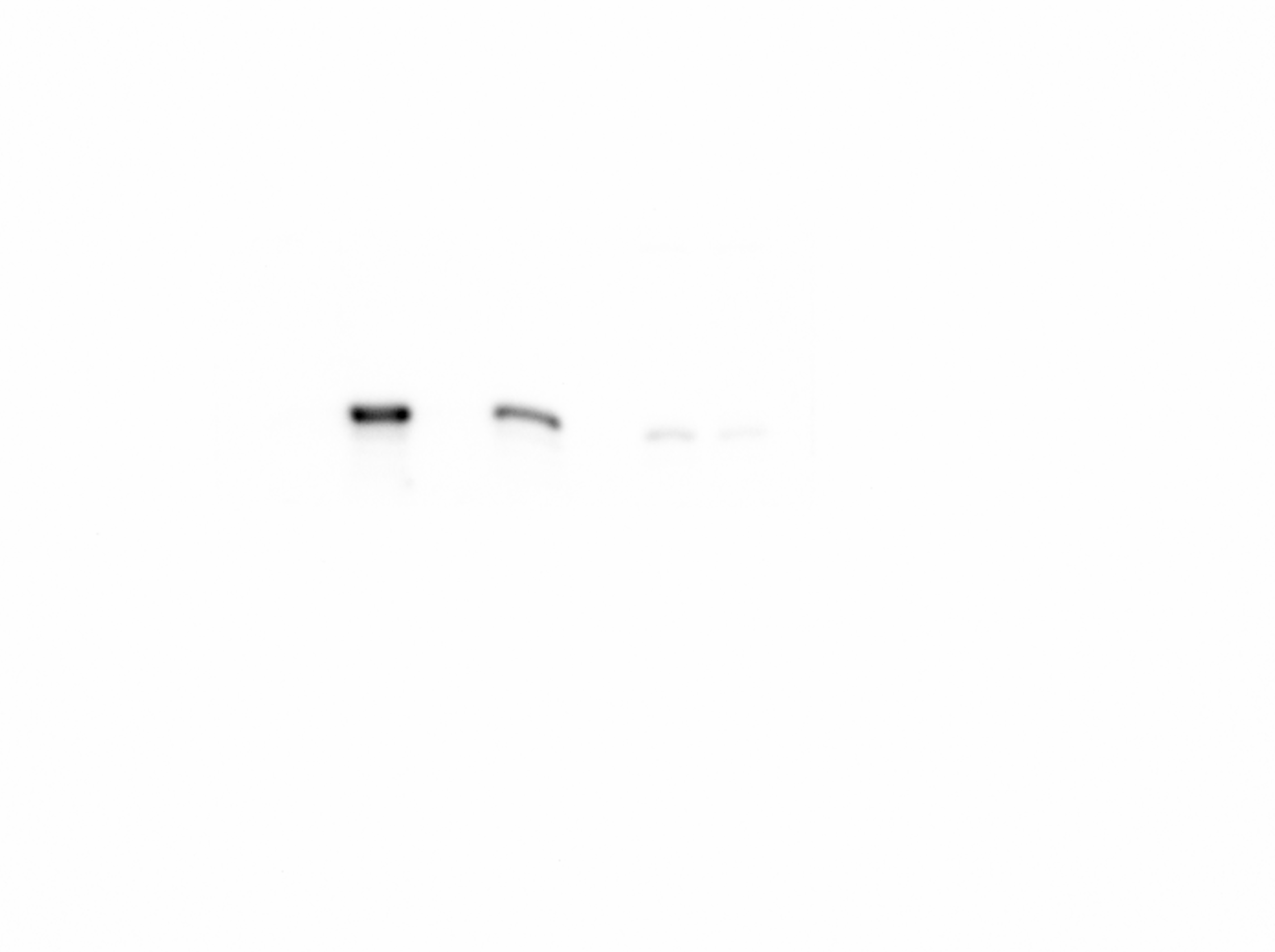

Supplement: Figure 2—source data 6. [file elife-73523-fig2-data6.zip › Raw blots/IP_ anti-IPMK.tif]

**F**

IP :    IgG    IPMK    IgG    IPMK  
         Control   Control   *Ipmk*KD   *Ipmk*KD

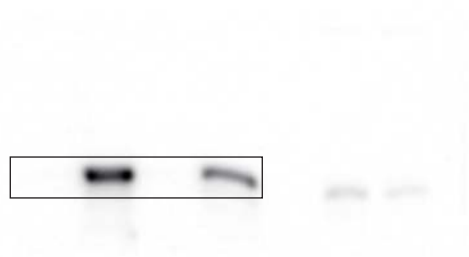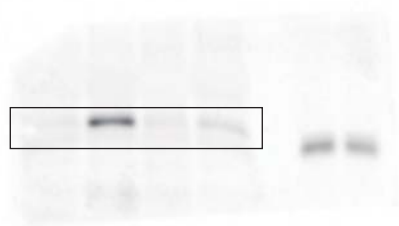

Input  
Control   *Ipmk*KD

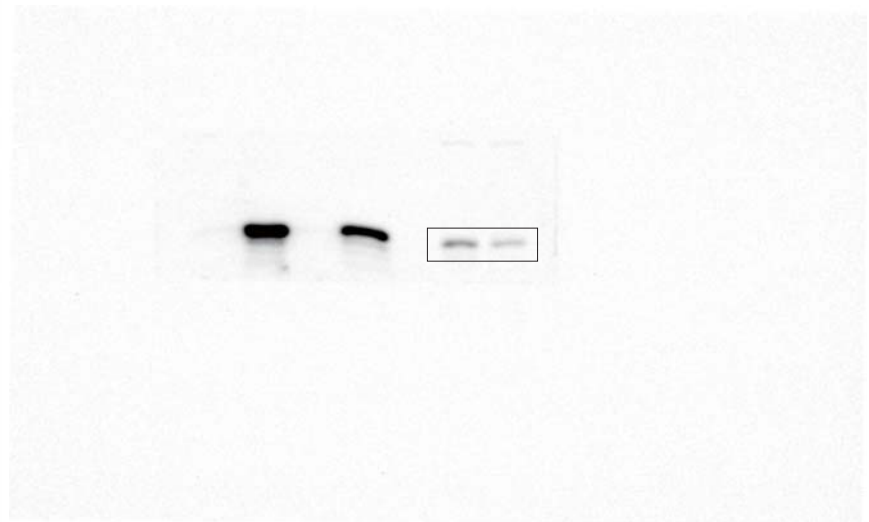

IPMK

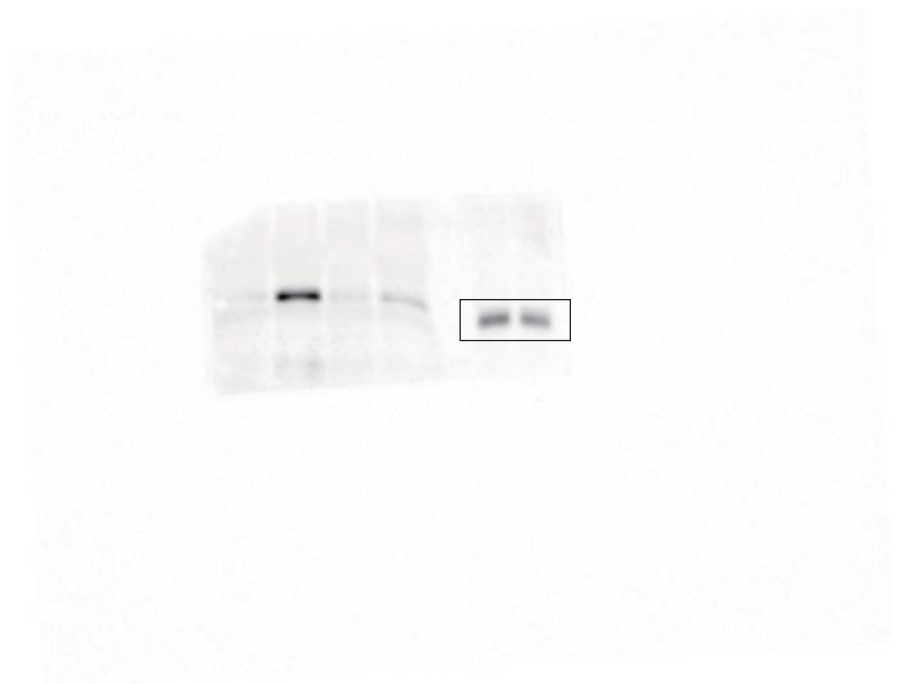

SMARCB1

Supplement: Figure 2—source data 6. [file elife-73523-fig2-data6.zip › Labelled blots.pdf]

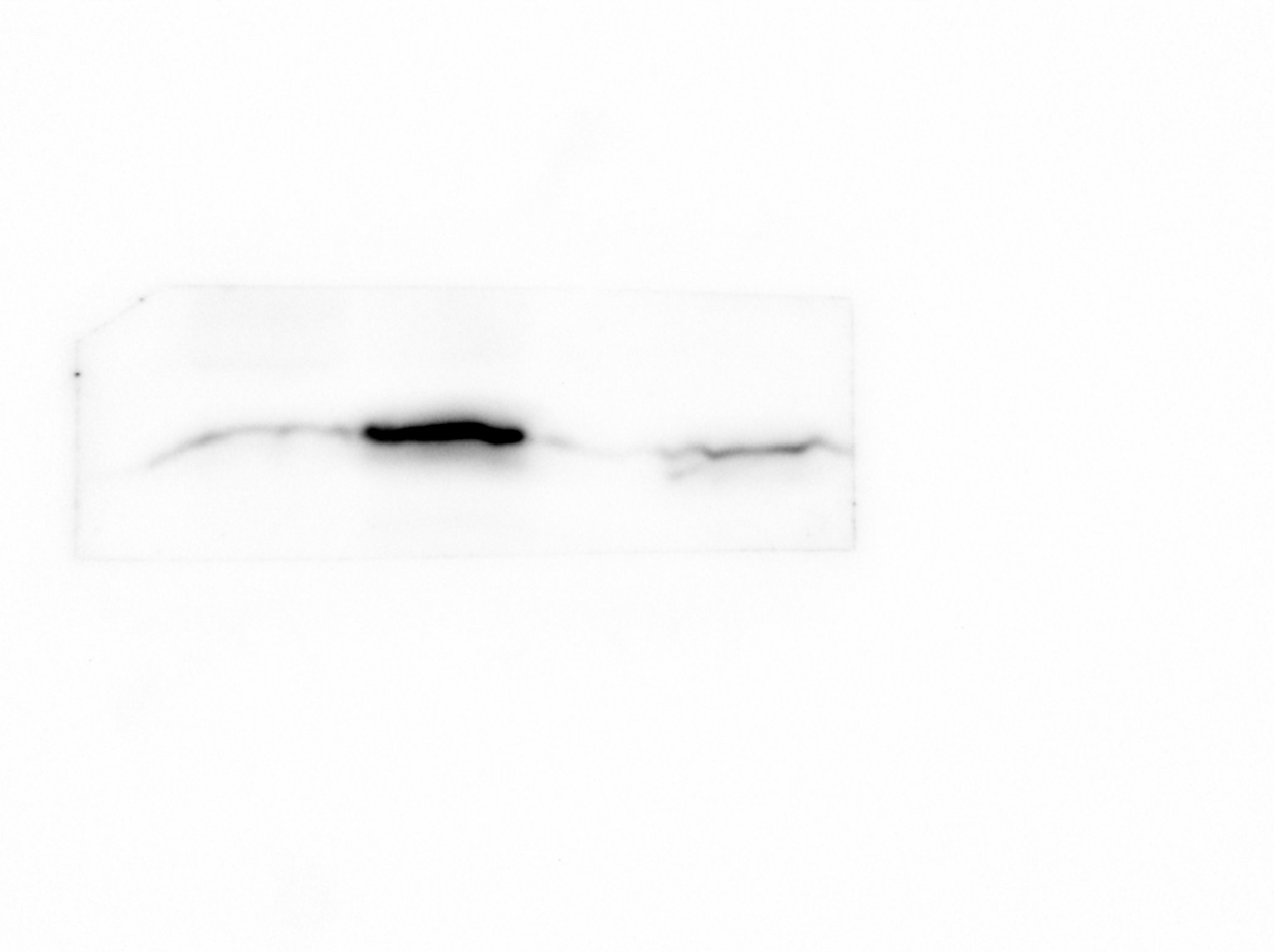

Supplement: Figure 2—figure supplement 1—source data 1. [file elife-73523-fig2-figsupp1-data1.zip › Raw blots/Input_ anti-IPMK.tif]

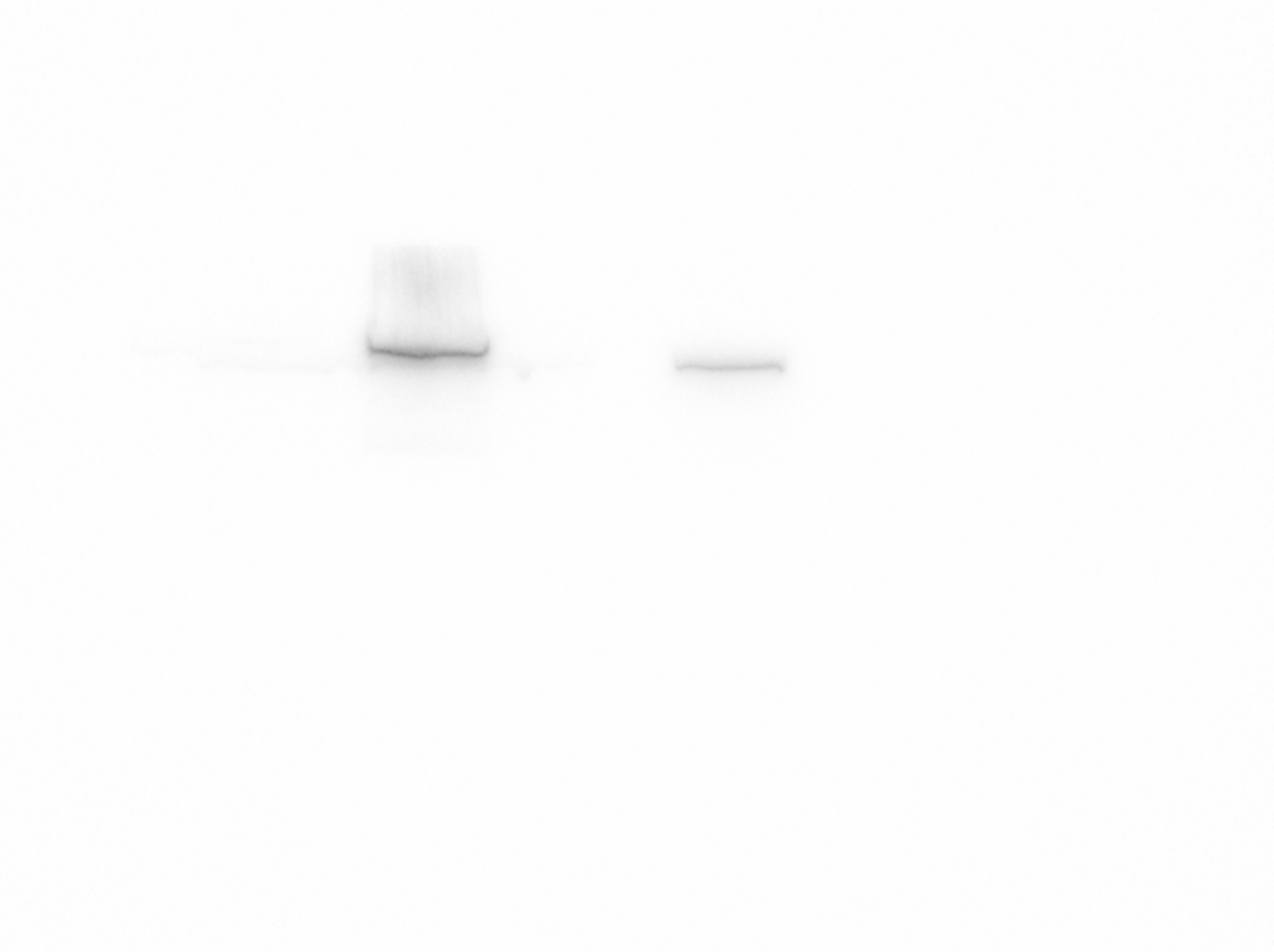

Supplement: Figure 2—figure supplement 1—source data 1. [file elife-73523-fig2-figsupp1-data1.zip › Raw blots/Anti-BRG1.tif]

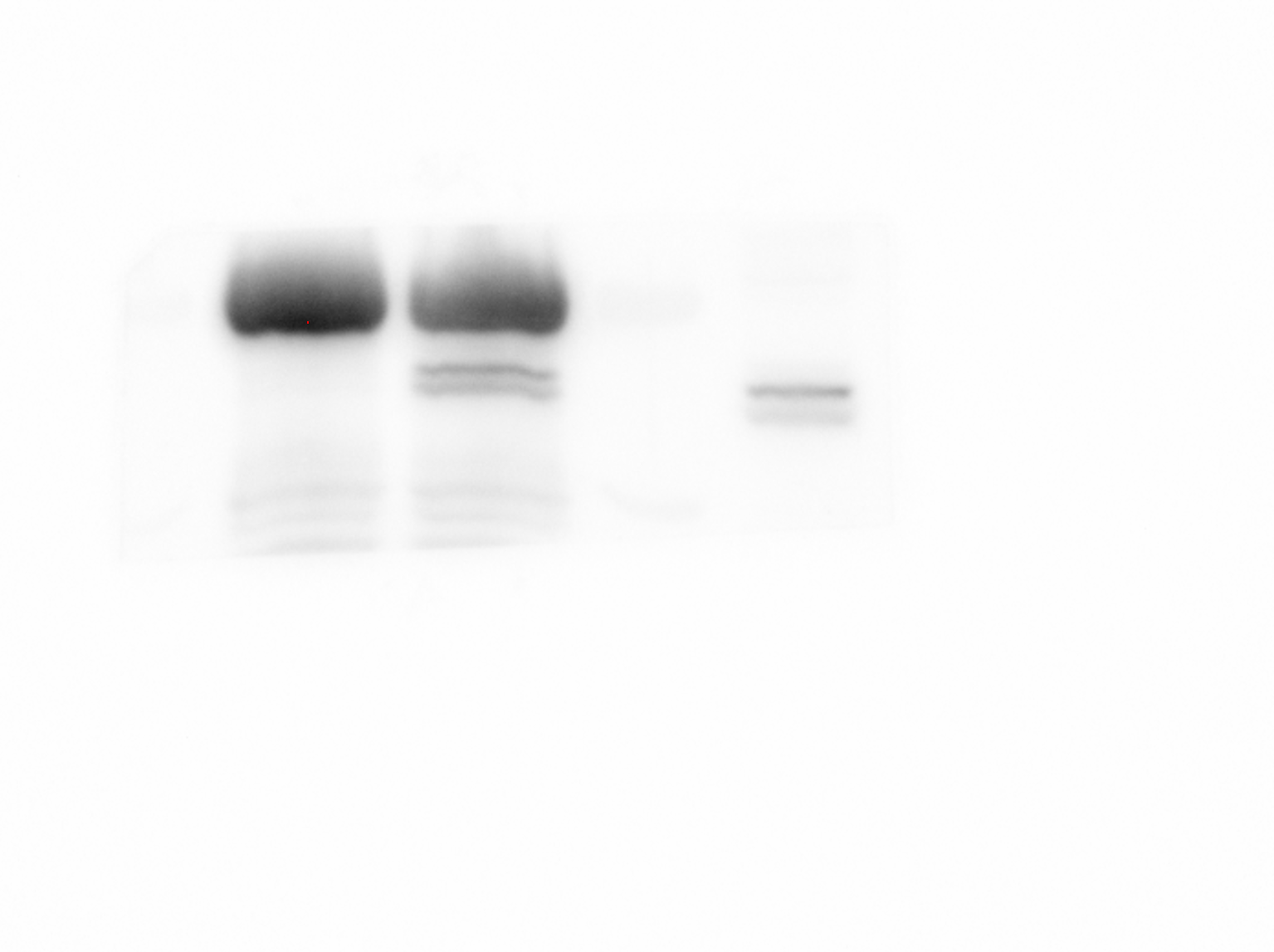

Supplement: Figure 2—figure supplement 1—source data 1. [file elife-73523-fig2-figsupp1-data1.zip › Raw blots/anti-SMARCB1.tif]

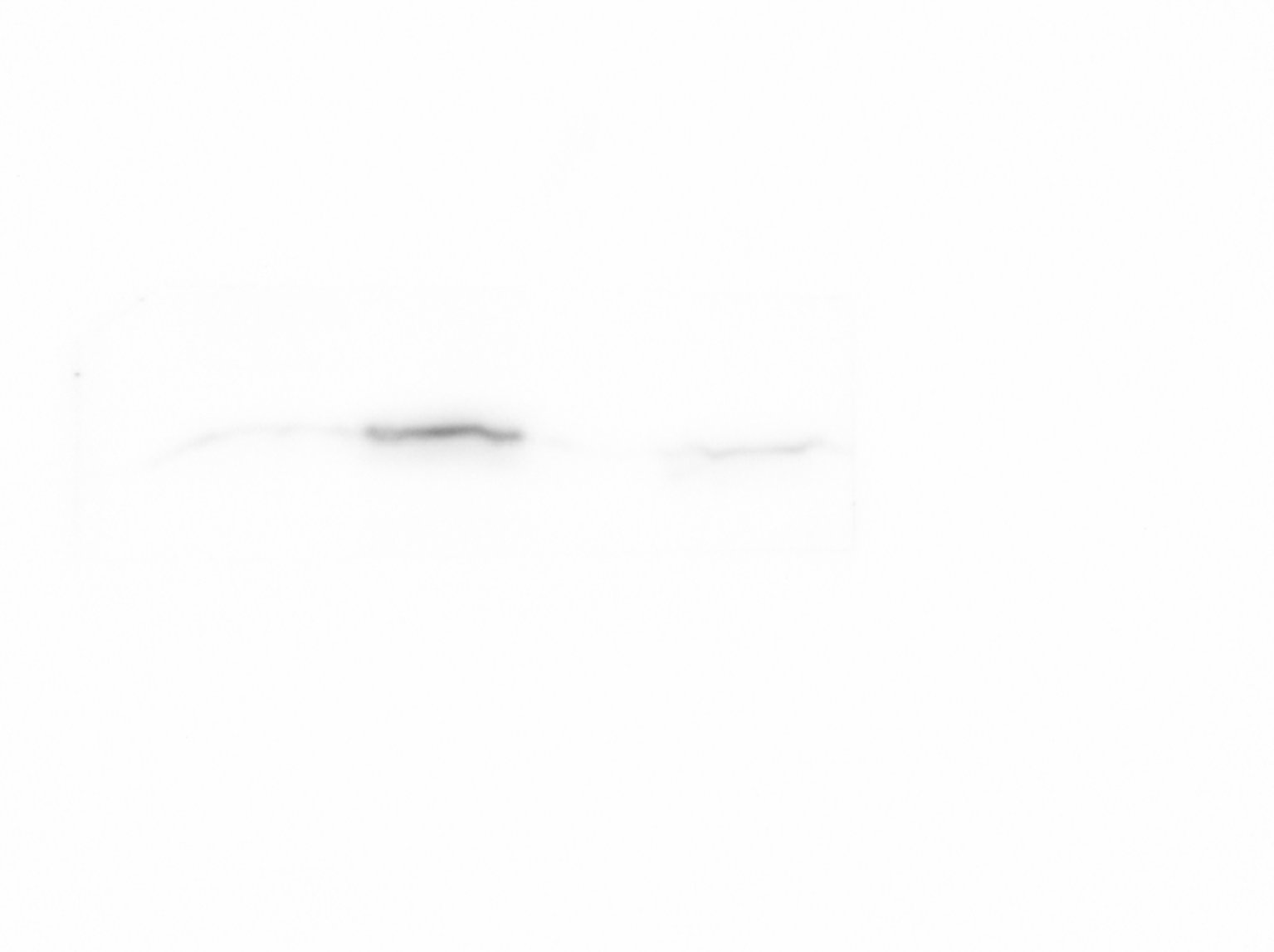

Supplement: Figure 2—figure supplement 1—source data 1. [file elife-73523-fig2-figsupp1-data1.zip › Raw blots/IP_ anti-IPMK.tif]

**A**

IP  
IgG IPMK

Input

IPMK

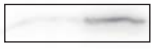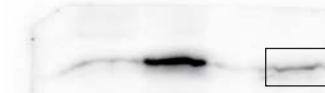

SMARCB1

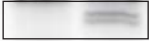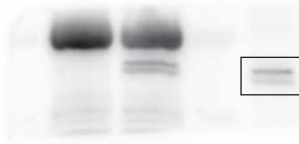

BRG1

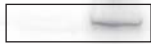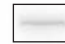

Supplement: Figure 2—figure supplement 1—source data 1. [file elife-73523-fig2-figsupp1-data1.zip › Labelled blots.pdf]

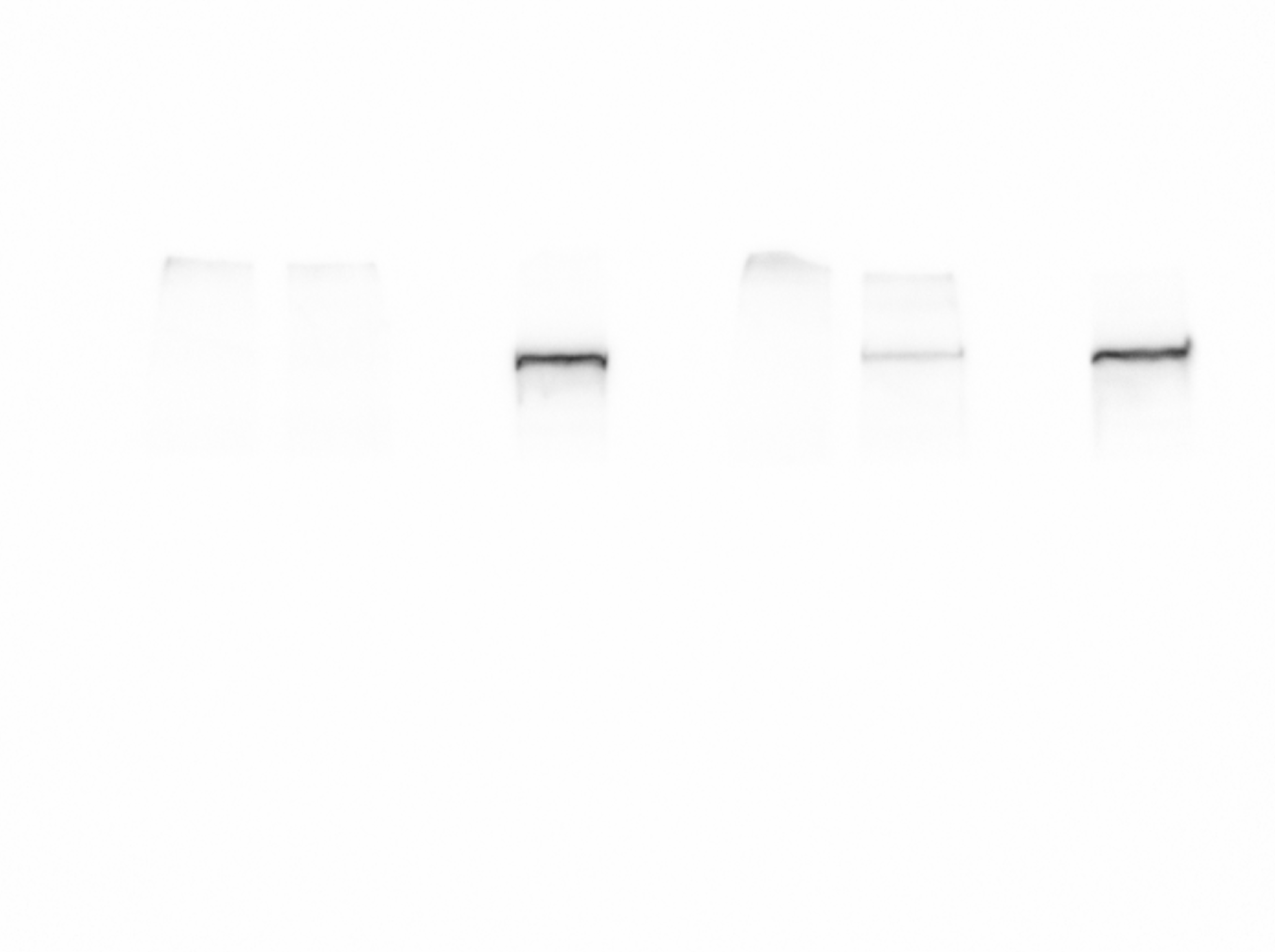

Supplement: Figure 2—figure supplement 1—source data 2. [file elife-73523-fig2-figsupp1-data2.zip › Raw blots/Input_ anti-BRG1.tif]

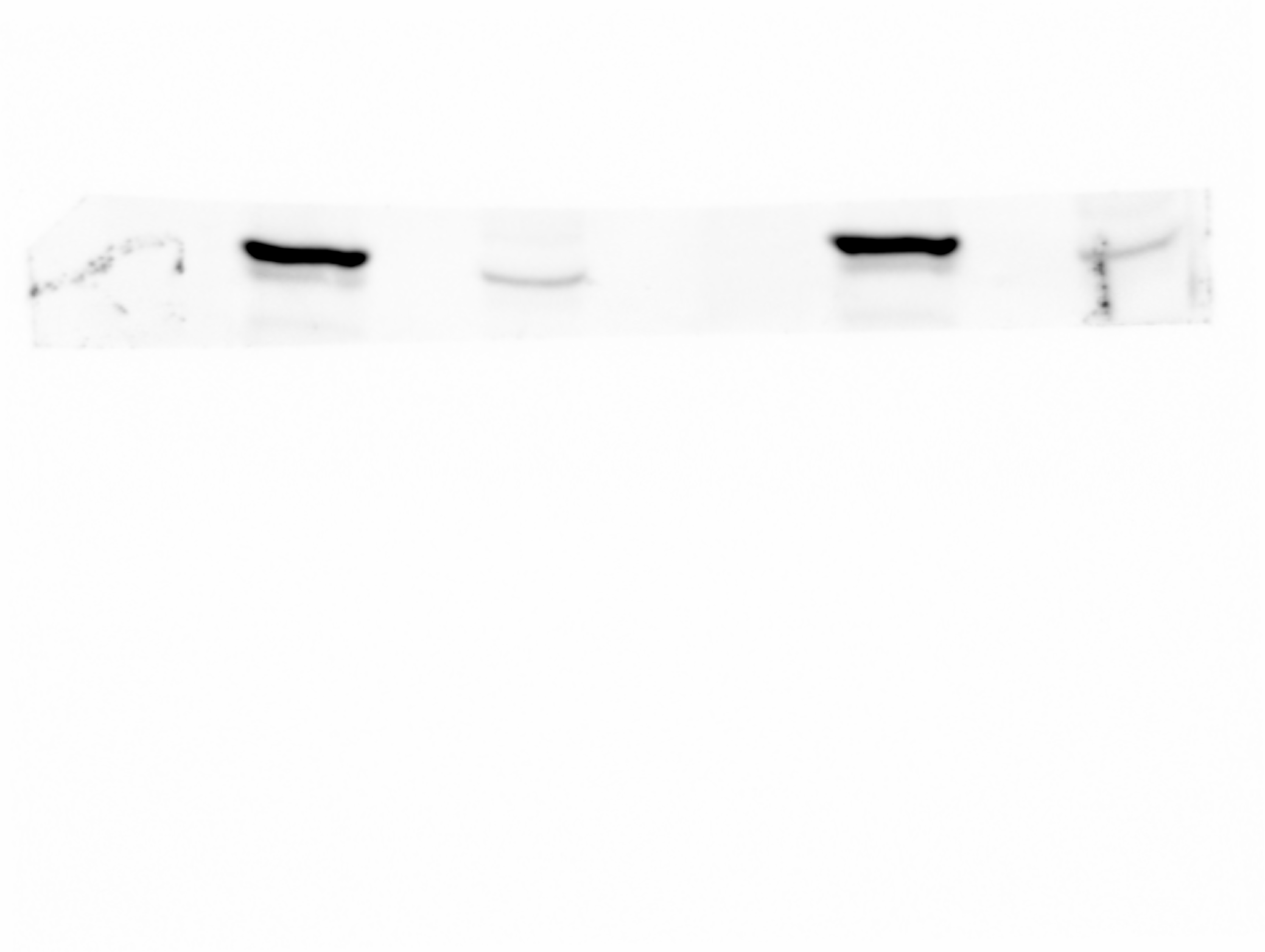

Supplement: Figure 2—figure supplement 1—source data 2. [file elife-73523-fig2-figsupp1-data2.zip › Raw blots/Input_ anti-IPMK.tif]

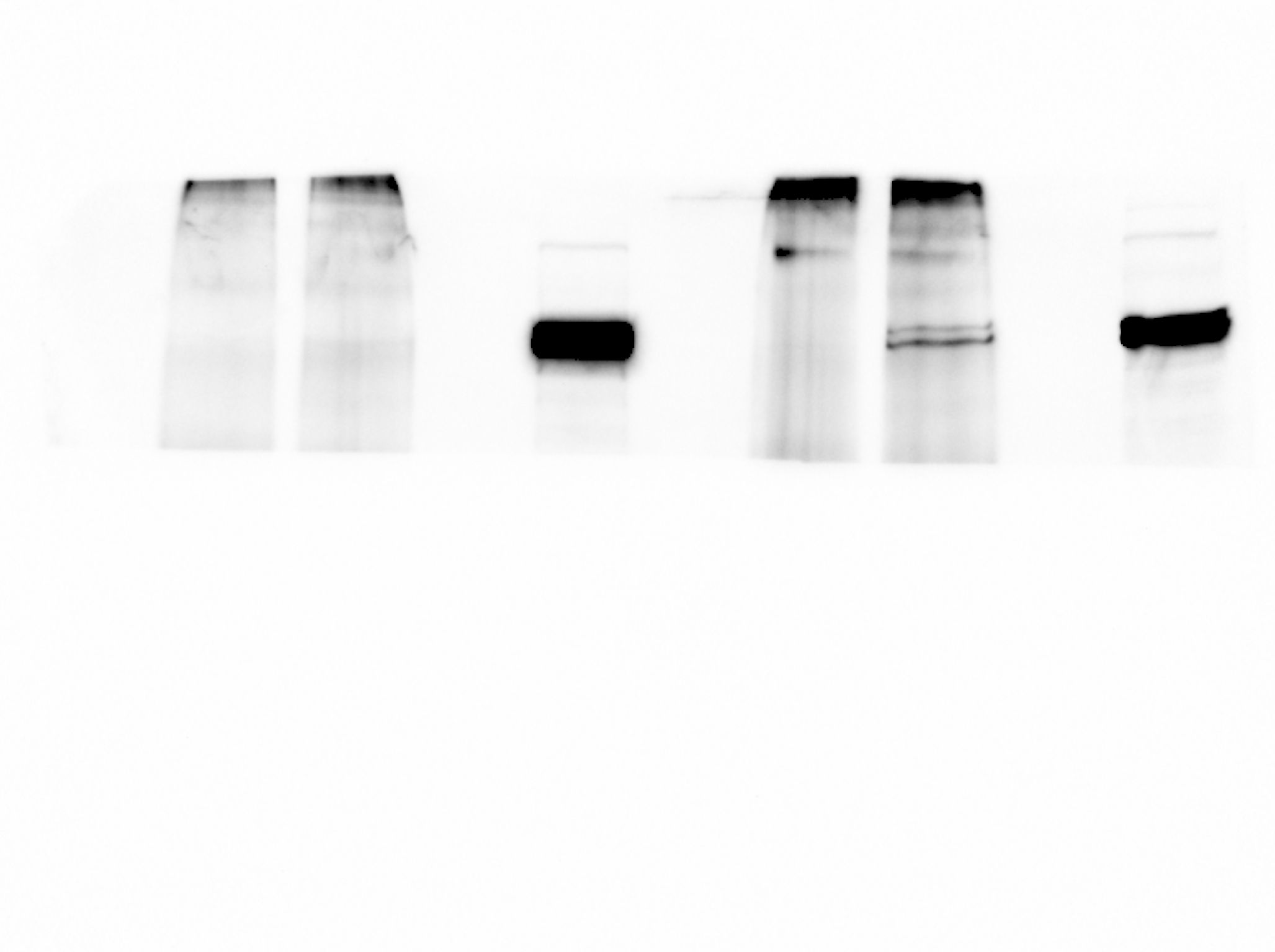

Supplement: Figure 2—figure supplement 1—source data 2. [file elife-73523-fig2-figsupp1-data2.zip › Raw blots/IP_ anti-BAF170.tif]

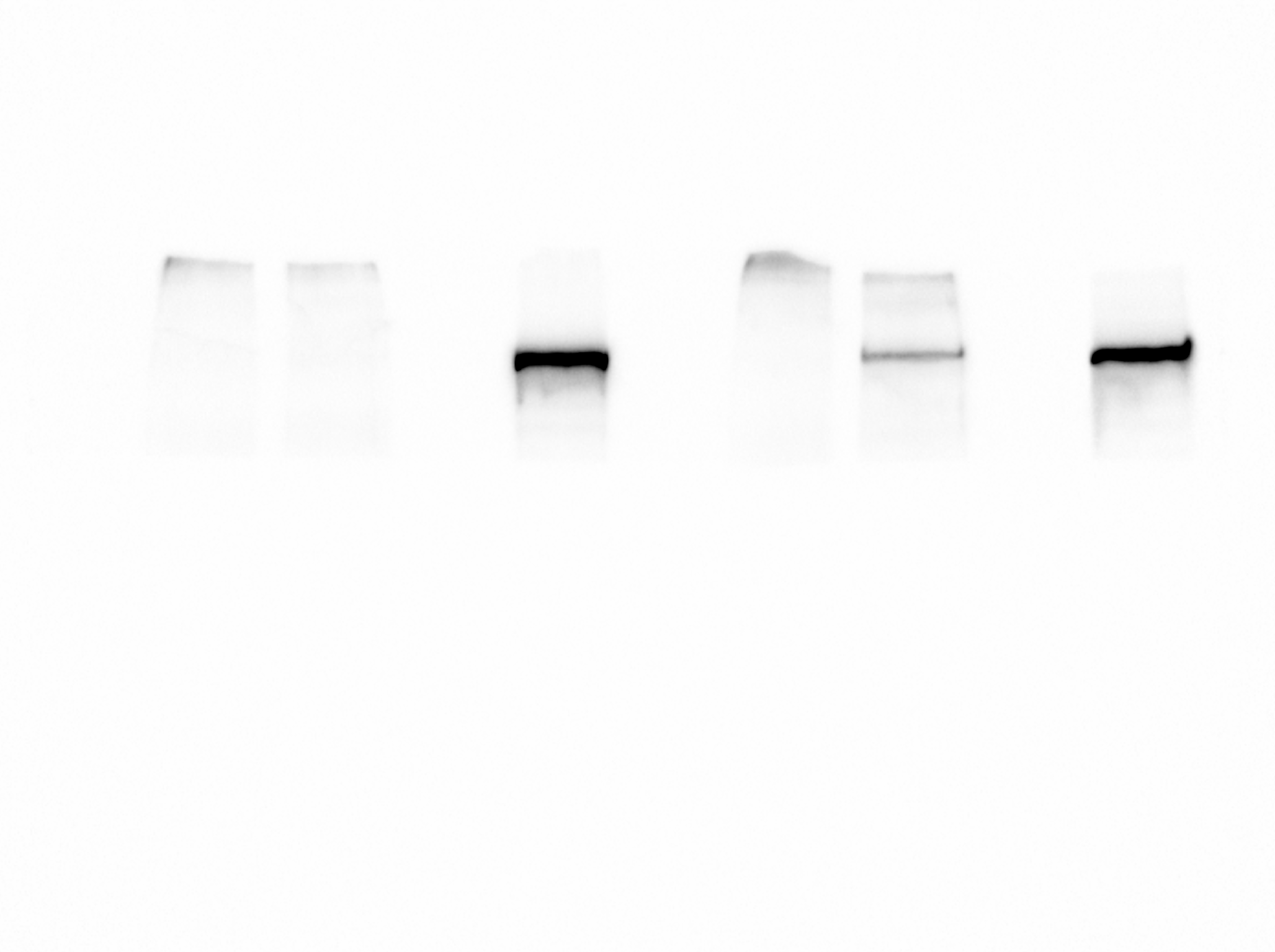

Supplement: Figure 2—figure supplement 1—source data 2. [file elife-73523-fig2-figsupp1-data2.zip › Raw blots/IP_ anti-BRG1.tif]

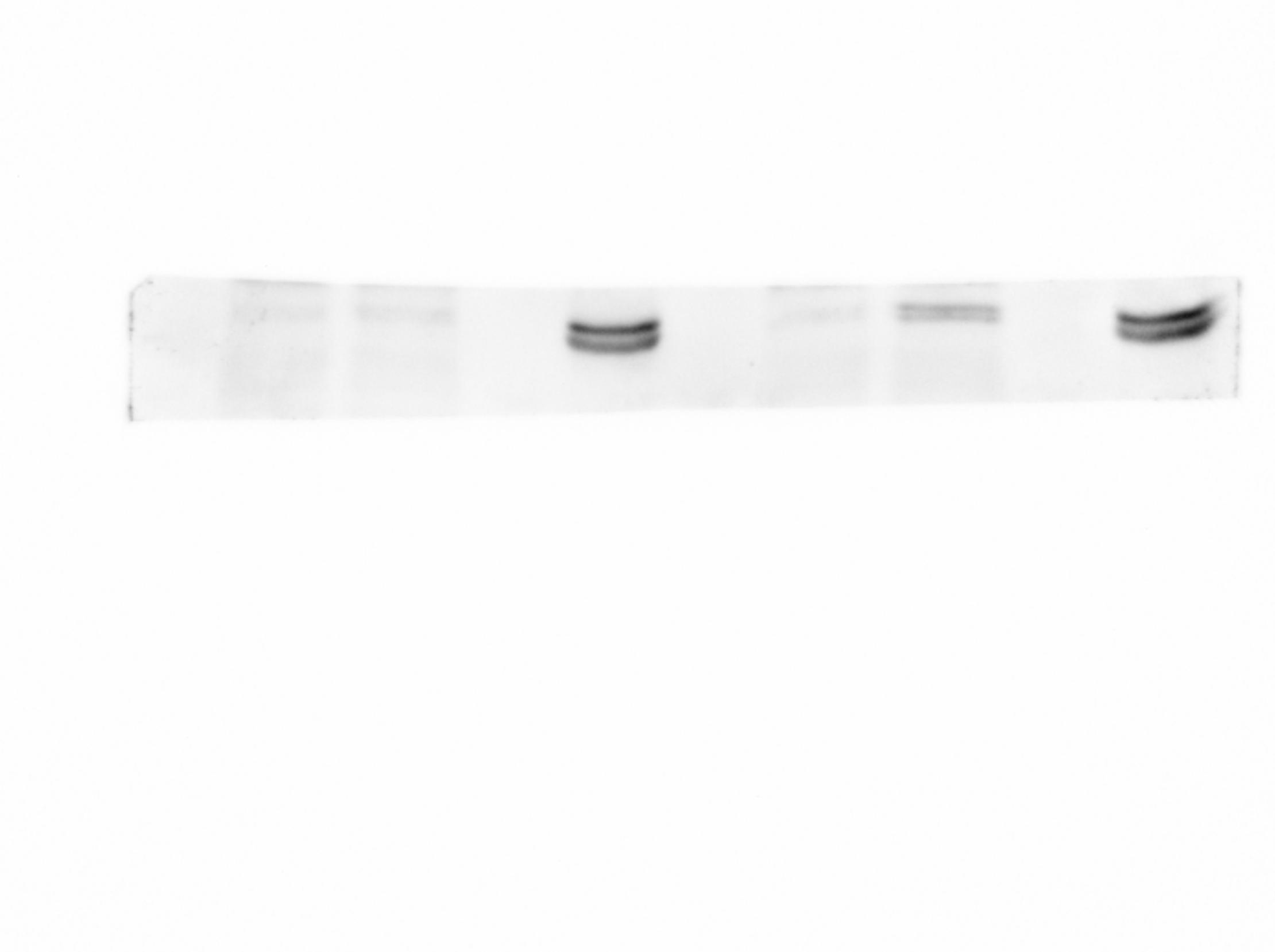

Supplement: Figure 2—figure supplement 1—source data 2. [file elife-73523-fig2-figsupp1-data2.zip › Raw blots/IP_ anti-SMARCB1.tif]

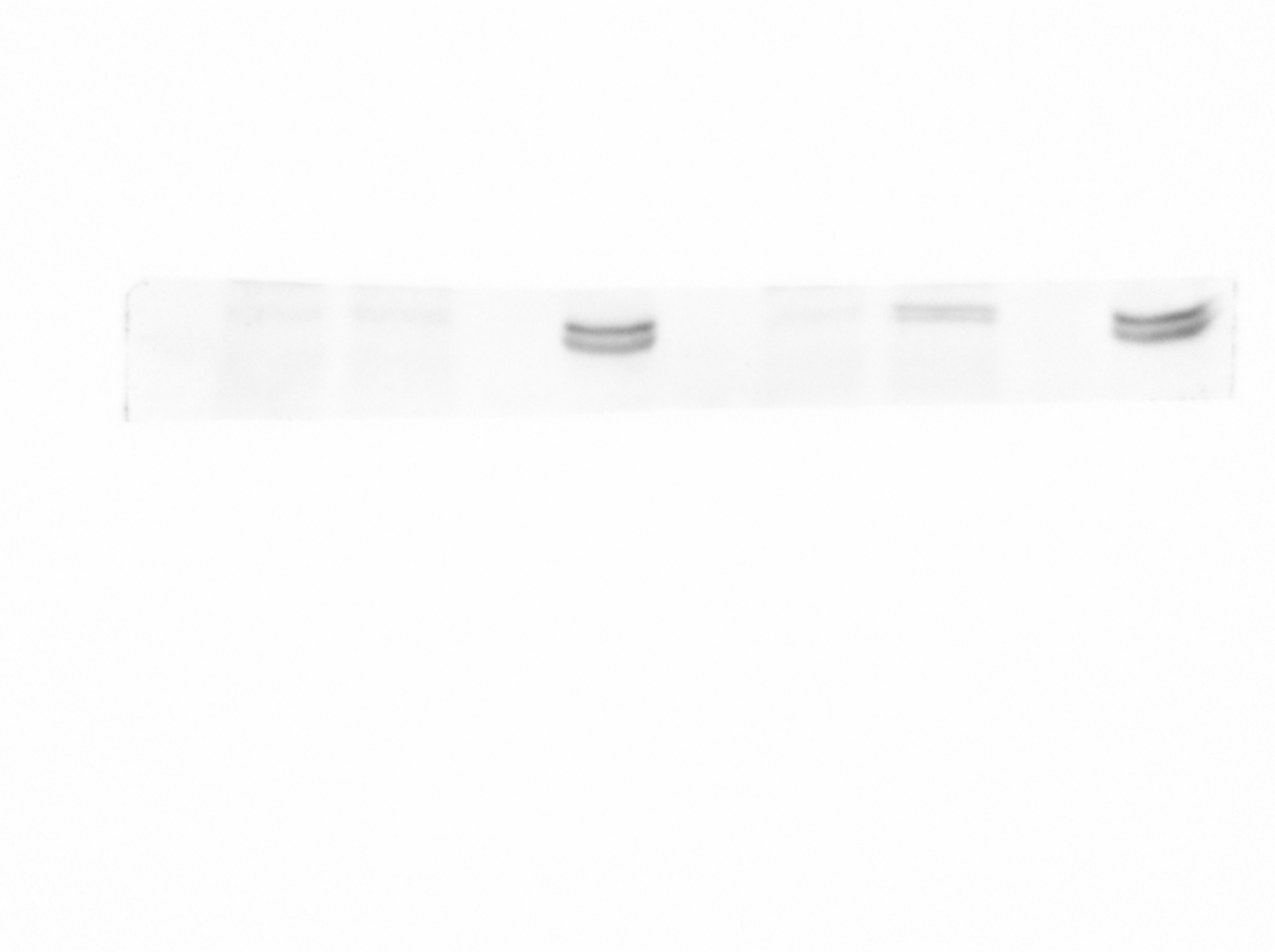

Supplement: Figure 2—figure supplement 1—source data 2. [file elife-73523-fig2-figsupp1-data2.zip › Raw blots/Input_ anti-SMARCB1.tif]

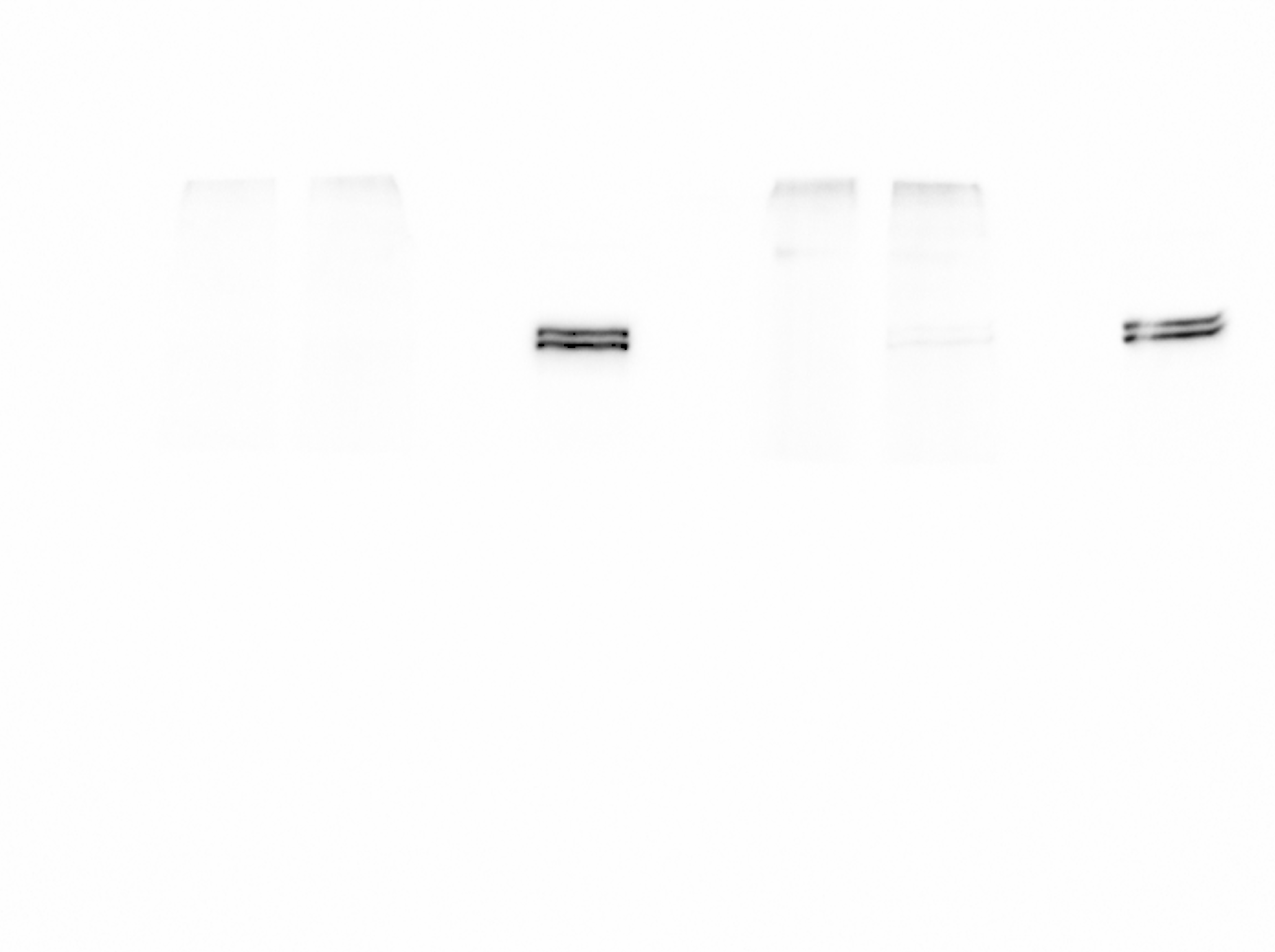

Supplement: Figure 2—figure supplement 1—source data 2. [file elife-73523-fig2-figsupp1-data2.zip › Raw blots/Input_ anti-BAF170.tif]

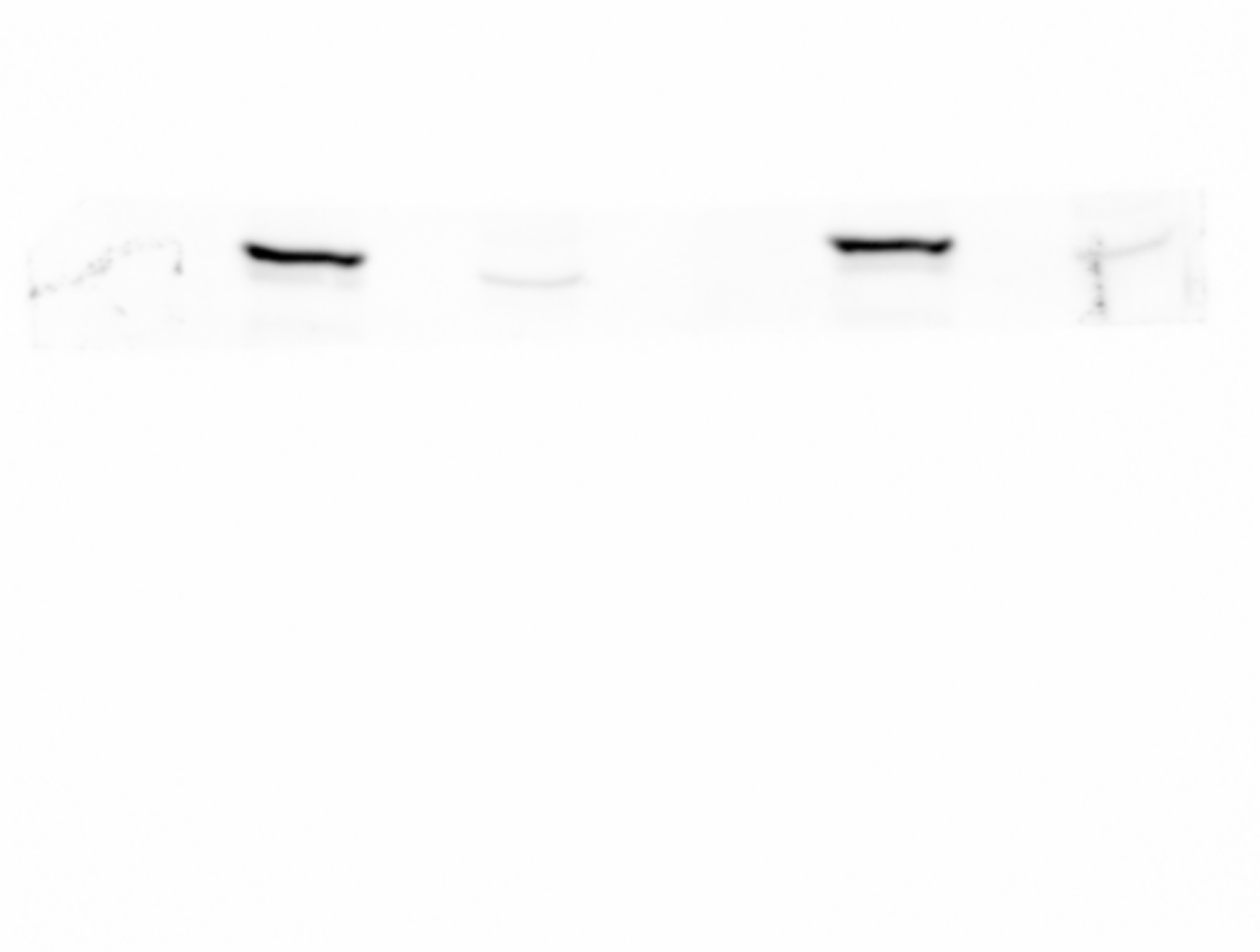

Supplement: Figure 2—figure supplement 1—source data 2. [file elife-73523-fig2-figsupp1-data2.zip › Raw blots/IP_ anti-IPMK.tif]

**B**

|  | IP  |      |
|--|-----|------|
|  | IgG | IPMK |

Input

IPMK

SMARCB1

BAF170

BRG1

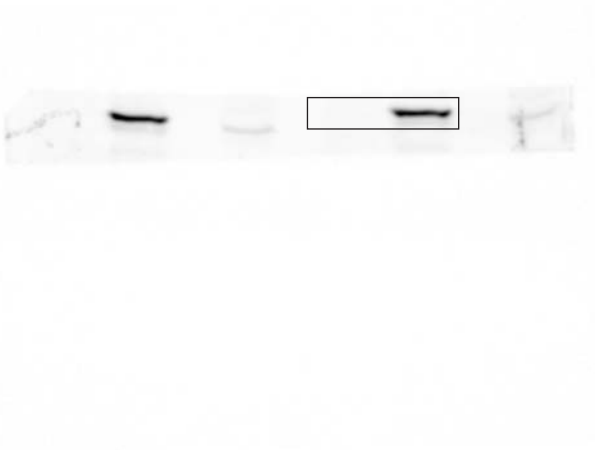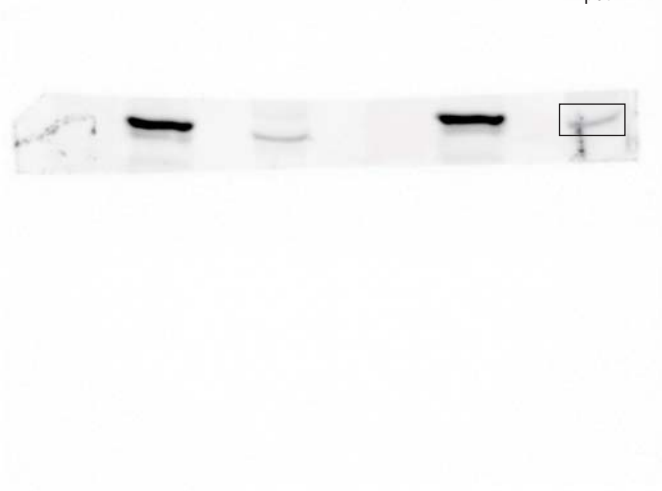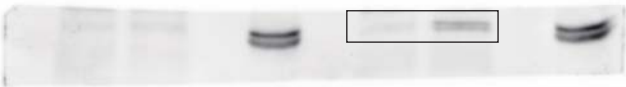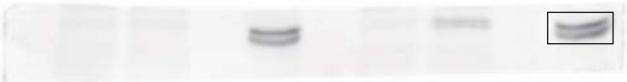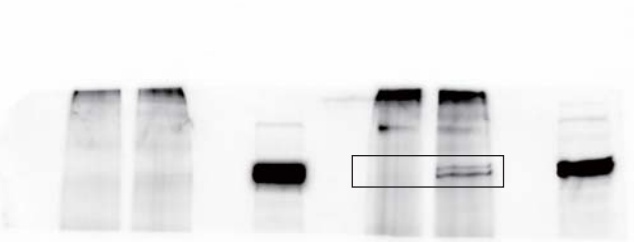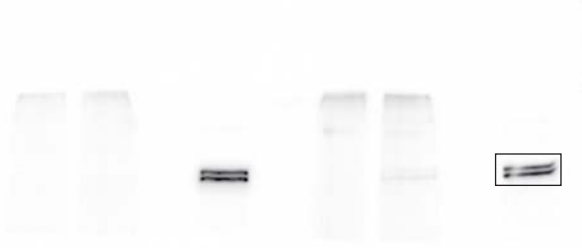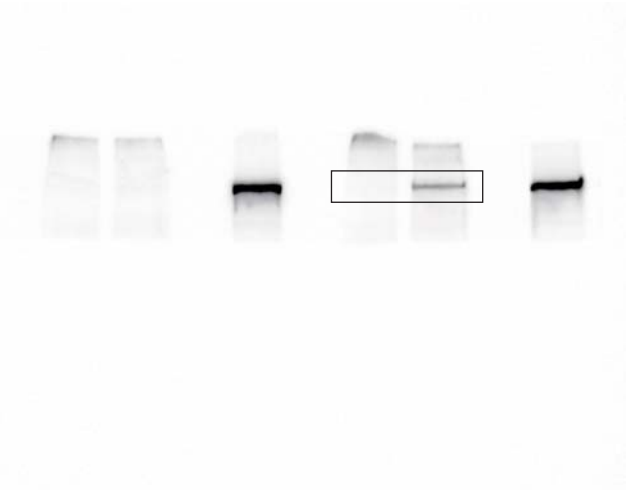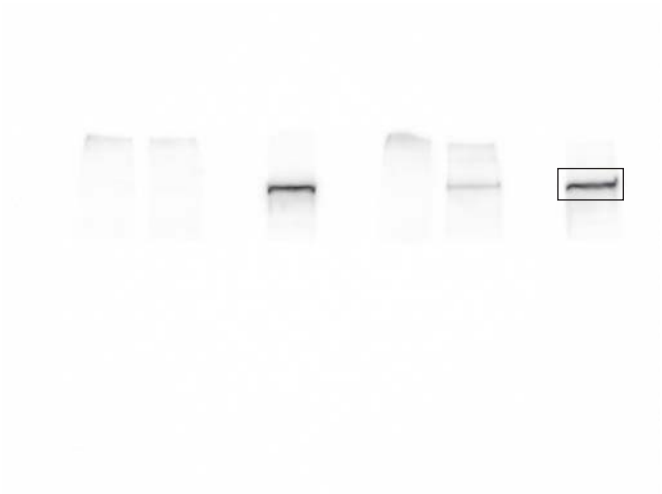

Supplement: Figure 2—figure supplement 1—source data 2. [file elife-73523-fig2-figsupp1-data2.zip › Labelled blots.pdf]

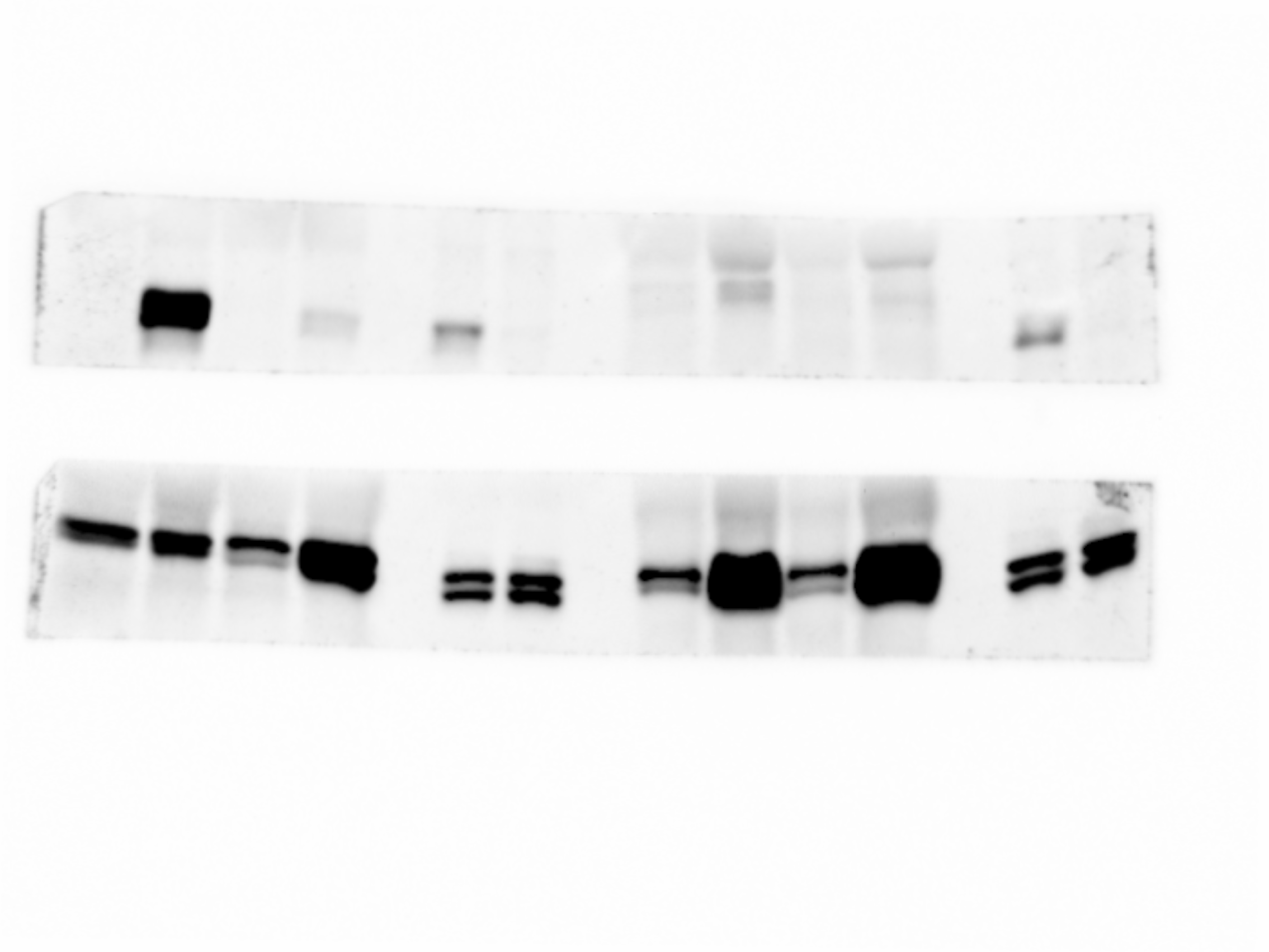

Supplement: Figure 2—figure supplement 1—source data 3. [file elife-73523-fig2-figsupp1-data3.zip › Raw blots/anti-IPMK.tif]

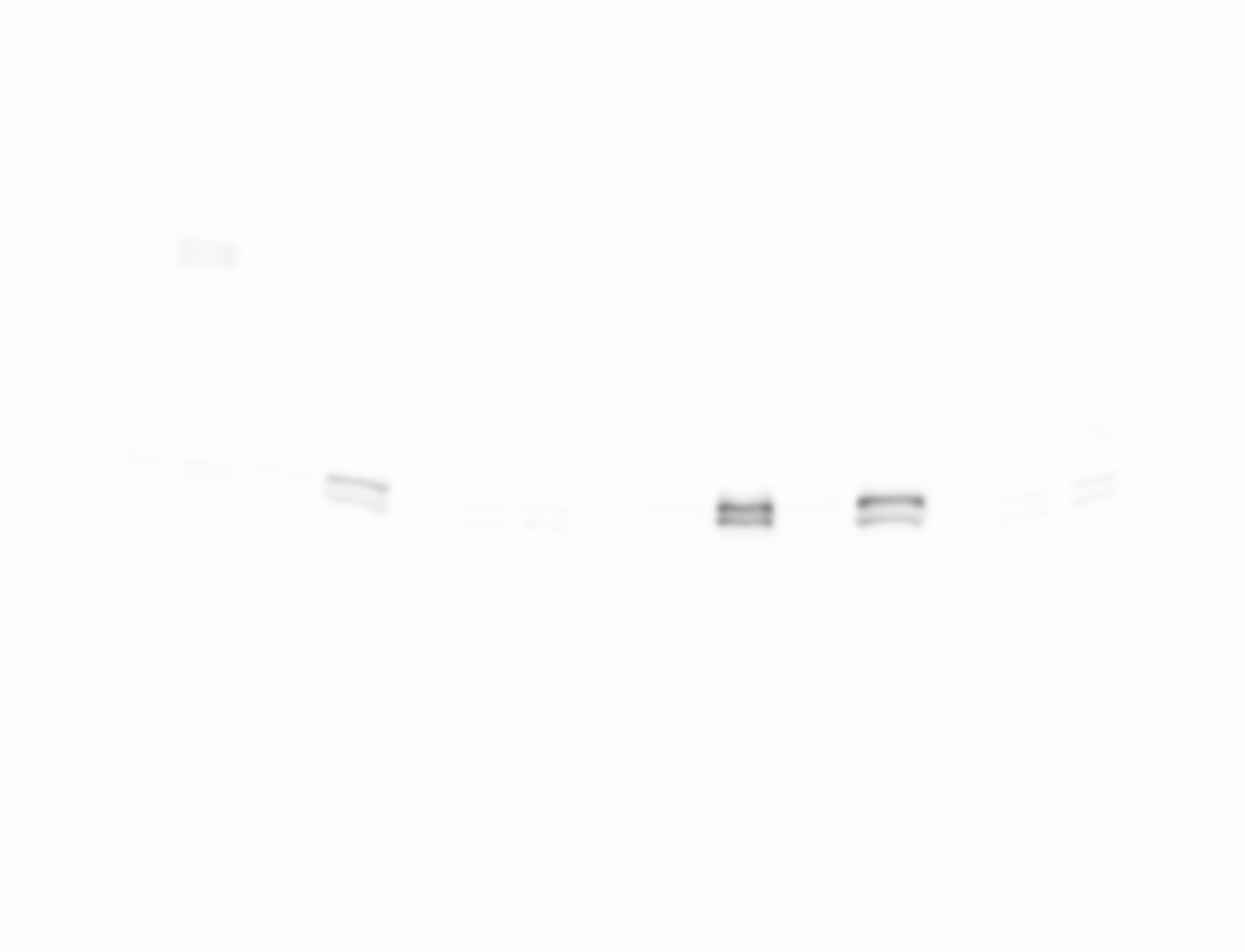

Supplement: Figure 2—figure supplement 1—source data 3. [file elife-73523-fig2-figsupp1-data3.zip › Raw blots/IP_ anti-SMARCB1.tif]

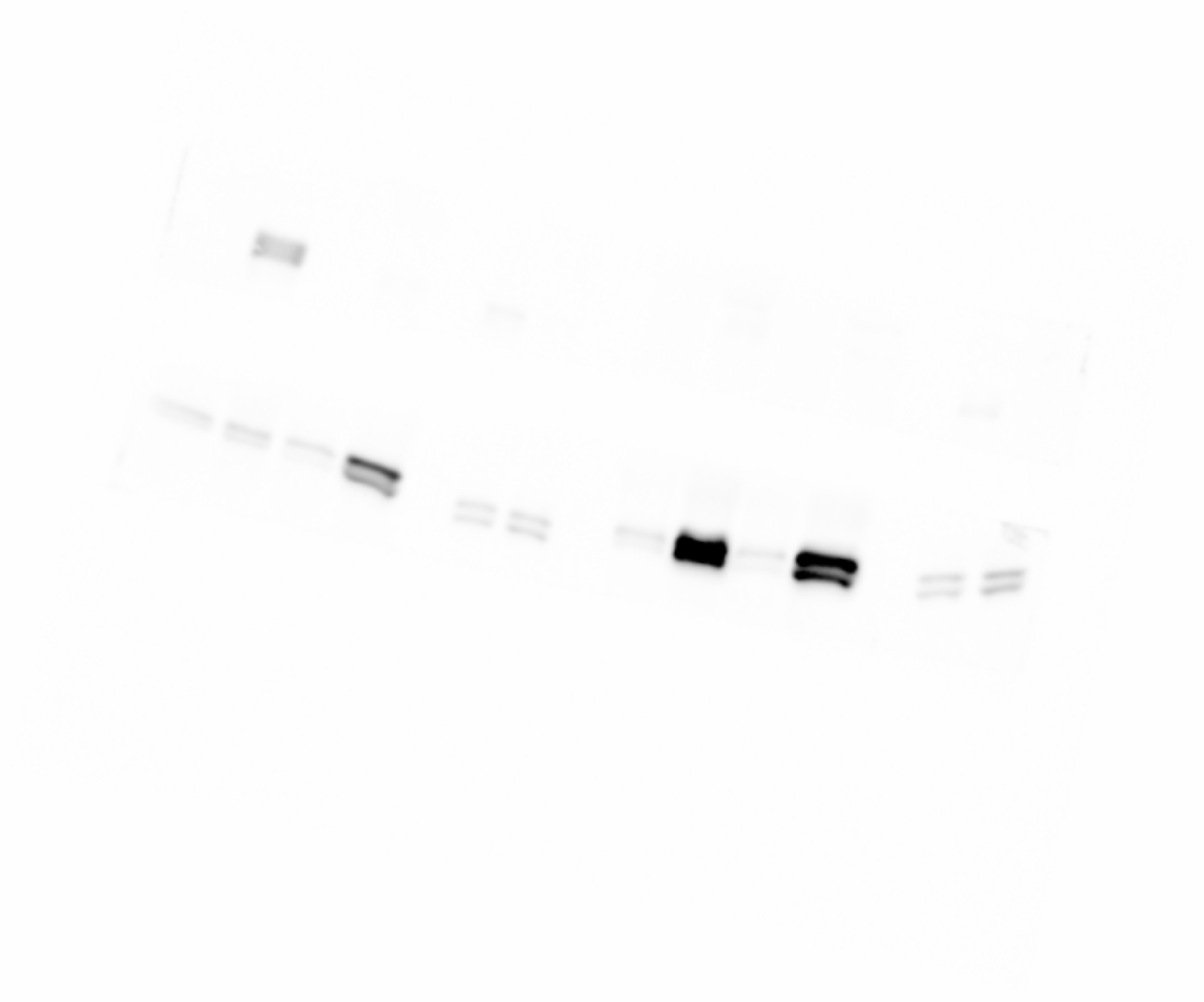

Supplement: Figure 2—figure supplement 1—source data 3. [file elife-73523-fig2-figsupp1-data3.zip › Raw blots/Input_ anti-SMARCB1.tif]

**C**

IP : IgG SMARCB1  
WT WT KO KO

Input  
WT KO

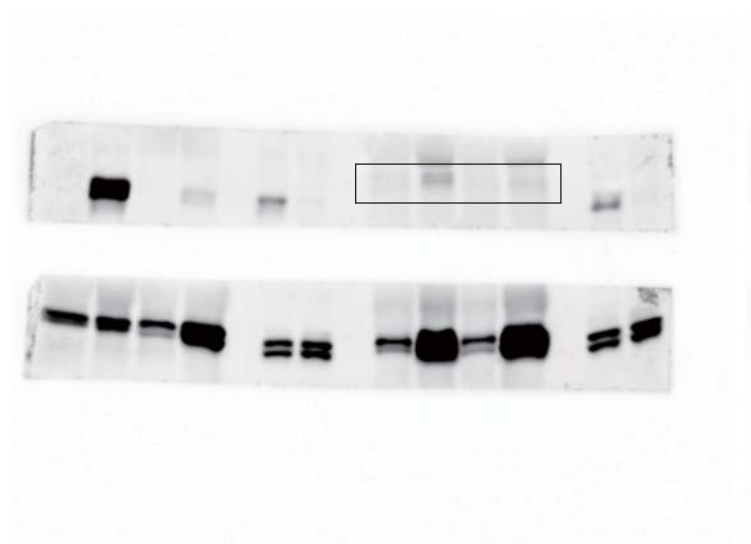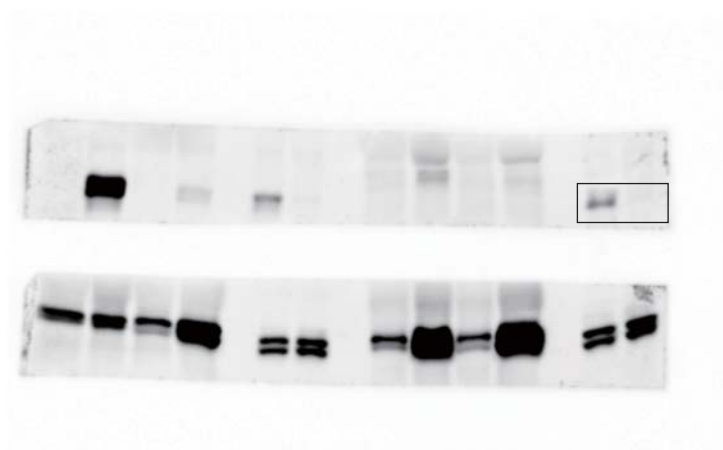

IPMK

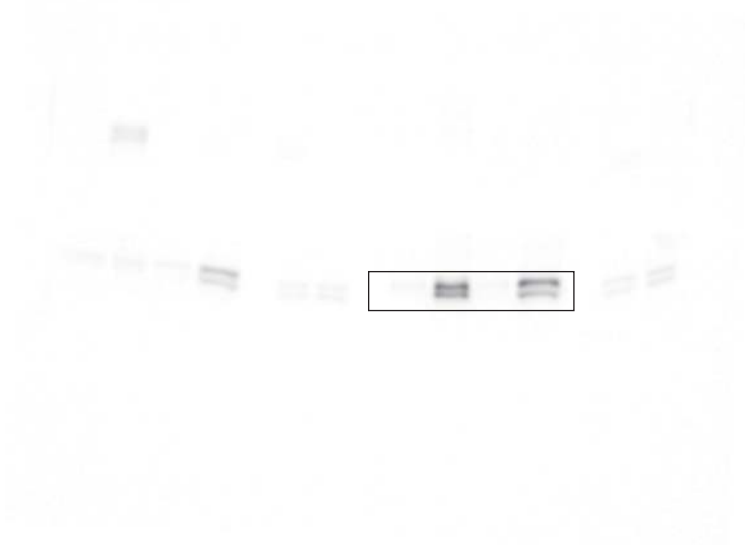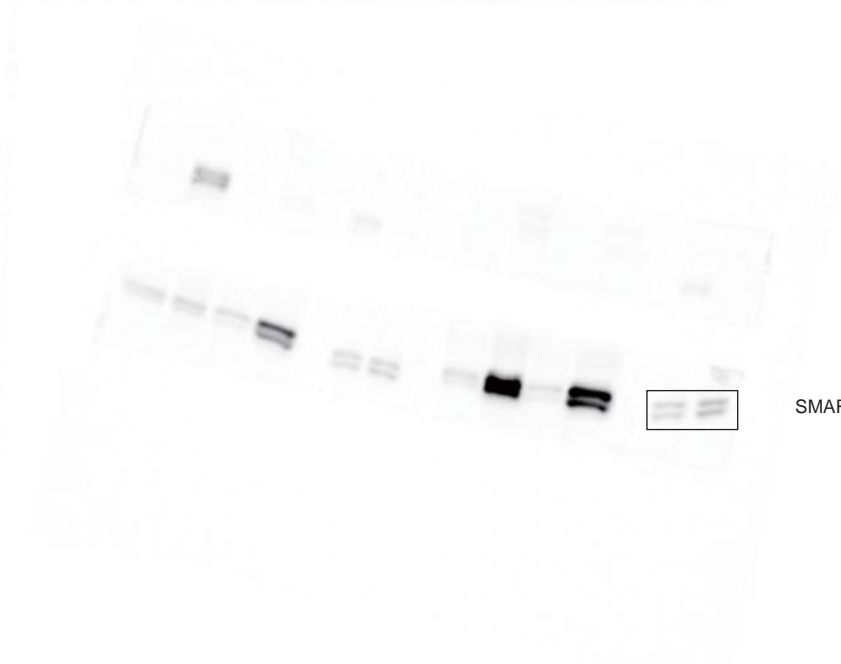

SMARCB1

Supplement: Figure 2—figure supplement 1—source data 3. [file elife-73523-fig2-figsupp1-data3.zip › Labelled blots.pdf]

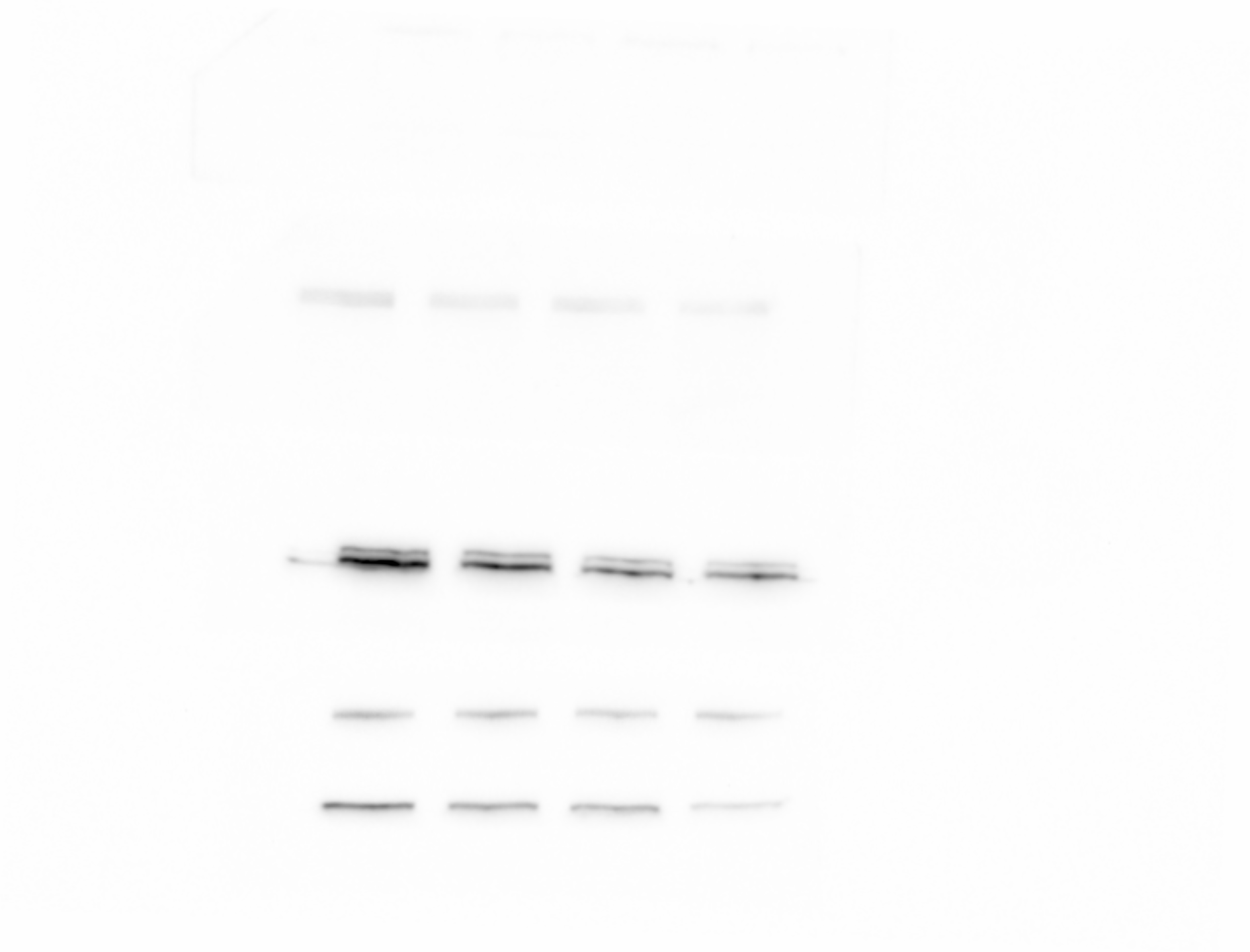

Supplement: Figure 2—figure supplement 1—source data 4. [file elife-73523-fig2-figsupp1-data4.zip › Raw blots/NIH3T3/anti-BAF155.tif]

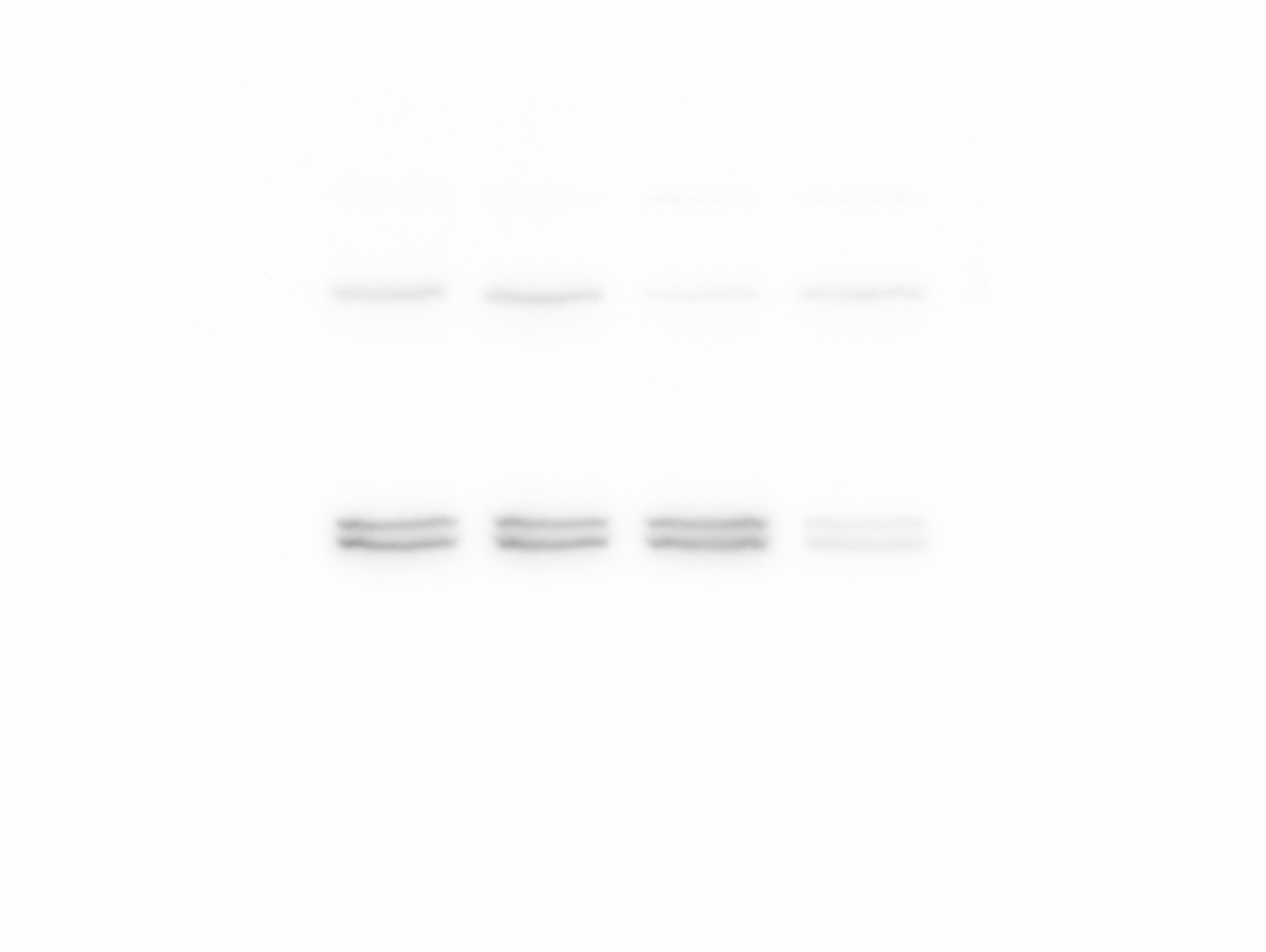

Supplement: Figure 2—figure supplement 1—source data 4. [file elife-73523-fig2-figsupp1-data4.zip › Raw blots/NIH3T3/anti-IPMK.tif]

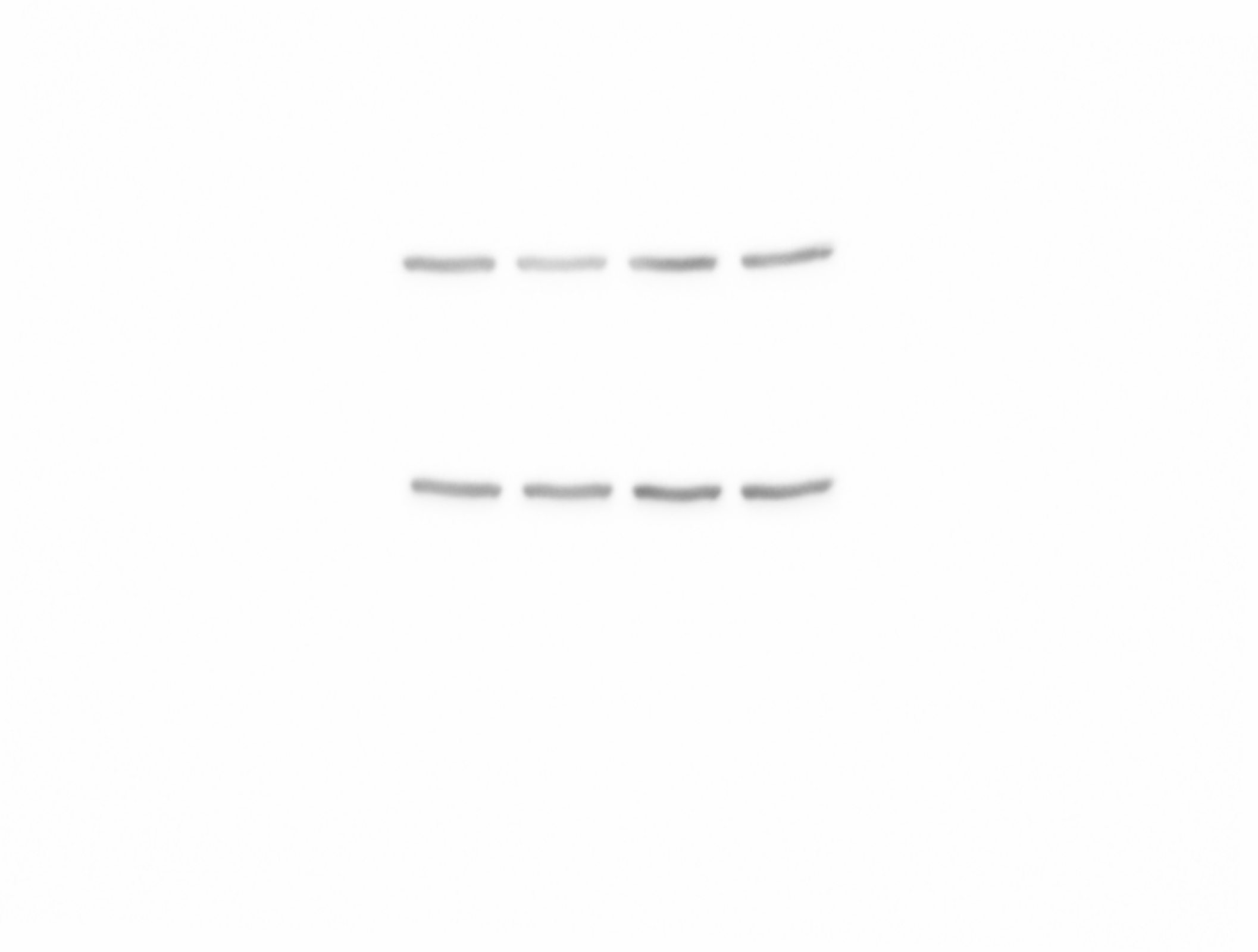

Supplement: Figure 2—figure supplement 1—source data 4. [file elife-73523-fig2-figsupp1-data4.zip › Raw blots/NIH3T3/anti-alpha TUBULIN.tif]

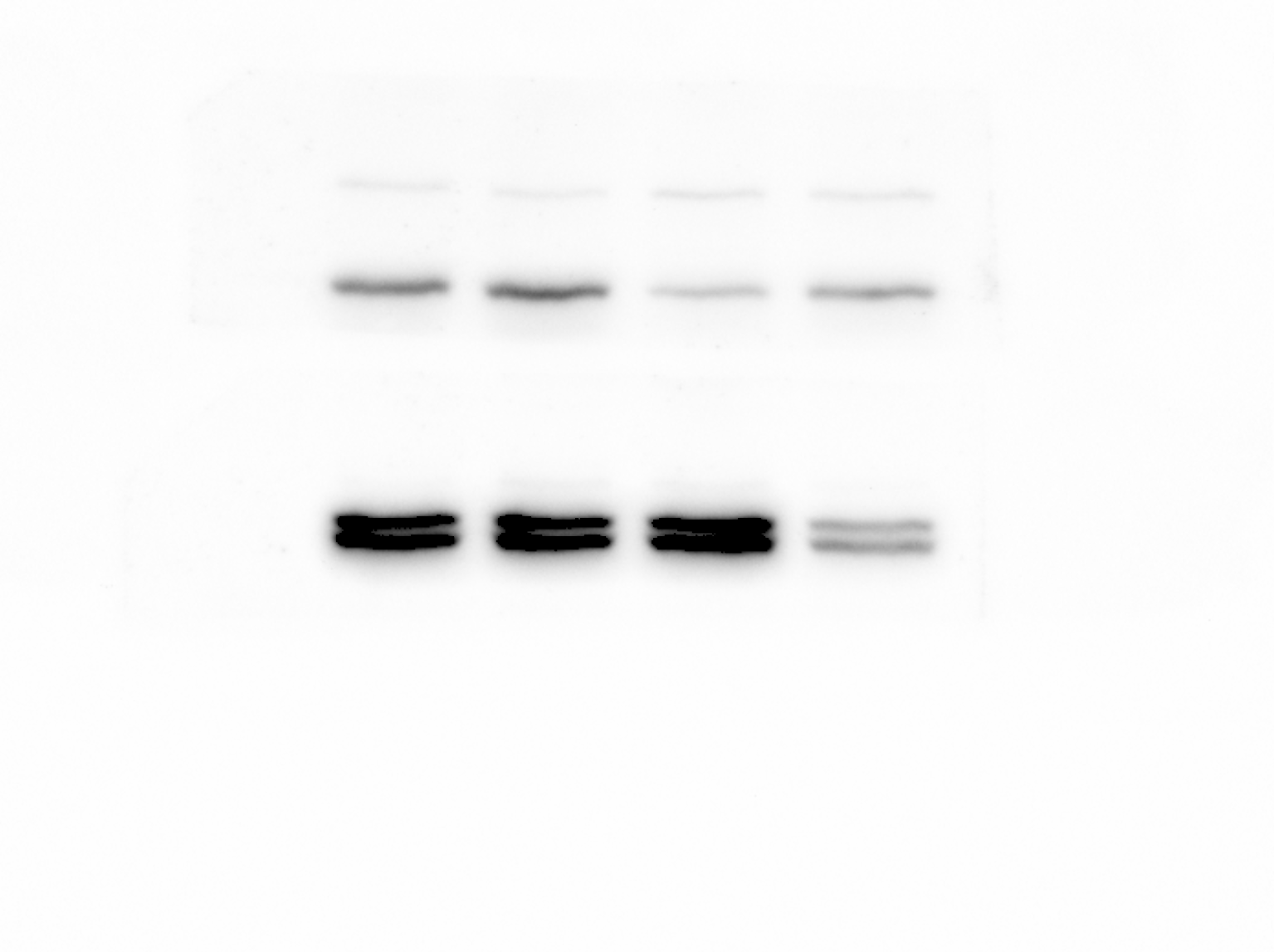

Supplement: Figure 2—figure supplement 1—source data 4. [file elife-73523-fig2-figsupp1-data4.zip › Raw blots/NIH3T3/anti-SMARCB1.tif]

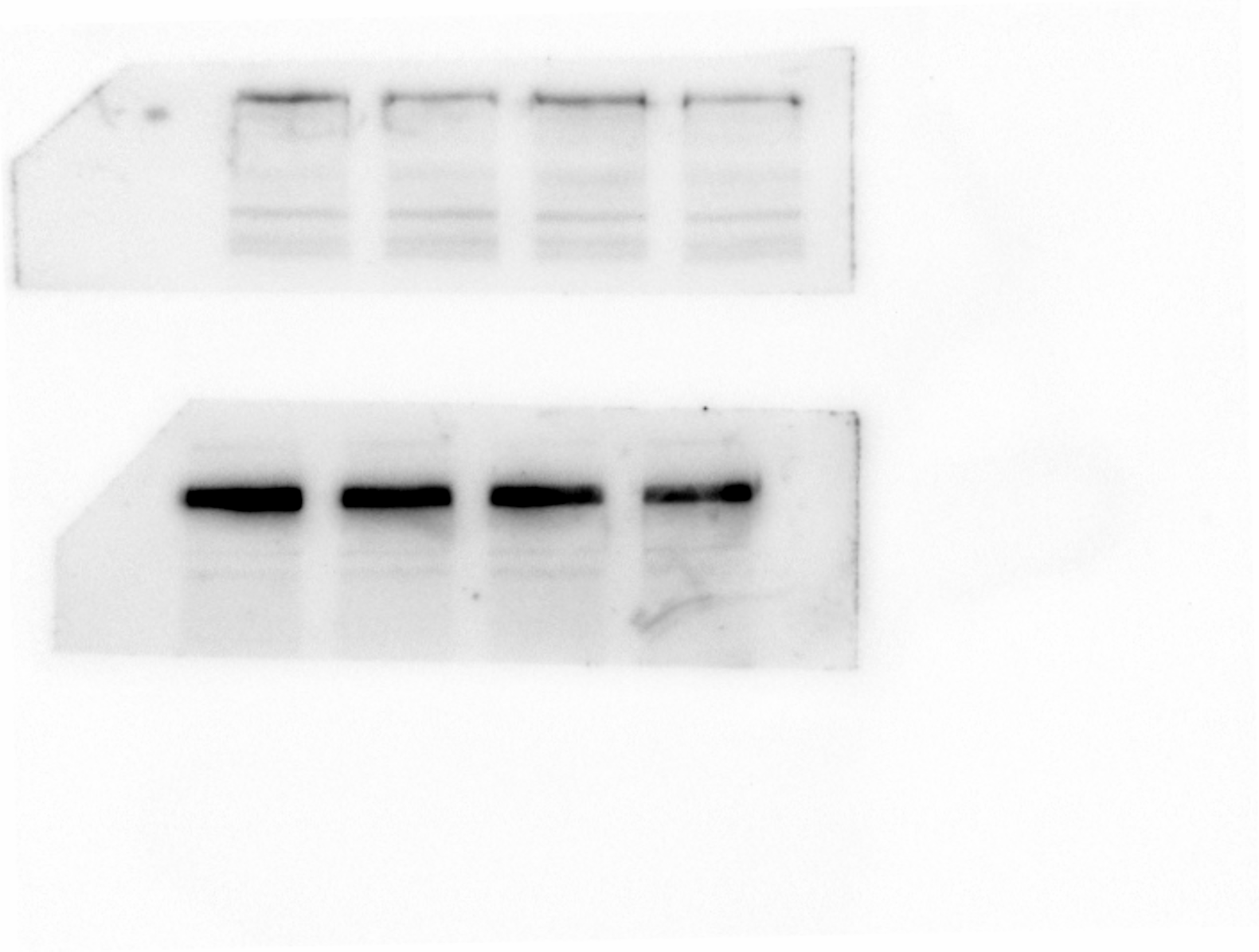

Supplement: Figure 2—figure supplement 1—source data 4. [file elife-73523-fig2-figsupp1-data4.zip › Raw blots/NIH3T3/anti-BAF250A.tif]

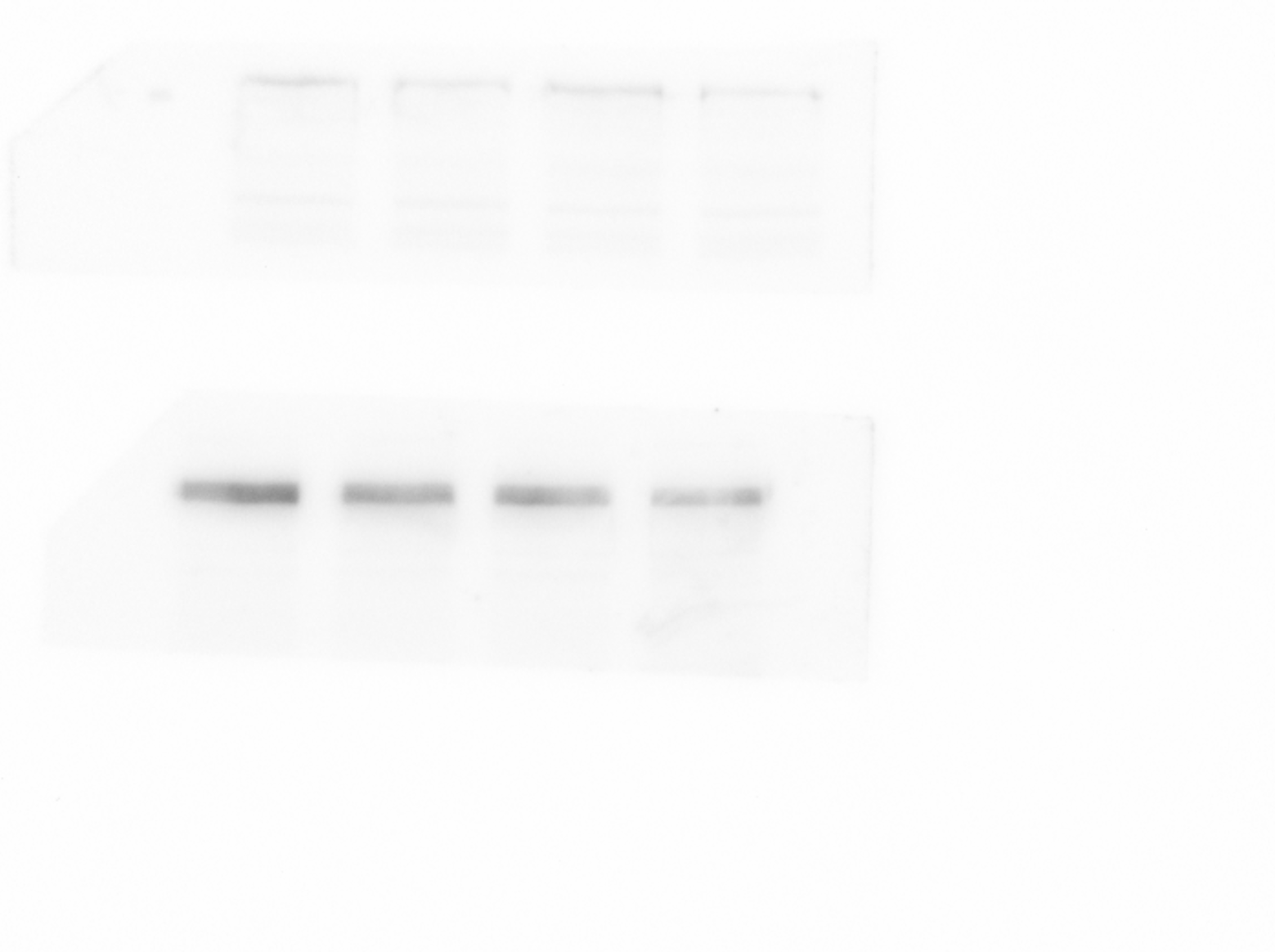

Supplement: Figure 2—figure supplement 1—source data 4. [file elife-73523-fig2-figsupp1-data4.zip › Raw blots/NIH3T3/anti-PBRM1.tif]

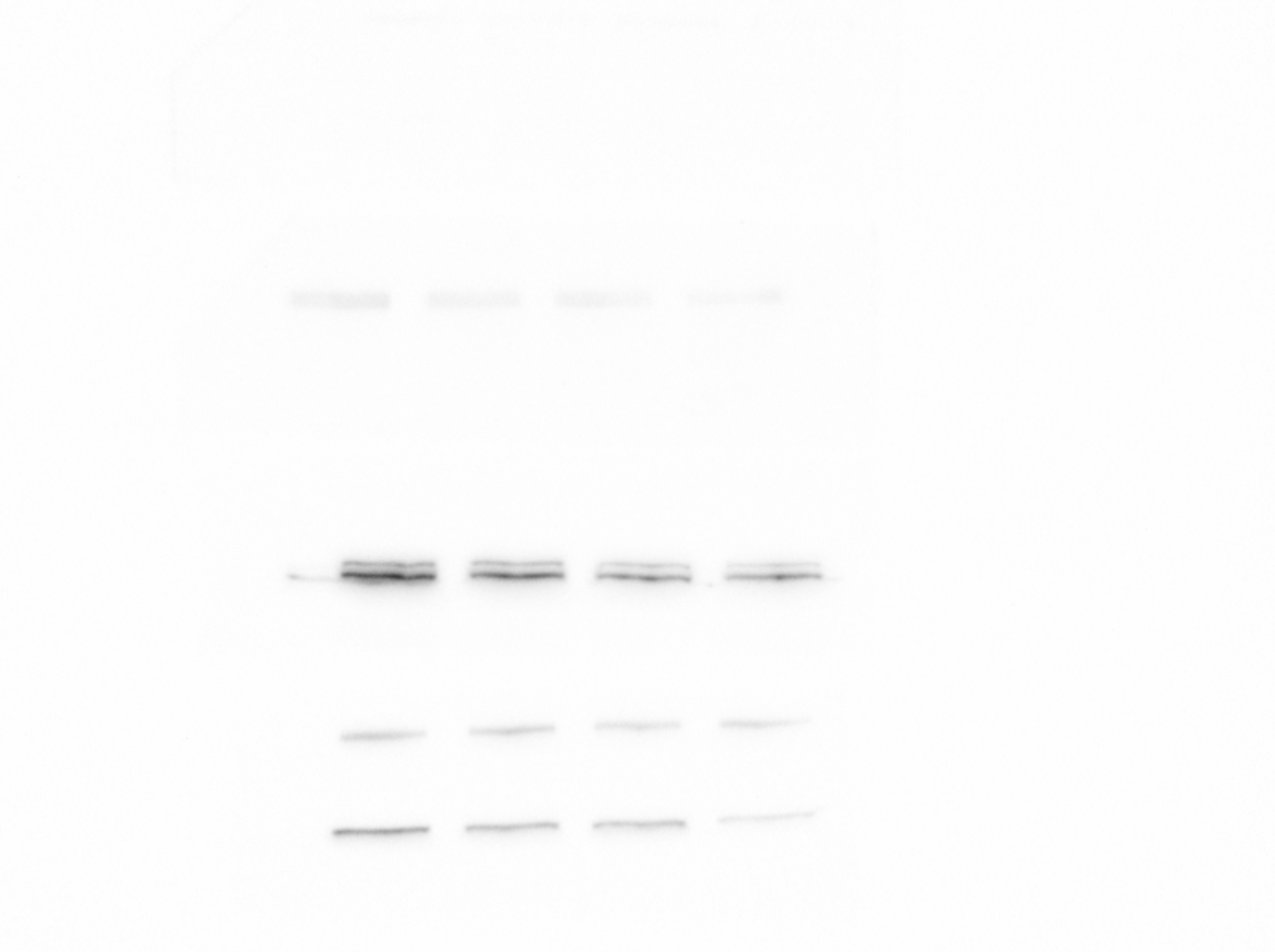

Supplement: Figure 2—figure supplement 1—source data 4. [file elife-73523-fig2-figsupp1-data4.zip › Raw blots/NIH3T3/anti-BAF170.tif]

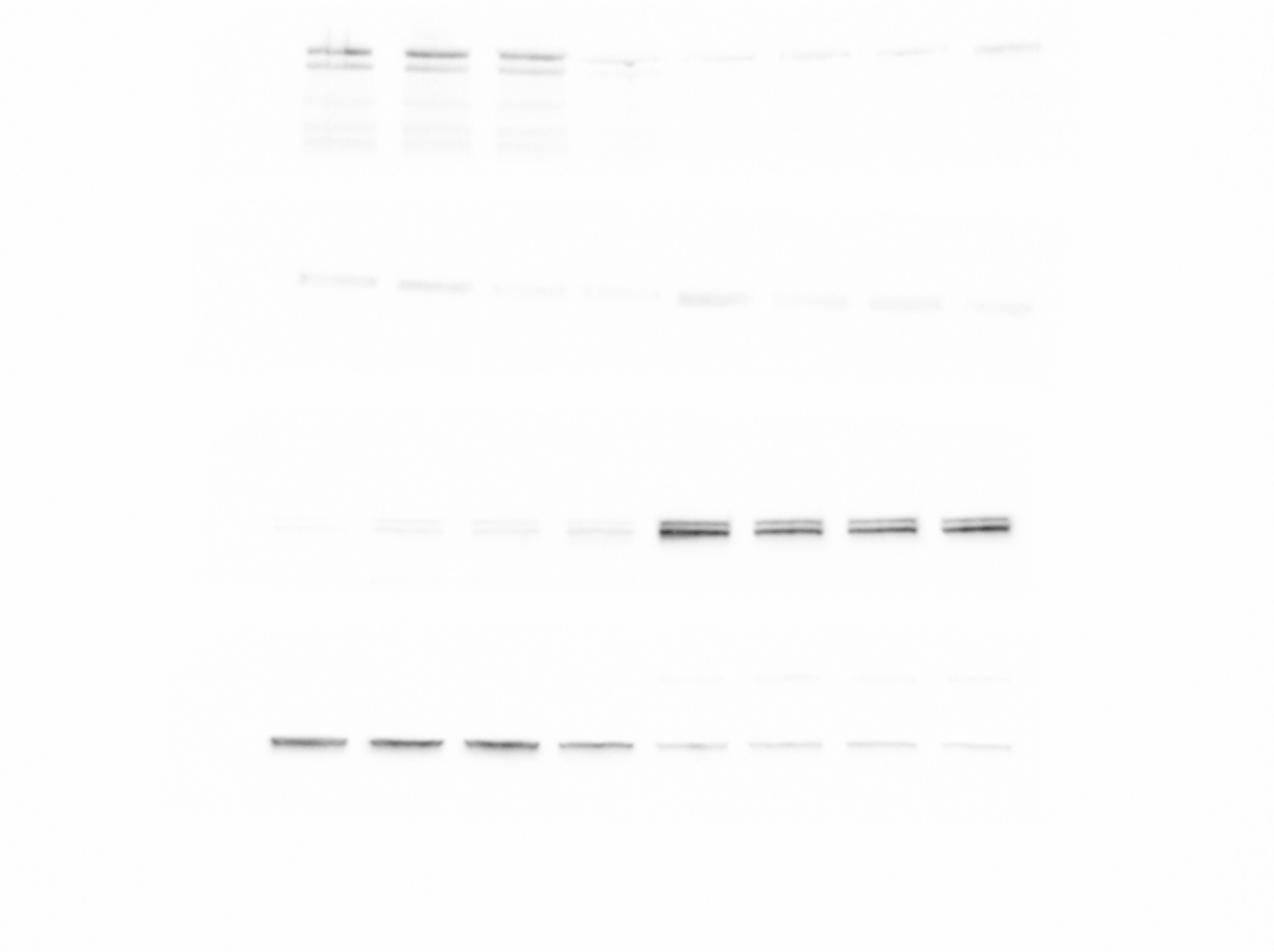

Supplement: Figure 2—figure supplement 1—source data 4. [file elife-73523-fig2-figsupp1-data4.zip › Raw blots/E14Tg2a/anti-BAF155.tif]

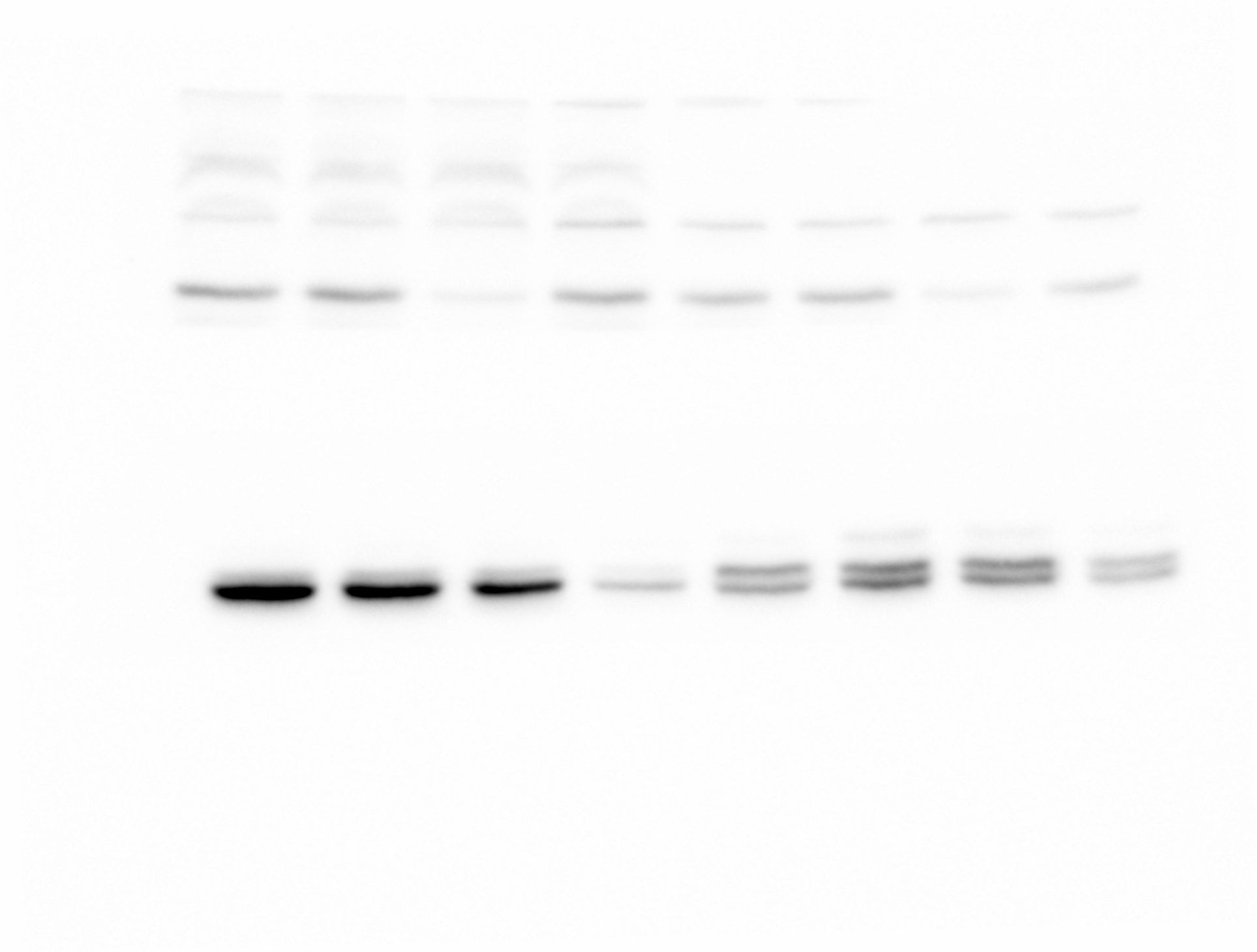

Supplement: Figure 2—figure supplement 1—source data 4. [file elife-73523-fig2-figsupp1-data4.zip › Raw blots/E14Tg2a/anti-IPMK.tif]

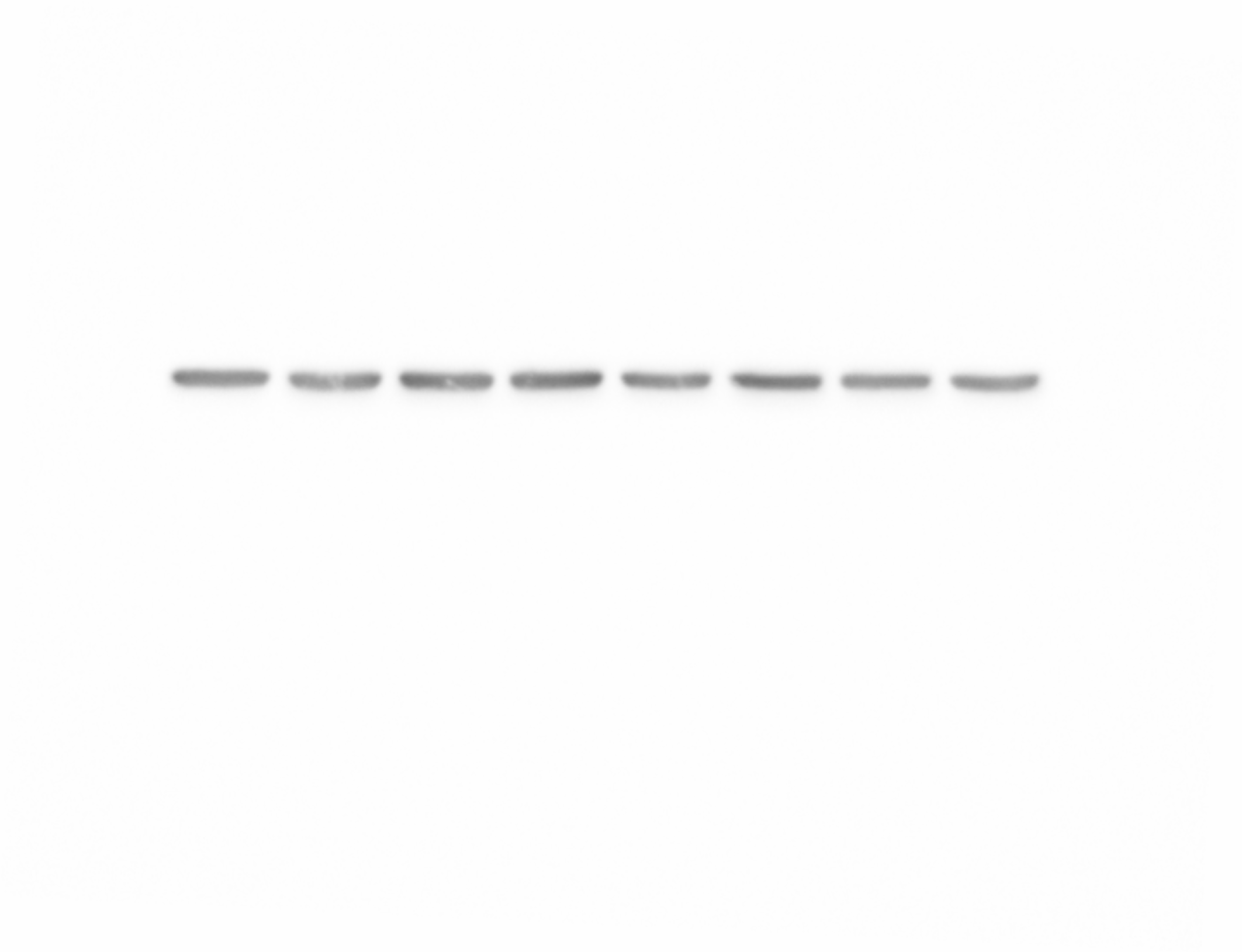

Supplement: Figure 2—figure supplement 1—source data 4. [file elife-73523-fig2-figsupp1-data4.zip › Raw blots/E14Tg2a/anti-alpha TUBULIN.tif]

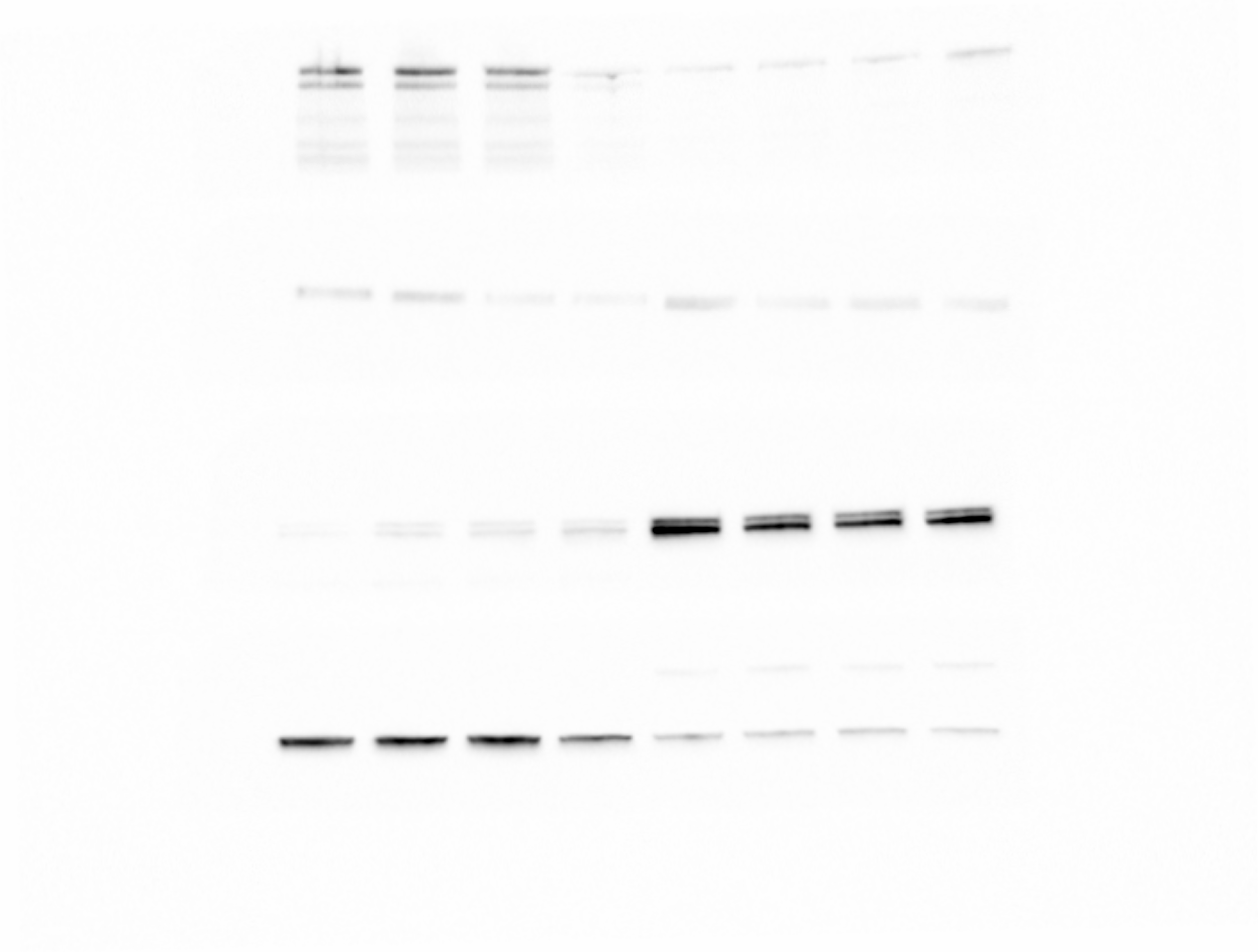

Supplement: Figure 2—figure supplement 1—source data 4. [file elife-73523-fig2-figsupp1-data4.zip › Raw blots/E14Tg2a/anti-BAF250.tif]

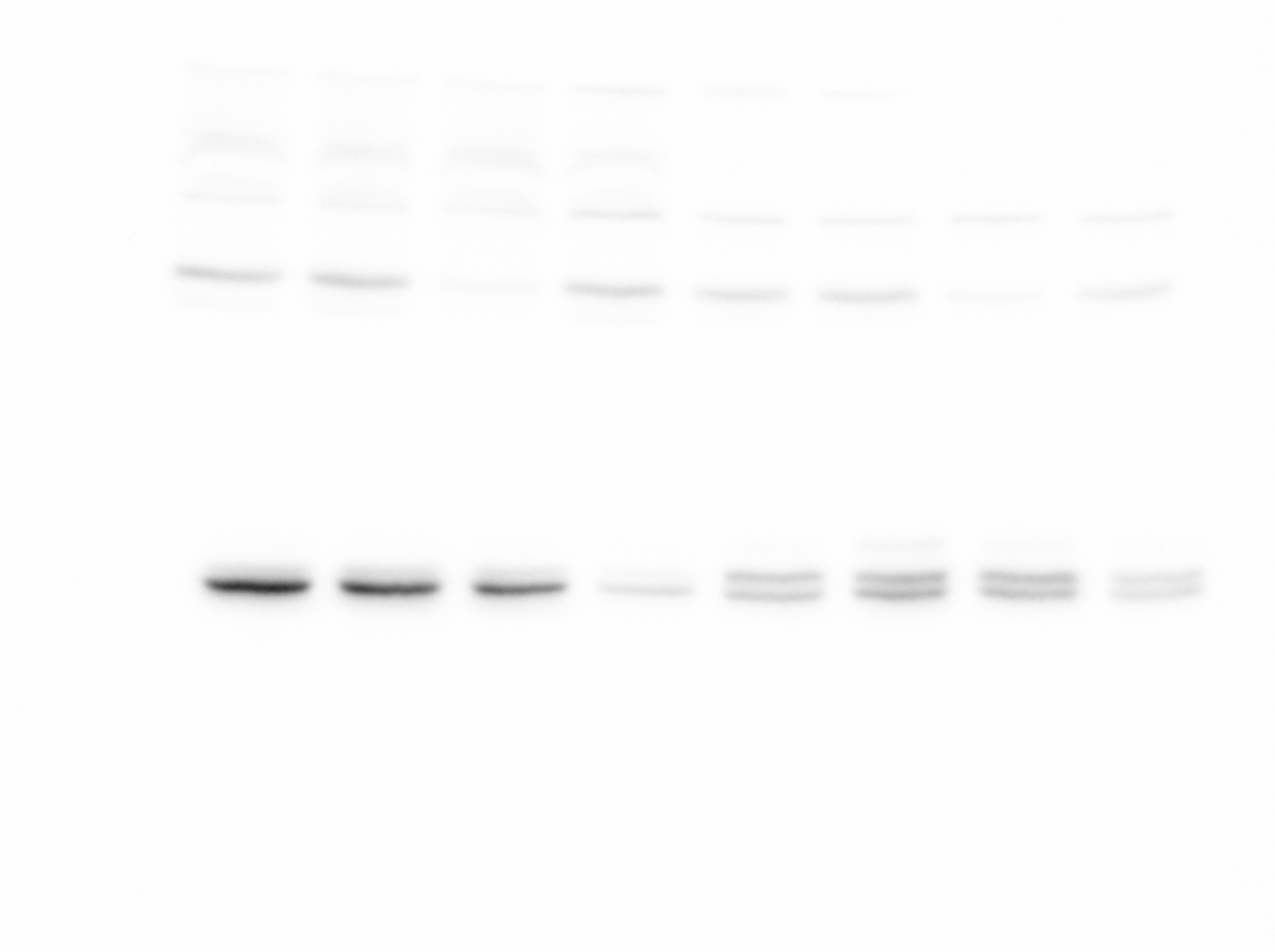

Supplement: Figure 2—figure supplement 1—source data 4. [file elife-73523-fig2-figsupp1-data4.zip › Raw blots/E14Tg2a/anti-SMARCB1.tif]

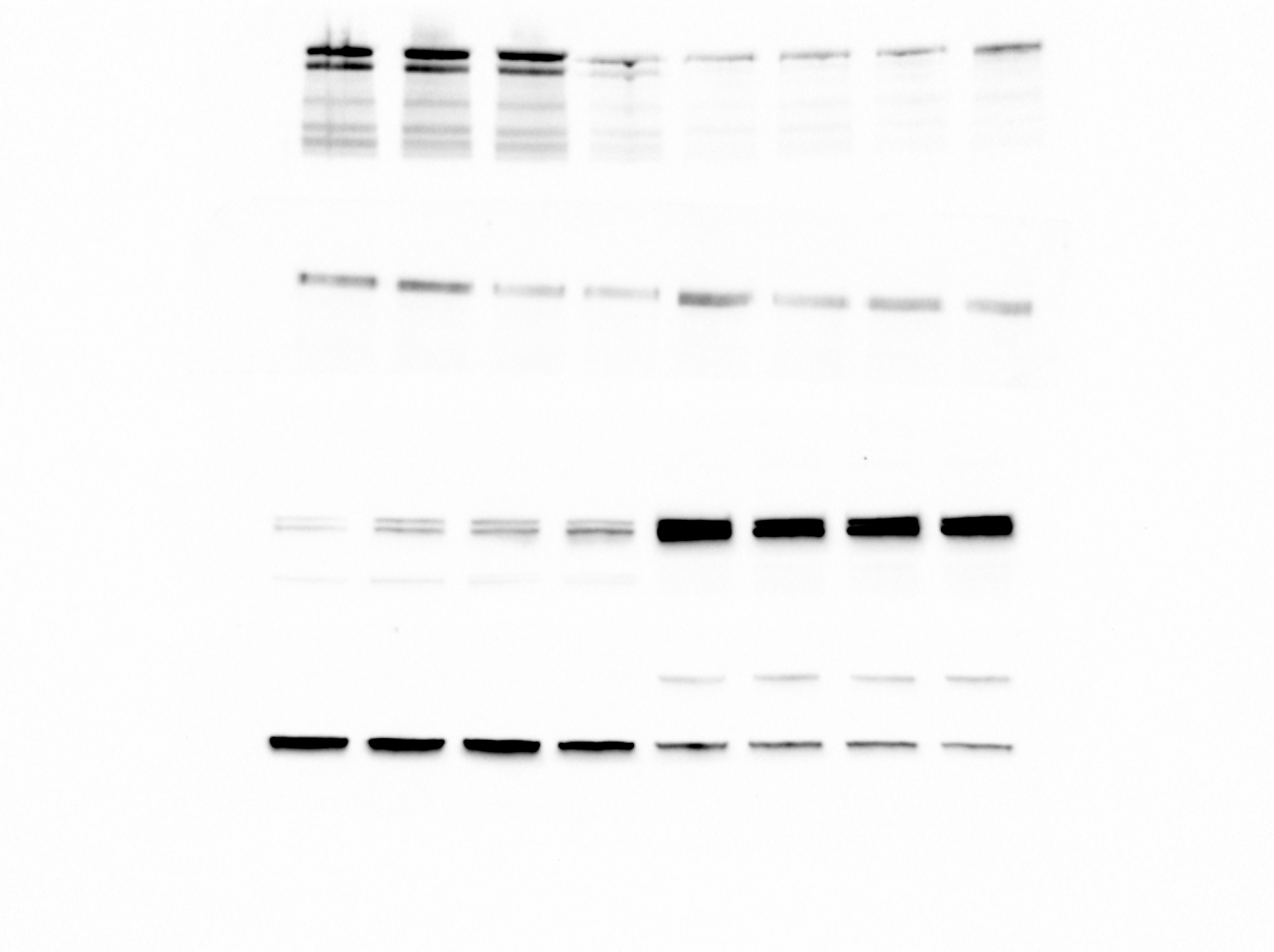

Supplement: Figure 2—figure supplement 1—source data 4. [file elife-73523-fig2-figsupp1-data4.zip › Raw blots/E14Tg2a/anti-BAF170.tif]

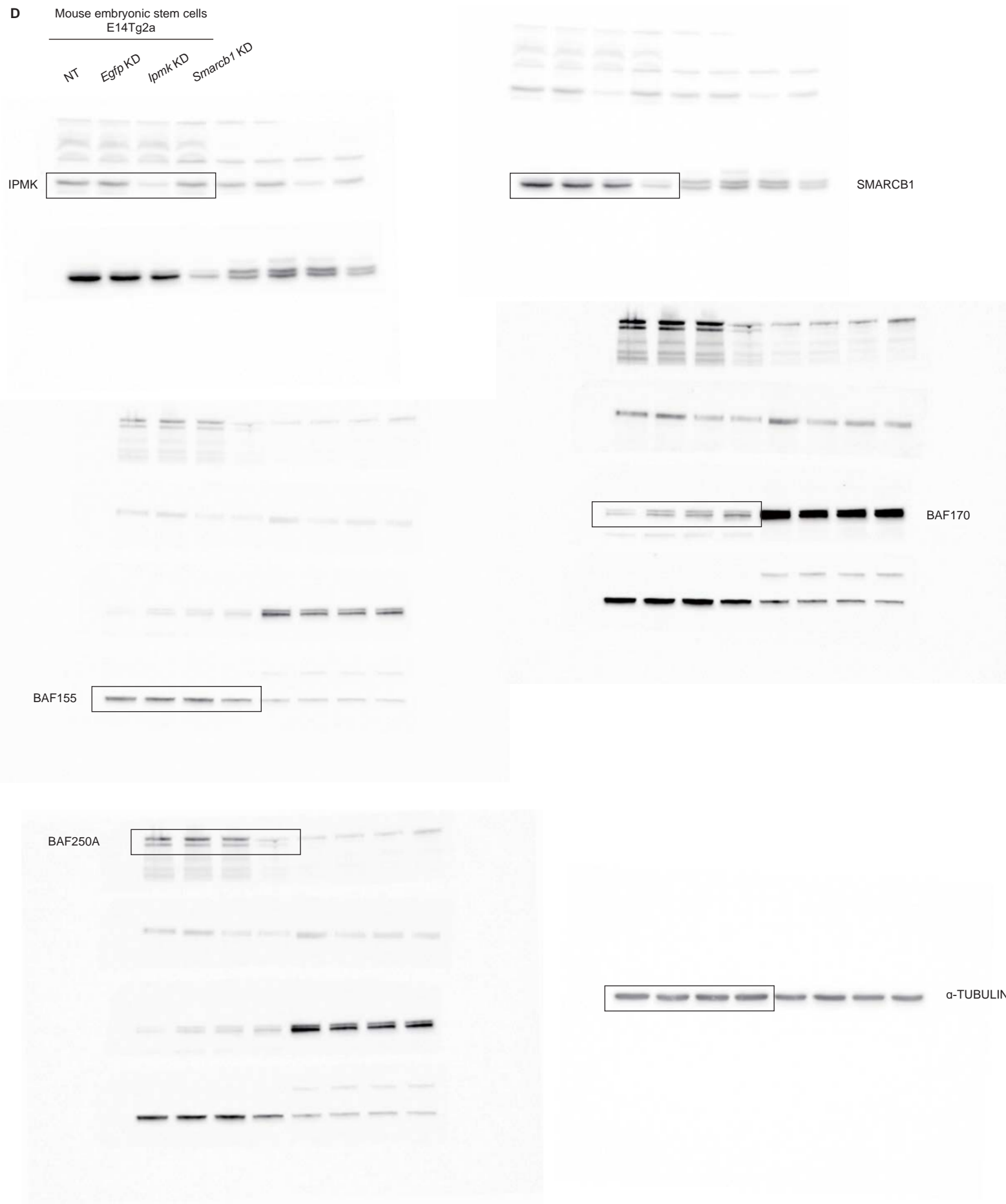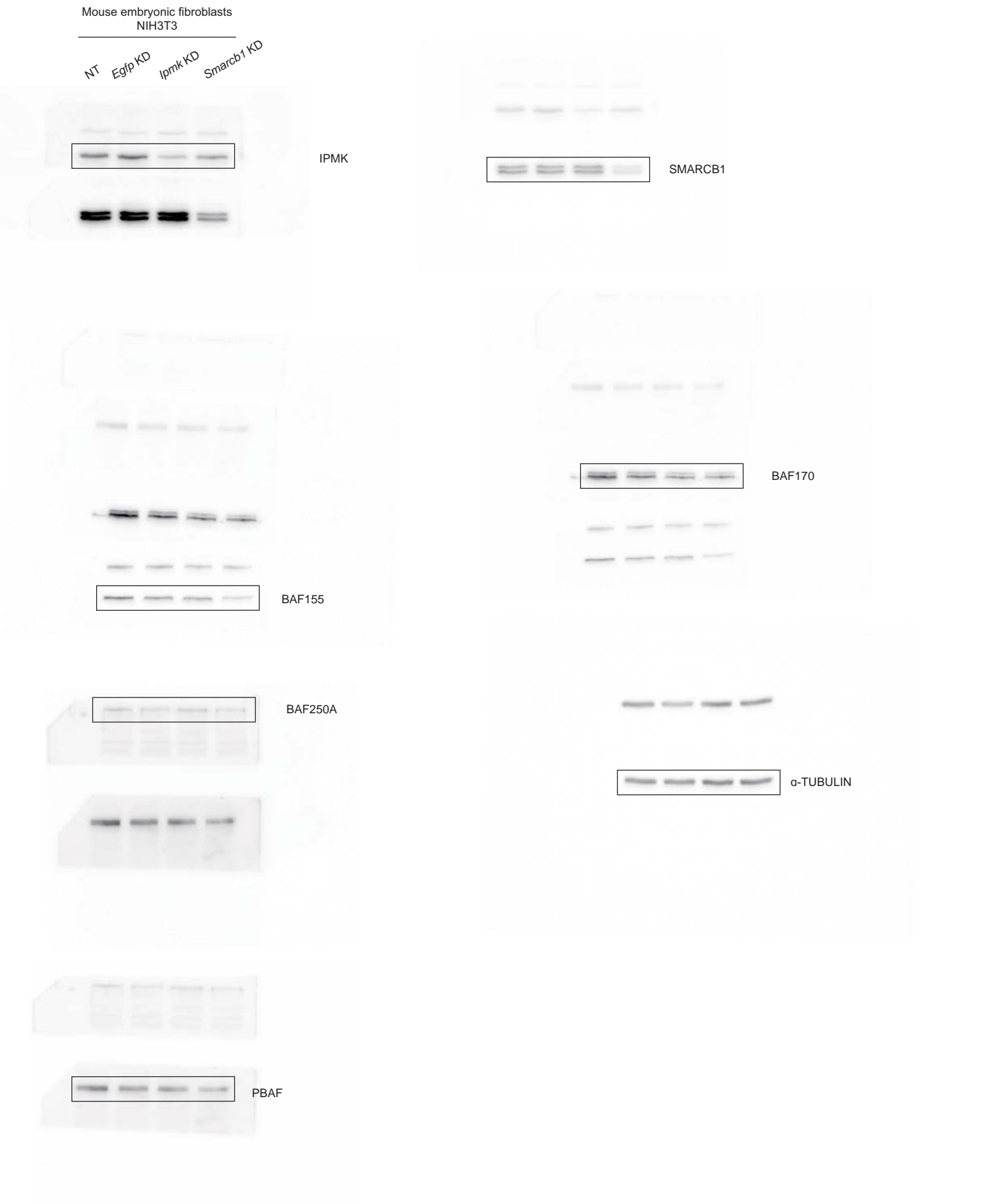

Supplement: Figure 2—figure supplement 1—source data 4. [file elife-73523-fig2-figsupp1-data4.zip › Labelled blots.pdf]

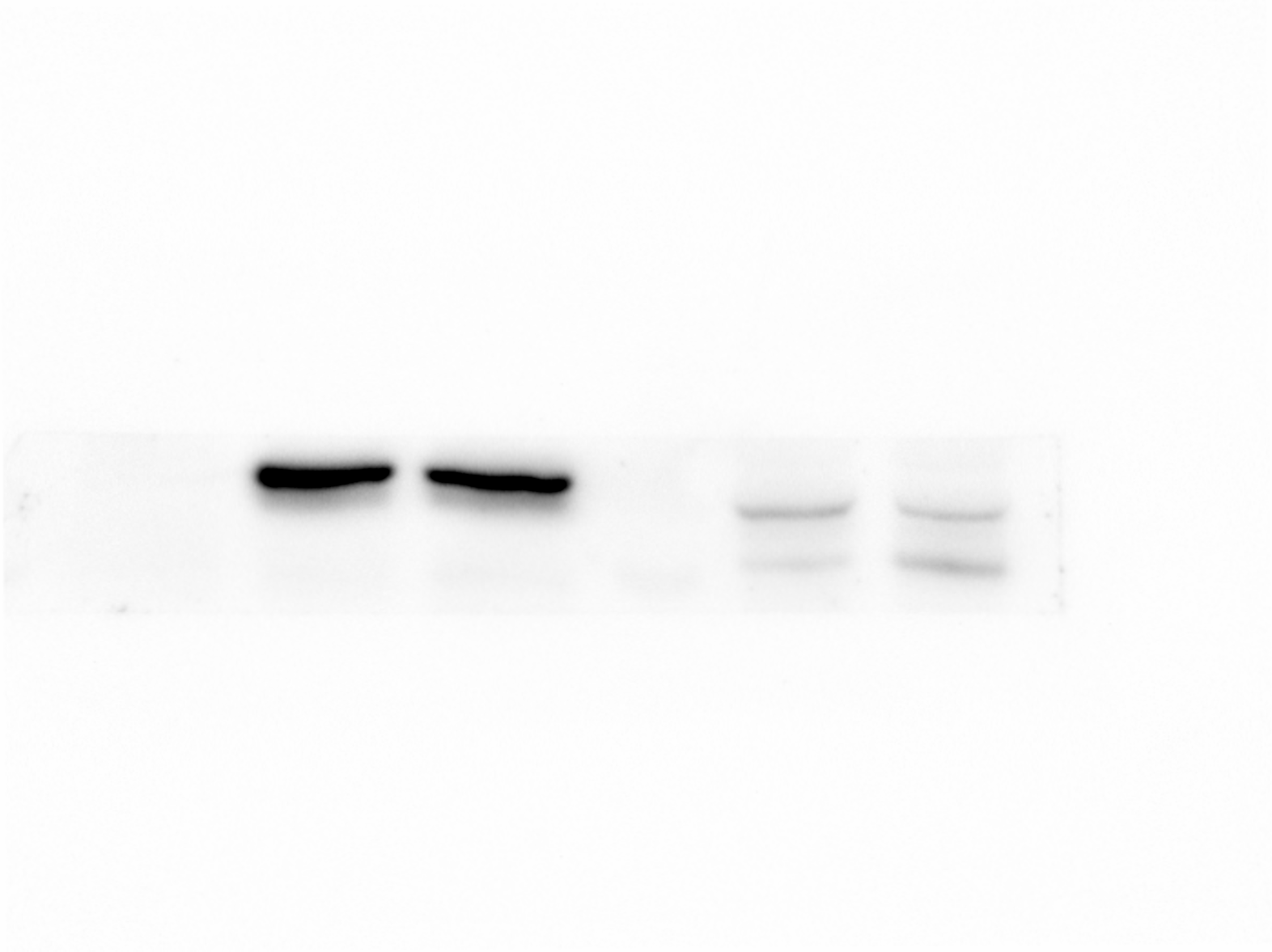

Supplement: Figure 2—figure supplement 1—source data 5. [file elife-73523-fig2-figsupp1-data5.zip › Raw blots/Input_ anti-IPMK.tif]

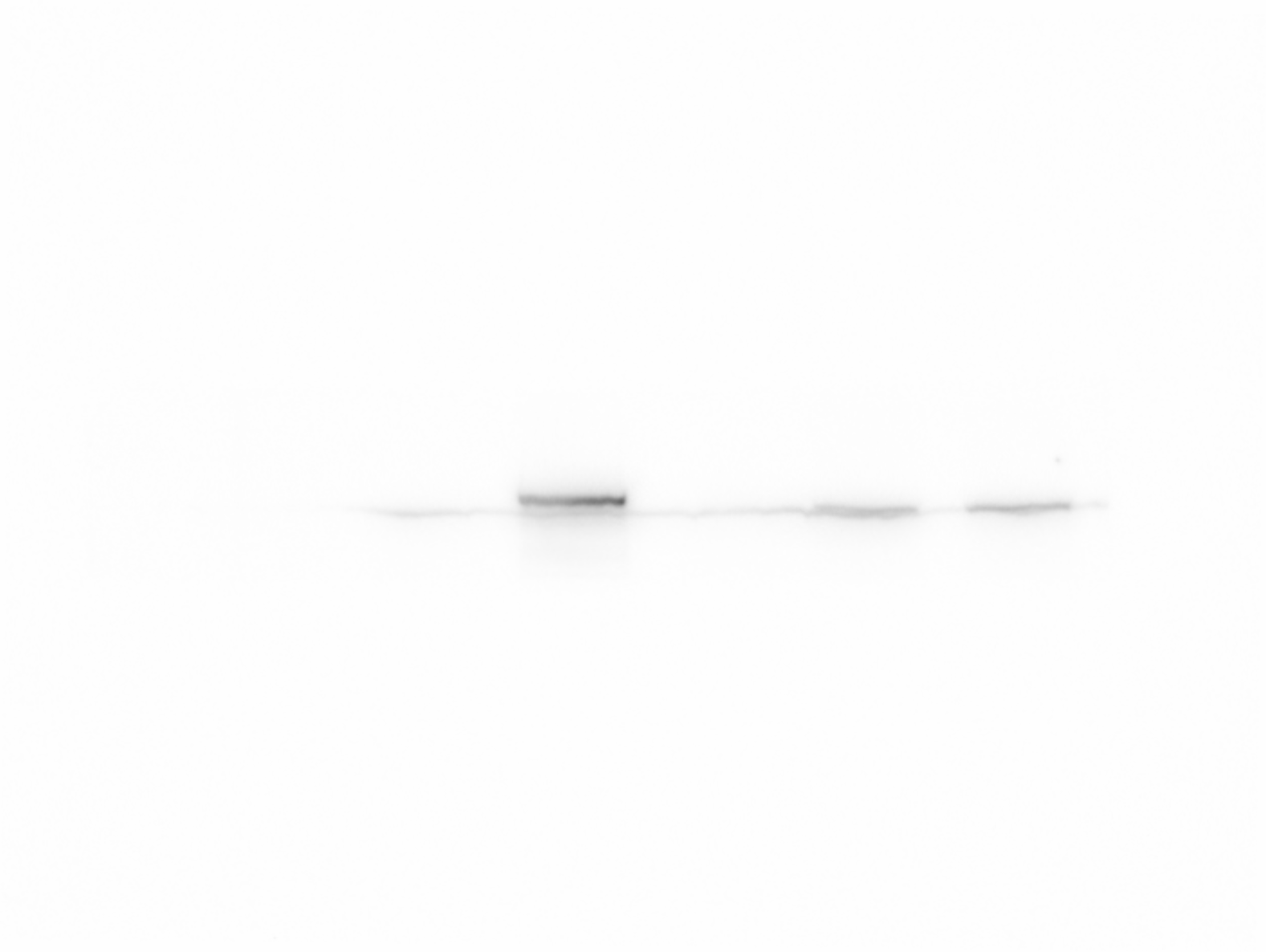

Supplement: Figure 2—figure supplement 1—source data 5. [file elife-73523-fig2-figsupp1-data5.zip › Raw blots/anti-BRG1.tif]

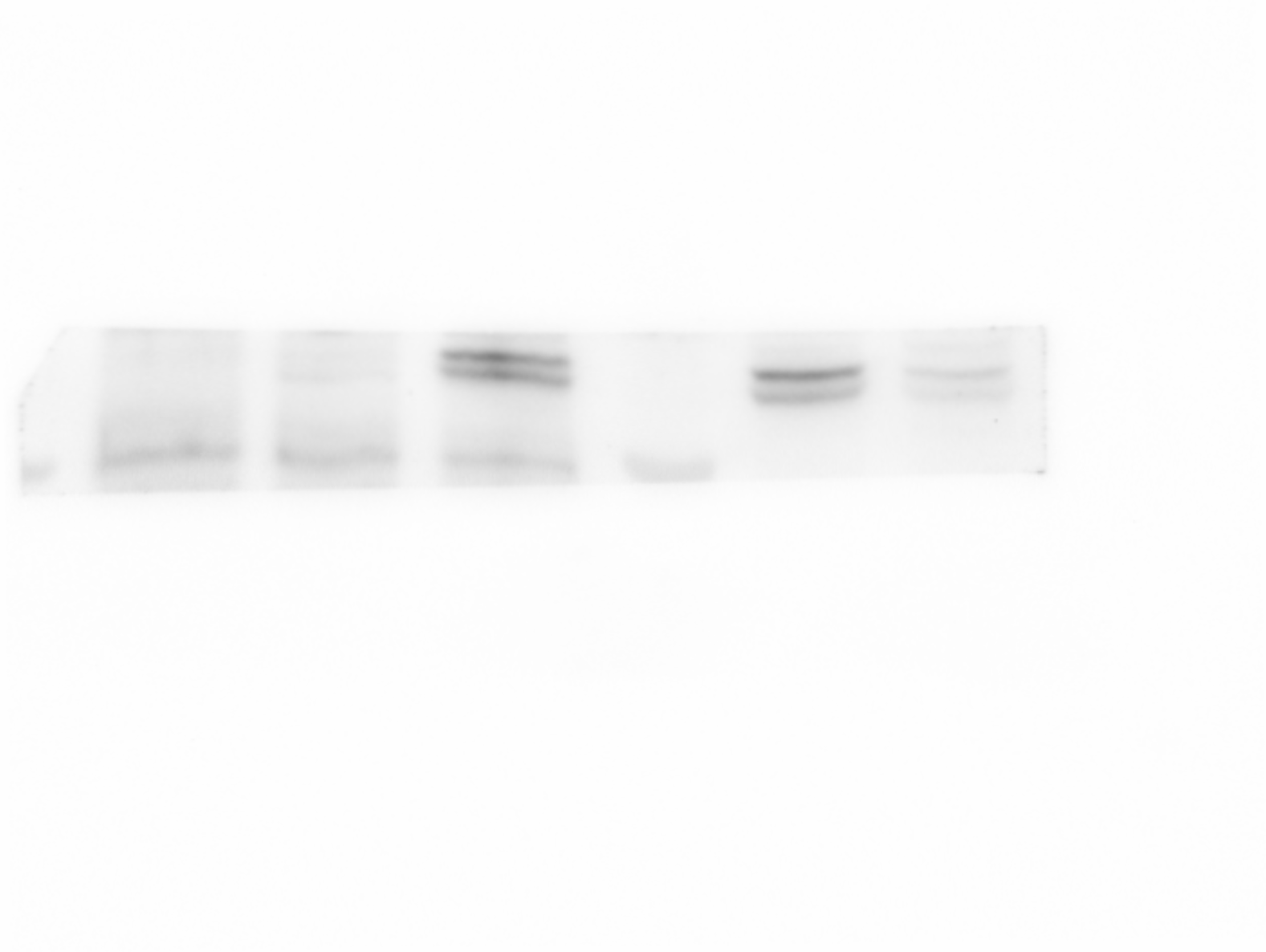

Supplement: Figure 2—figure supplement 1—source data 5. [file elife-73523-fig2-figsupp1-data5.zip › Raw blots/anti-SMARCB1.tif]

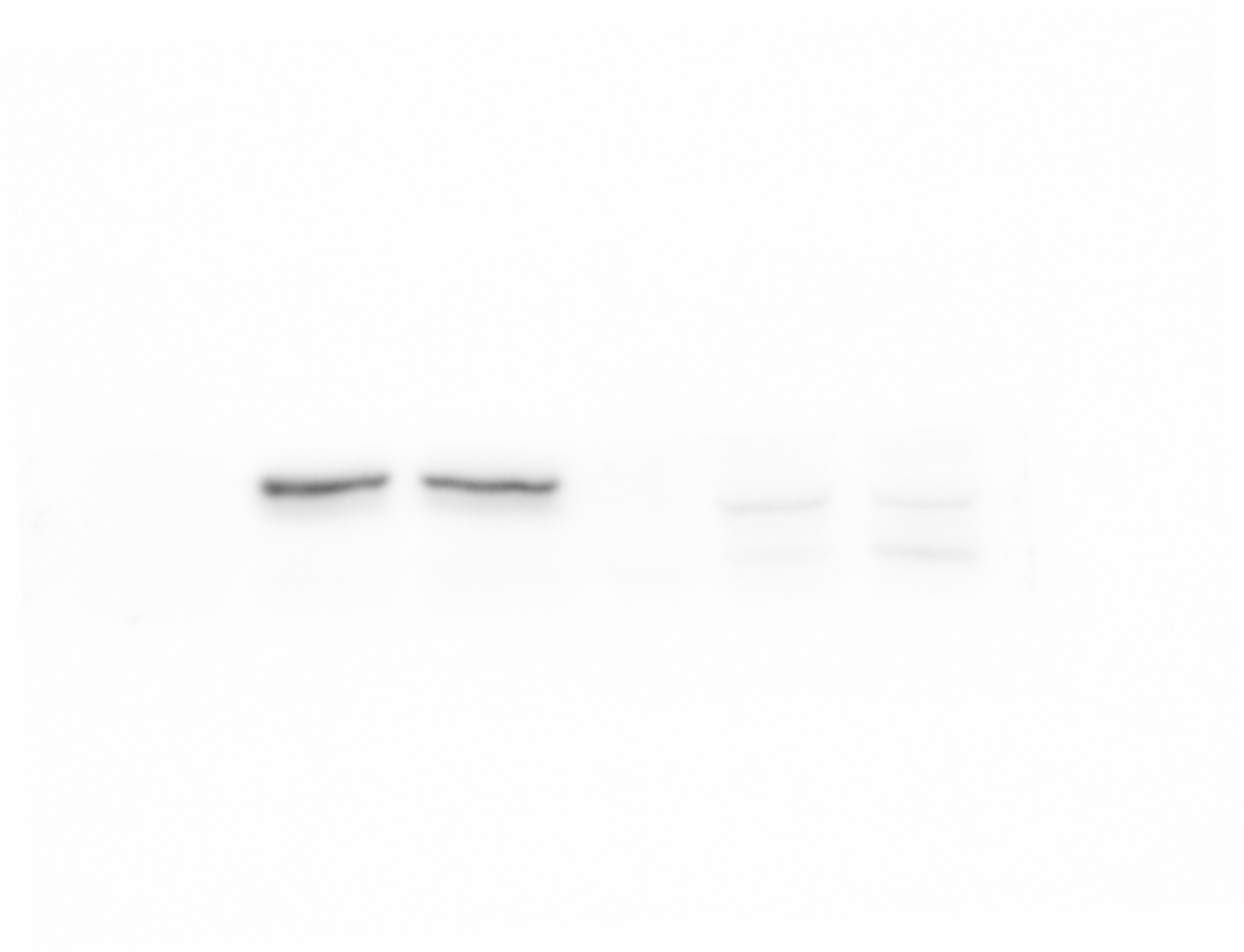

Supplement: Figure 2—figure supplement 1—source data 5. [file elife-73523-fig2-figsupp1-data5.zip › Raw blots/IP_ anti-IPMK.tif]

E

IP : IgG    IP : IPMK

Egfp KD    Smarcb1 KD    Egfp KD

IPMK

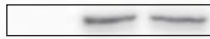

SMARCB1

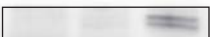

BRG1

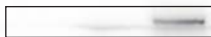

Input

Smarcb1 KD    Egfp KD

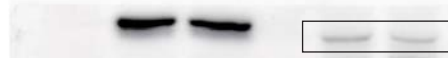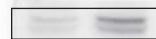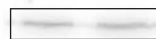

Supplement: Figure 2—figure supplement 1—source data 5. [file elife-73523-fig2-figsupp1-data5.zip › Labelled blots.pdf]

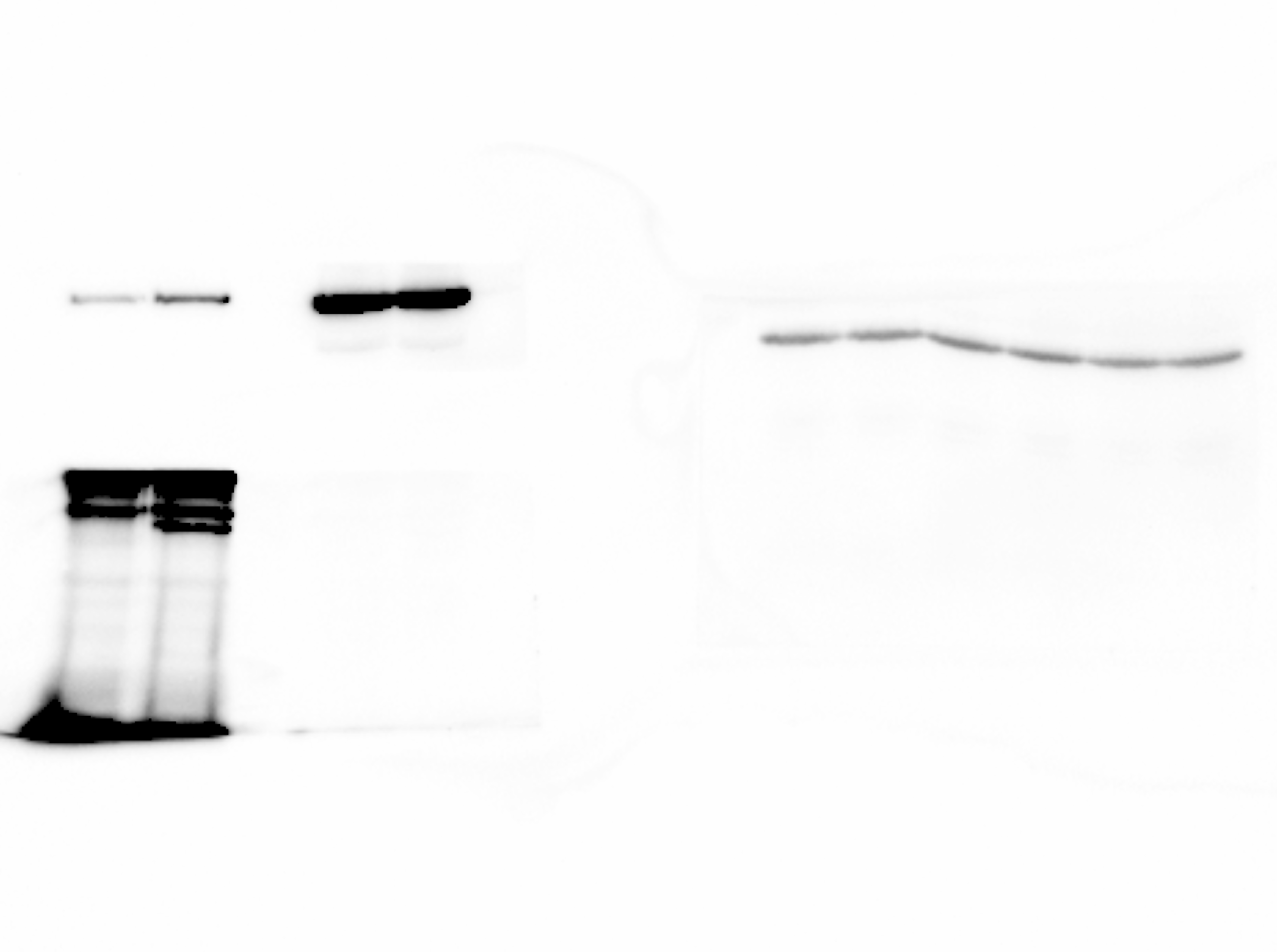

Supplement: Figure 2—figure supplement 1—source data 6. [file elife-73523-fig2-figsupp1-data6.zip › Raw blots/IP_ anti-GST.tif]

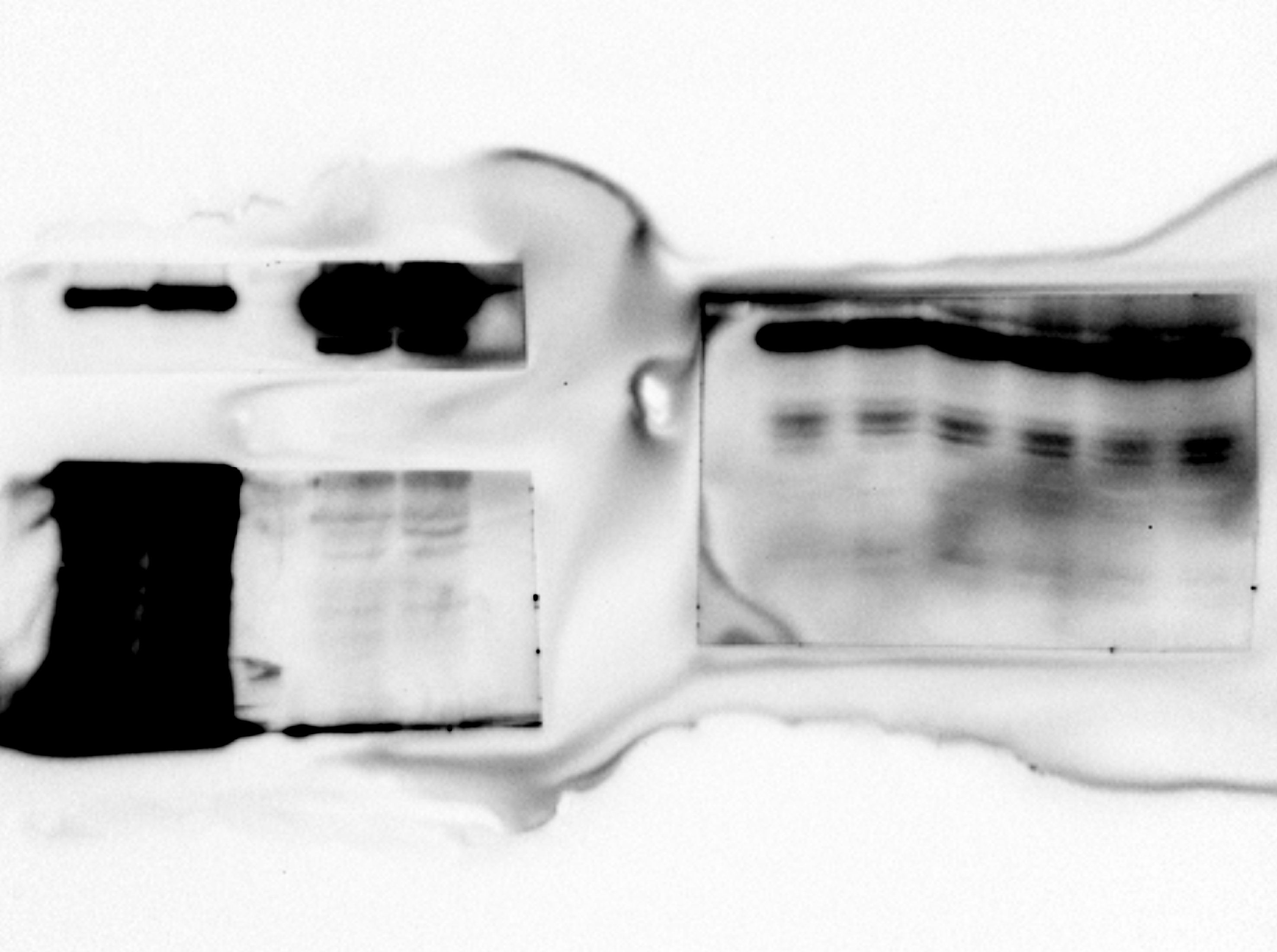

Supplement: Figure 2—figure supplement 1—source data 6. [file elife-73523-fig2-figsupp1-data6.zip › Raw blots/Input_ anti-FLAG.tif]

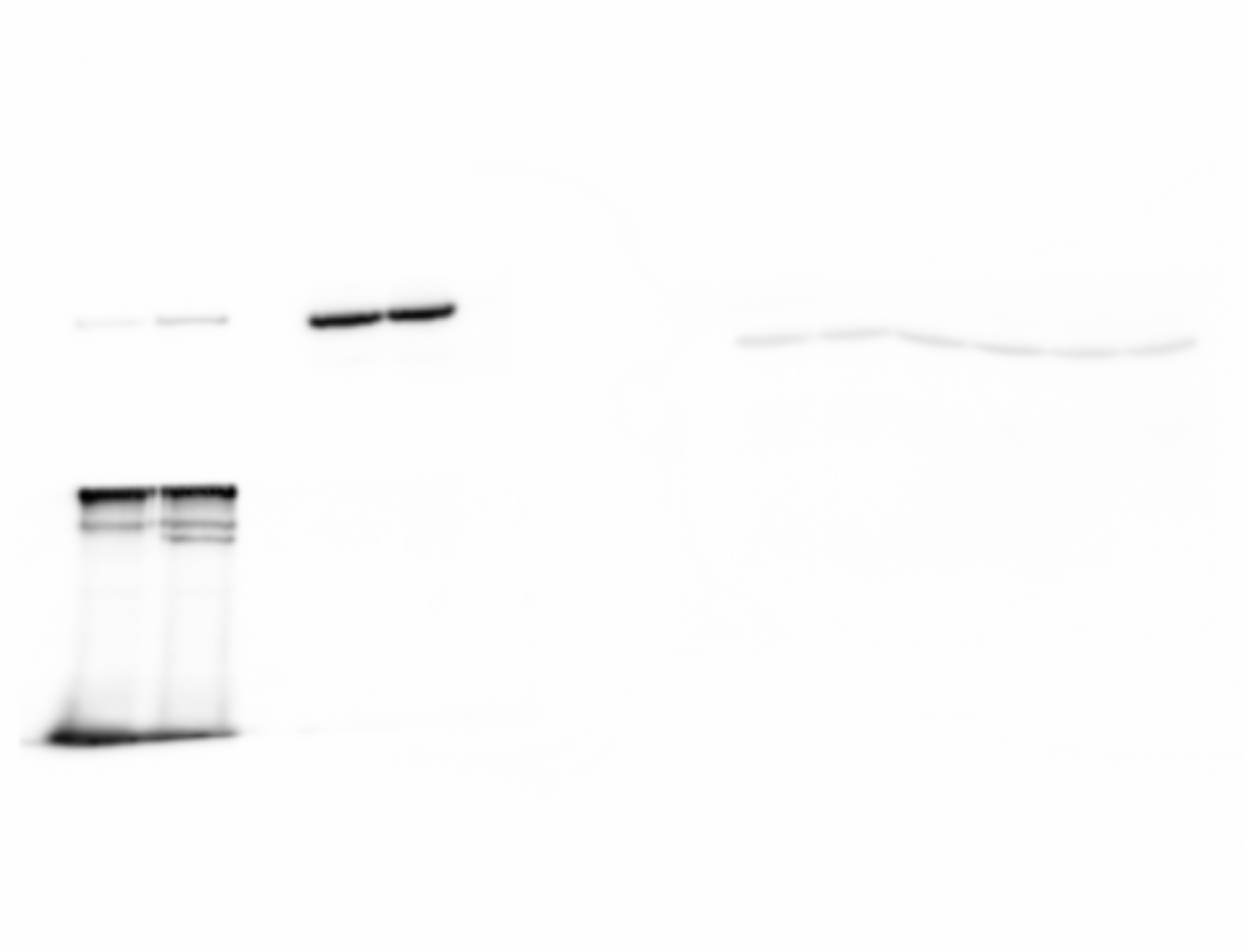

Supplement: Figure 2—figure supplement 1—source data 6. [file elife-73523-fig2-figsupp1-data6.zip › Raw blots/IP_ anti-FLAG.tif]

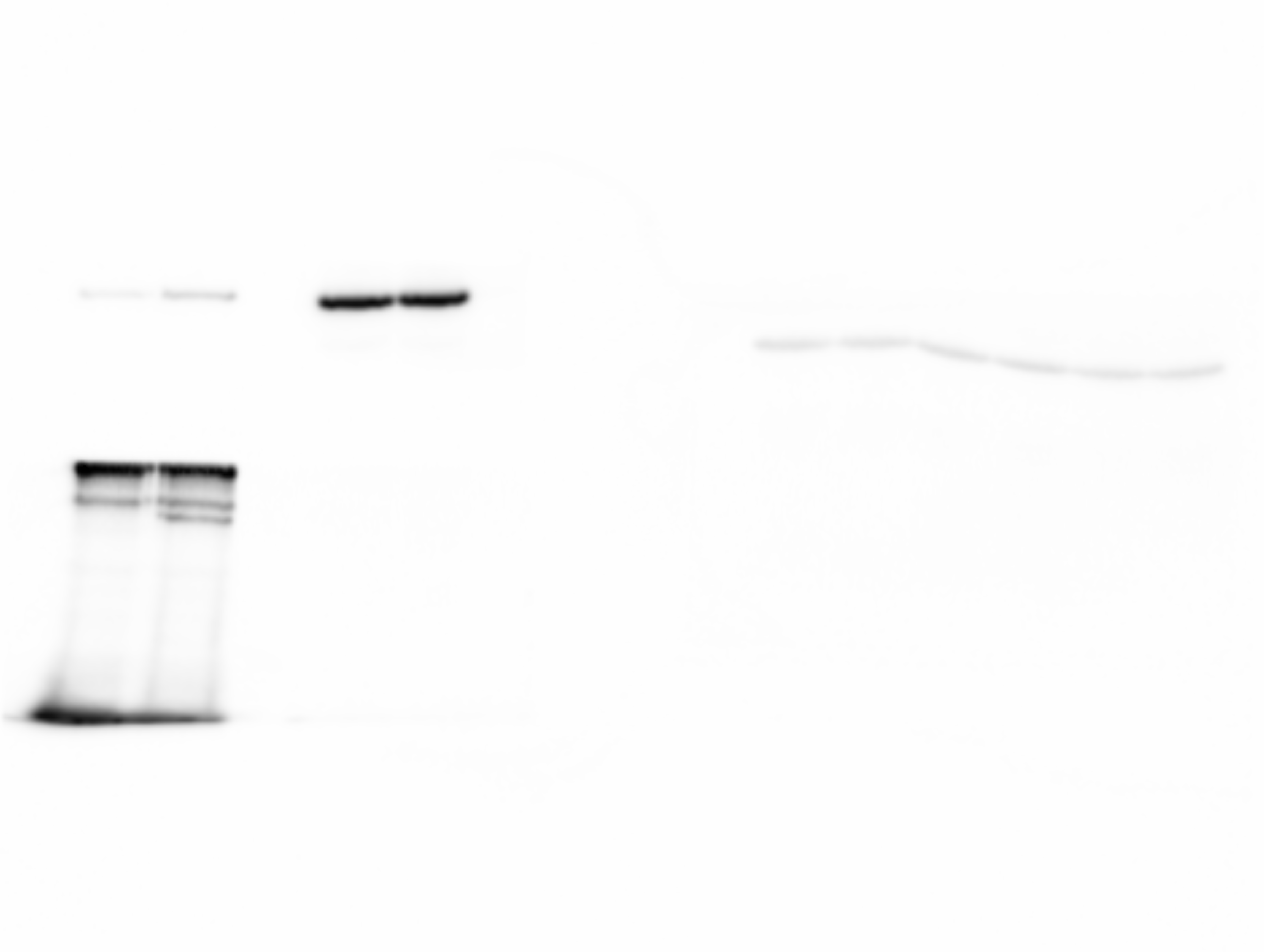

Supplement: Figure 2—figure supplement 1—source data 6. [file elife-73523-fig2-figsupp1-data6.zip › Raw blots/Input_ anti-GST.tif]

**F**

|                |   |   |
|----------------|---|---|
| FLAG :         | + | - |
| FLAG-SMARCB1 : | - | + |
| GST-IPMK :     | + | + |

IP :  
FLAG

GST

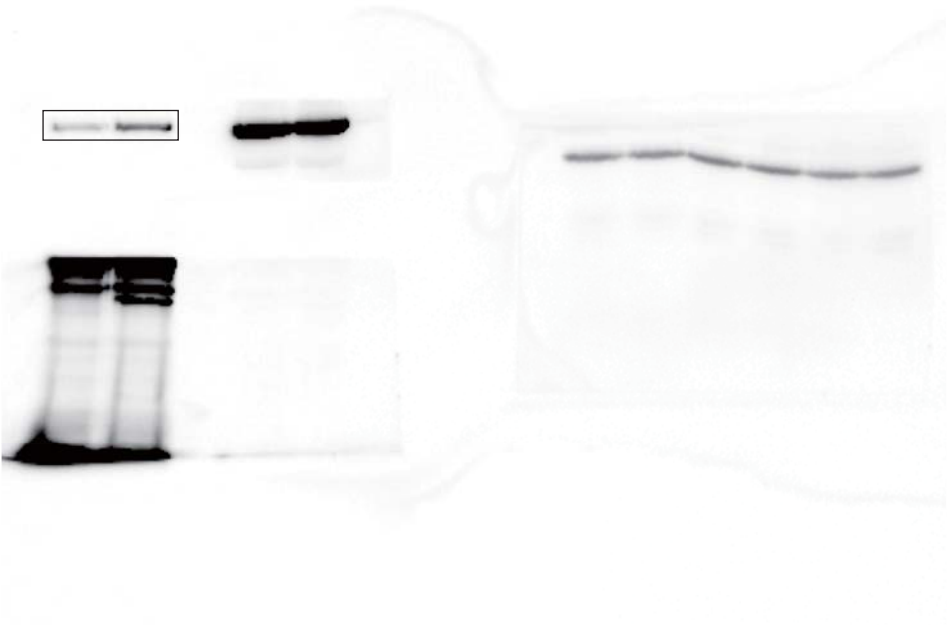

IP :  
FLAG

FLAG

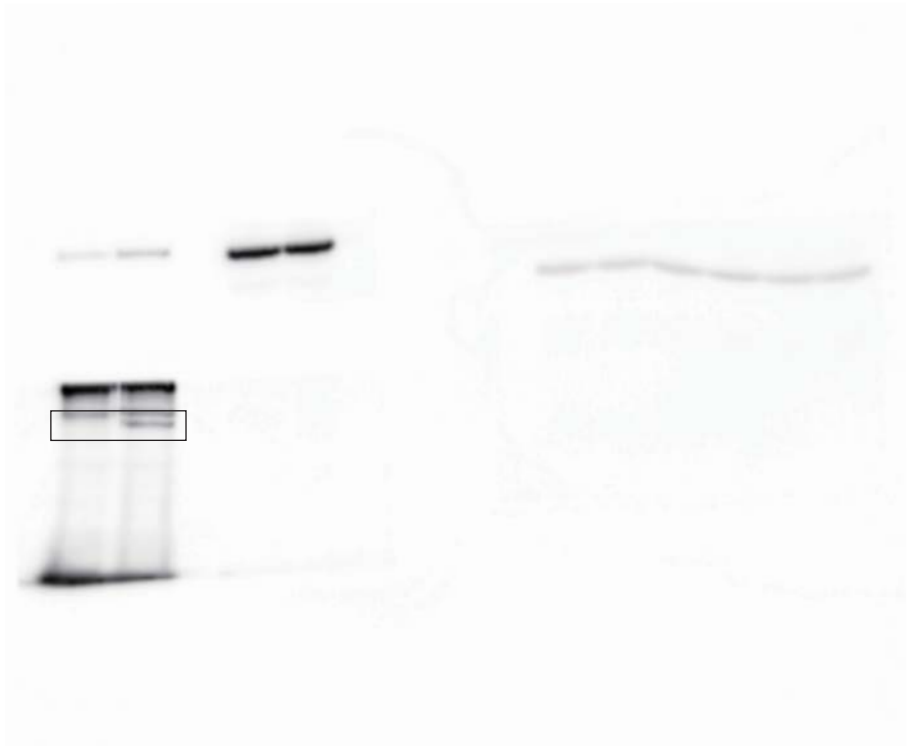

Input

GST

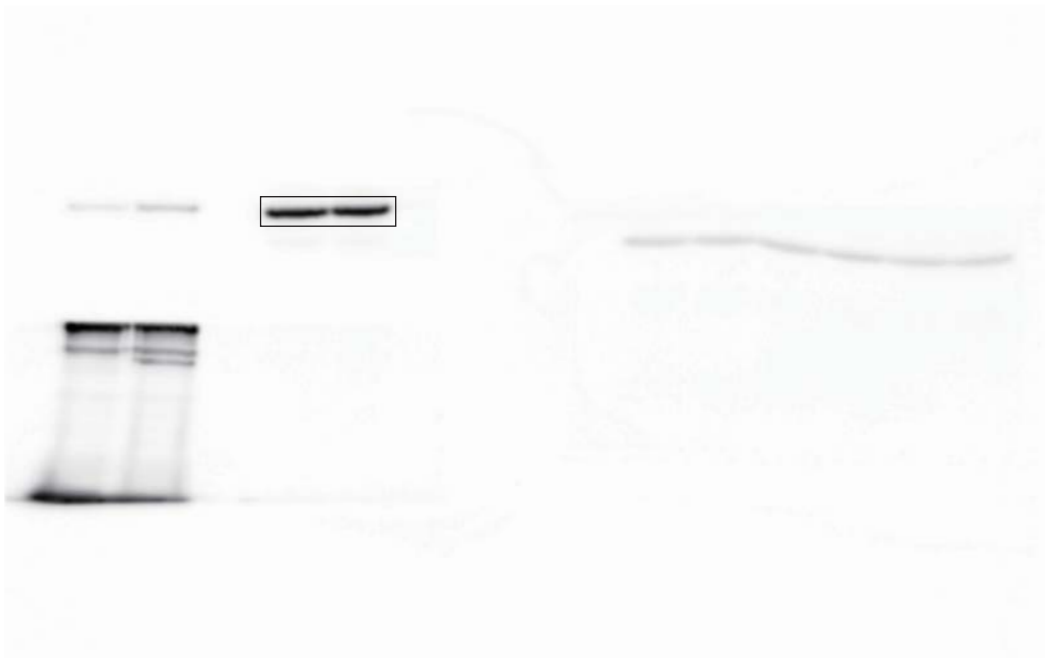

Input

FLAG

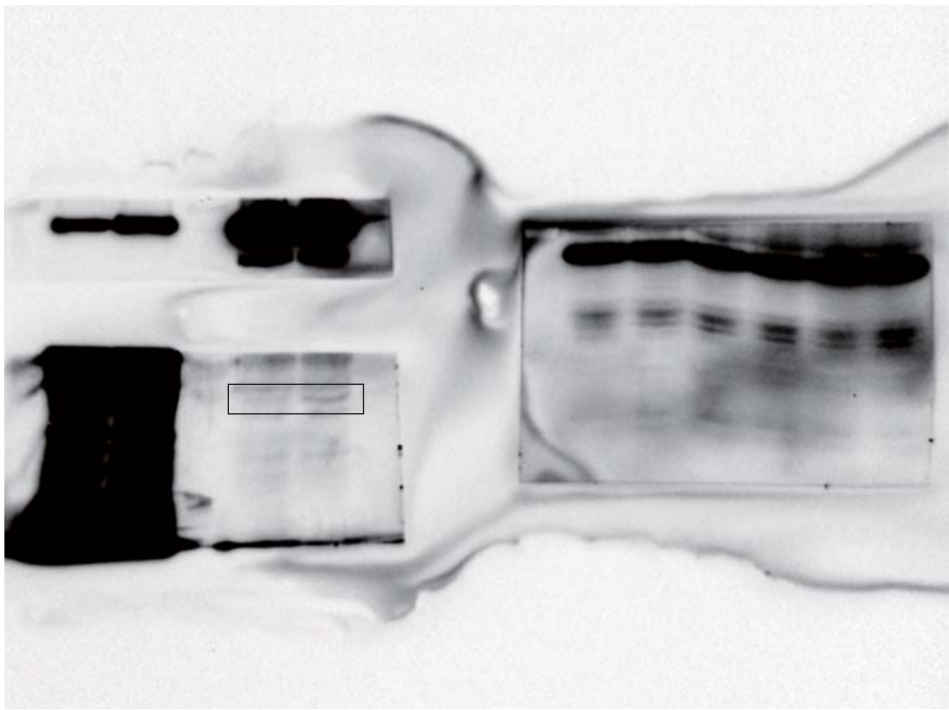

Supplement: Figure 2—figure supplement 1—source data 6. [file elife-73523-fig2-figsupp1-data6.zip › Labelled blots.pdf]

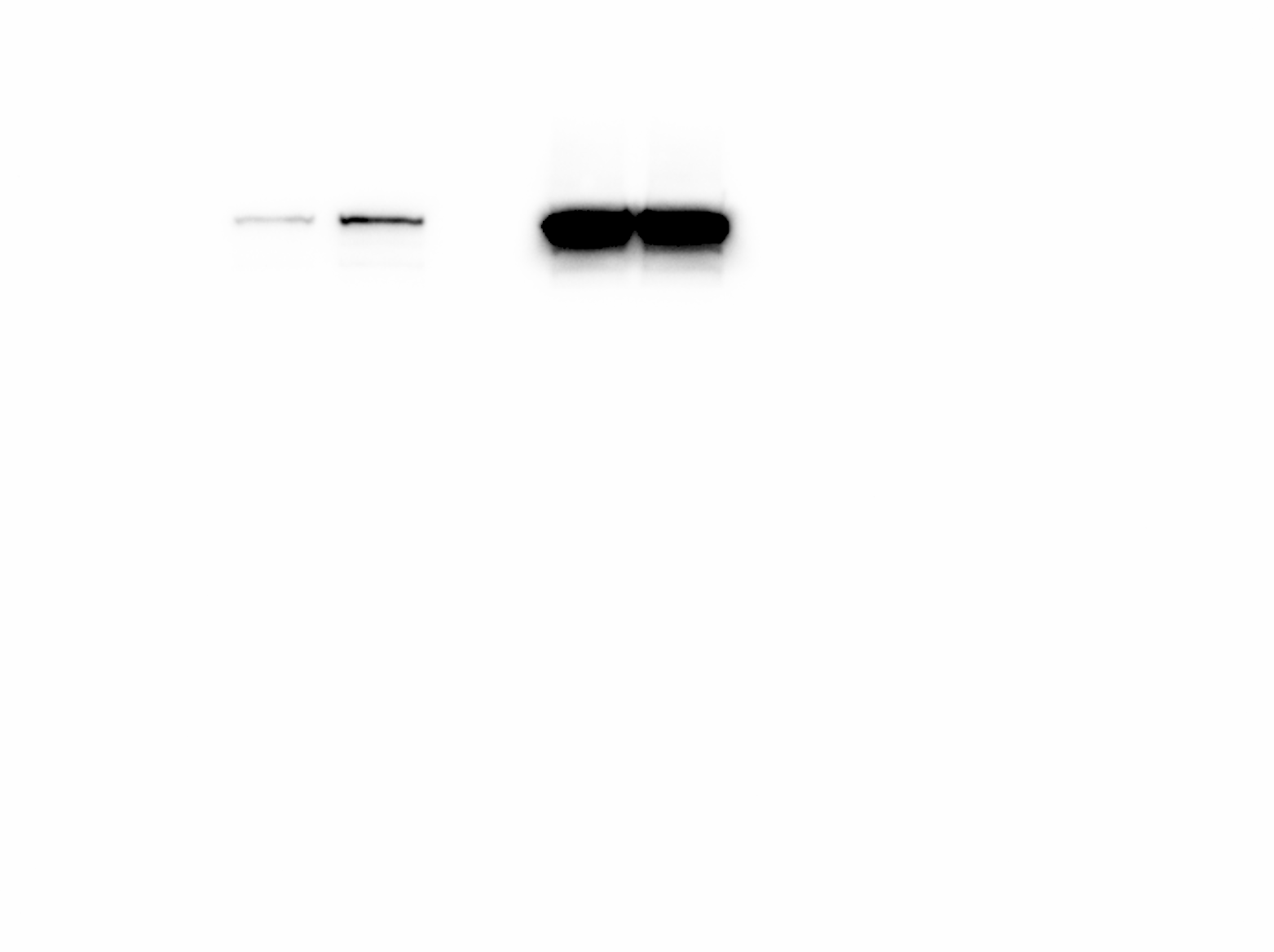

Supplement: Figure 2—figure supplement 1—source data 7. [file elife-73523-fig2-figsupp1-data7.zip › Raw blots/Pulldown_ anti-BAF155.tif]

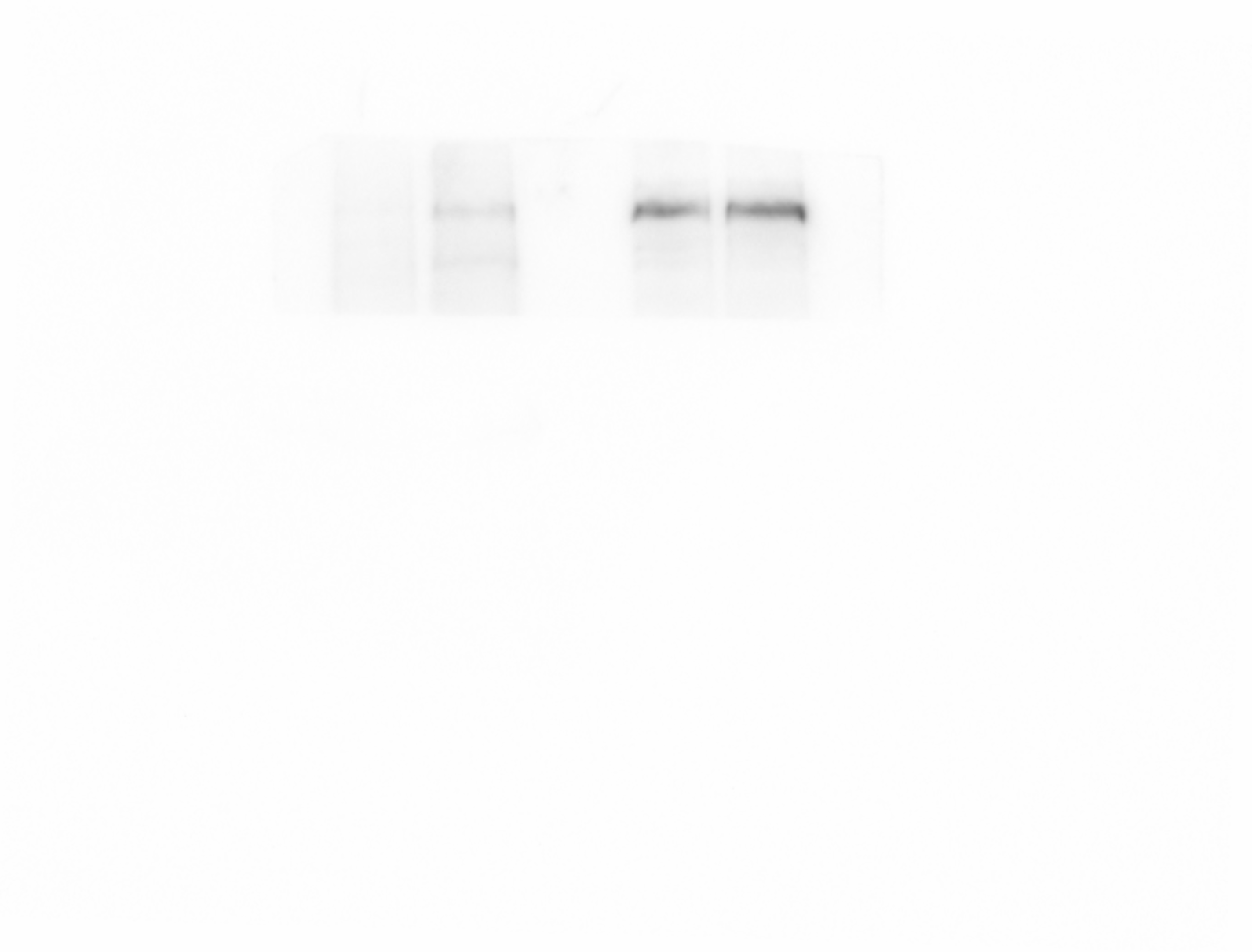

Supplement: Figure 2—figure supplement 1—source data 7. [file elife-73523-fig2-figsupp1-data7.zip › Raw blots/Input_ anti-BAF180.tif]

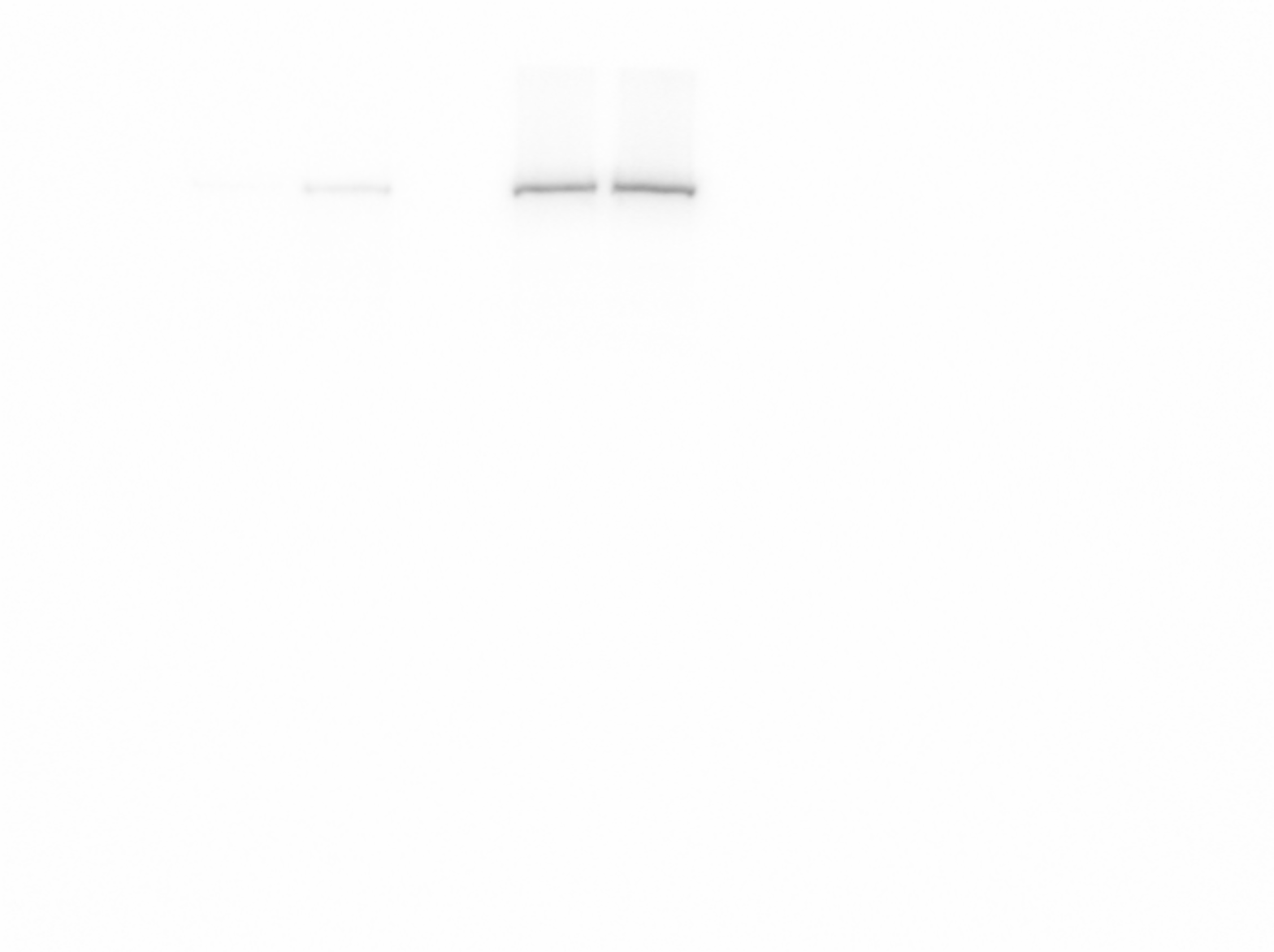

Supplement: Figure 2—figure supplement 1—source data 7. [file elife-73523-fig2-figsupp1-data7.zip › Raw blots/Input_ anti-BRG1.tif]

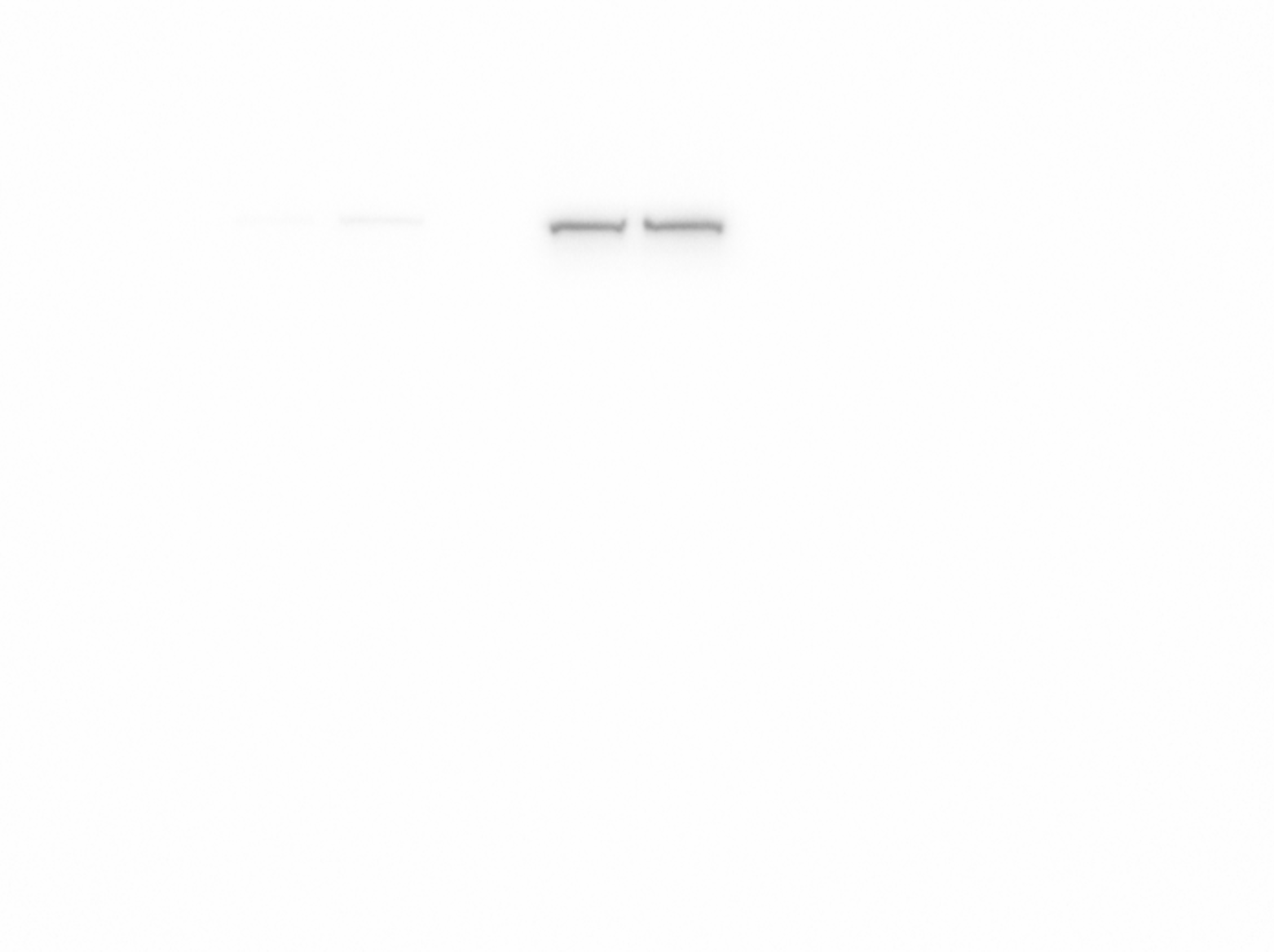

Supplement: Figure 2—figure supplement 1—source data 7. [file elife-73523-fig2-figsupp1-data7.zip › Raw blots/Input_ anti-BAF155.tif]

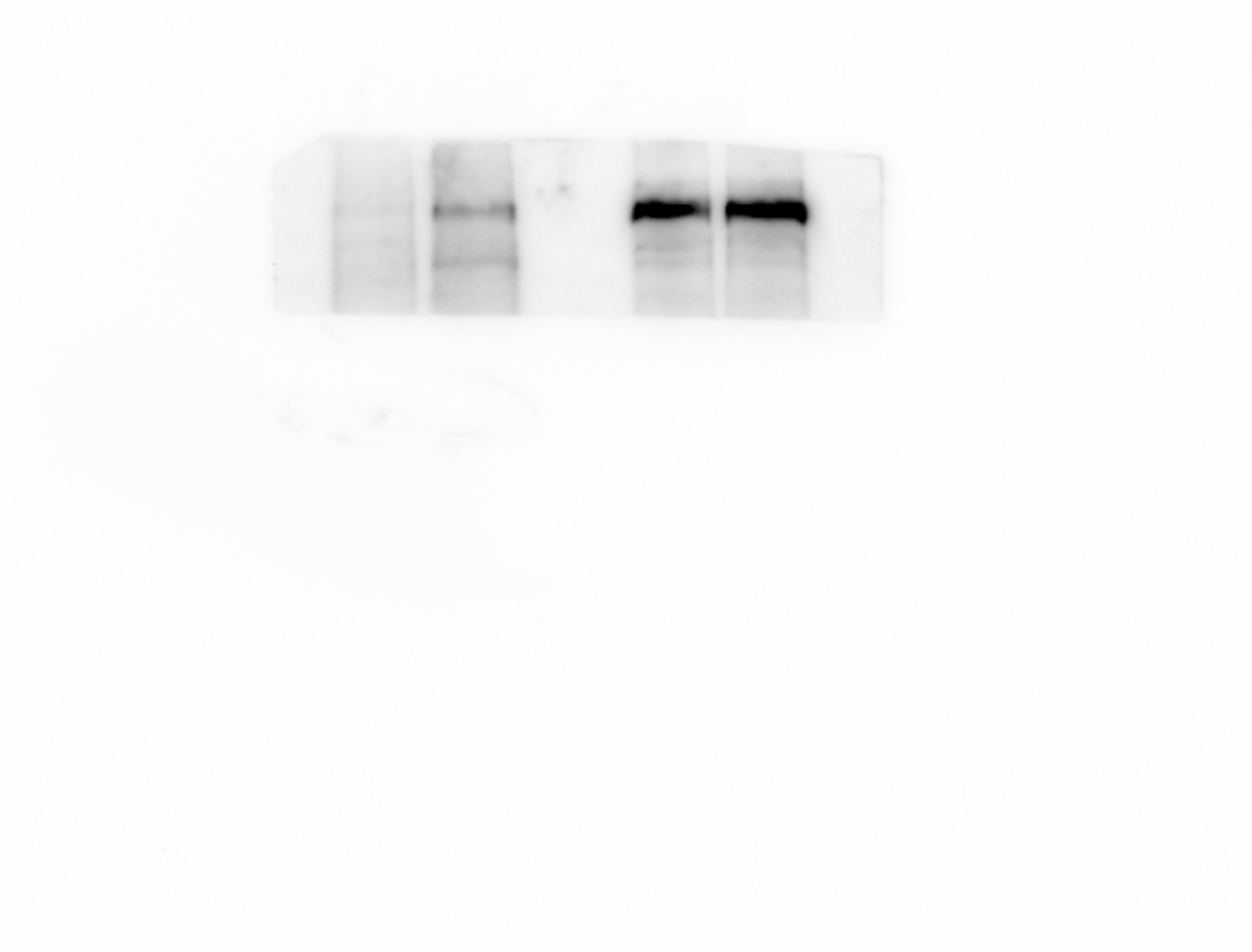

Supplement: Figure 2—figure supplement 1—source data 7. [file elife-73523-fig2-figsupp1-data7.zip › Raw blots/Pulldown_ anti-BAF180.tif]

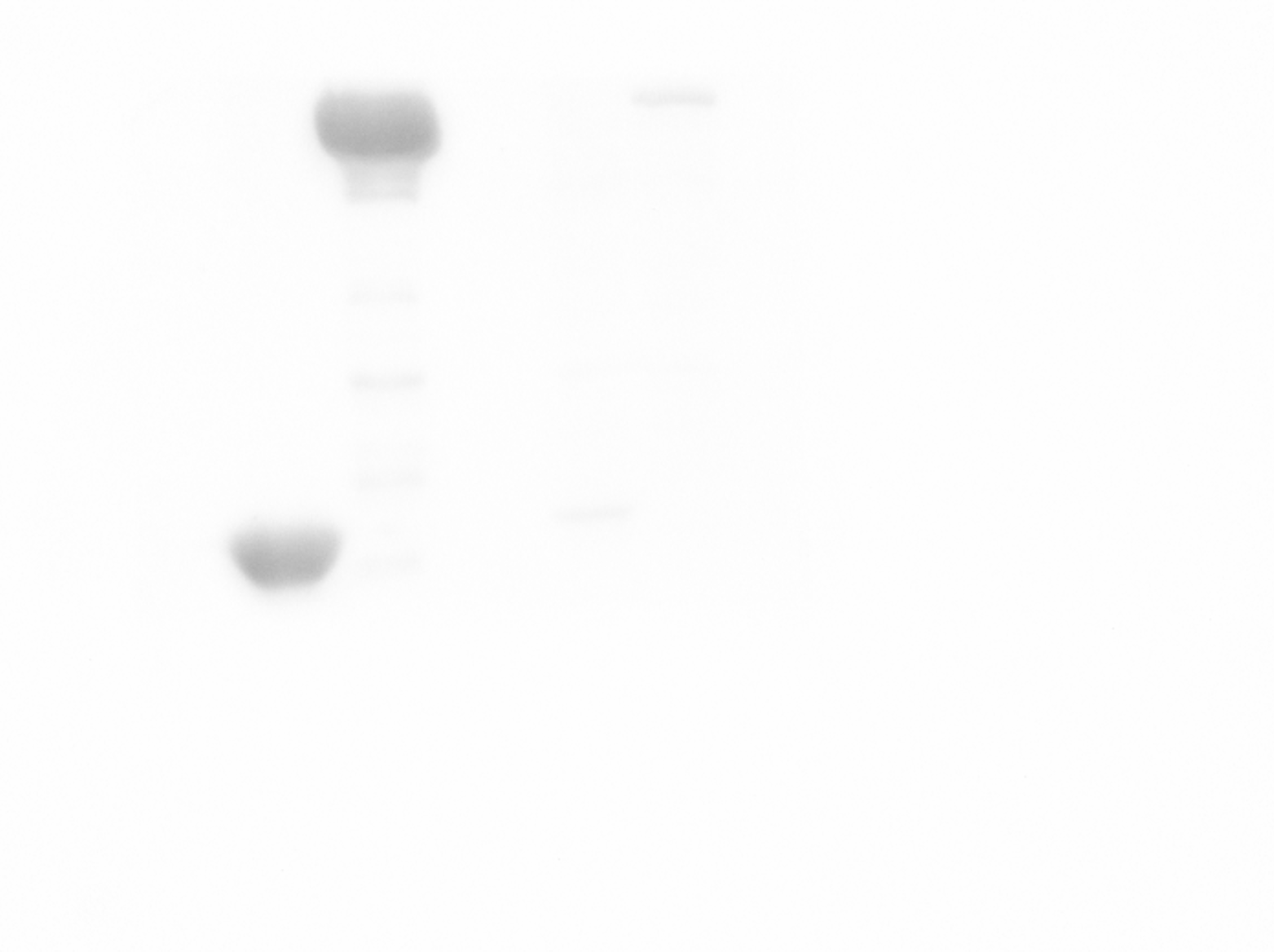

Supplement: Figure 2—figure supplement 1—source data 7. [file elife-73523-fig2-figsupp1-data7.zip › Raw blots/Pulldown_ anti-GST.tif]

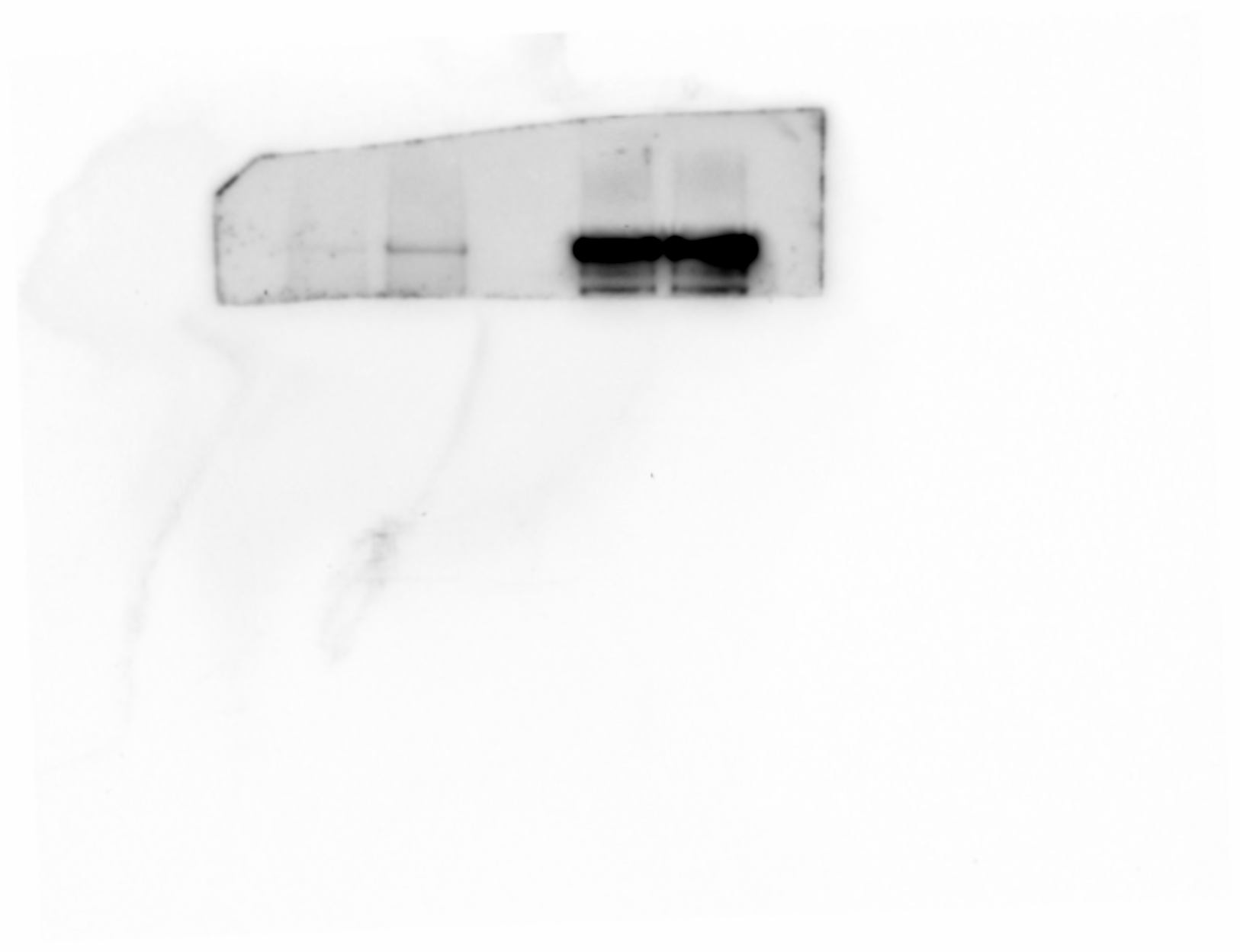

Supplement: Figure 2—figure supplement 1—source data 7. [file elife-73523-fig2-figsupp1-data7.zip › Raw blots/Pulldown_ anti-BAF250A.tif]

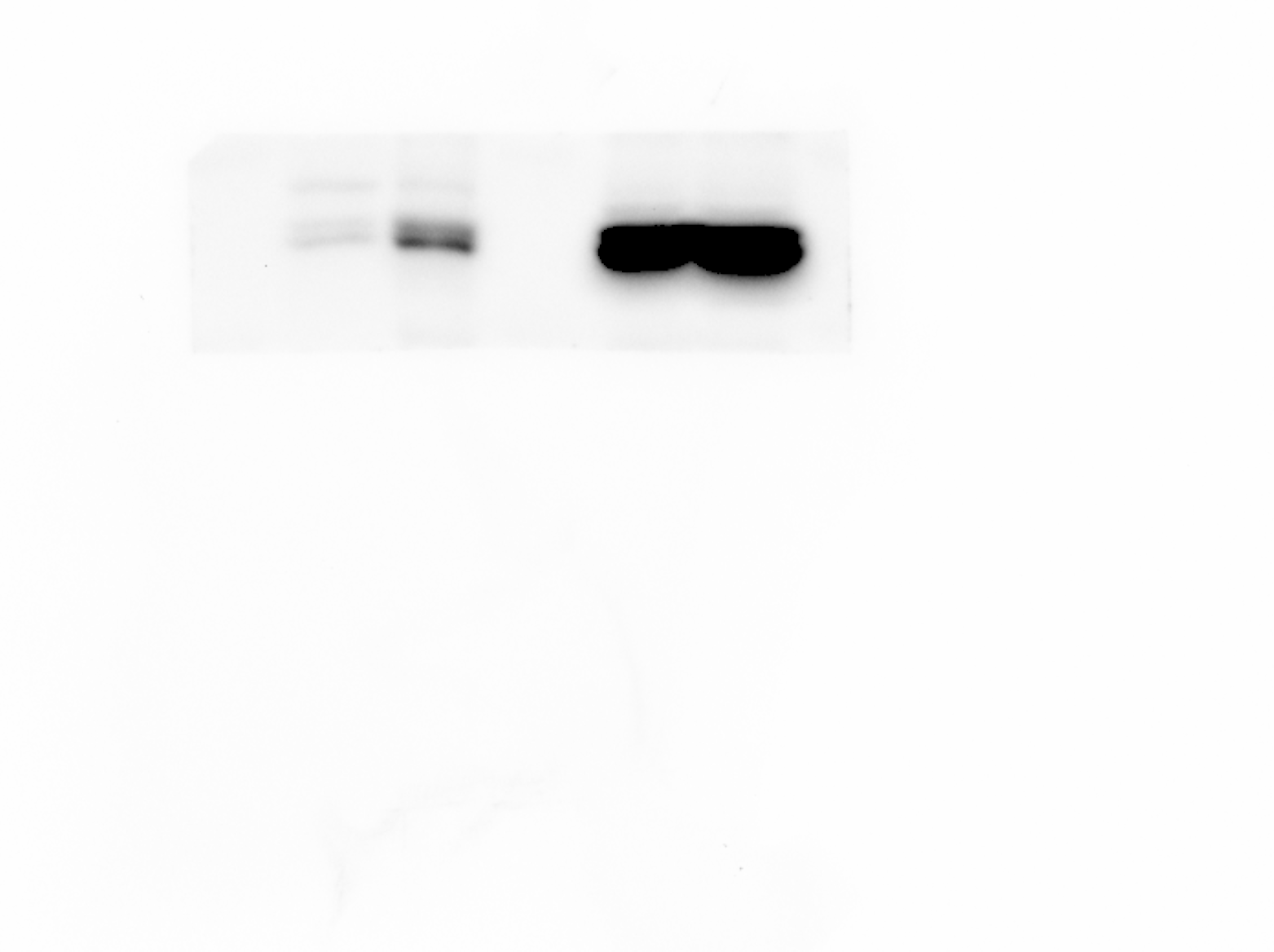

Supplement: Figure 2—figure supplement 1—source data 7. [file elife-73523-fig2-figsupp1-data7.zip › Raw blots/Pulldown_ anti-SMARCB1.tif]

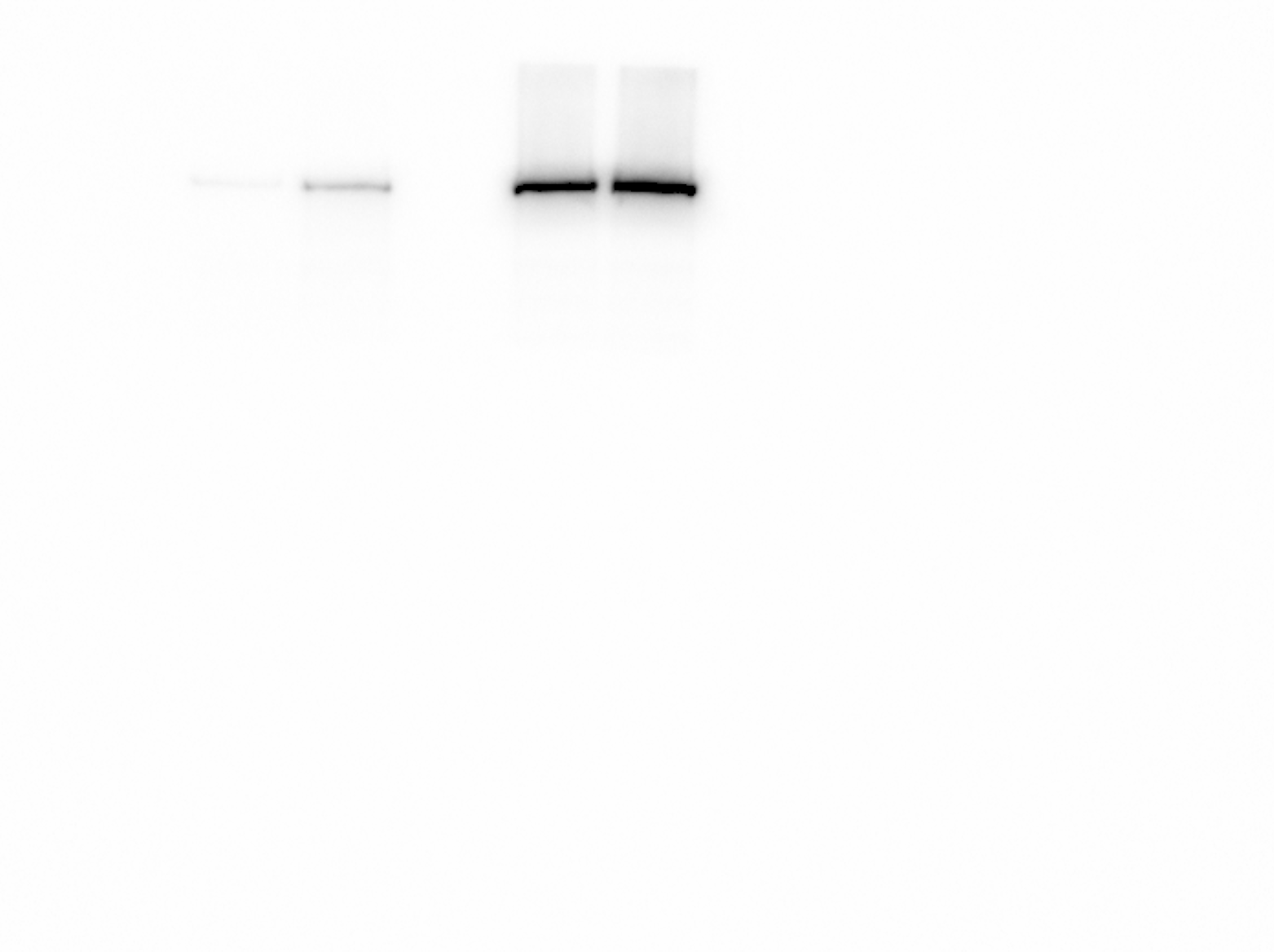

Supplement: Figure 2—figure supplement 1—source data 7. [file elife-73523-fig2-figsupp1-data7.zip › Raw blots/Pulldown_ anti-BRG1.tif]

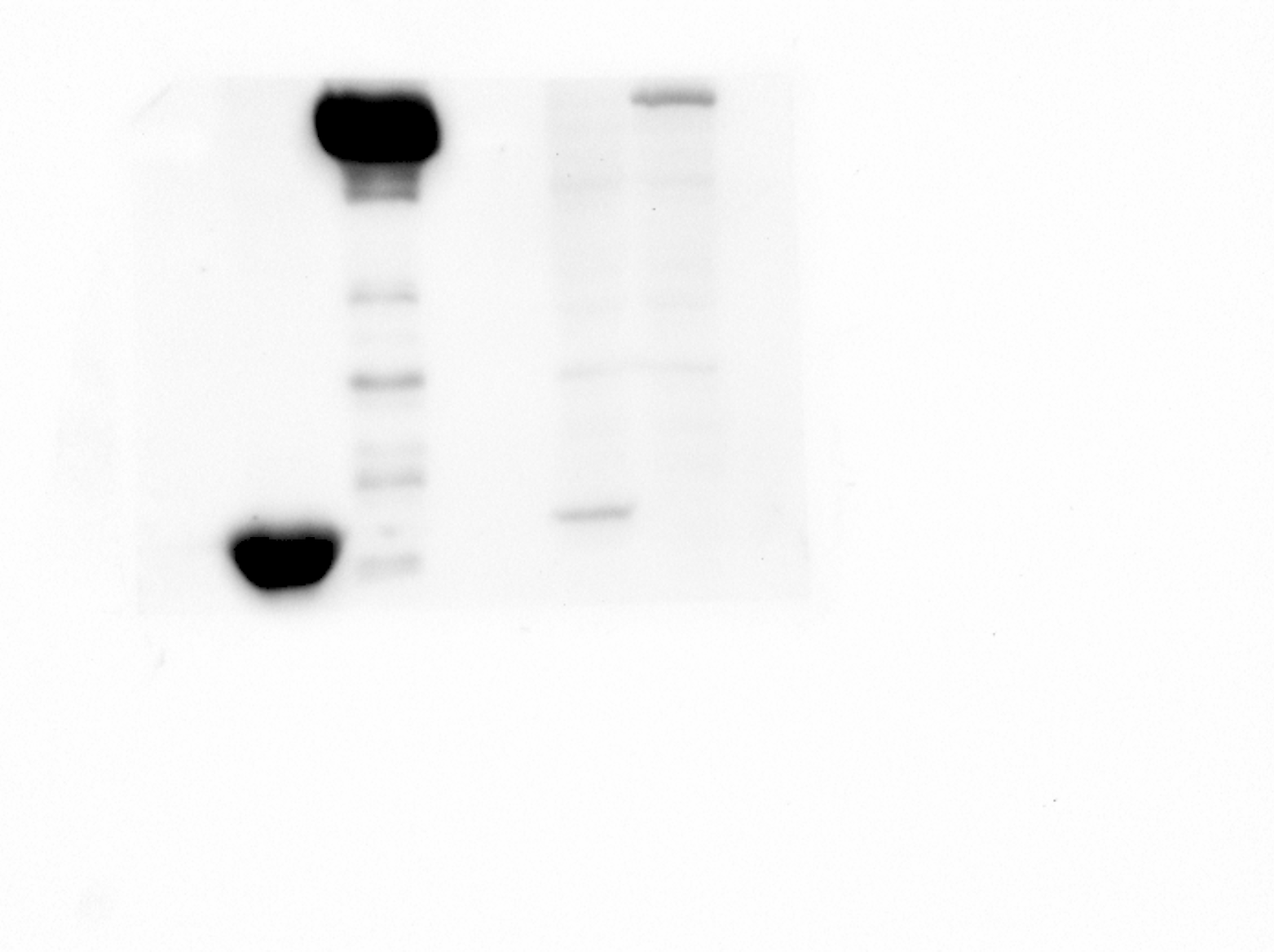

Supplement: Figure 2—figure supplement 1—source data 7. [file elife-73523-fig2-figsupp1-data7.zip › Raw blots/Input_ anti-GST.tif]

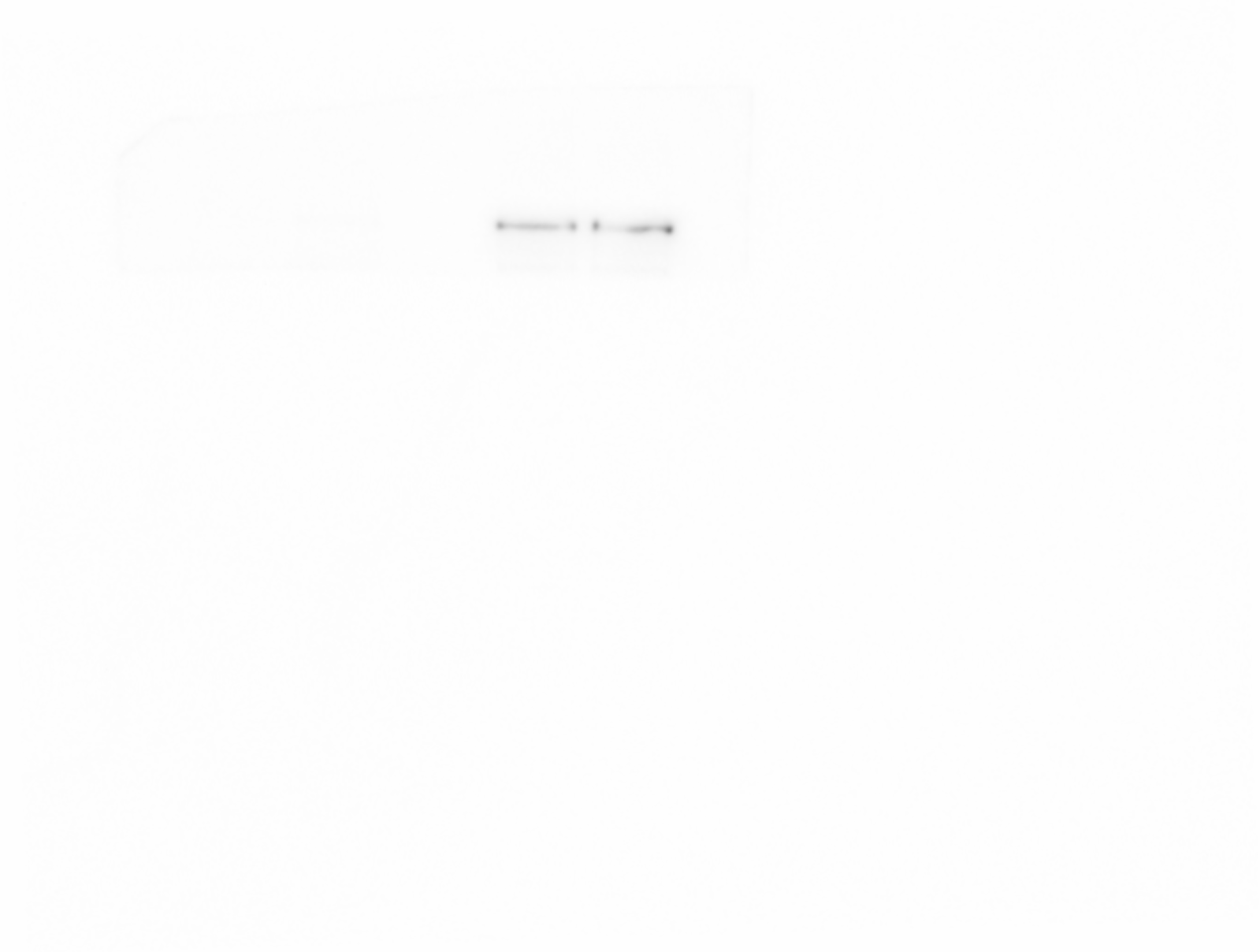

Supplement: Figure 2—figure supplement 1—source data 7. [file elife-73523-fig2-figsupp1-data7.zip › Raw blots/Input_ anti-BAF250A.tif]

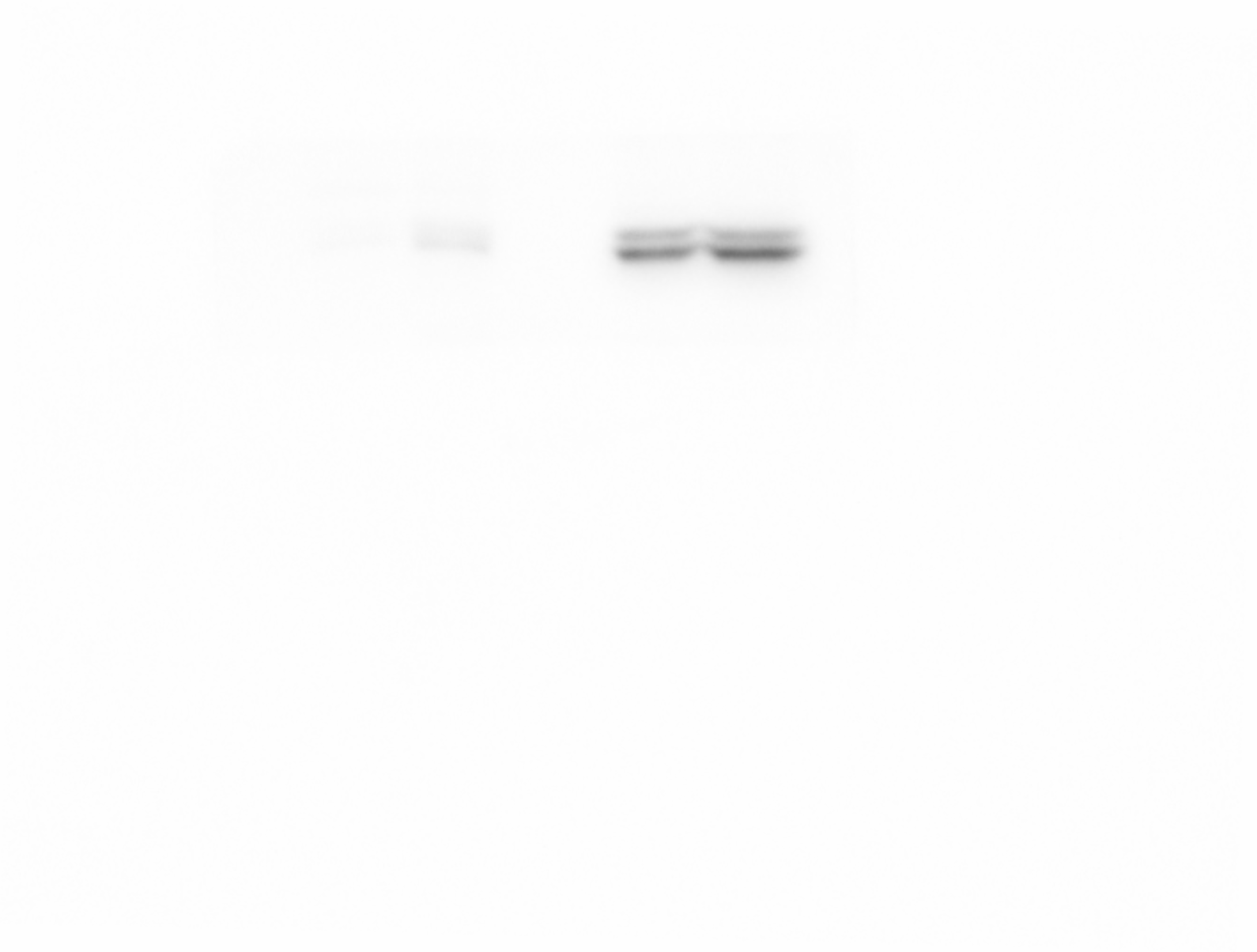

Supplement: Figure 2—figure supplement 1—source data 7. [file elife-73523-fig2-figsupp1-data7.zip › Raw blots/Input_ anti-SMARCB1.tif]

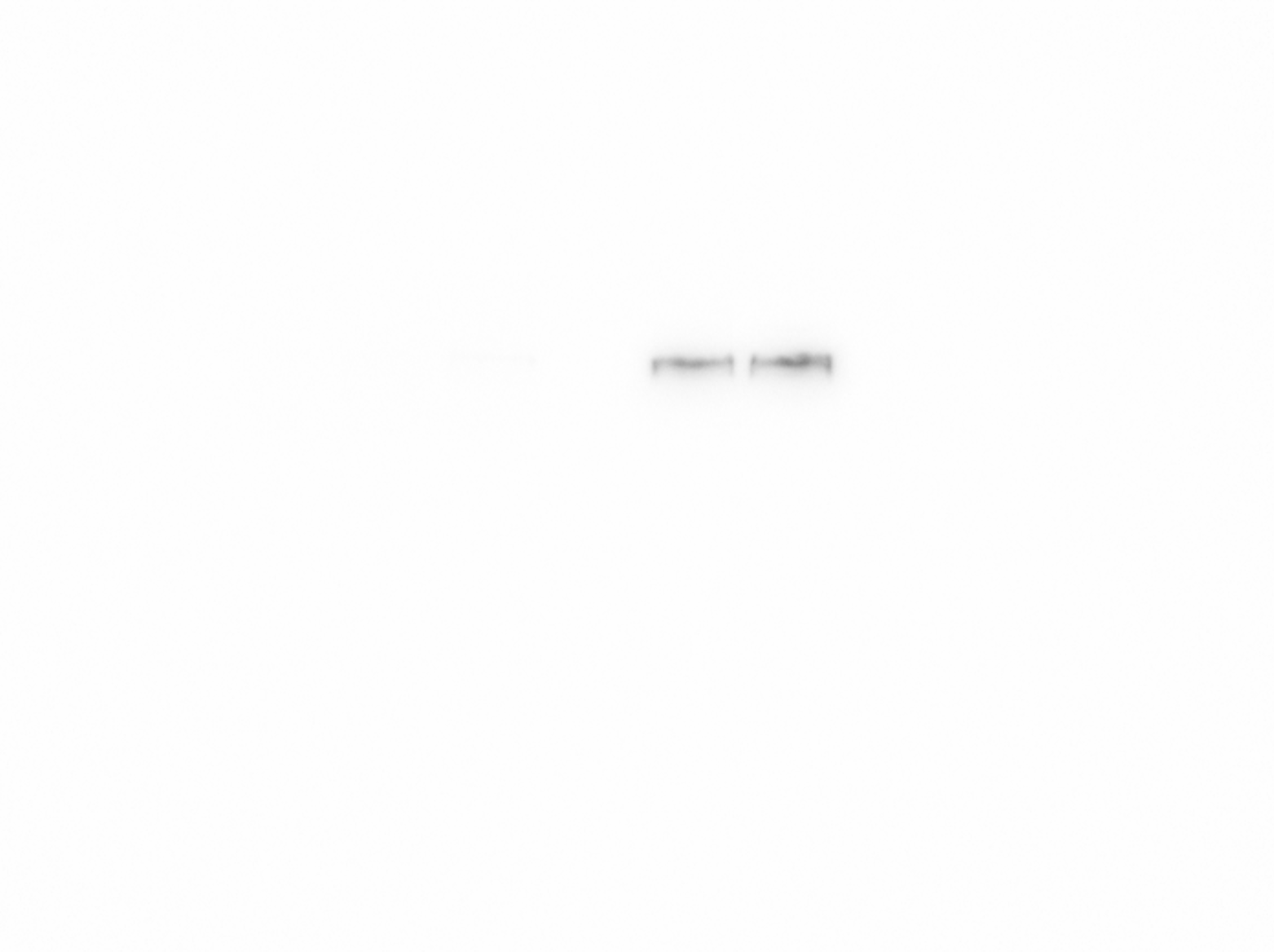

Supplement: Figure 2—figure supplement 1—source data 7. [file elife-73523-fig2-figsupp1-data7.zip › Raw blots/Input_ anti-BAF170.tif]

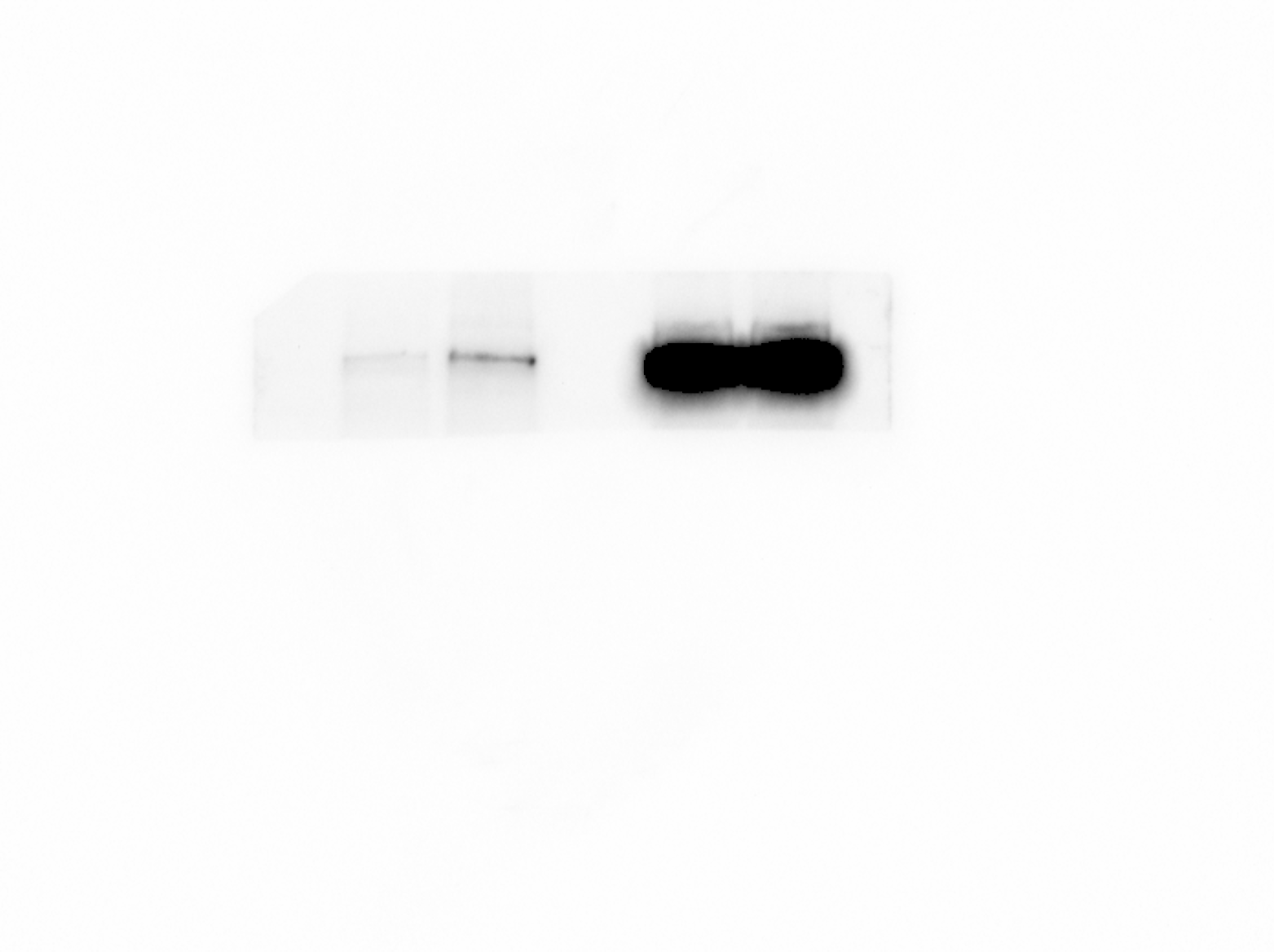

Supplement: Figure 2—figure supplement 1—source data 7. [file elife-73523-fig2-figsupp1-data7.zip › Raw blots/Pulldown_ anti-BAF170.tif]

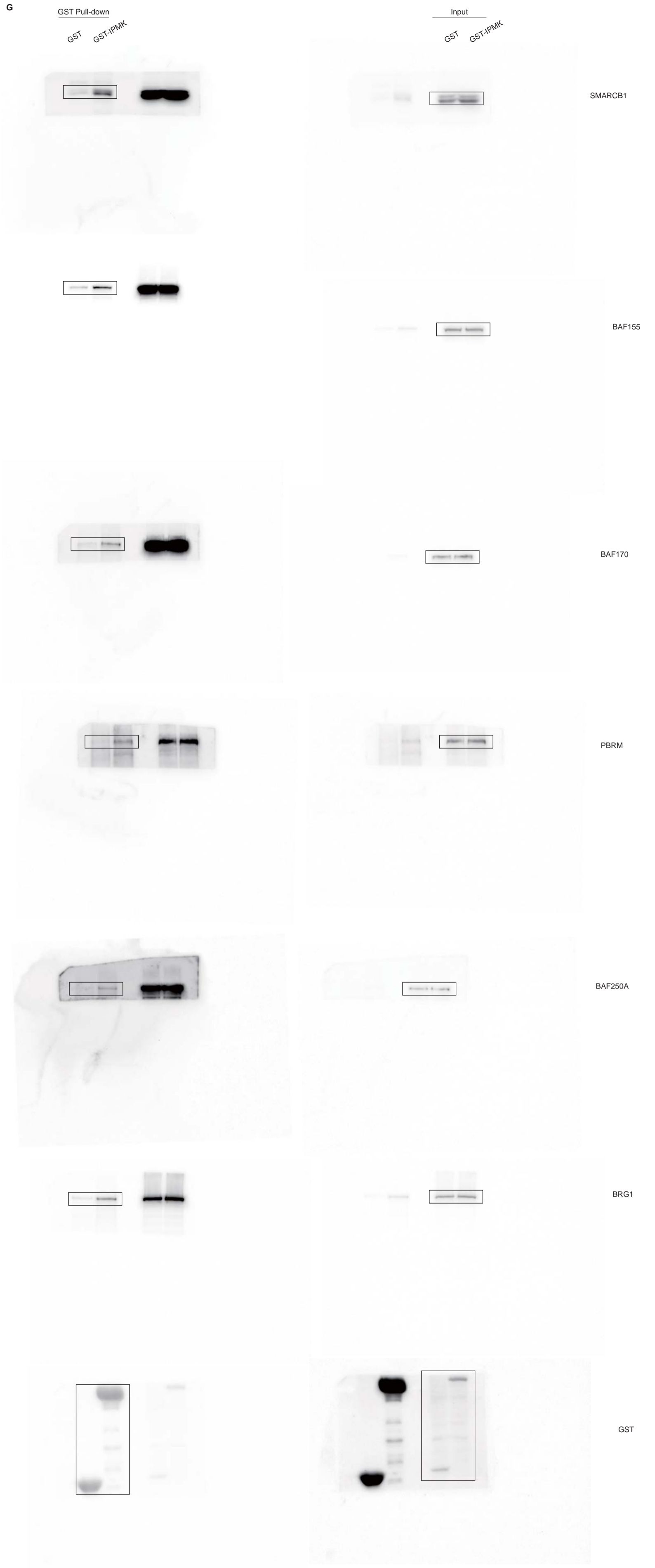

Supplement: Figure 2—figure supplement 1—source data 7. [file elife-73523-fig2-figsupp1-data7.zip › Labelled blots.pdf]

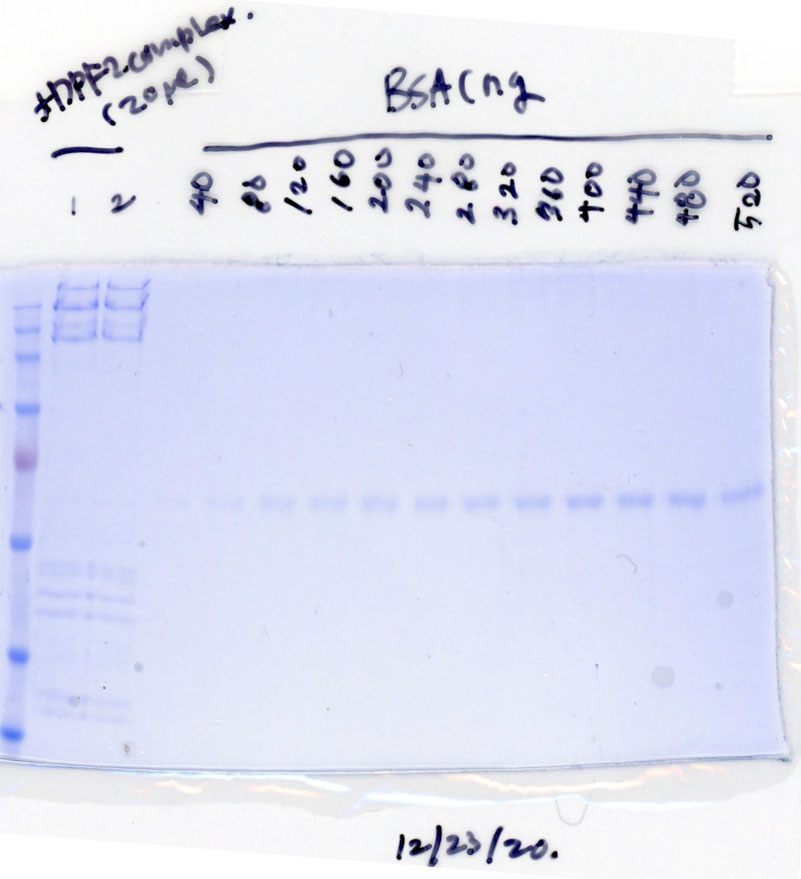

Supplement: Figure 2—figure supplement 1—source data 8. [file elife-73523-fig2-figsupp1-data8.zip › Raw blots/SWI:SNF complex with BSA.jpg]

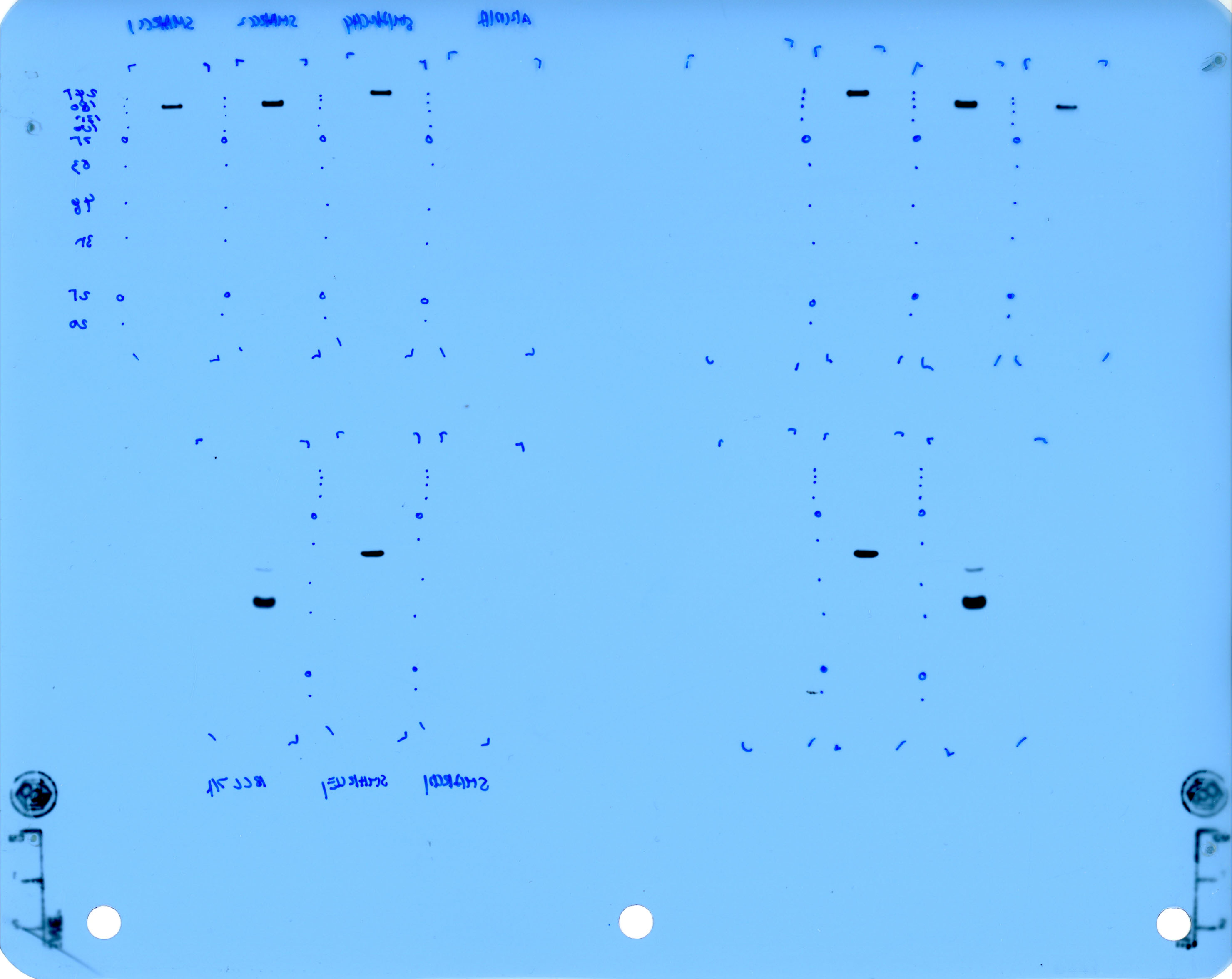

Supplement: Figure 2—figure supplement 1—source data 8. [file elife-73523-fig2-figsupp1-data8.zip › Raw blots/Anti-SMARCA4_SMARCC1_SMARCC2.jpg]

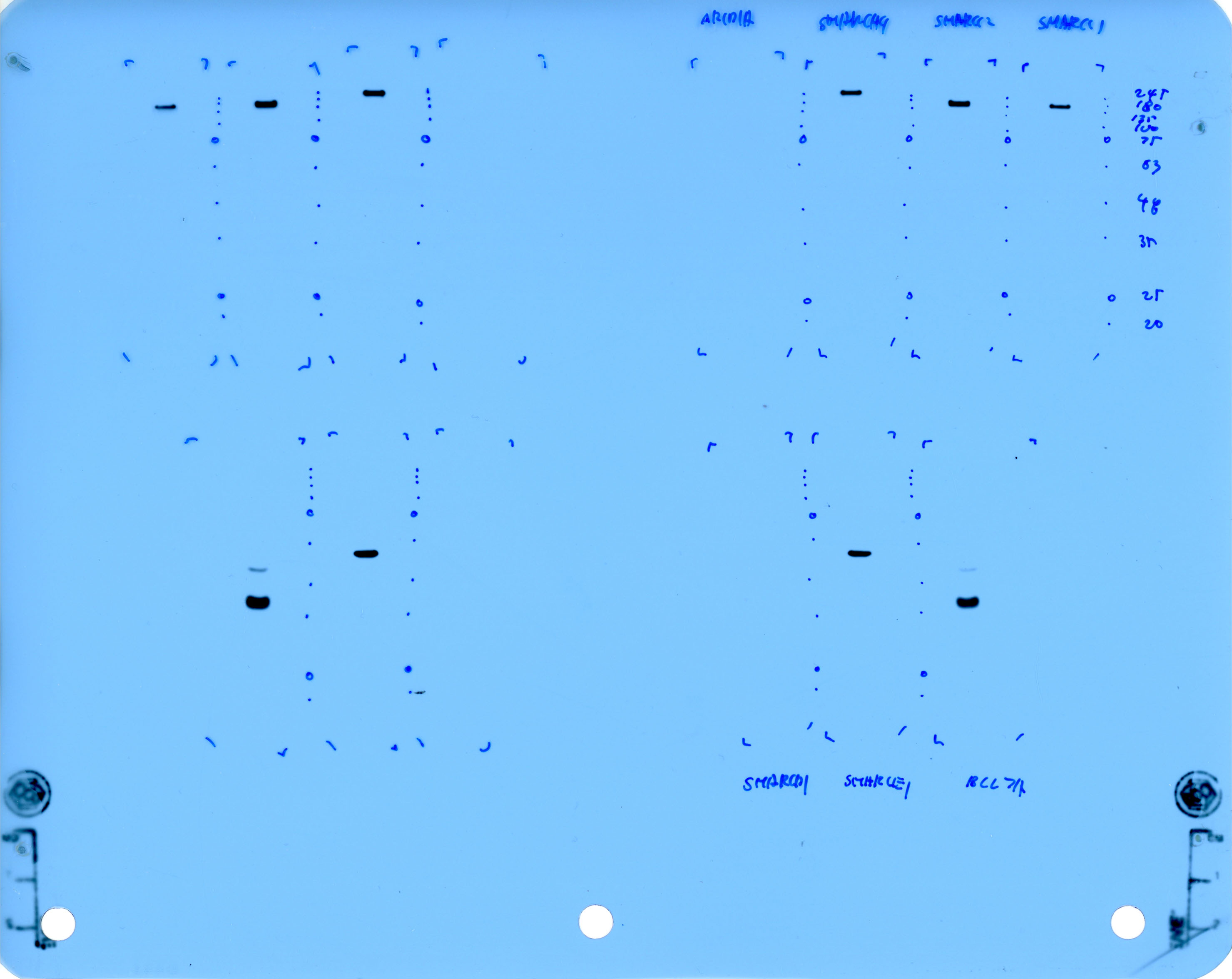

Supplement: Figure 2—figure supplement 1—source data 8. [file elife-73523-fig2-figsupp1-data8.zip › Raw blots/Anti-SMARCE1_BCL7A.jpg]

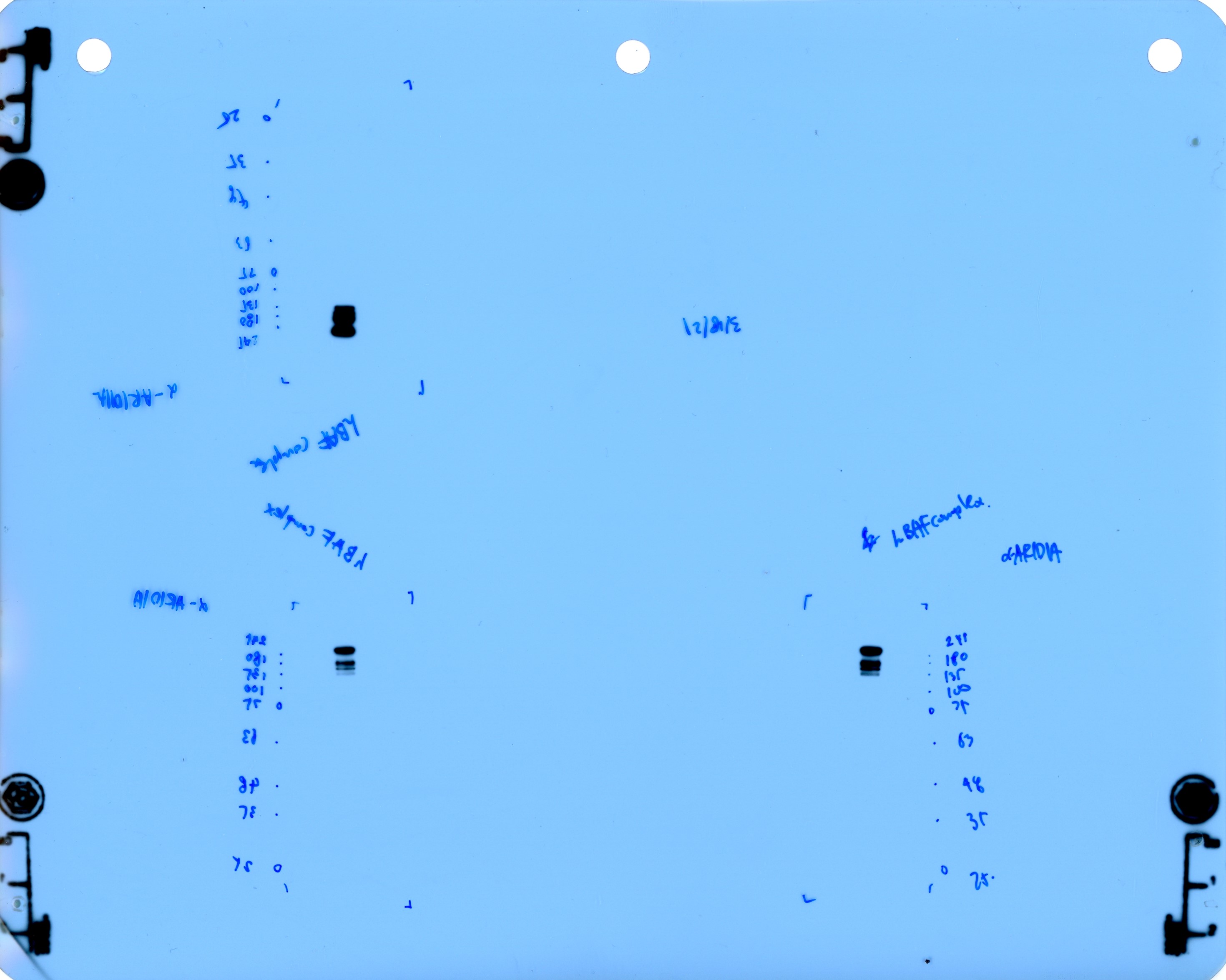

Supplement: Figure 2—figure supplement 1—source data 8. [file elife-73523-fig2-figsupp1-data8.zip › Raw blots/Anti-ARID1A.jpg]

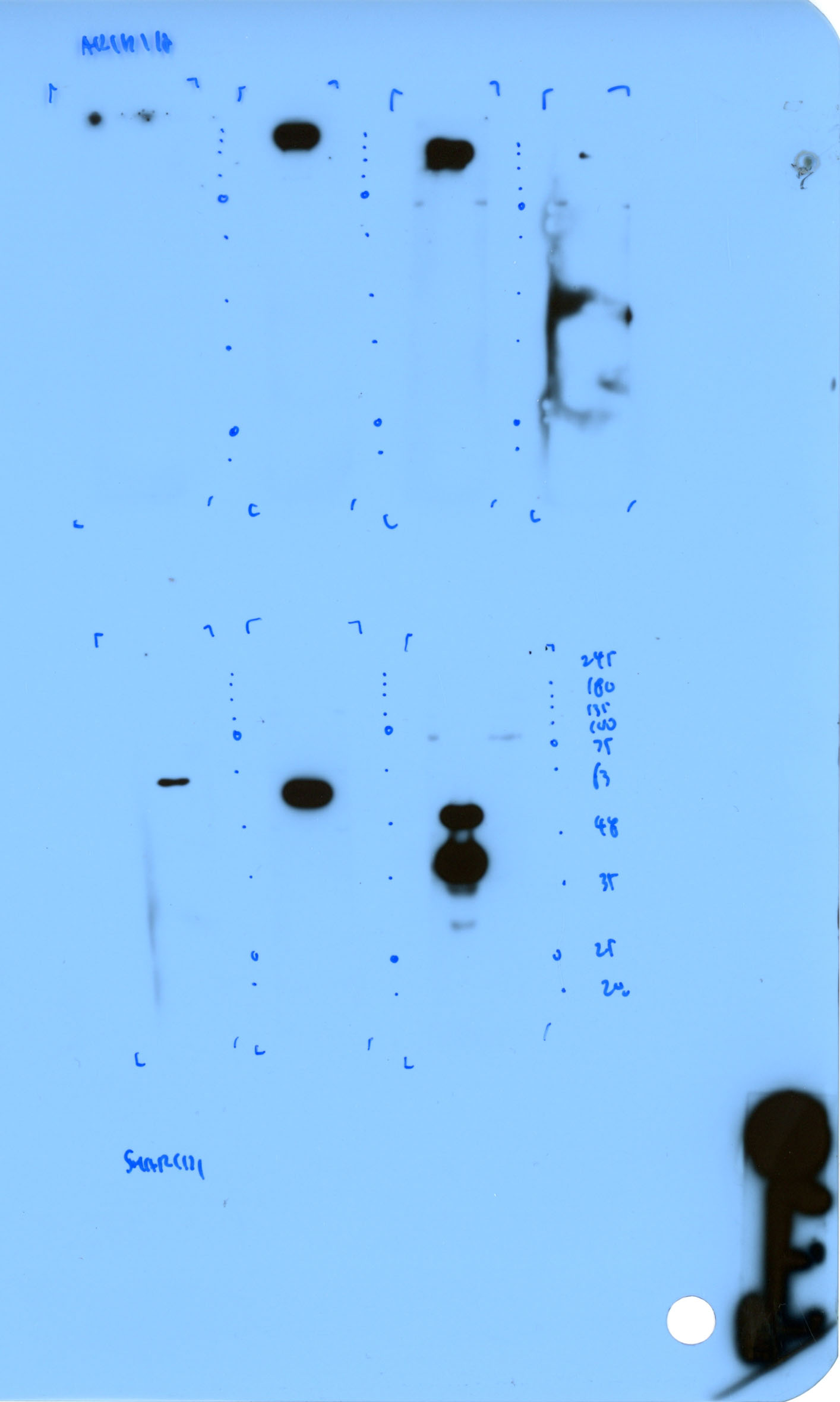

Supplement: Figure 2—figure supplement 1—source data 8. [file elife-73523-fig2-figsupp1-data8.zip › Raw blots/Anti-SMARCD1.jpg]

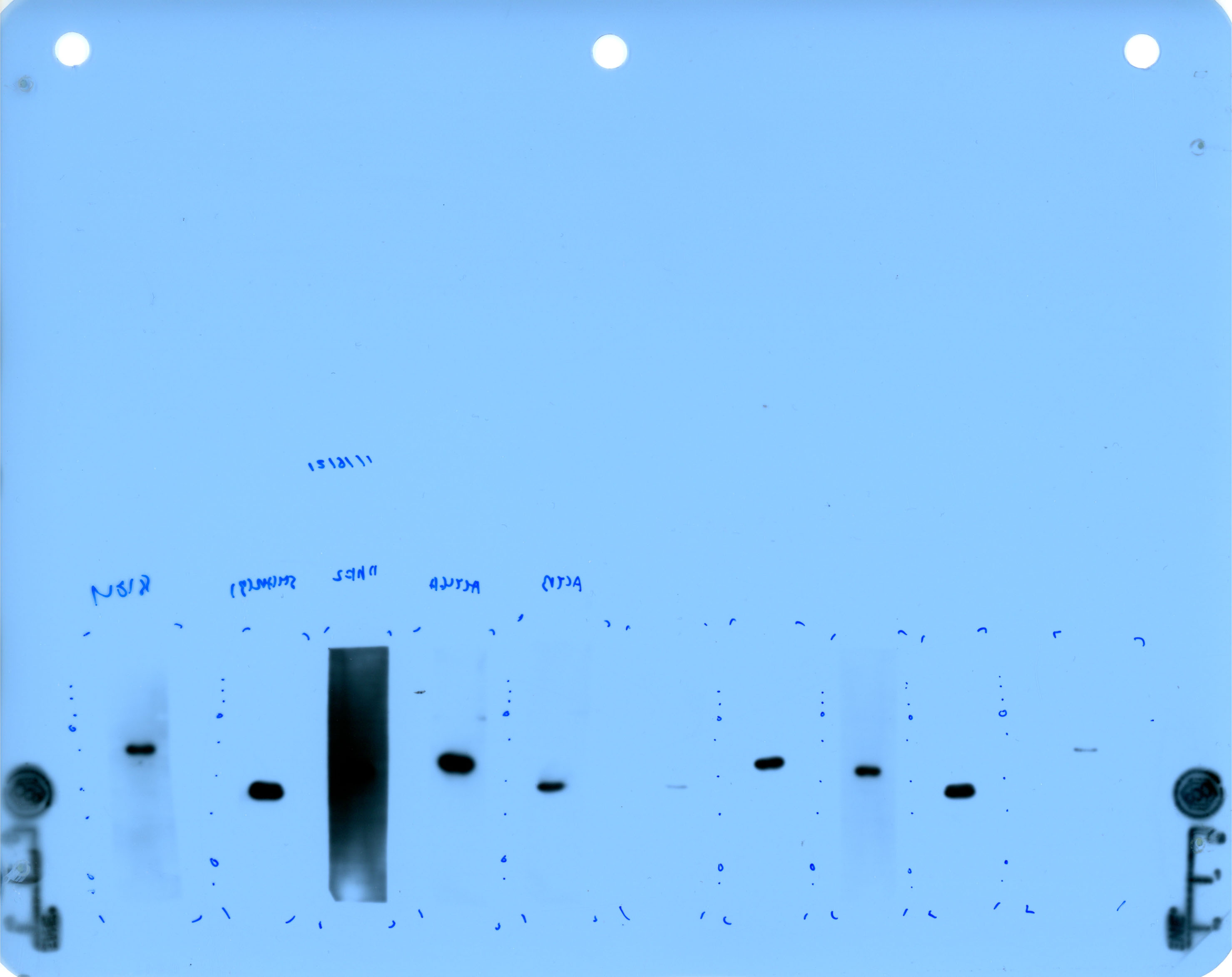

Supplement: Figure 2—figure supplement 1—source data 8. [file elife-73523-fig2-figsupp1-data8.zip › Raw blots/Anti-ACTL6A_DPF2.jpg]
