# Supplementary figures and images for: Inositol polyphosphate multikinase physically binds to the SWI/SNF complex and modulates BRG1 occupancy in mouse embryonic stem cells (part 2 of 2)
Source: eLife. 2022 May 12;11:e73523. doi: 10.7554/eLife.73523 (PMC9098221; doi:10.7554/eLife.73523)

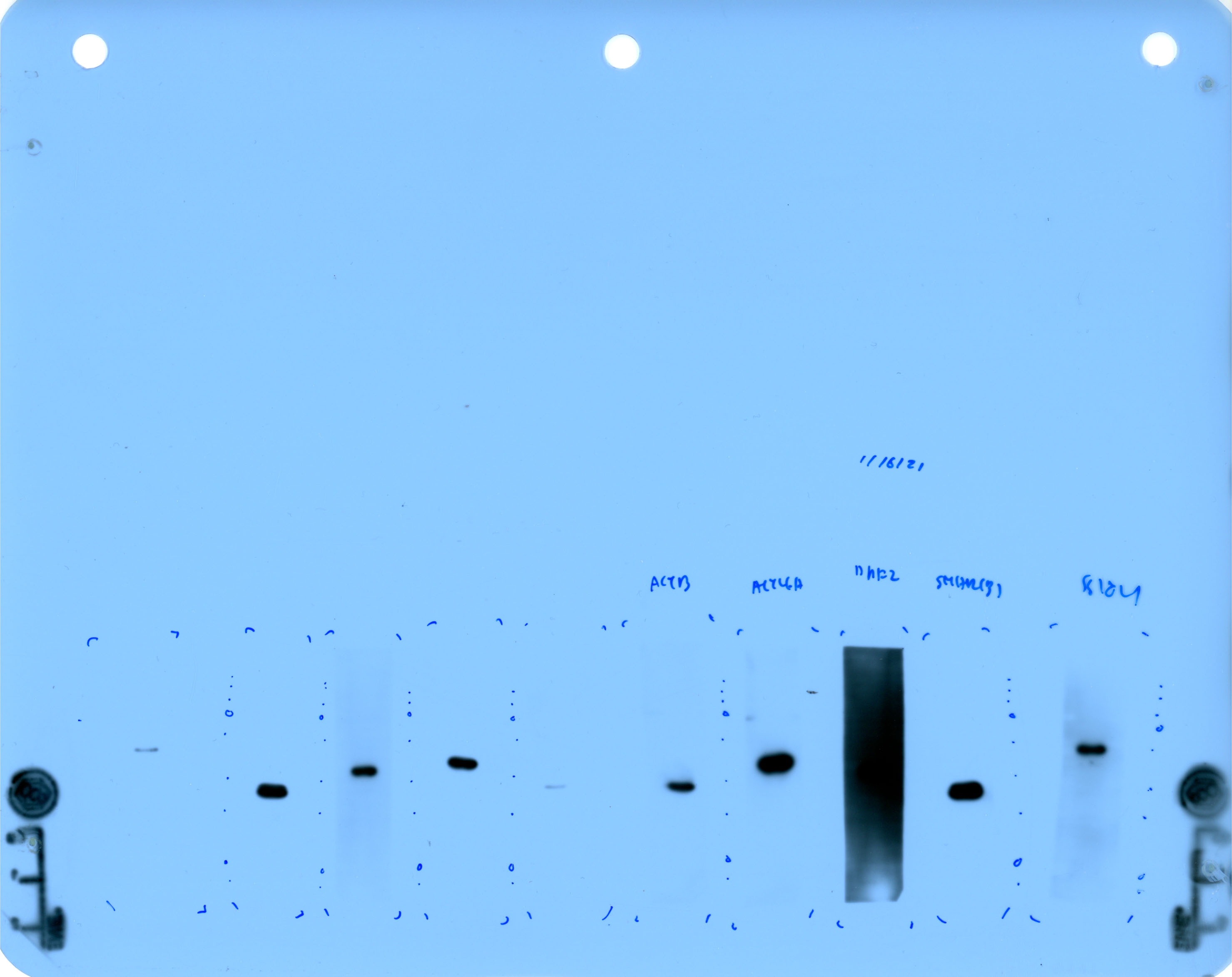

Supplement: Figure 2—figure supplement 1—source data 8. [file elife-73523-fig2-figsupp1-data8.zip › Raw blots/Anti-ACTB_SS18L1.jpg]

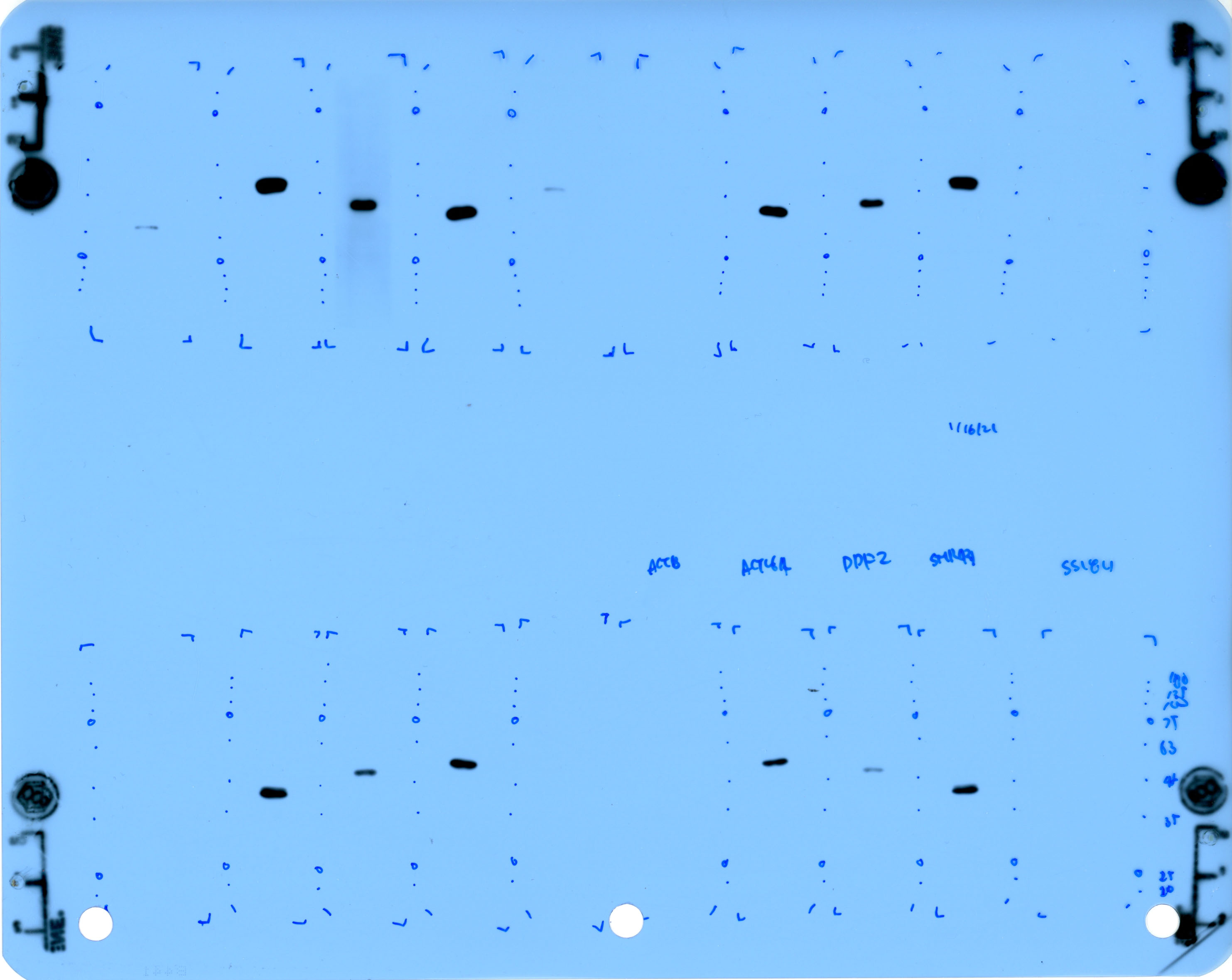

Supplement: Figure 2—figure supplement 1—source data 8. [file elife-73523-fig2-figsupp1-data8.zip › Raw blots/Anti-SMARCB1.jpg]

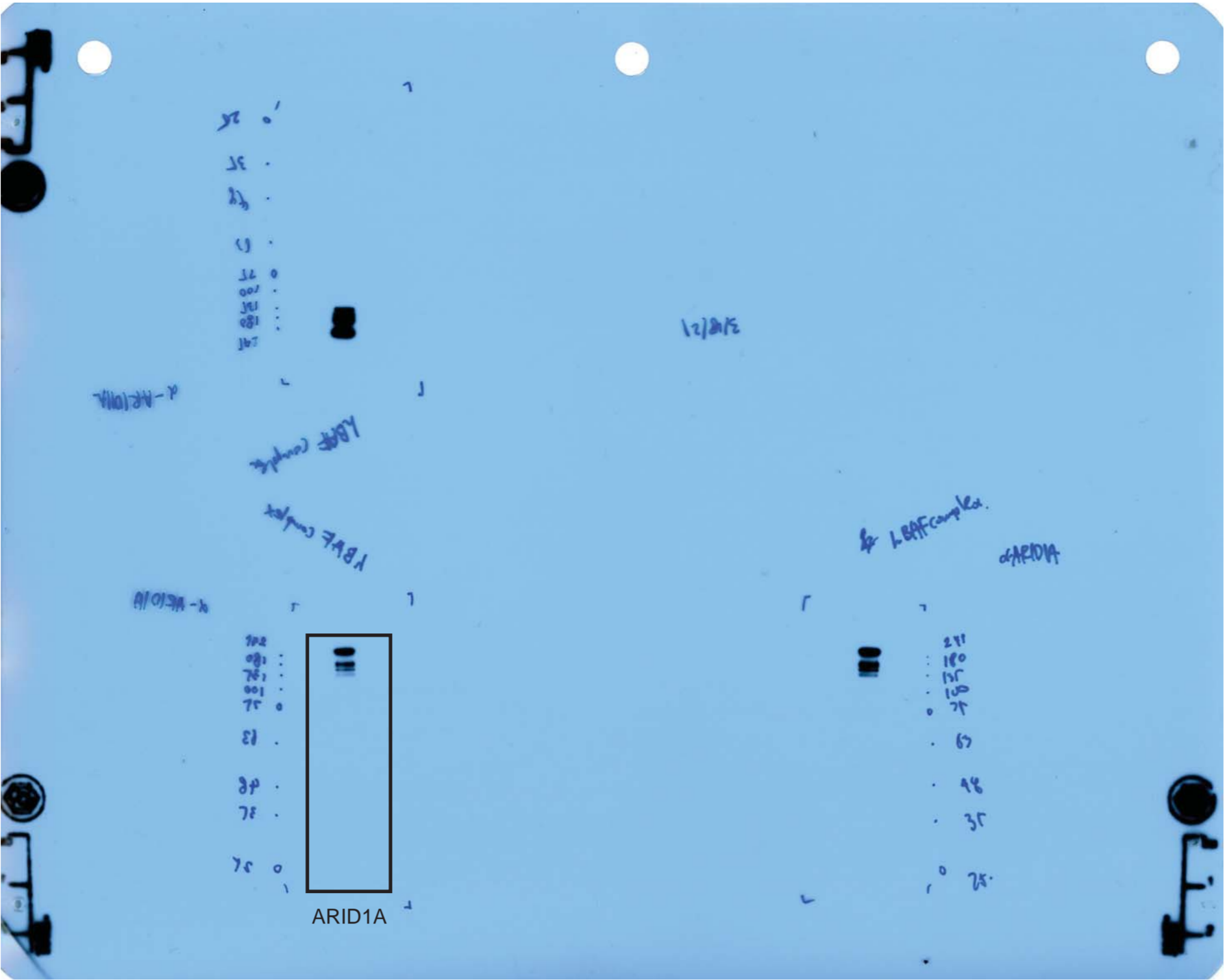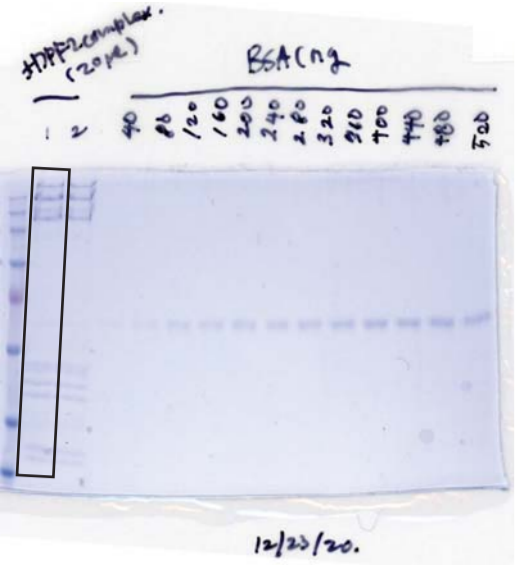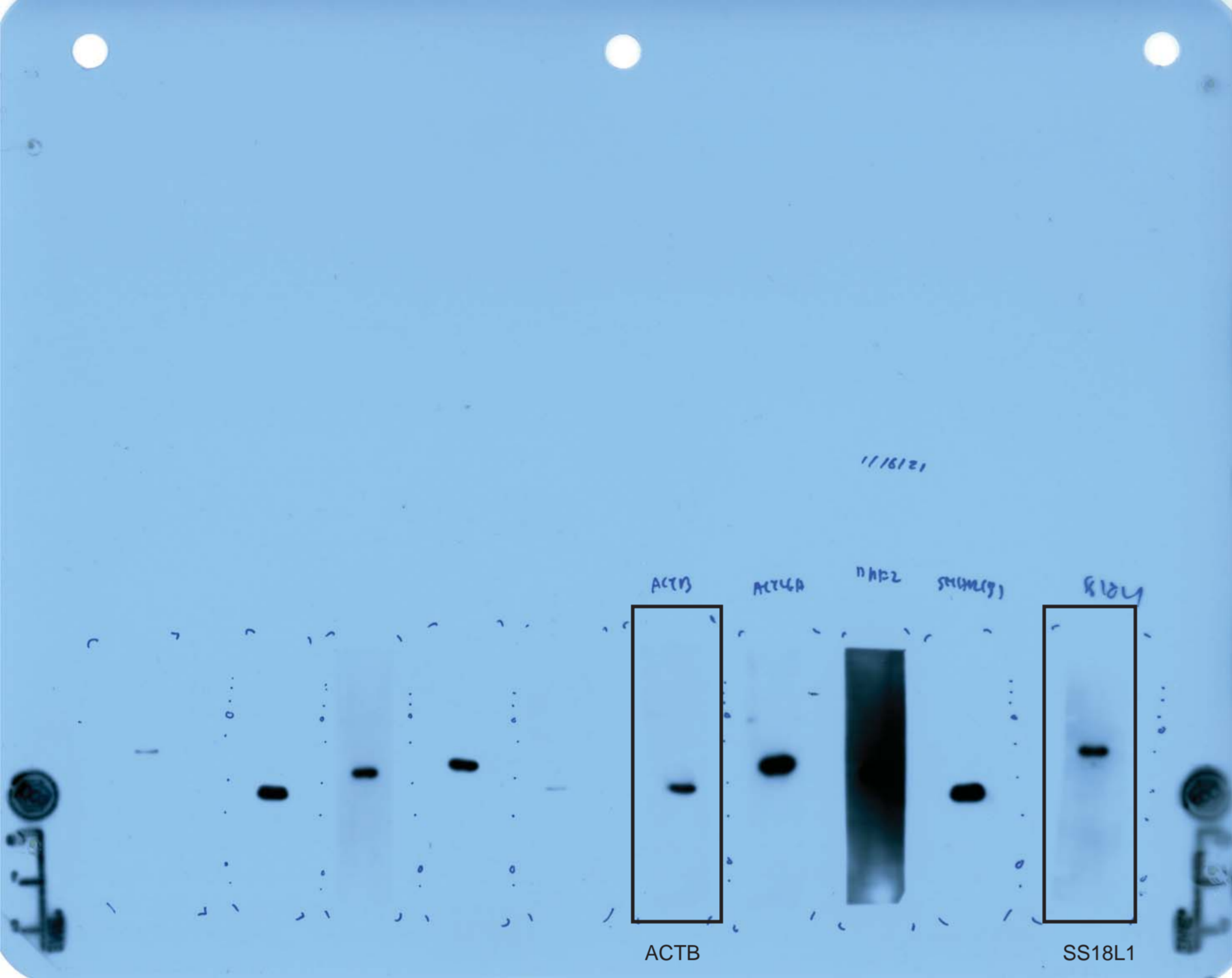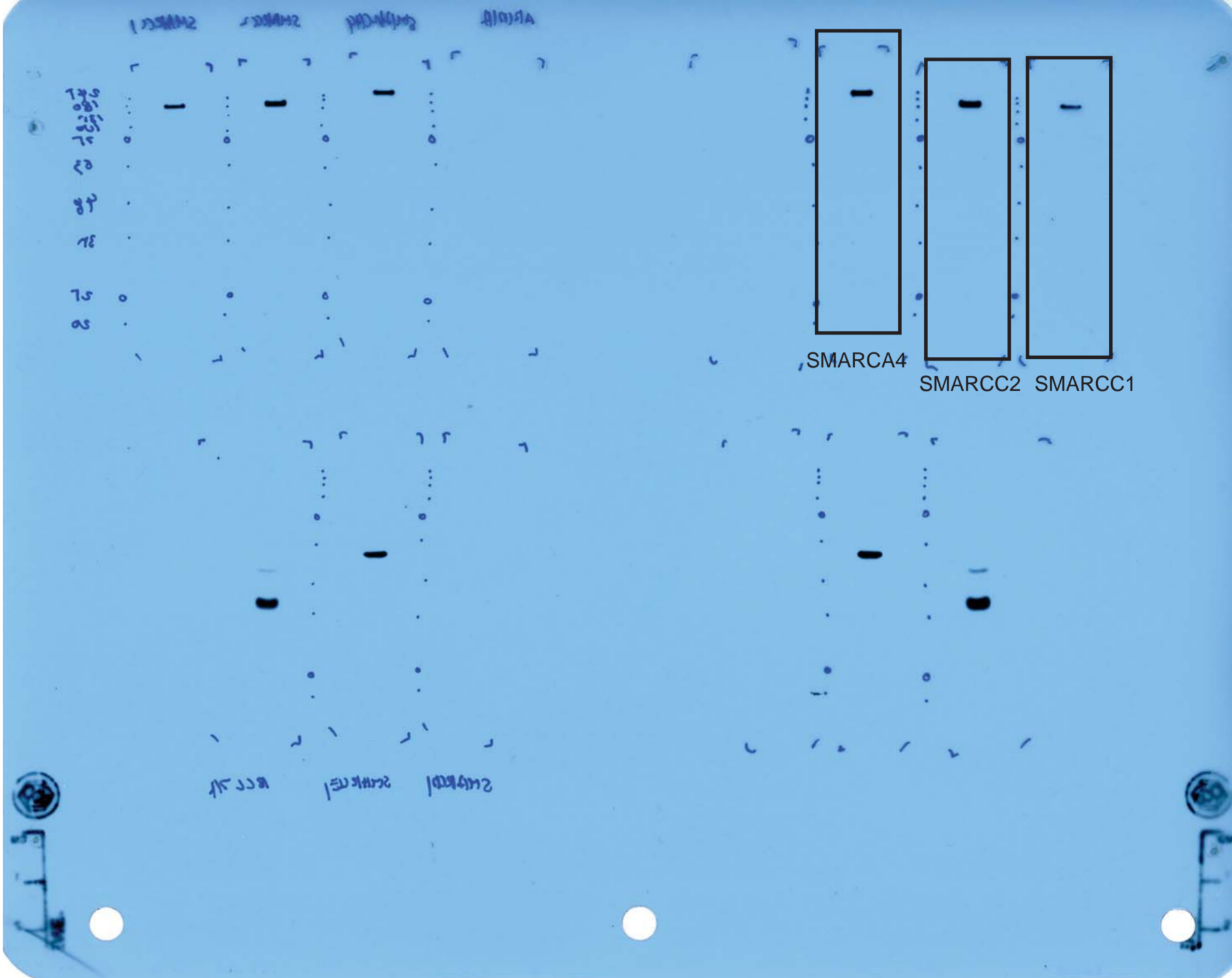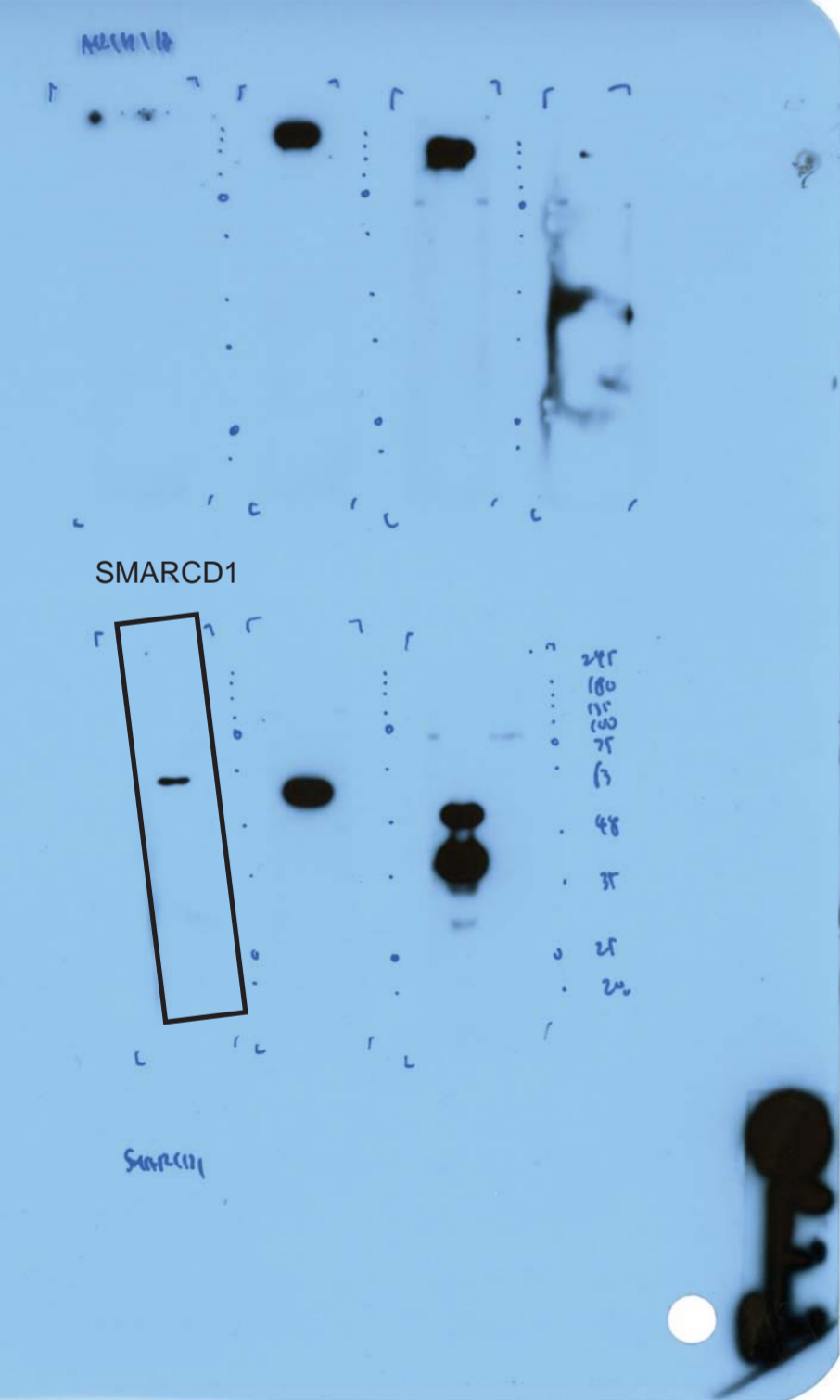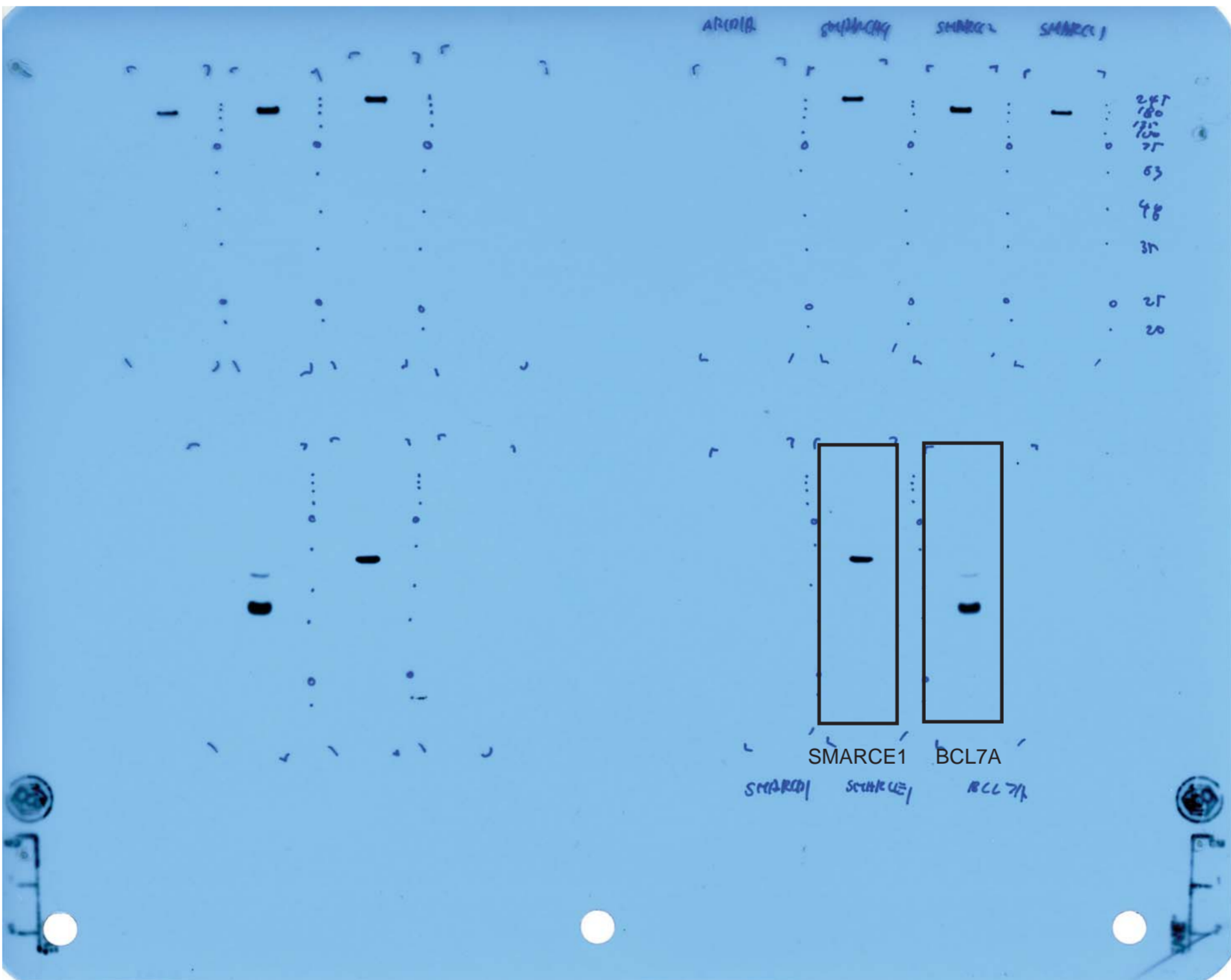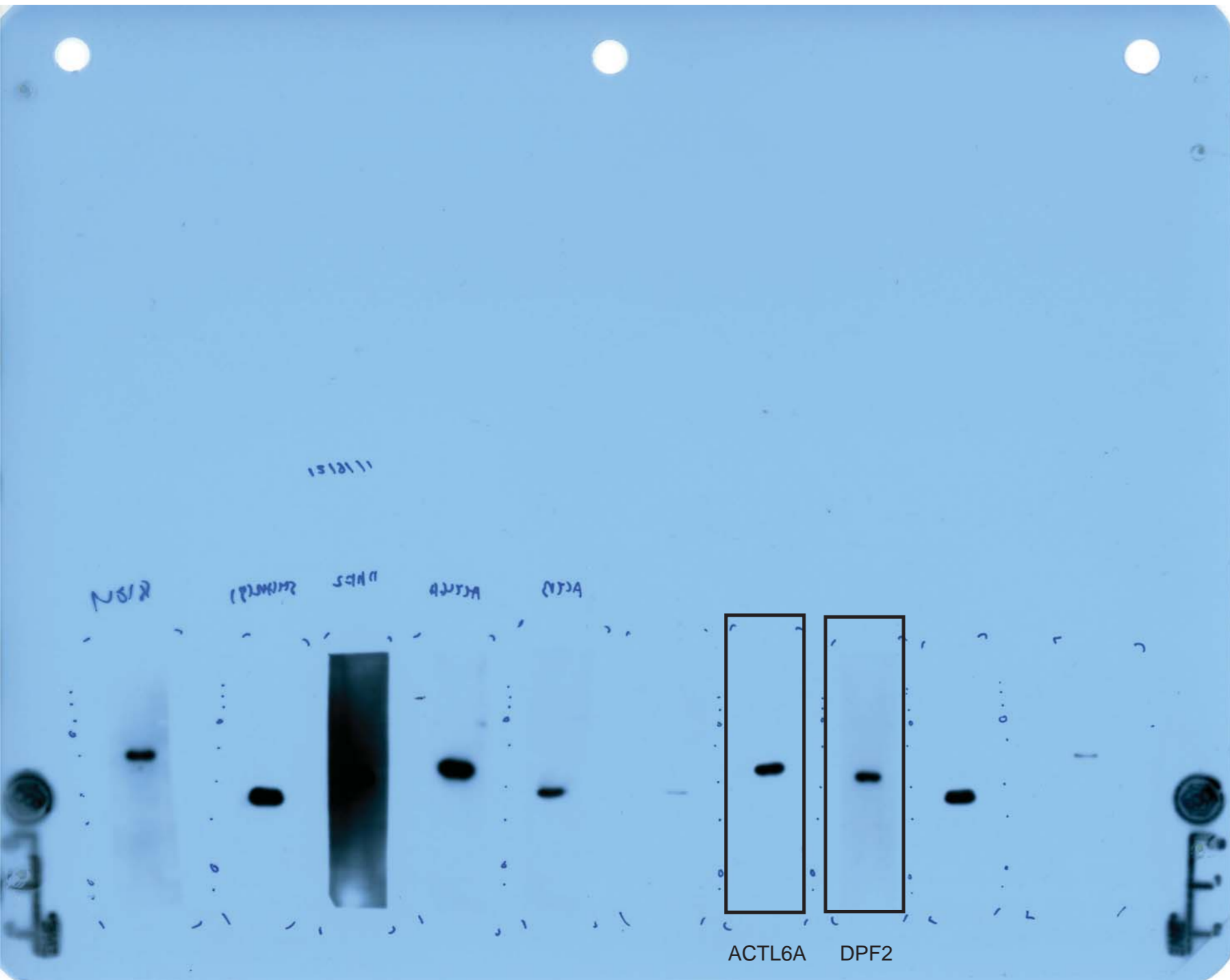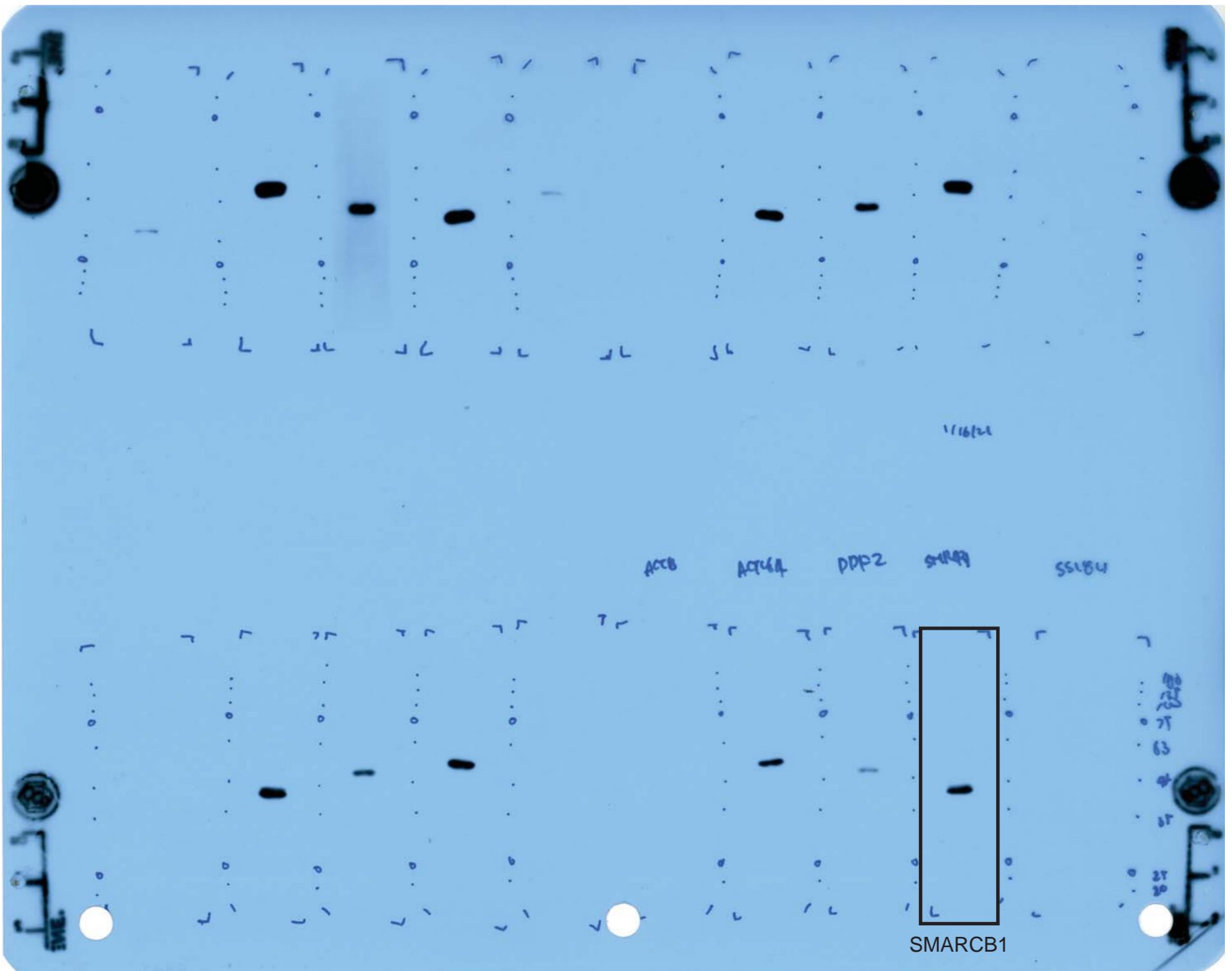

Supplement: Figure 2—figure supplement 1—source data 8. [file elife-73523-fig2-figsupp1-data8.zip › Labelled blots.pdf]

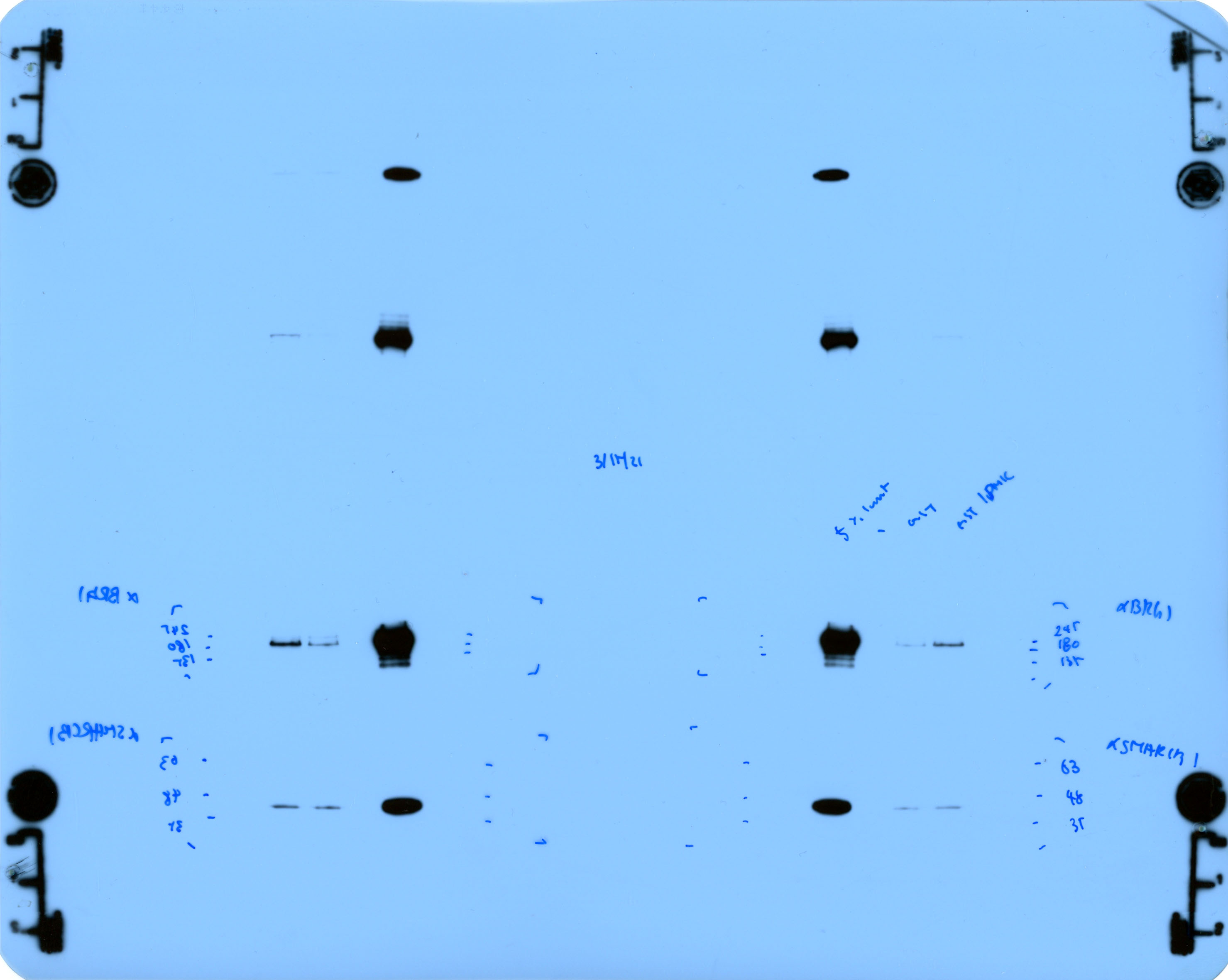

Supplement: Figure 2—figure supplement 1—source data 9. [file elife-73523-fig2-figsupp1-data9.zip › Raw blots and gels/Anti-BRG1.jpg]

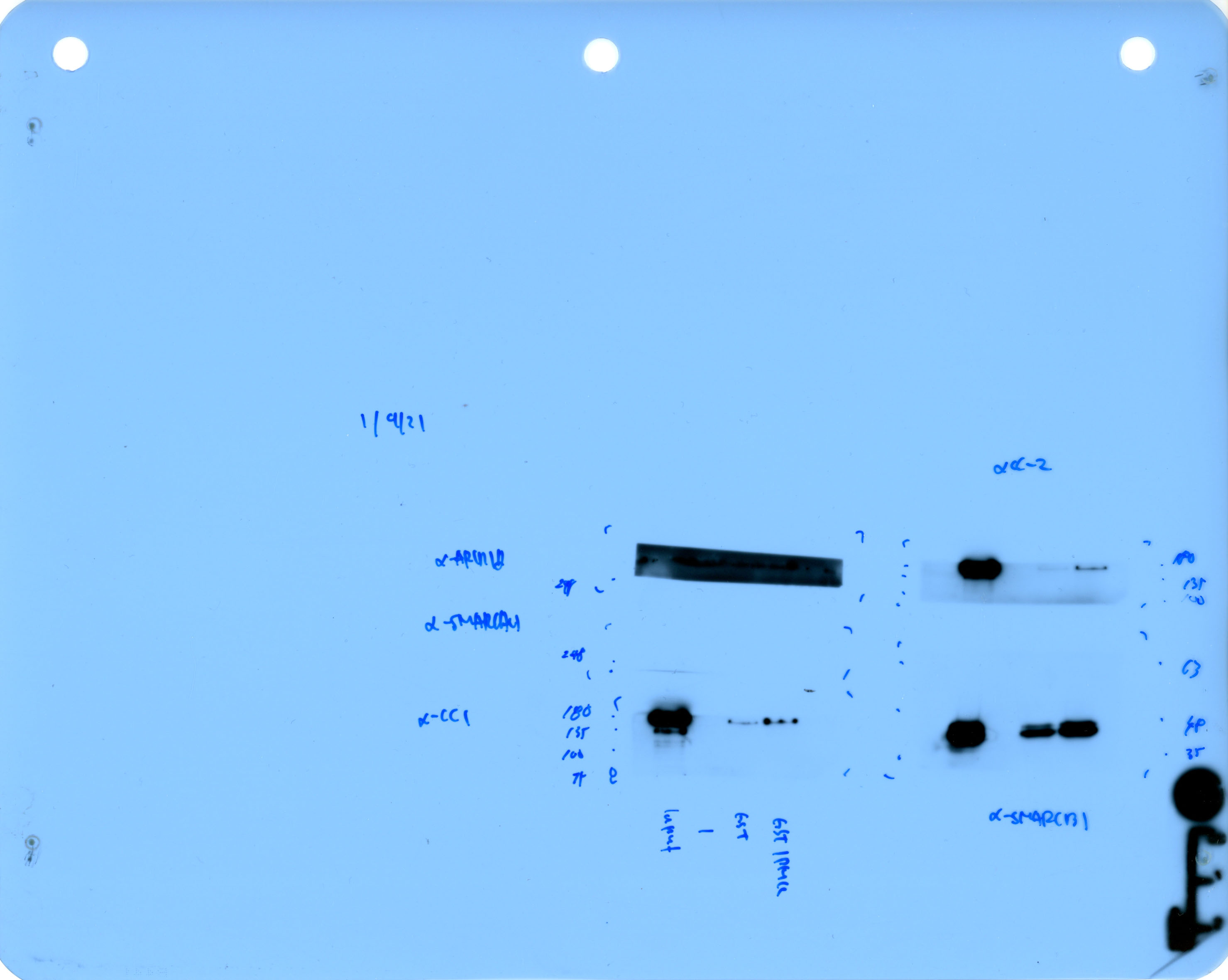

Supplement: Figure 2—figure supplement 1—source data 9. [file elife-73523-fig2-figsupp1-data9.zip › Raw blots and gels/Anti-BAF155_BAF170.jpg]

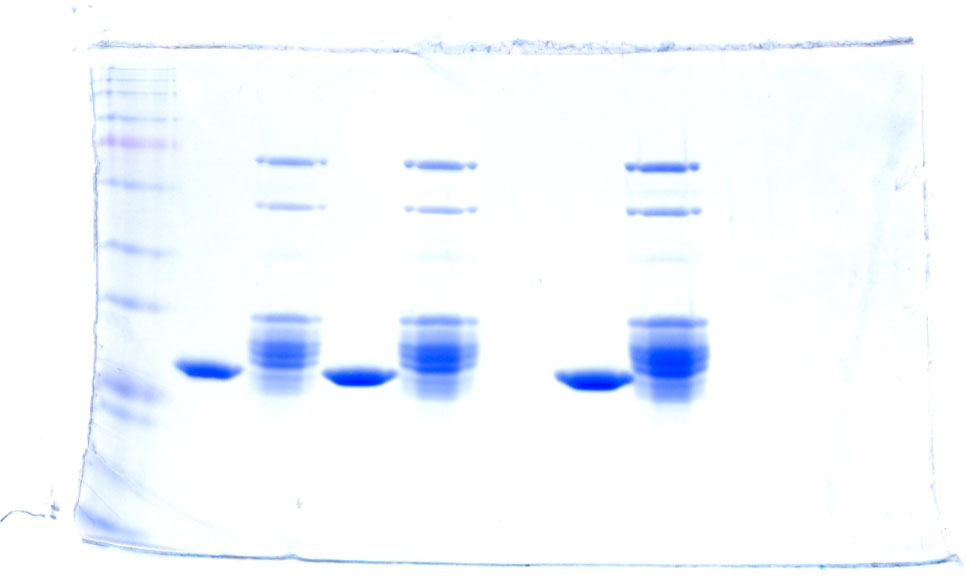

Supplement: Figure 2—figure supplement 1—source data 9. [file elife-73523-fig2-figsupp1-data9.zip › Raw blots and gels/GST and GST-IPMK.jpg]

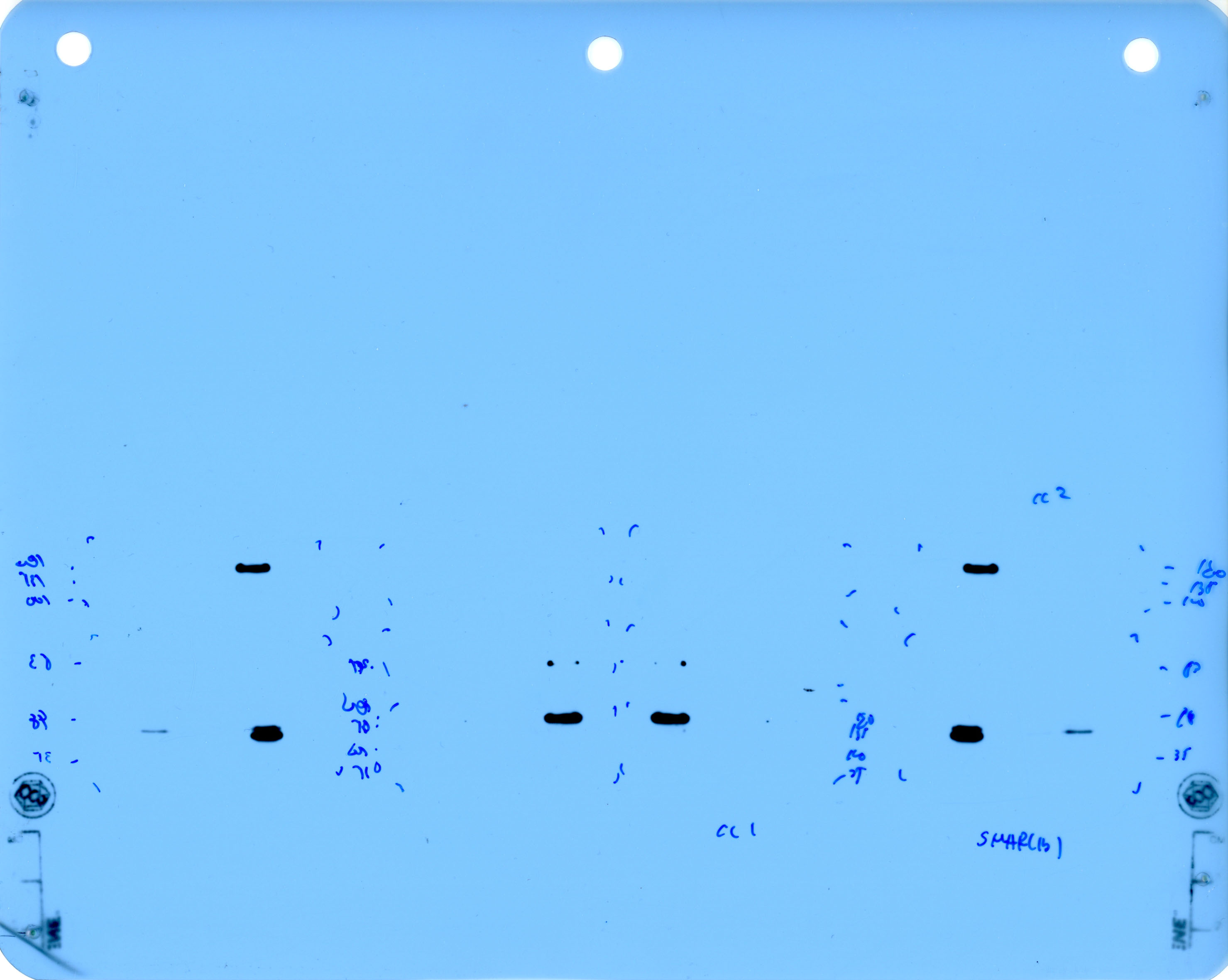

Supplement: Figure 2—figure supplement 1—source data 9. [file elife-73523-fig2-figsupp1-data9.zip › Raw blots and gels/Anti-SMARCB1.jpg]

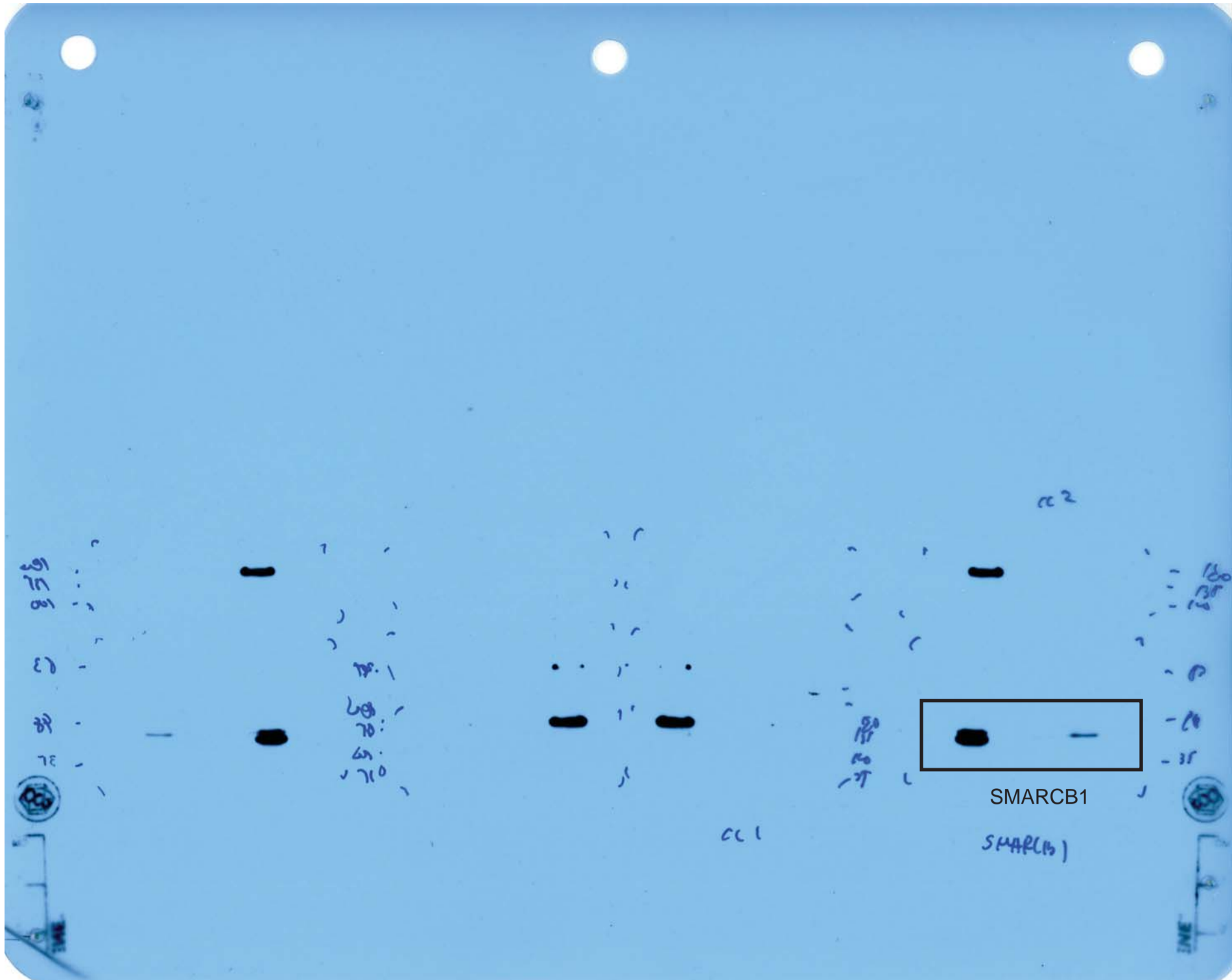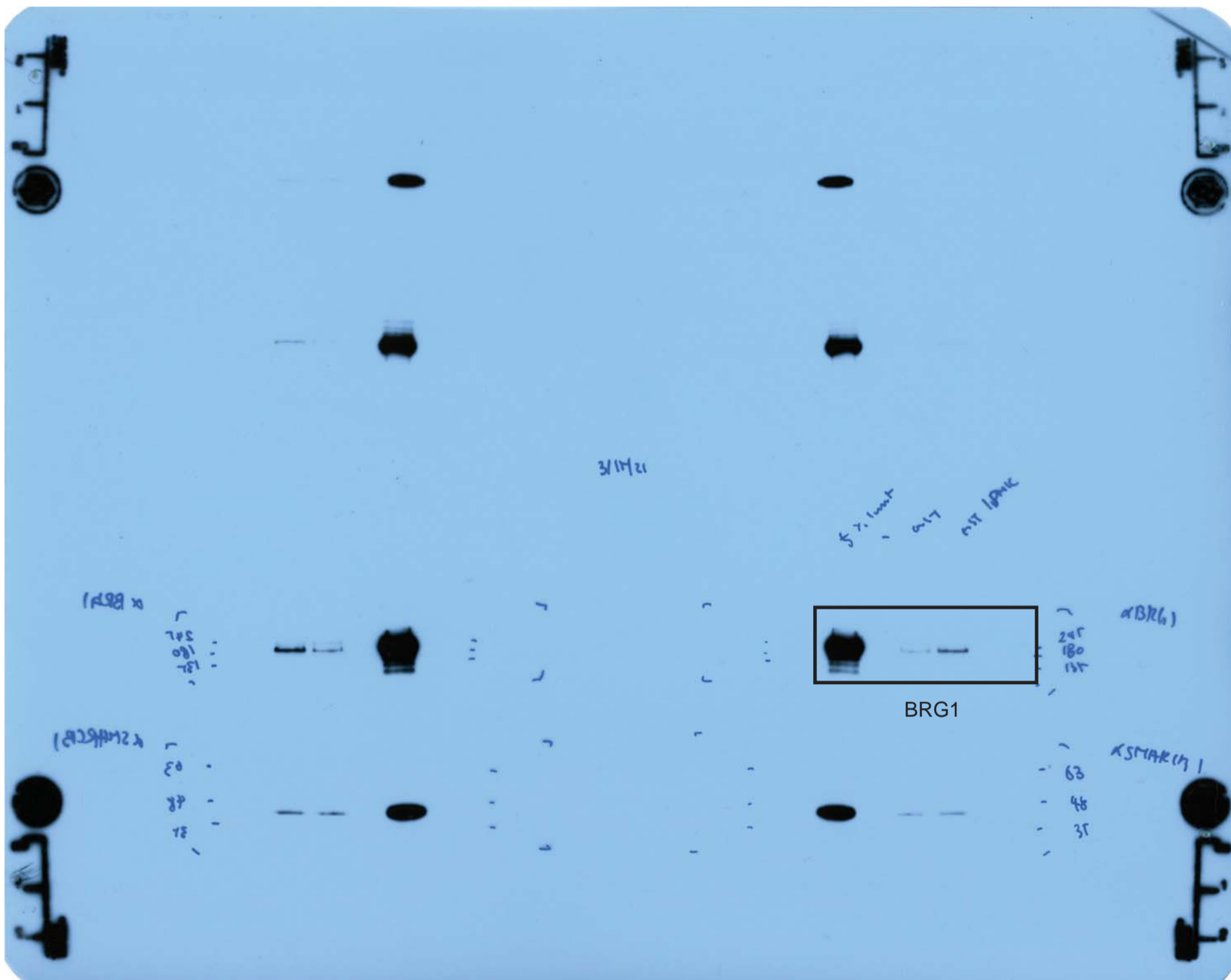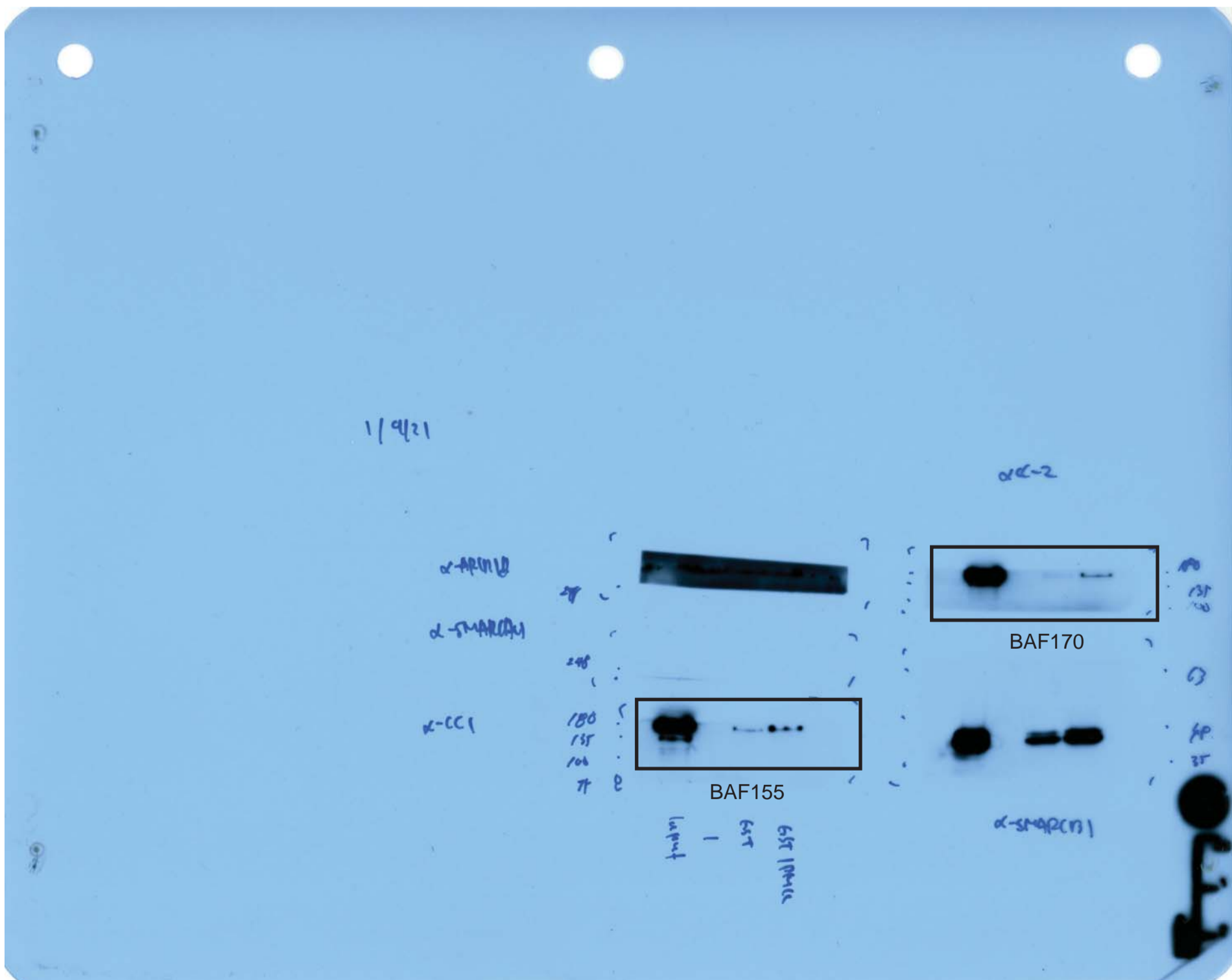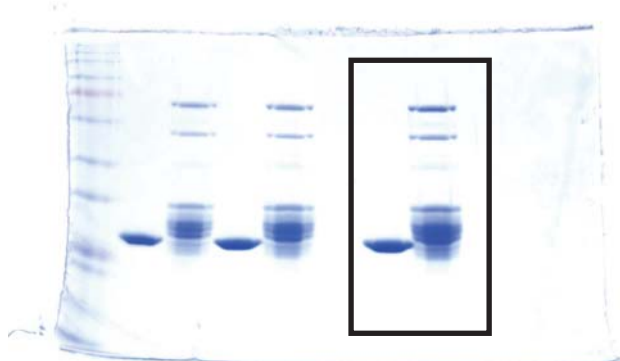

Supplement: Figure 2—figure supplement 1—source data 9. [file elife-73523-fig2-figsupp1-data9.zip › Labelled blots.pdf]

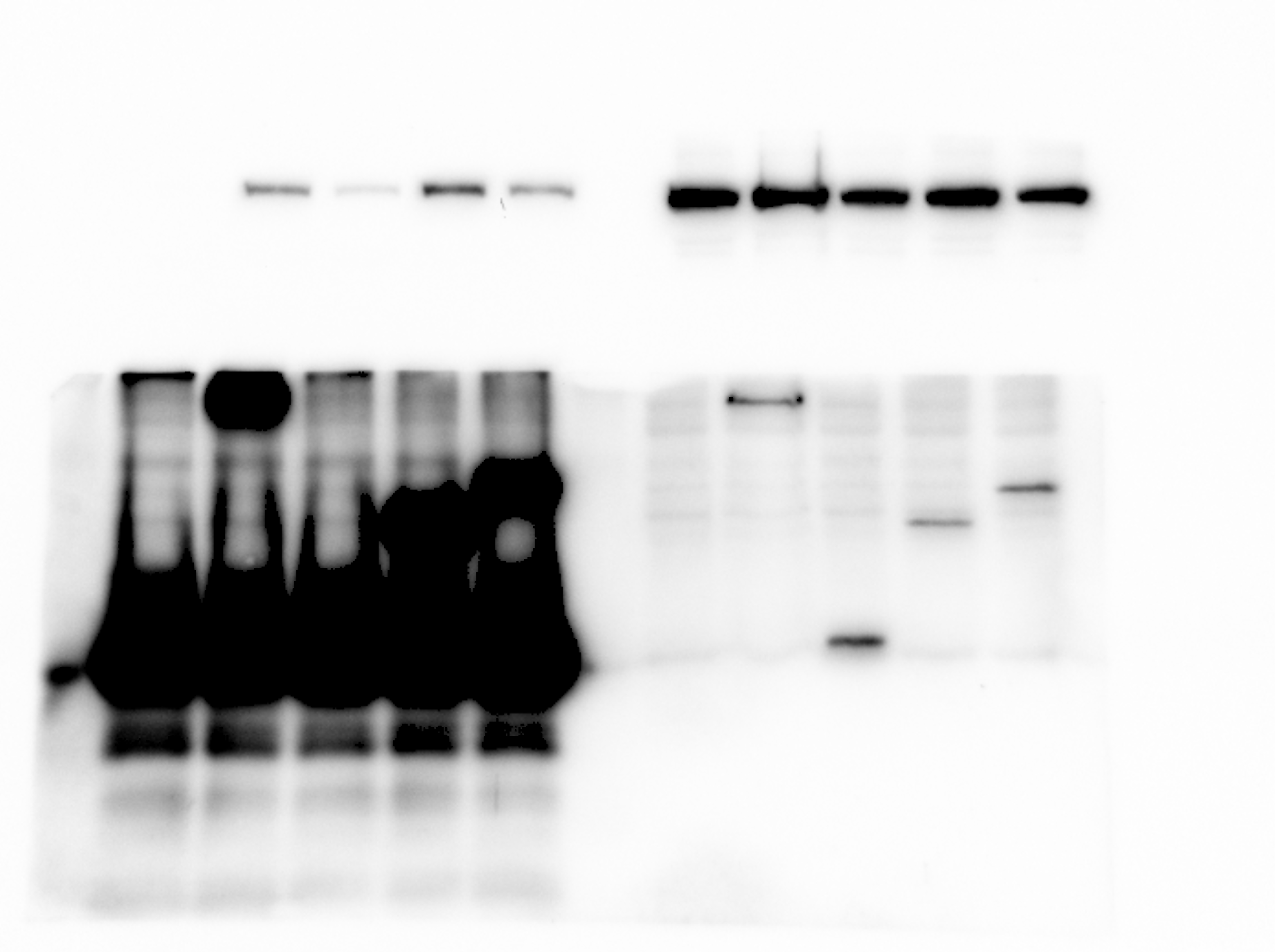

Supplement: Figure 3—source data 1. [file elife-73523-fig3-data1.zip › Raw blots/IP_ anti-GST and Input_ anti-FLAG.tif]

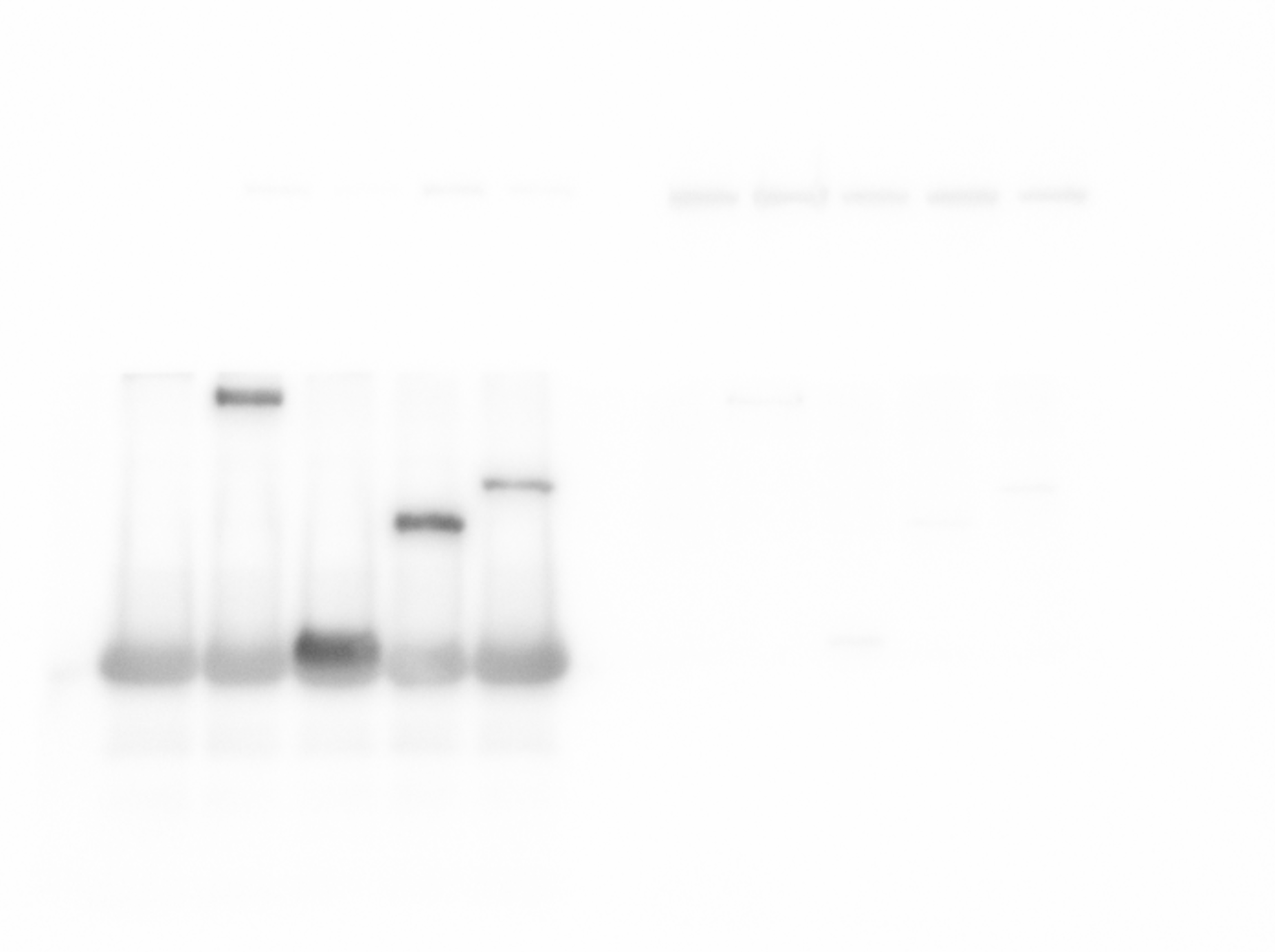

Supplement: Figure 3—source data 1. [file elife-73523-fig3-data1.zip › Raw blots/IP_ anti-FLAG.tif]

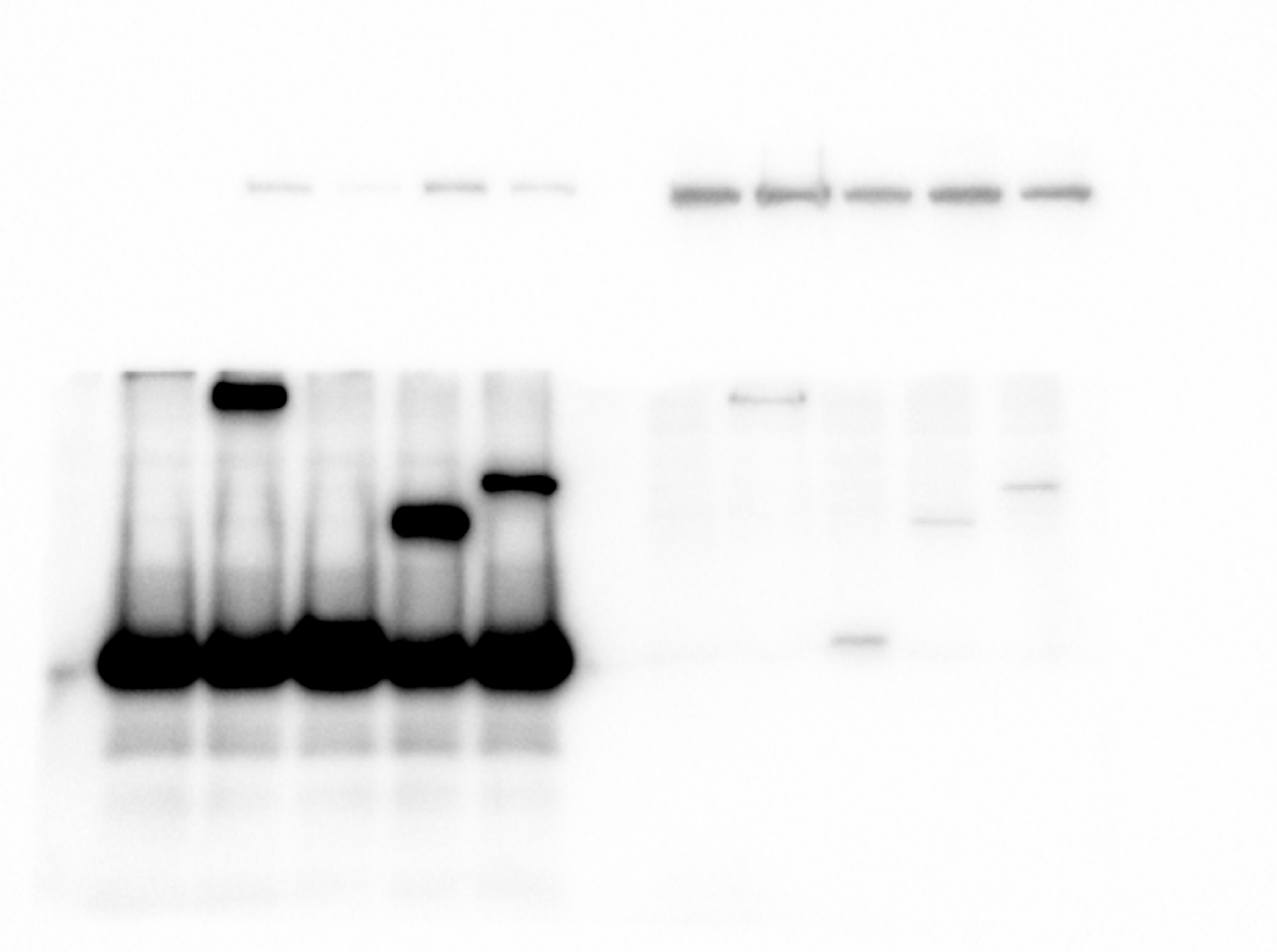

Supplement: Figure 3—source data 1. [file elife-73523-fig3-data1.zip › Raw blots/Input_ anti-GST.tif]

**B**

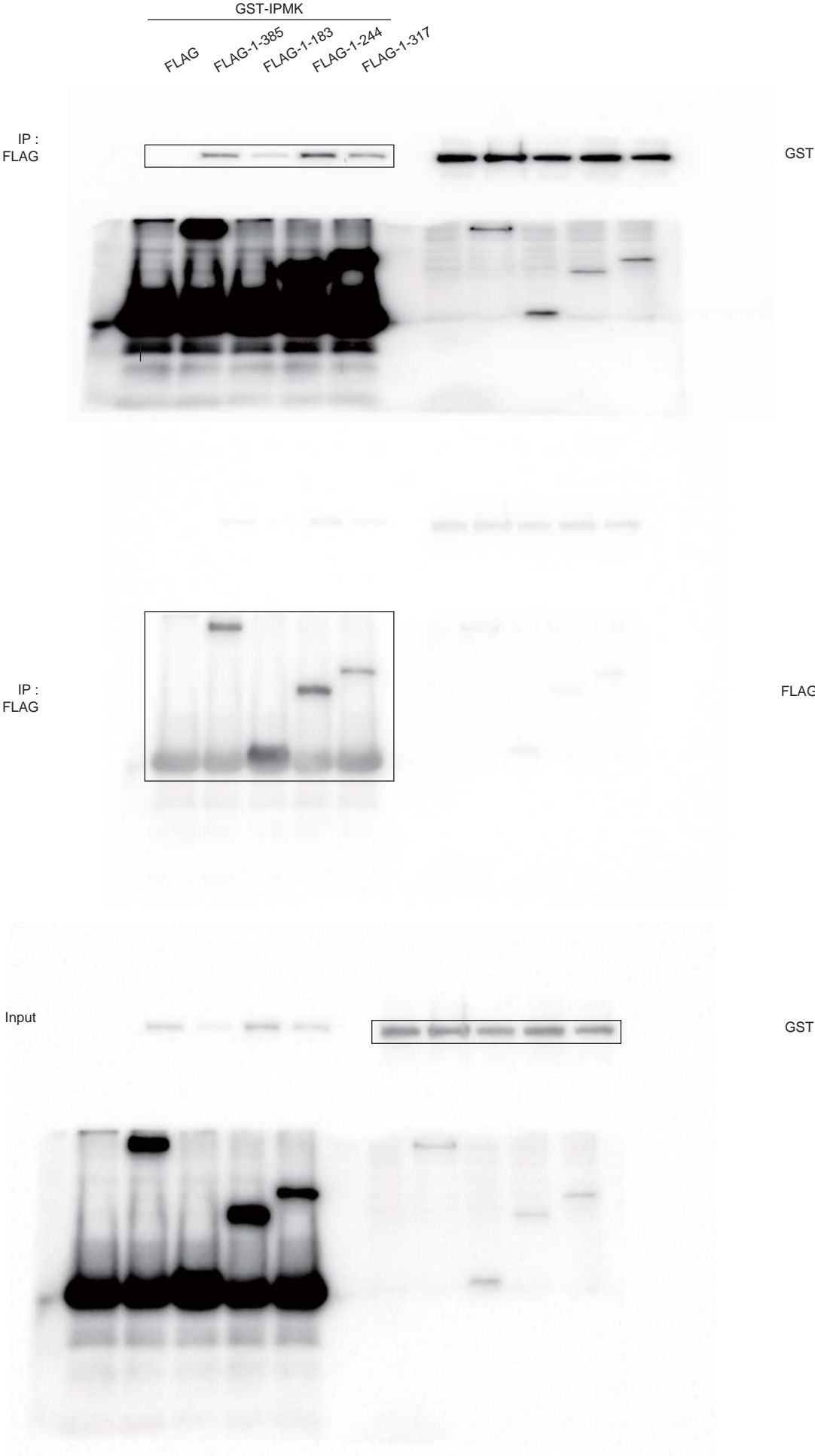

Supplement: Figure 3—source data 1. [file elife-73523-fig3-data1.zip › Labelled blots.pdf]

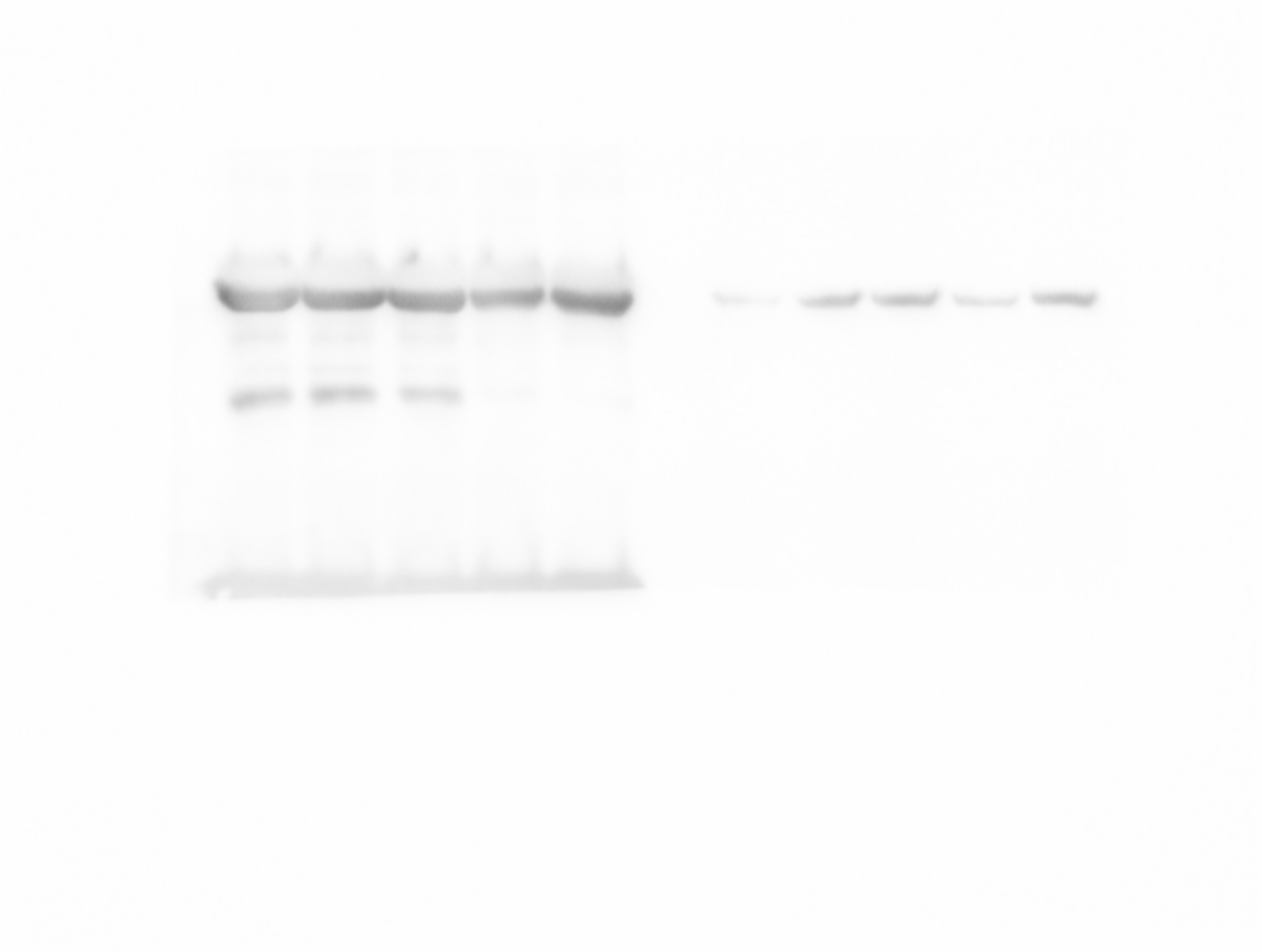

Supplement: Figure 3—source data 2. [file elife-73523-fig3-data2.zip › Raw blots/anti-FLAG.tif]

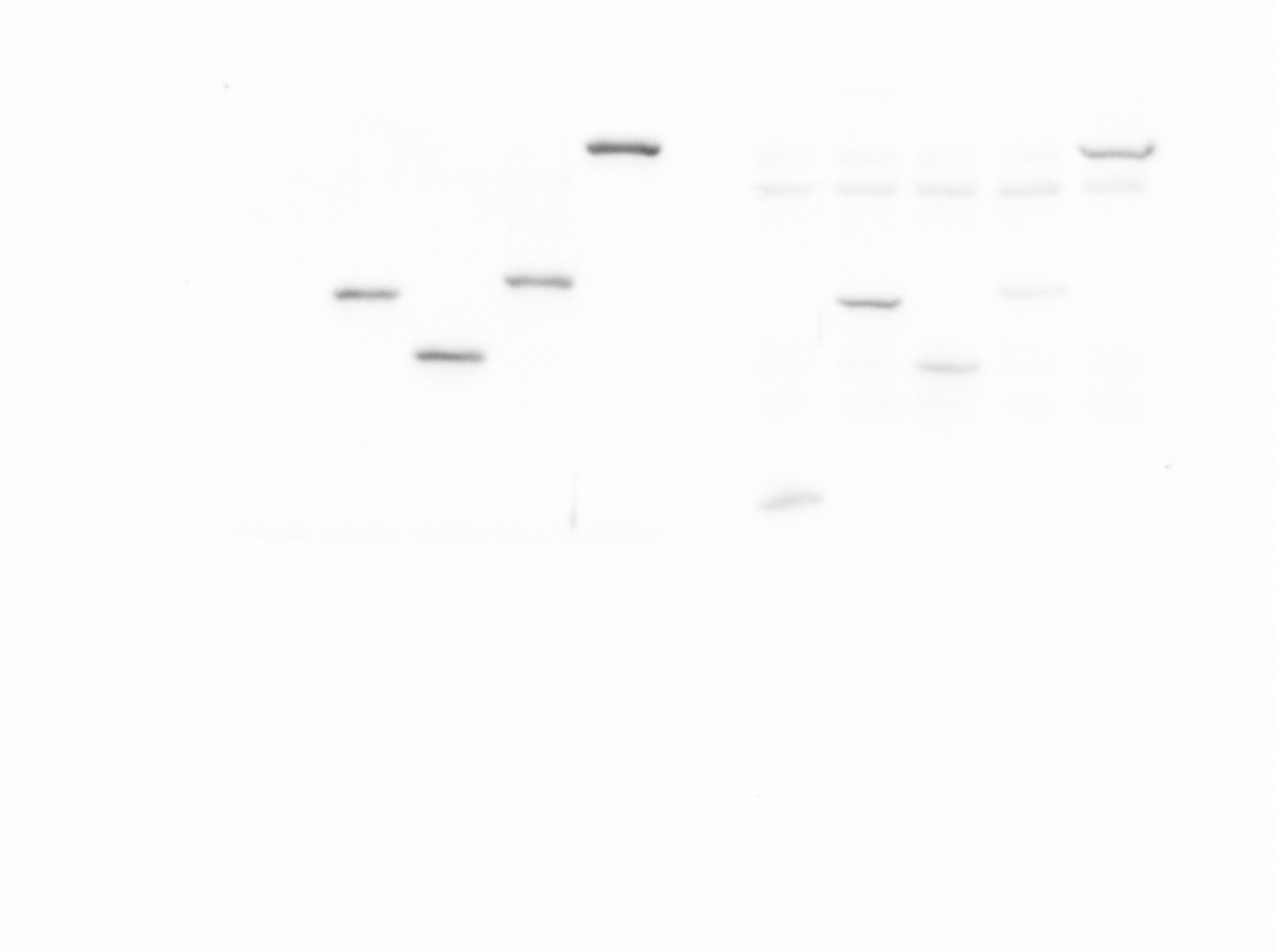

Supplement: Figure 3—source data 2. [file elife-73523-fig3-data2.zip › Raw blots/IP_ anti-GST.tif]

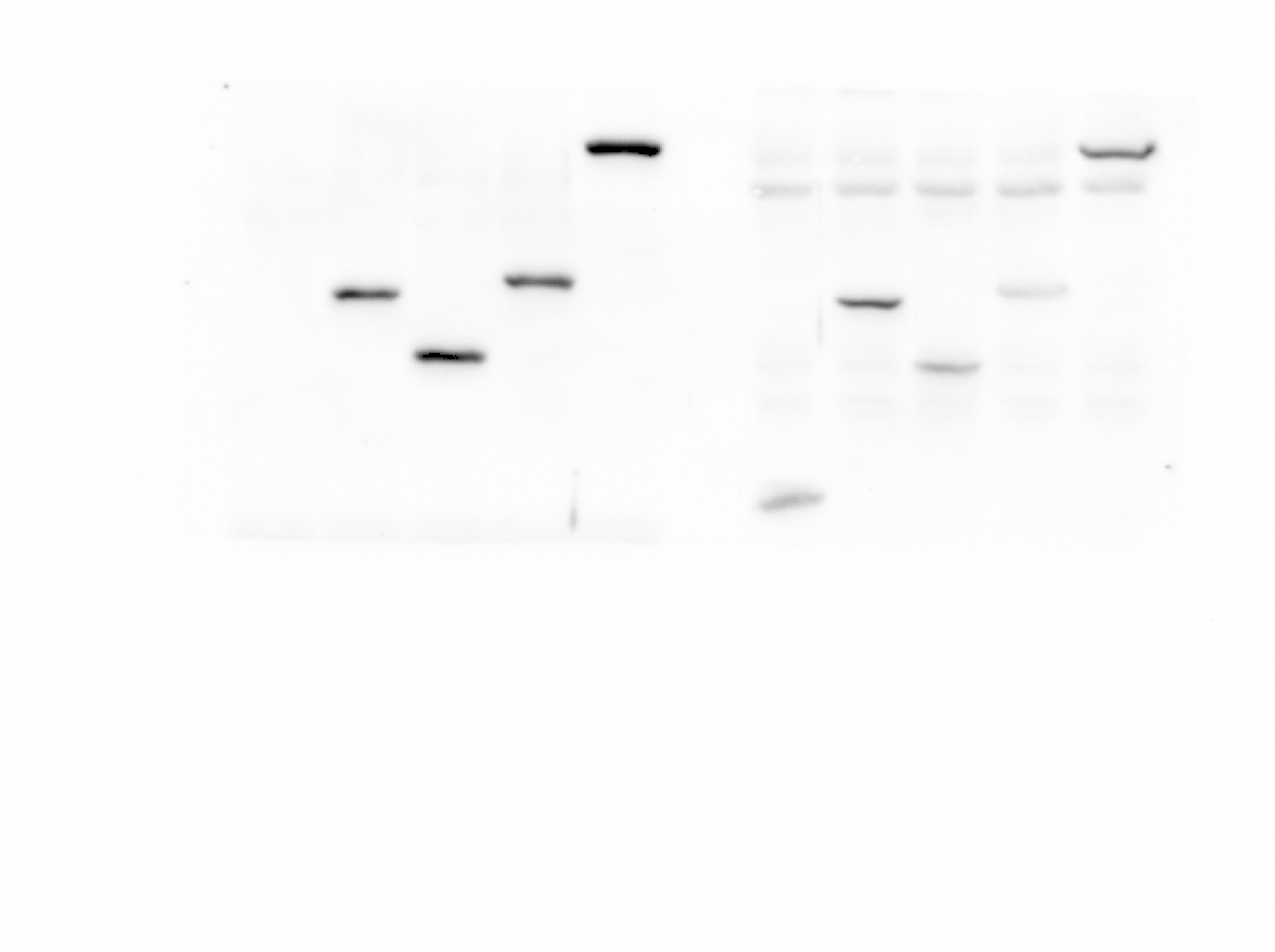

Supplement: Figure 3—source data 2. [file elife-73523-fig3-data2.zip › Raw blots/Input_ anti-GST.tif]

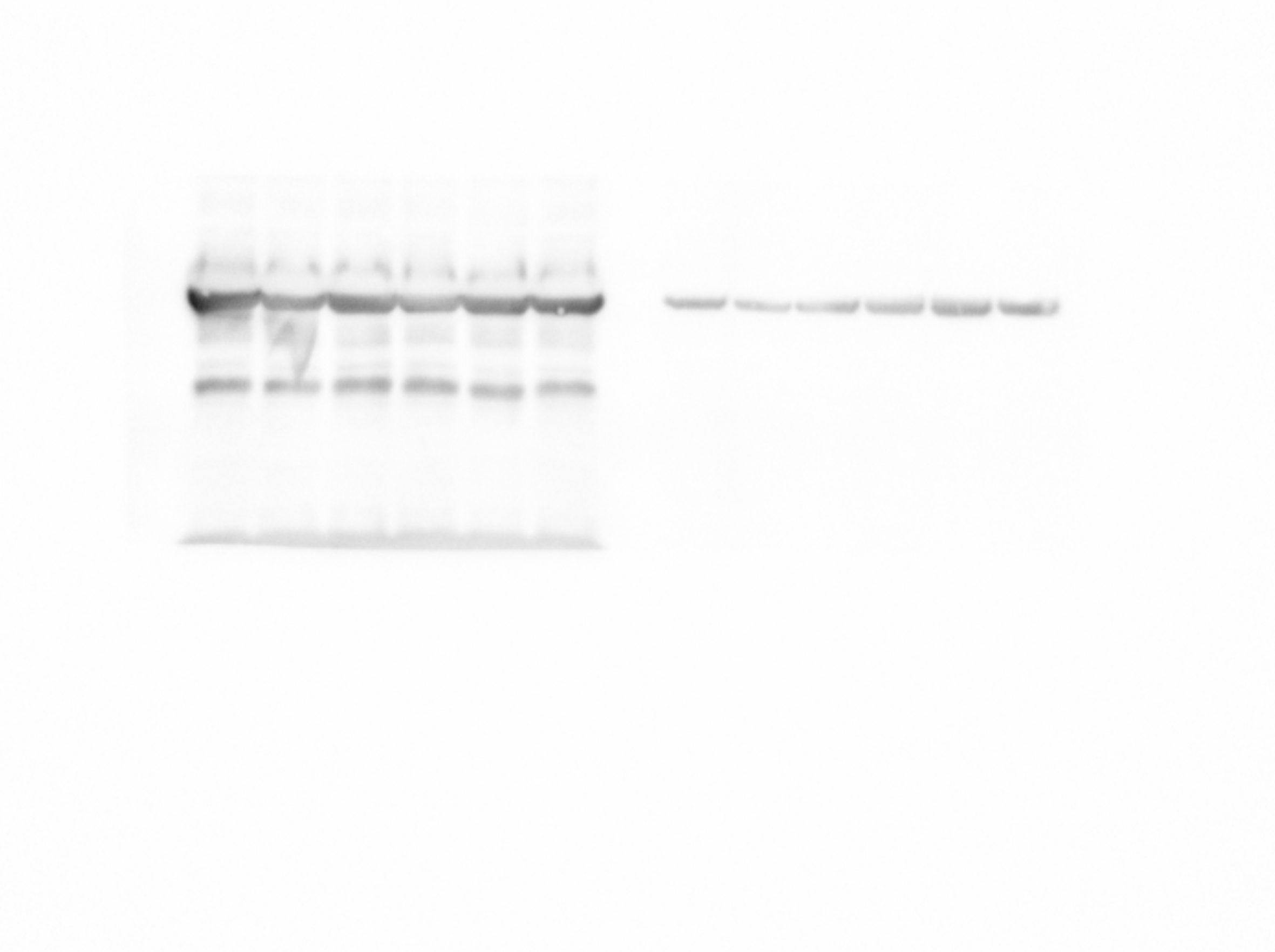

Supplement: Figure 3—source data 3. [file elife-73523-fig3-data3.zip › Raw blots/anti-FLAG.tif]

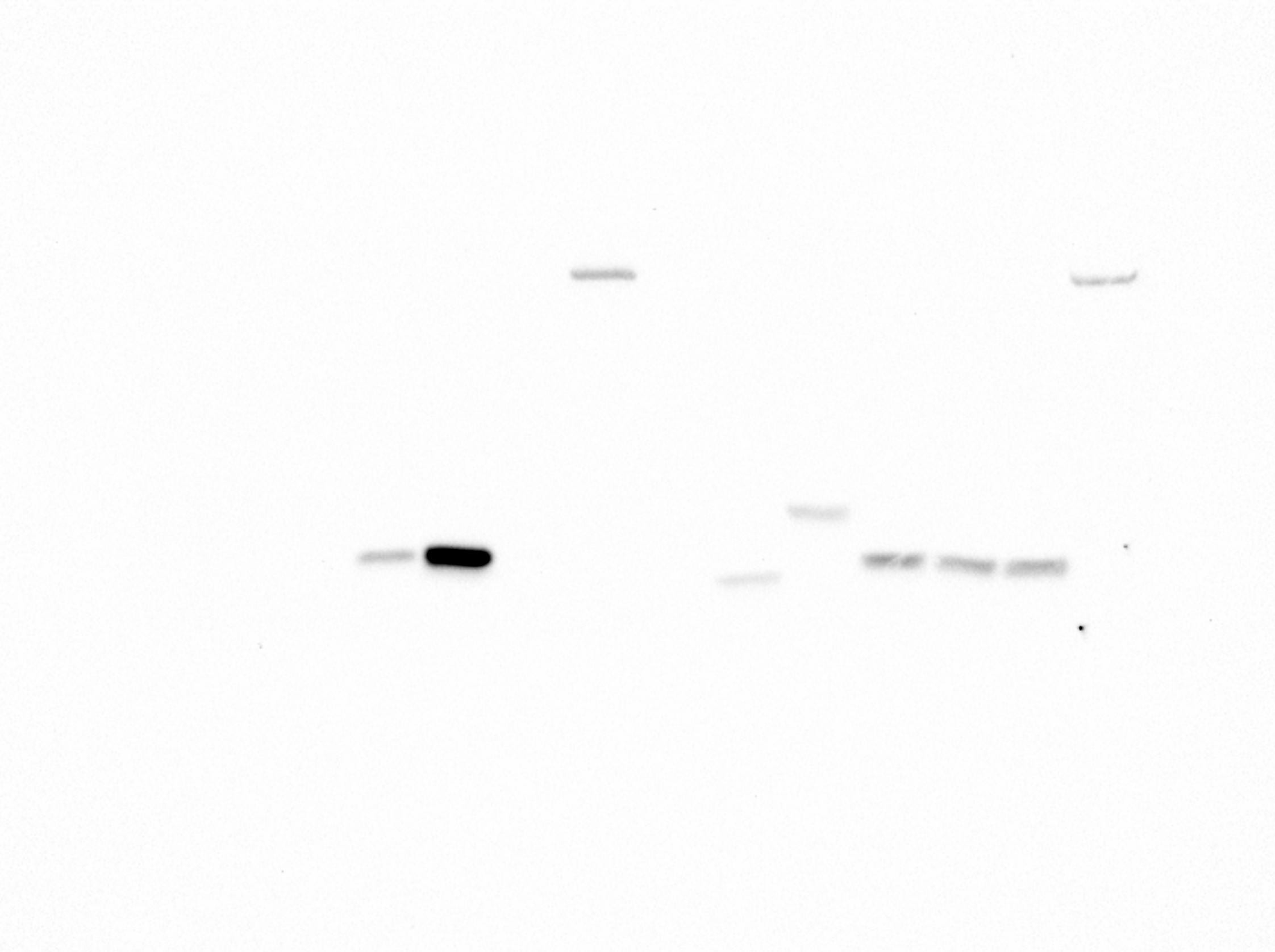

Supplement: Figure 3—source data 3. [file elife-73523-fig3-data3.zip › Raw blots/anti-GST.tif]

D

FLAG-IPMK

GST

GST-105-183

GST-186-244

GST-259-317

GST-335-378

GST-1-385

IP :  
FLAG

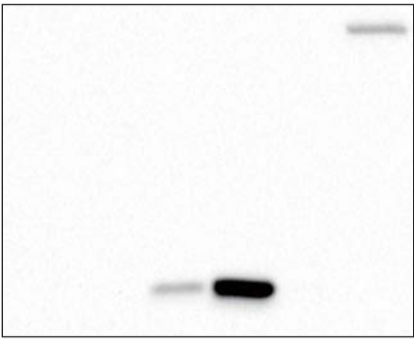

GST

IP :  
FLAG

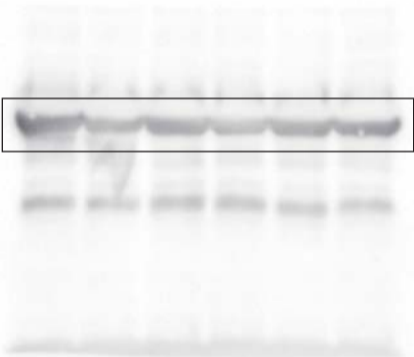

FLAG

Input

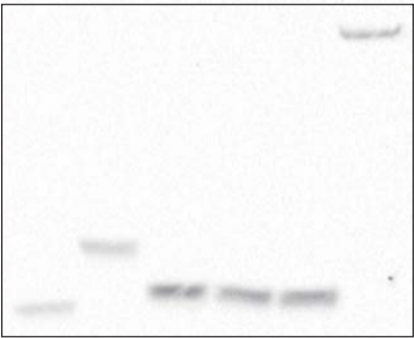

GST

Supplement: Figure 3—source data 3. [file elife-73523-fig3-data3.zip › Labelled blots.pdf]

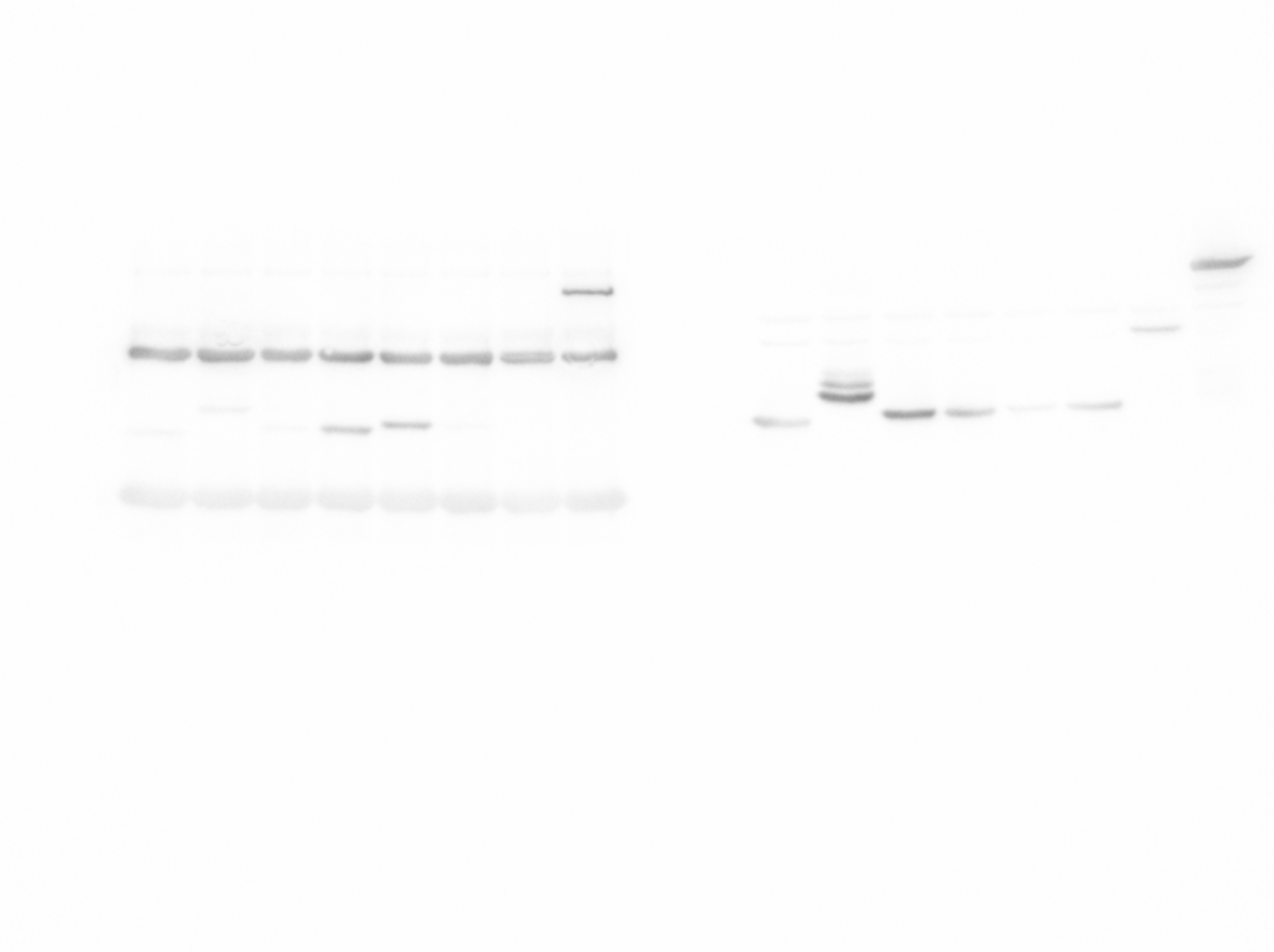

Supplement: Figure 3—source data 4. [file elife-73523-fig3-data4.zip › Raw blots/anti-FLAG.tif]

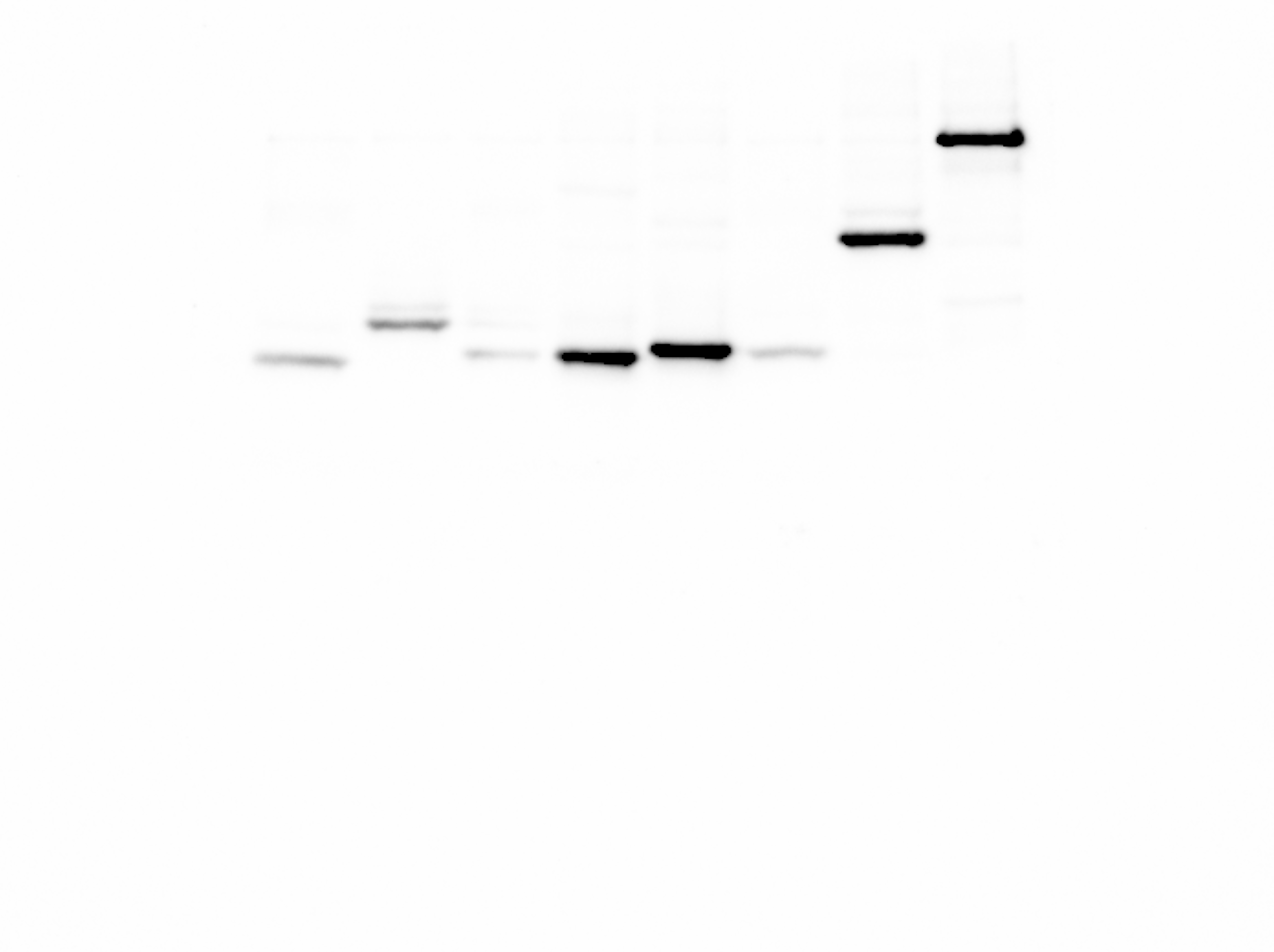

Supplement: Figure 3—source data 4. [file elife-73523-fig3-data4.zip › Raw blots/IP_ anti-GST.tif]

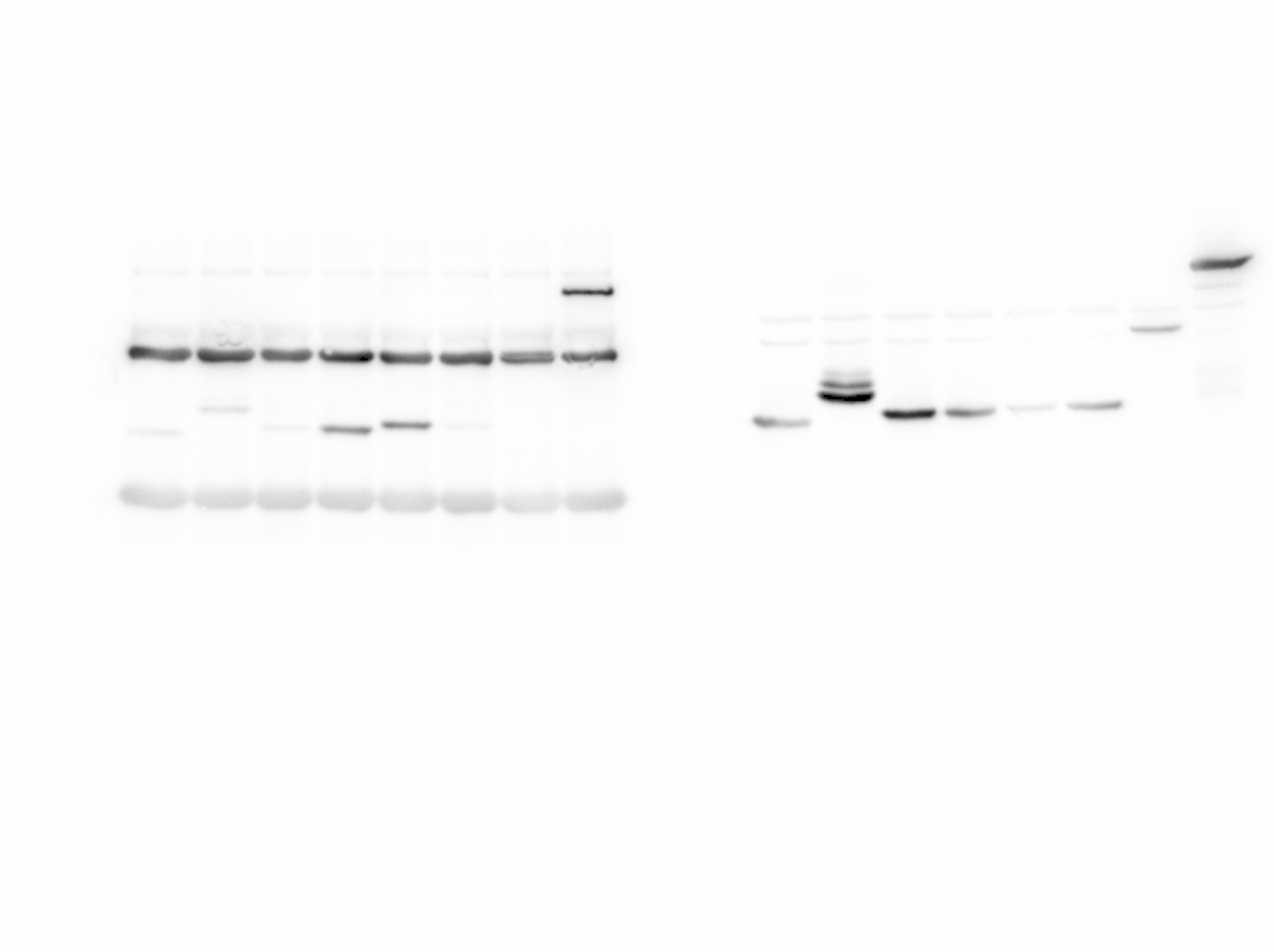

Supplement: Figure 3—source data 4. [file elife-73523-fig3-data4.zip › Raw blots/Input_ anti-GST.tif]

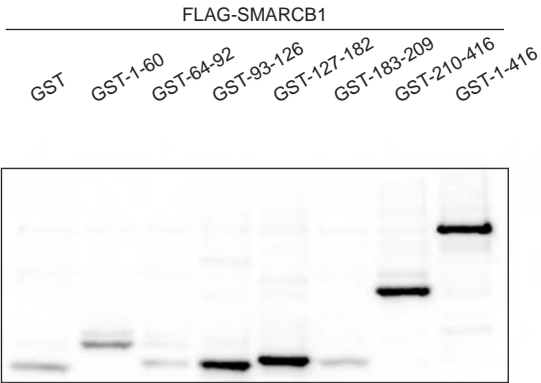

GST

IP :  
FLAG

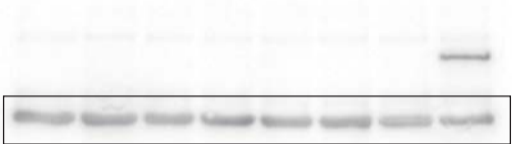

FLAG

Input

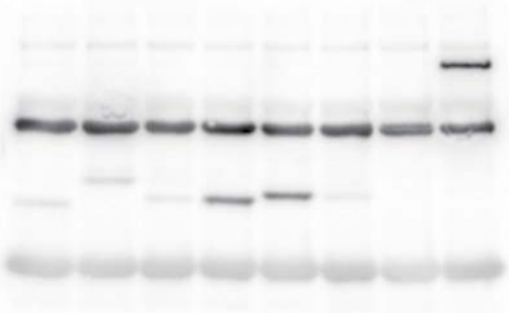

GST

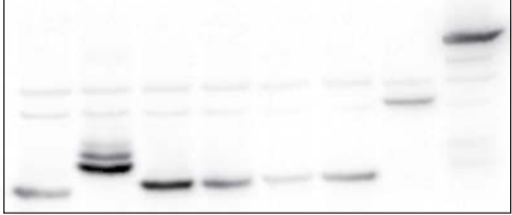

Supplement: Figure 3—source data 4. [file elife-73523-fig3-data4.zip › Labelled blots.pdf]

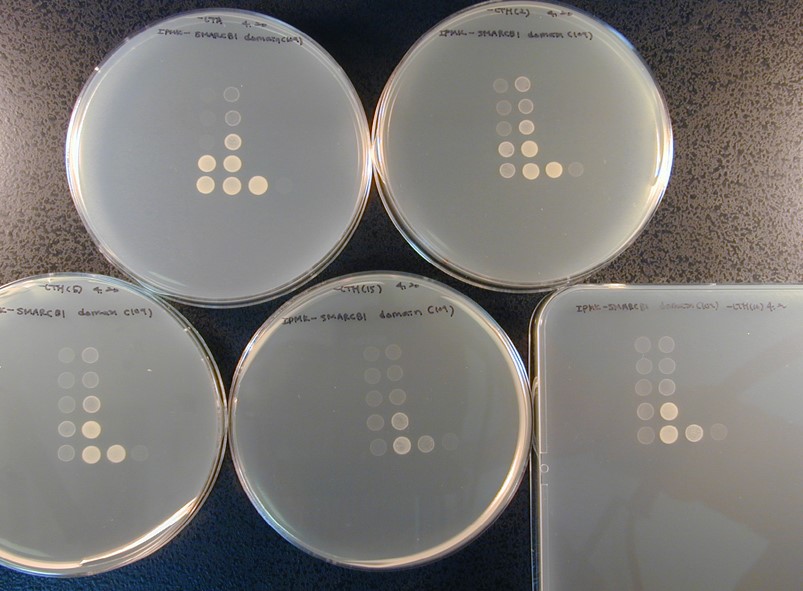

Supplement: Figure 3—figure supplement 1—source data 1. [file elife-73523-fig3-figsupp1-data1.zip › Raw image.jpg]

A

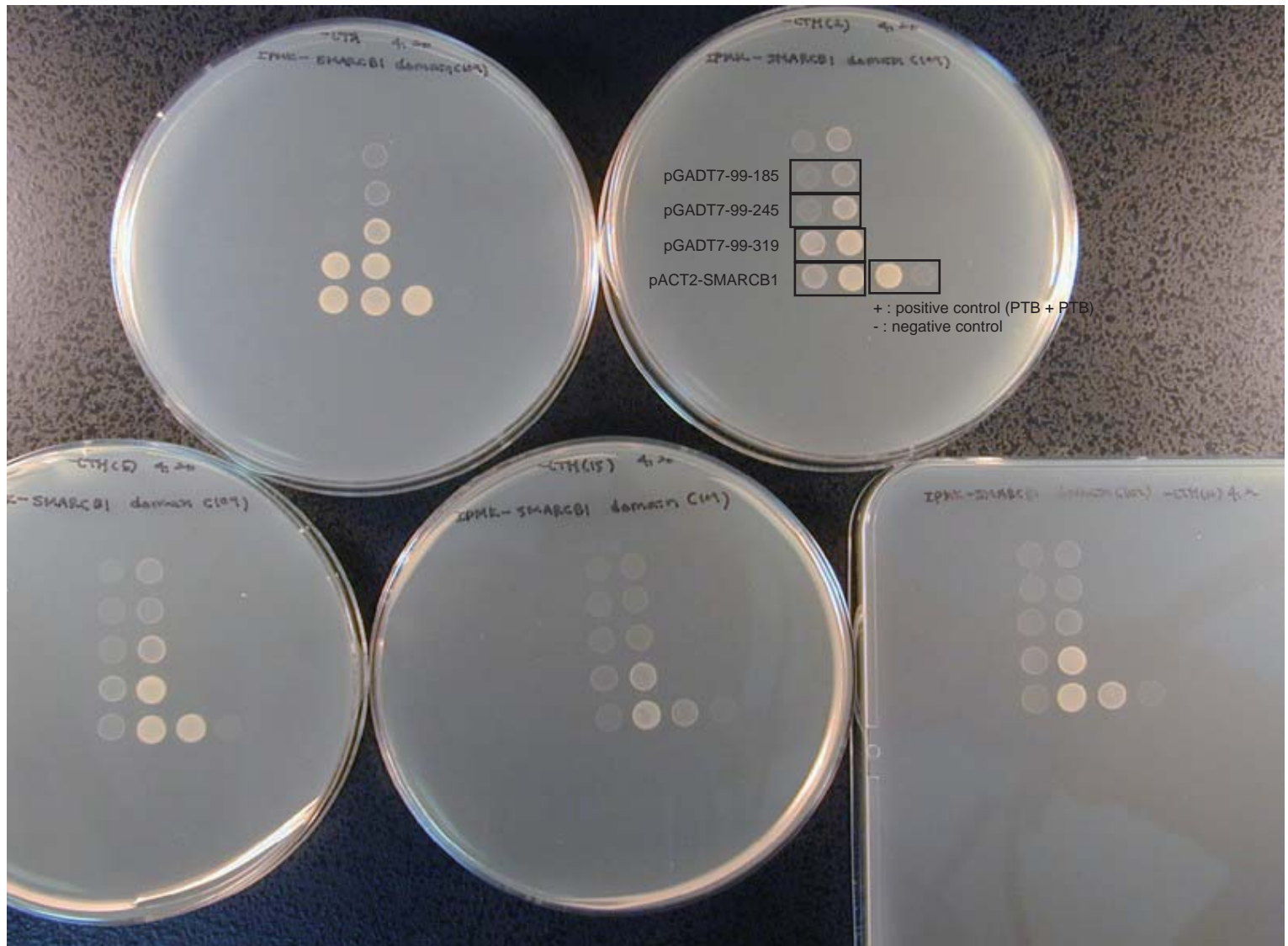

Supplement: Figure 3—figure supplement 1—source data 1. [file elife-73523-fig3-figsupp1-data1.zip › Labelled image.pdf]

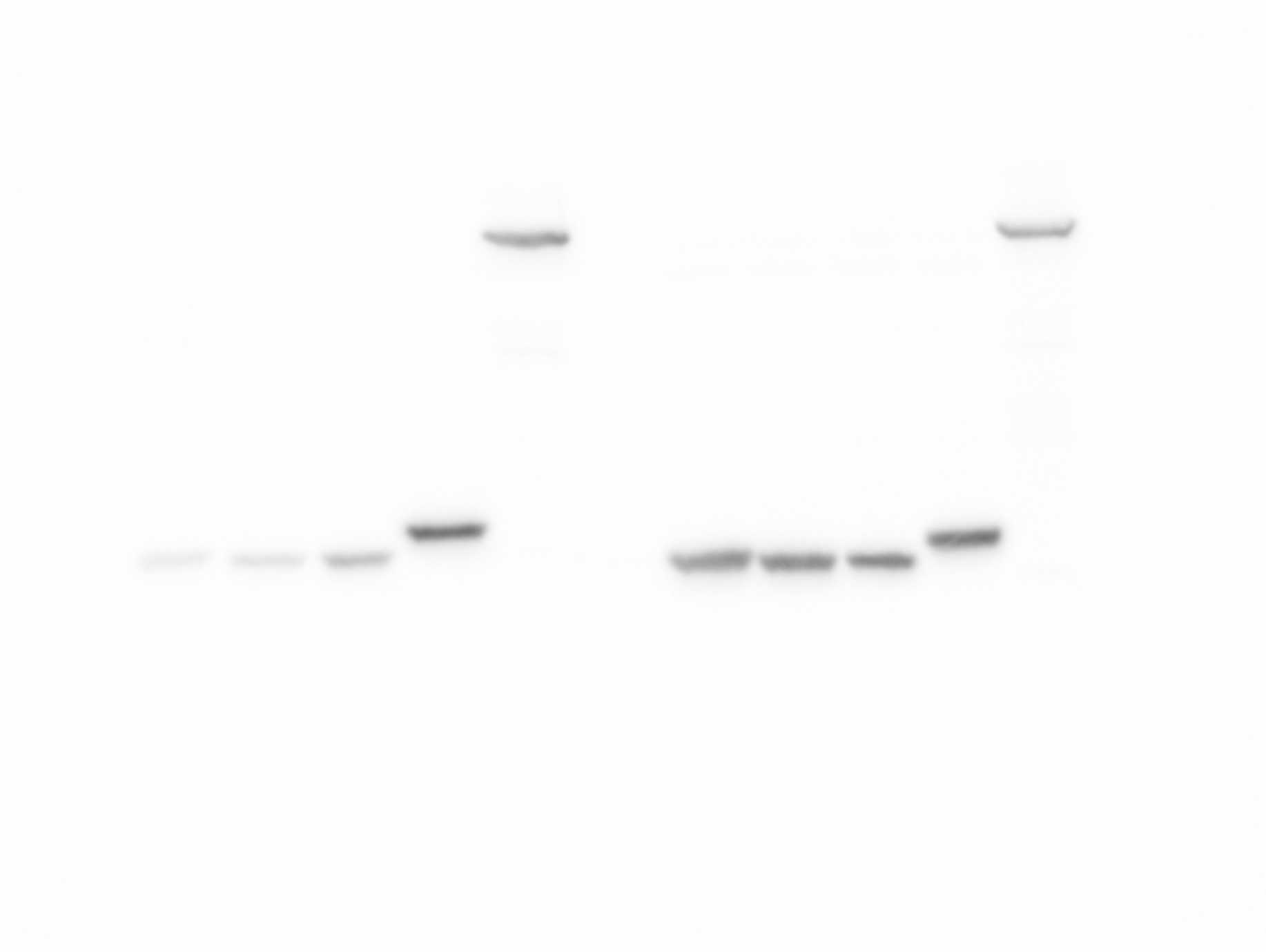

Supplement: Figure 3—figure supplement 1—source data 2. [file elife-73523-fig3-figsupp1-data2.zip › Raw blots/IP_ anti-GST.tif]

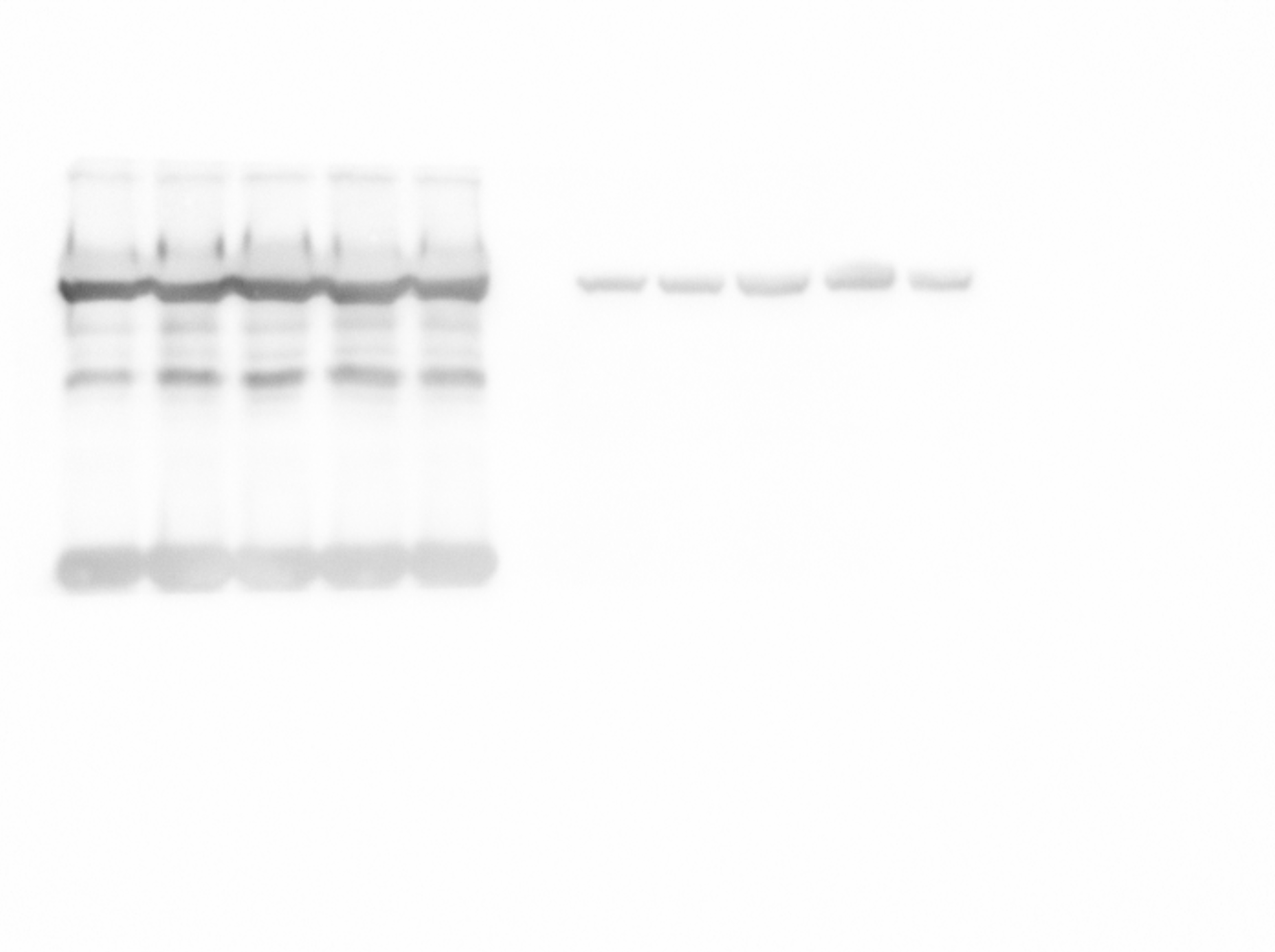

Supplement: Figure 3—figure supplement 1—source data 2. [file elife-73523-fig3-figsupp1-data2.zip › Raw blots/IP_ anti-FLAG.tif]

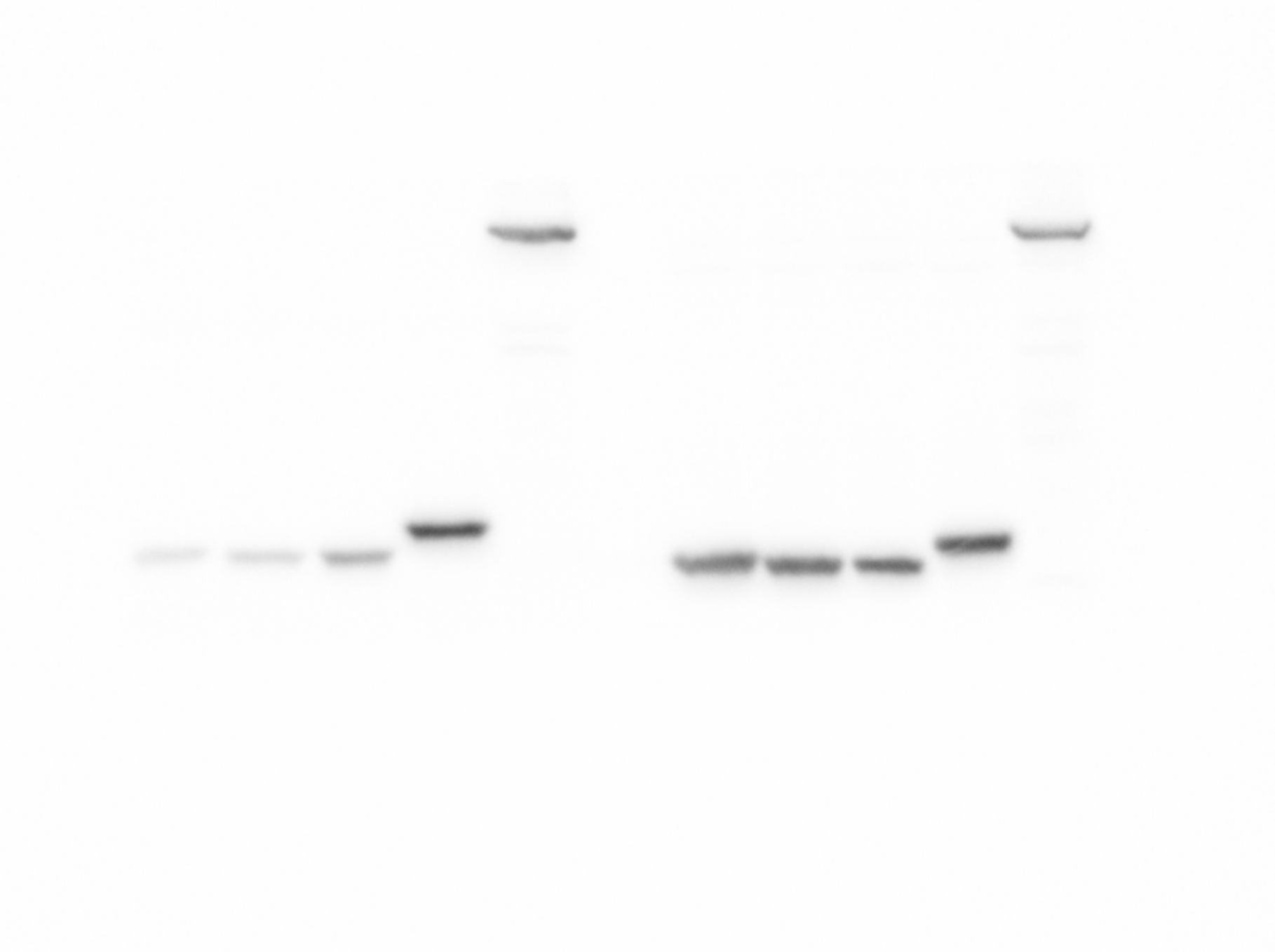

Supplement: Figure 3—figure supplement 1—source data 2. [file elife-73523-fig3-figsupp1-data2.zip › Raw blots/Input_ anti-GST.tif]

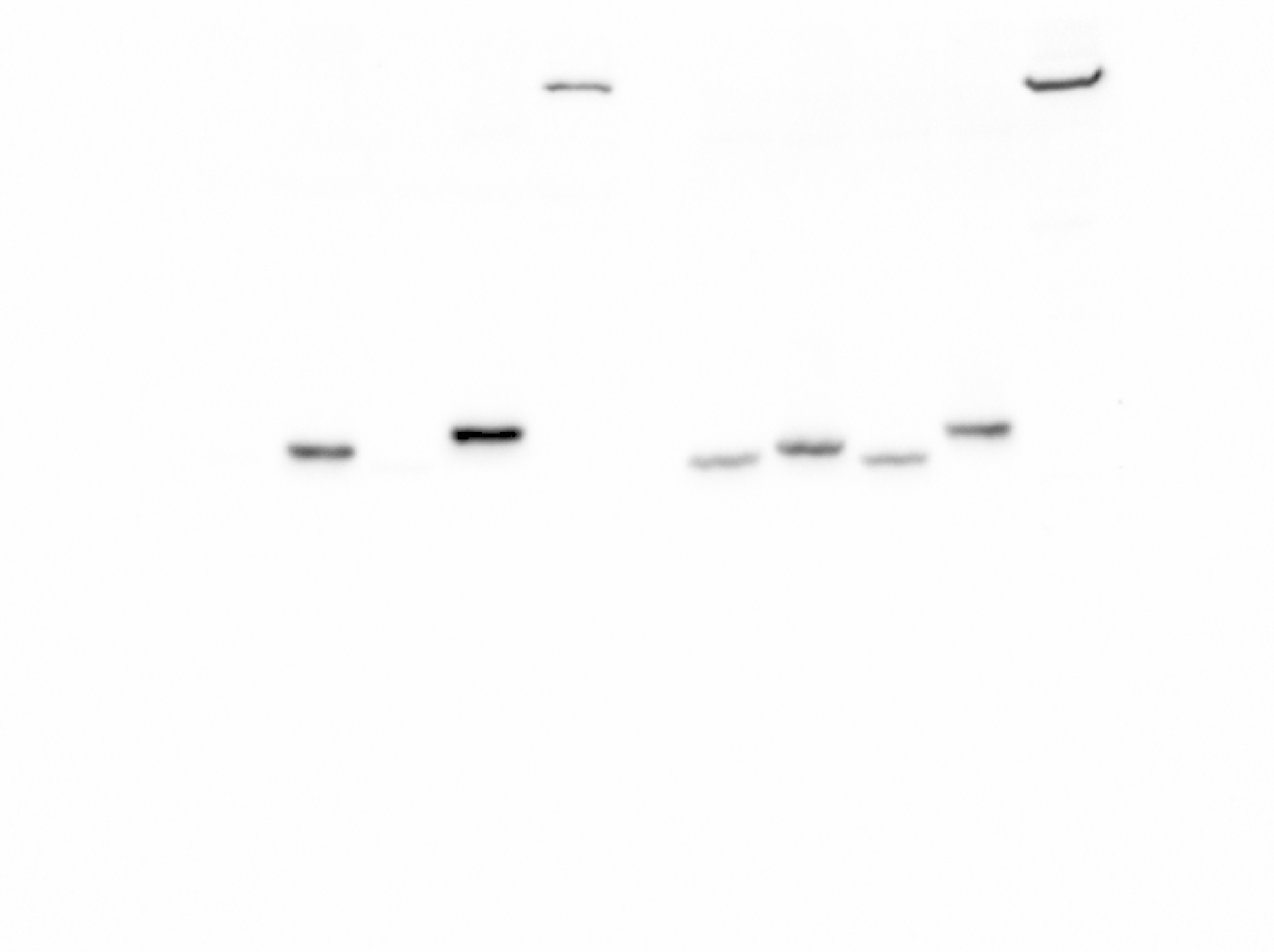

Supplement: Figure 3—figure supplement 1—source data 3. [file elife-73523-fig3-figsupp1-data3.zip › Raw blots/IP_ anti-GST.tif]

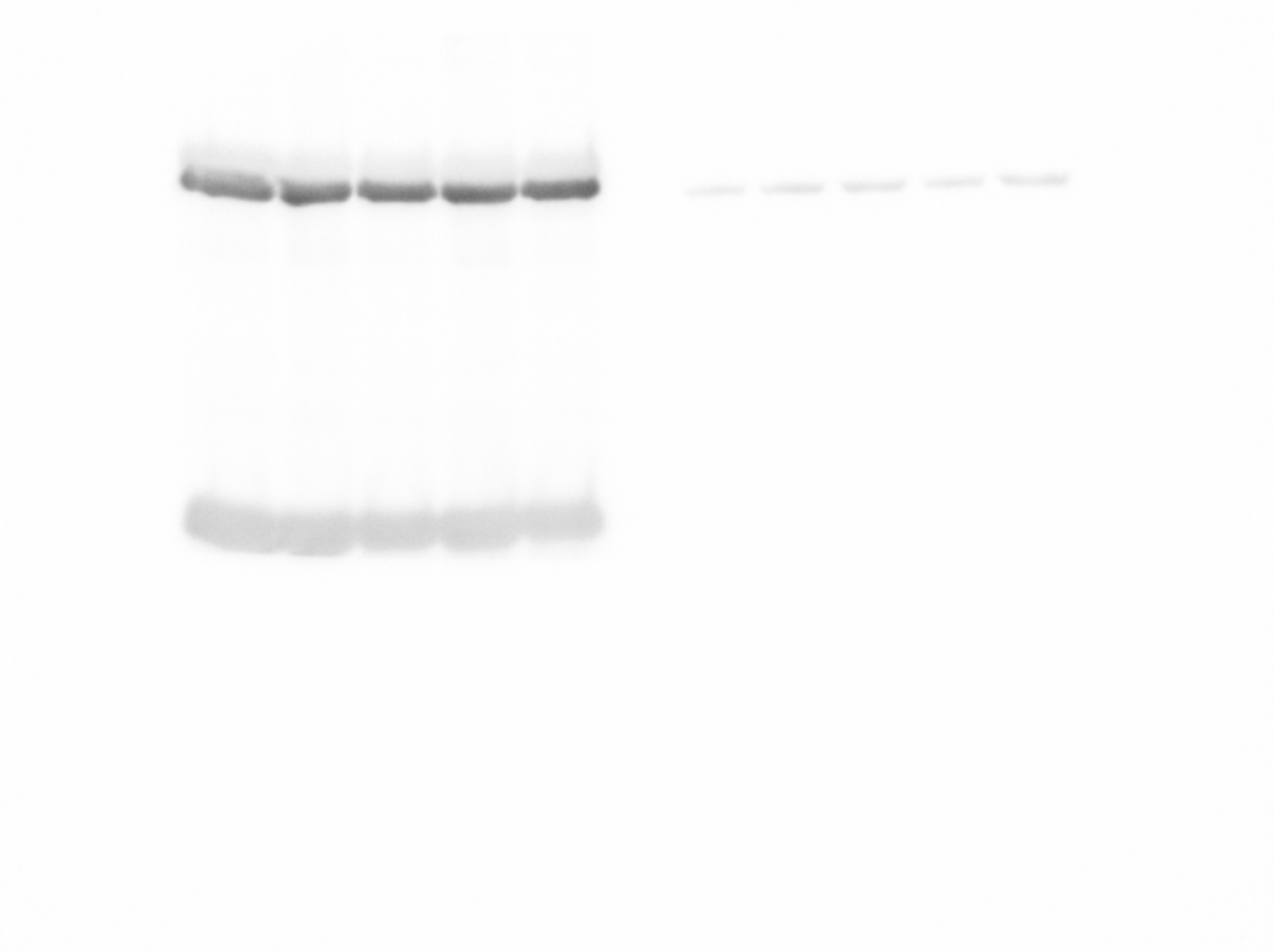

Supplement: Figure 3—figure supplement 1—source data 3. [file elife-73523-fig3-figsupp1-data3.zip › Raw blots/IP_ anti-FLAG.tif]

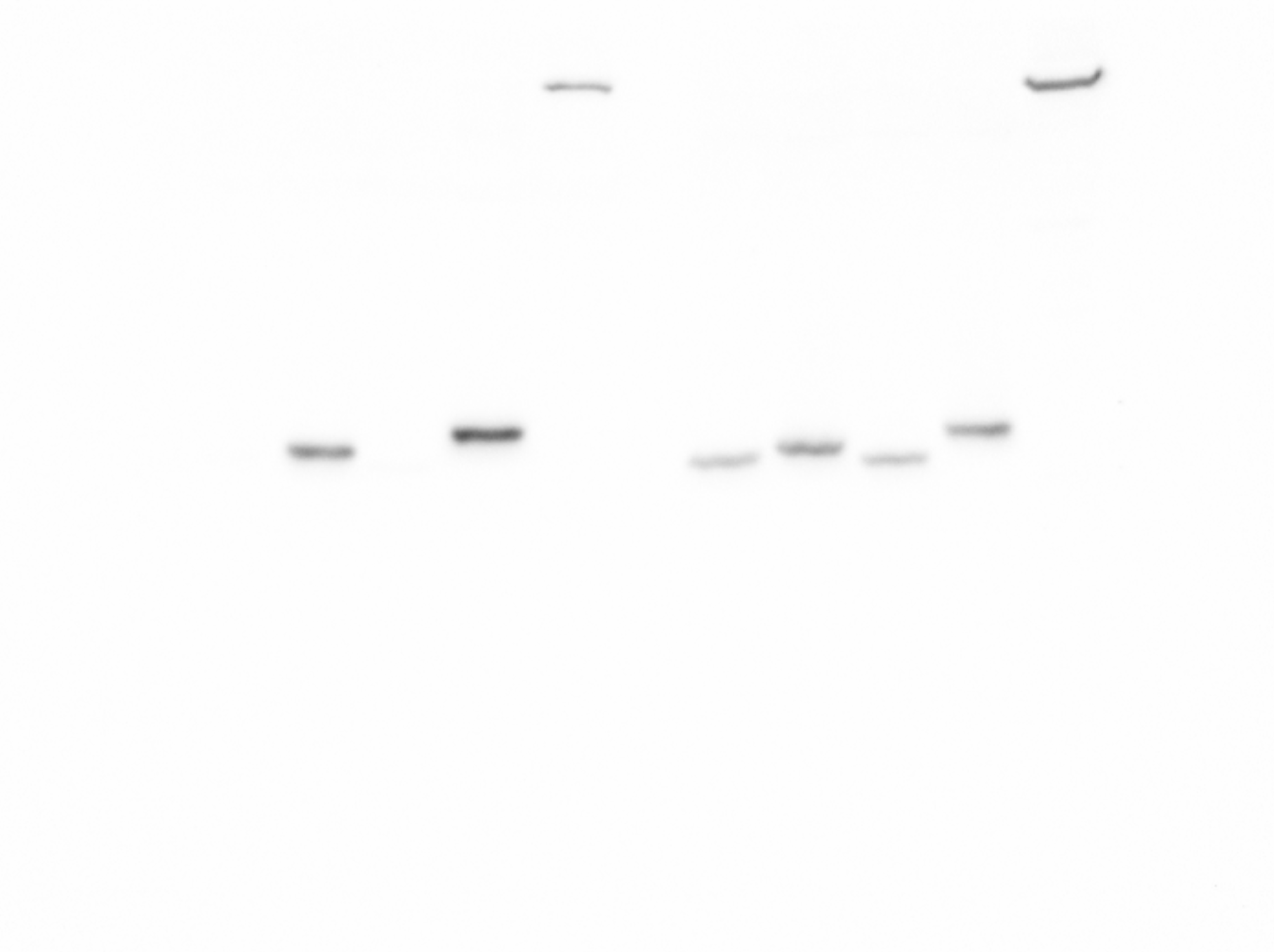

Supplement: Figure 3—figure supplement 1—source data 3. [file elife-73523-fig3-figsupp1-data3.zip › Raw blots/Input- anti-GST.tif]

C

GST GST-259-287 GST-288-317 GST-259-317 GST-1-385

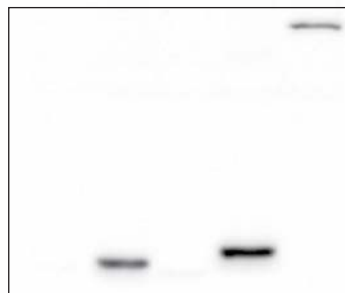

IP :  
FLAG

GST

IP :  
FLAG

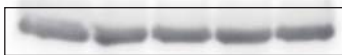

FLAG

Input

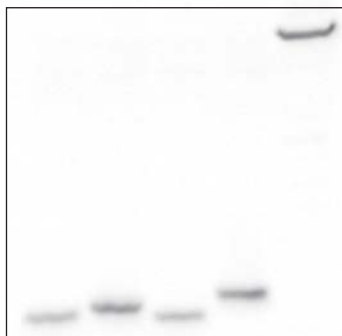

GST

Supplement: Figure 3—figure supplement 1—source data 3. [file elife-73523-fig3-figsupp1-data3.zip › Labelled blots.pdf]

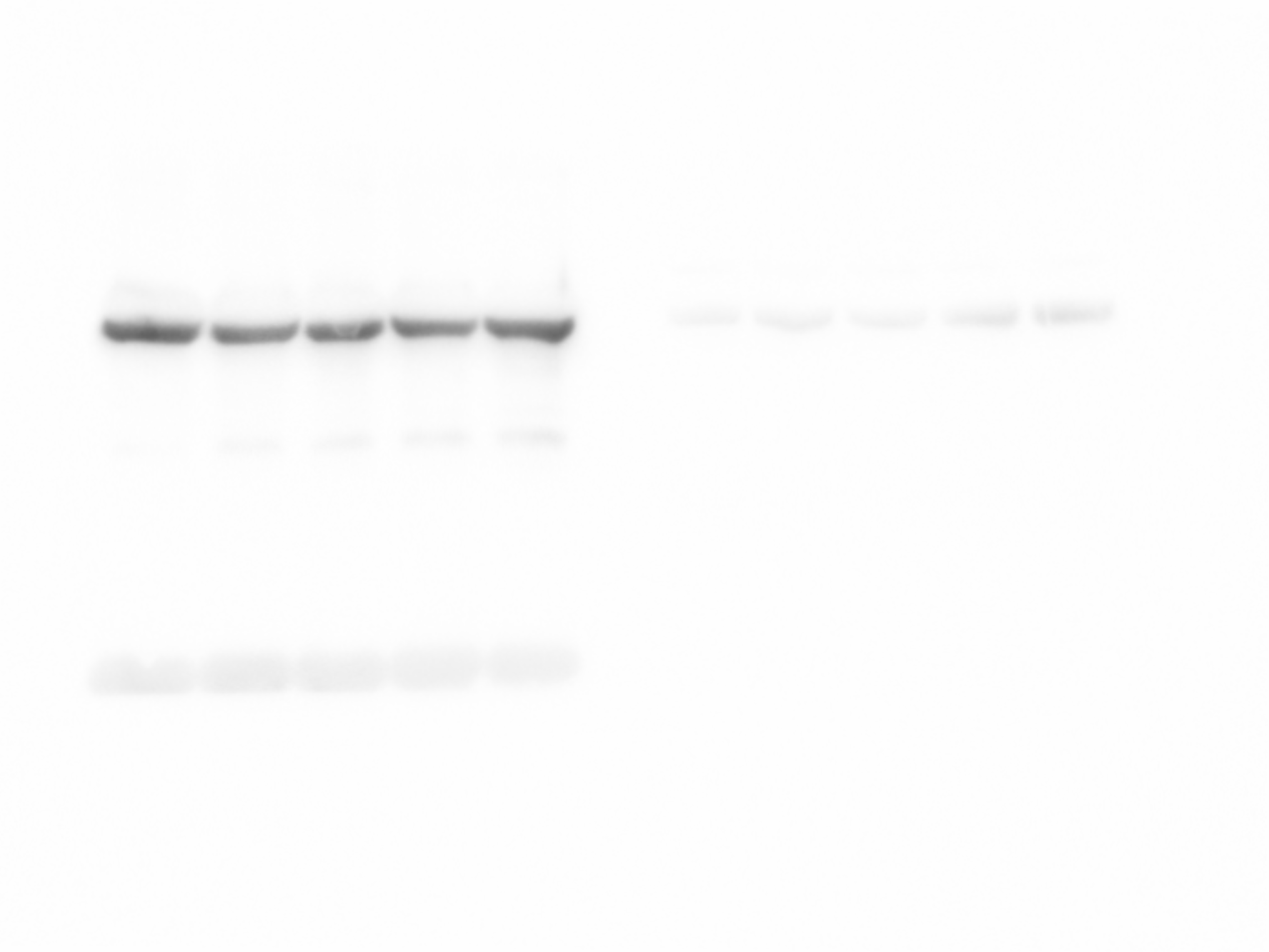

Supplement: Figure 3—figure supplement 1—source data 4. [file elife-73523-fig3-figsupp1-data4.zip › Raw blots/anti-FLAG.tif]

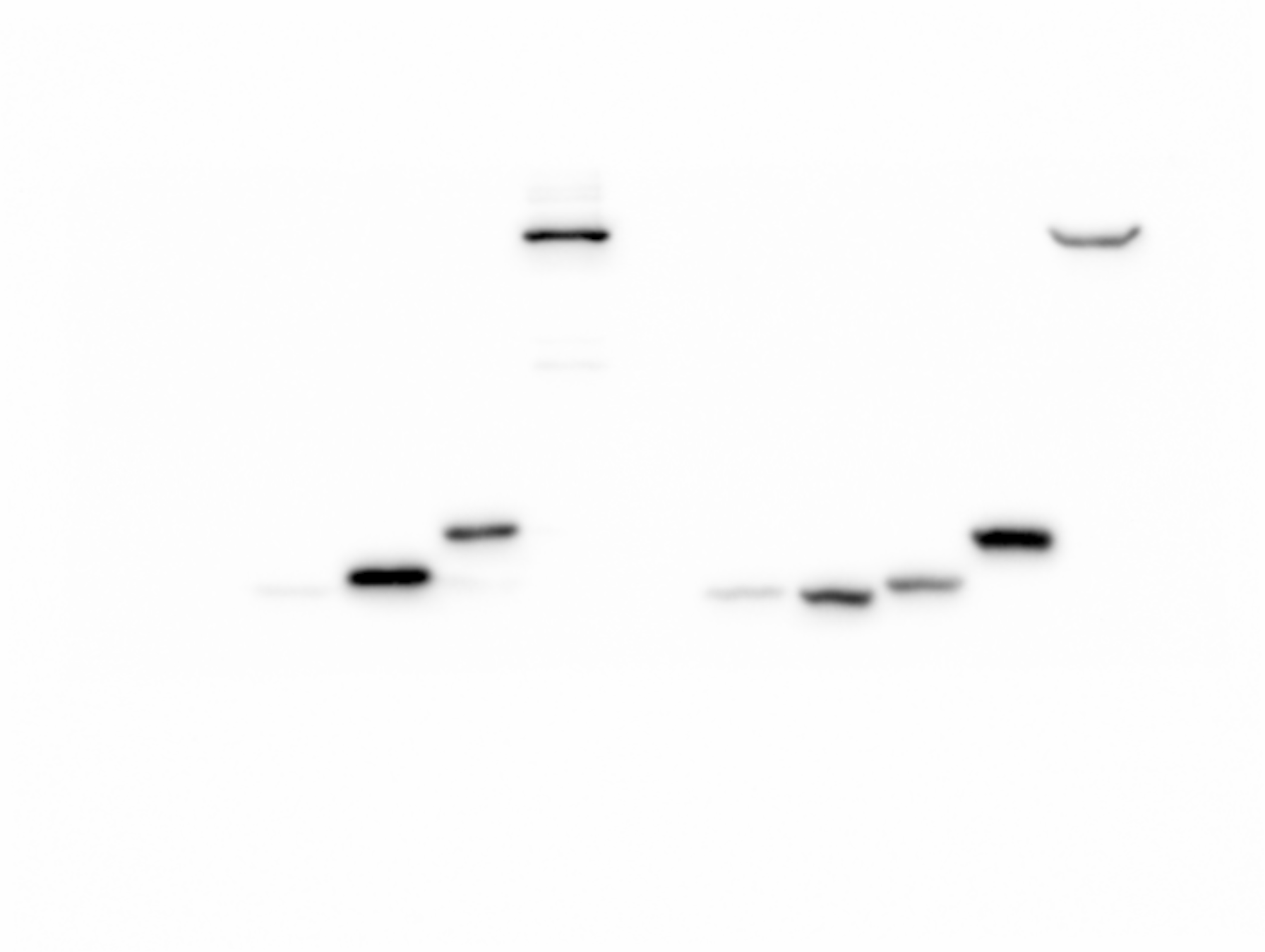

Supplement: Figure 3—figure supplement 1—source data 4. [file elife-73523-fig3-figsupp1-data4.zip › Raw blots/anti-GST.tif]

D

GST GST-211-244 GST-259-287 GST-211-287 GST-1-385

IP :  
FLAG

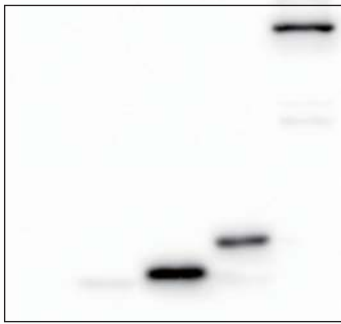

GST

IP :  
FLAG

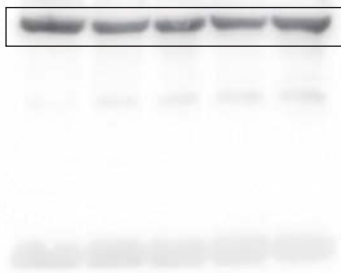

FLAG

Input

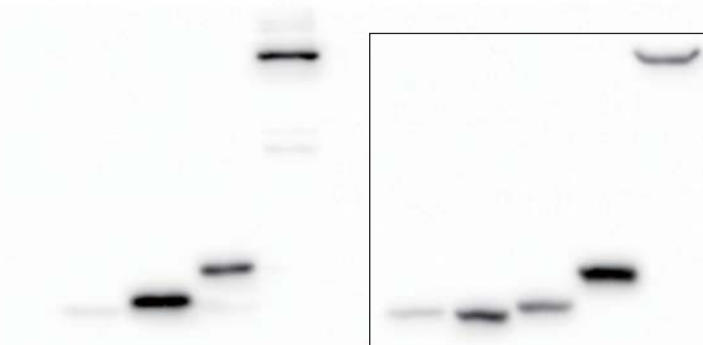

GST

Supplement: Figure 3—figure supplement 1—source data 4. [file elife-73523-fig3-figsupp1-data4.zip › Labelled blots.pdf]

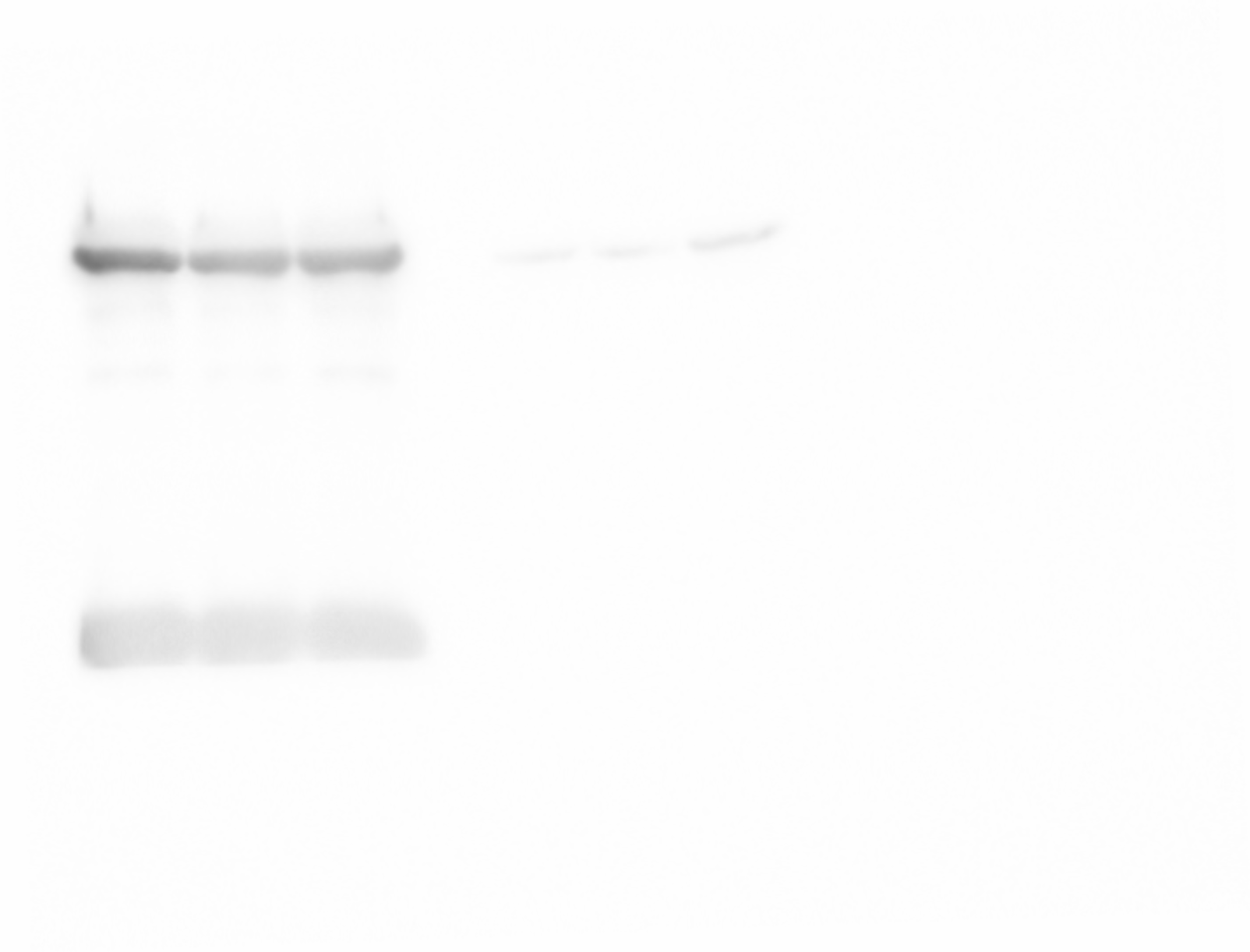

Supplement: Figure 3—figure supplement 1—source data 5. [file elife-73523-fig3-figsupp1-data5.zip › Raw blots/anti-FLAG.tif]

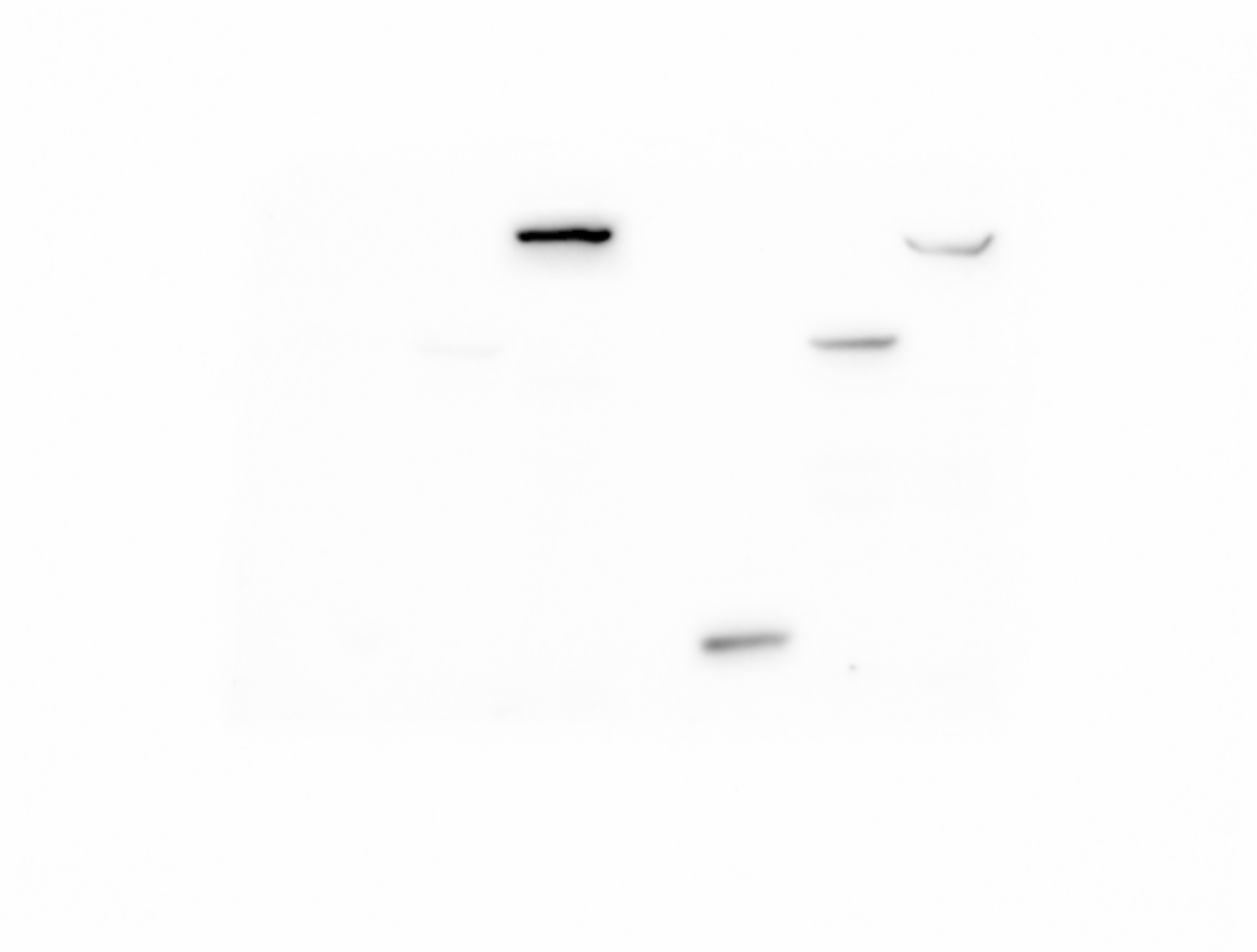

Supplement: Figure 3—figure supplement 1—source data 5. [file elife-73523-fig3-figsupp1-data5.zip › Raw blots/anti-GST.tif]

D

GST GST-Δ186-317 GST-1-385

IP :  
FLAG

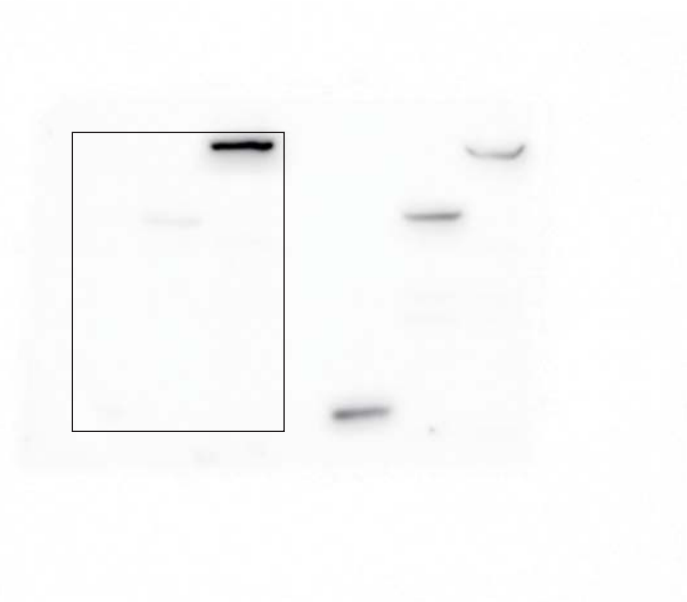

GST

IP :  
FLAG

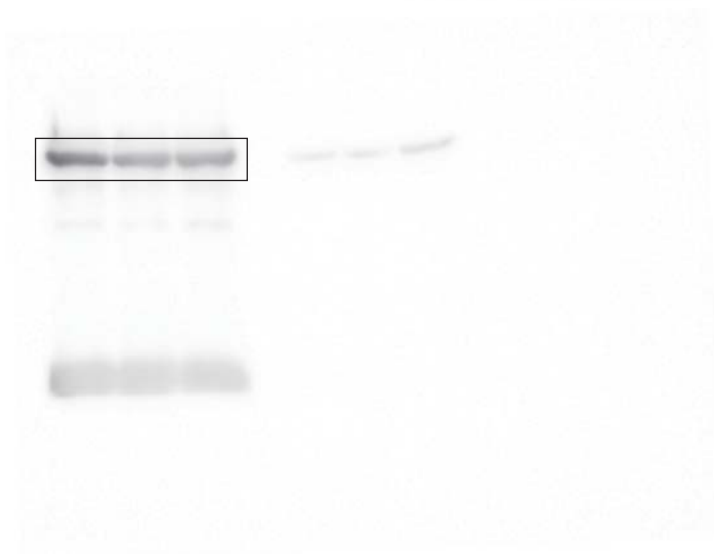

FLAG

Input

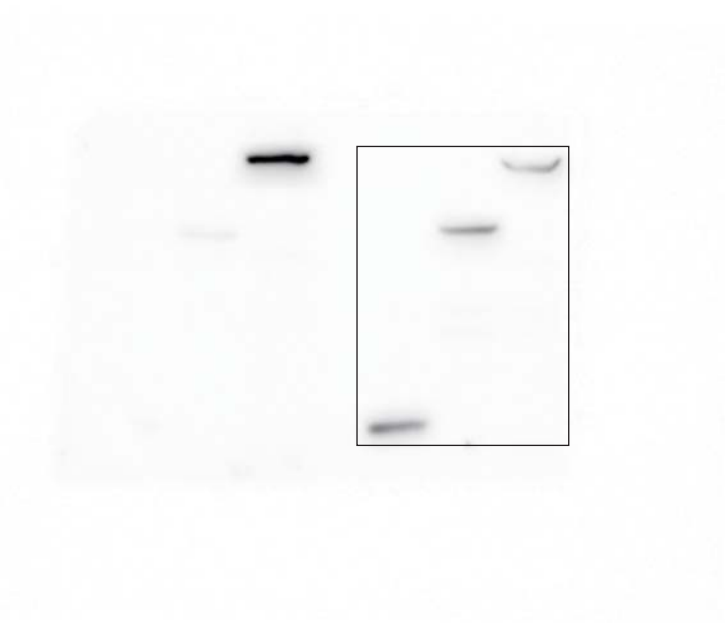

GST

Supplement: Figure 3—figure supplement 1—source data 5. [file elife-73523-fig3-figsupp1-data5.zip › Labelled blots.pdf]

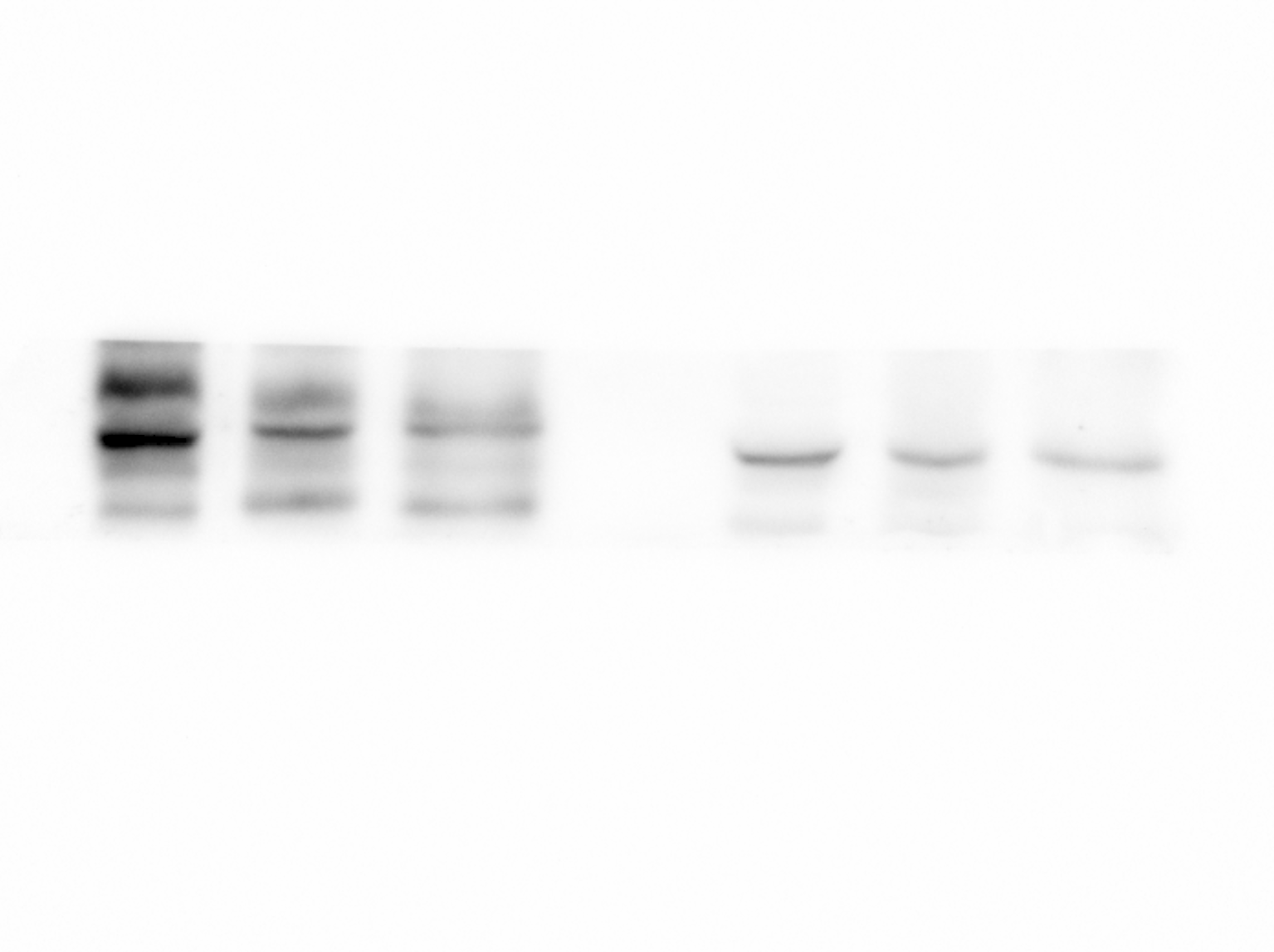

Supplement: Figure 4—figure supplement 1—source data 1. [file elife-73523-fig4-figsupp1-data1.zip › Raw blots/anti-IPMK.tif]

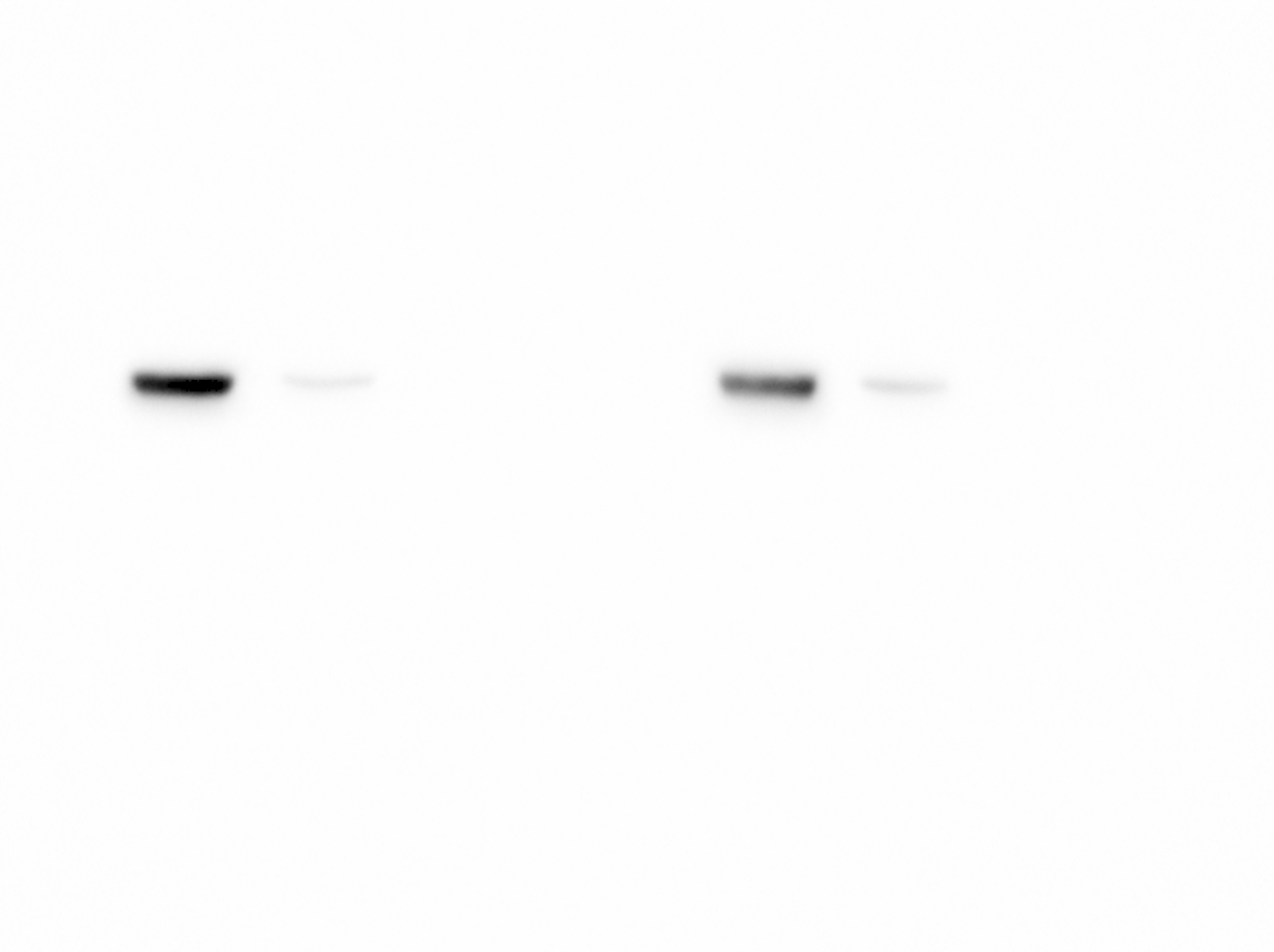

Supplement: Figure 4—figure supplement 1—source data 1. [file elife-73523-fig4-figsupp1-data1.zip › Raw blots/anti-alpha TUBULIN.tif]

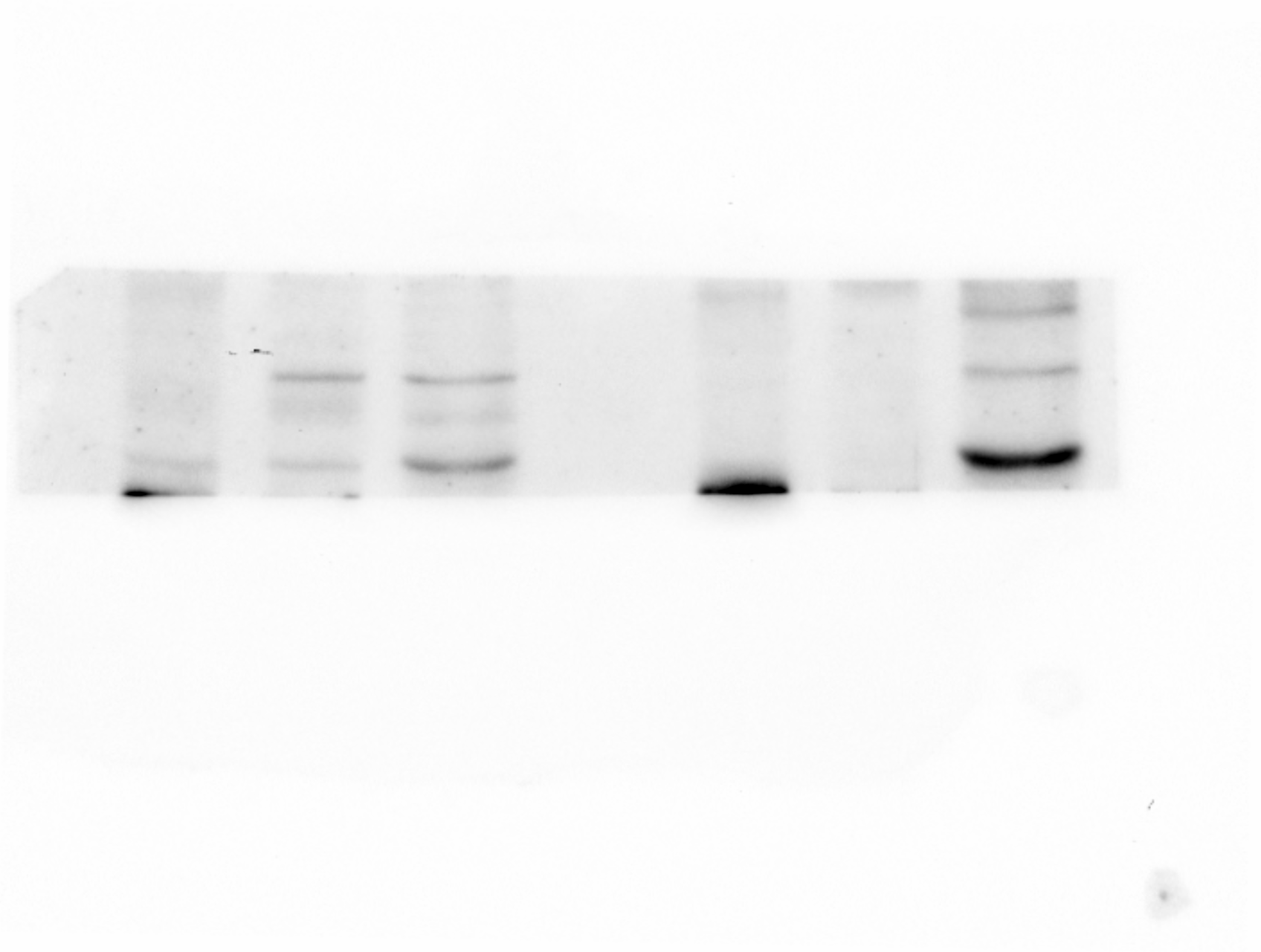

Supplement: Figure 4—figure supplement 1—source data 1. [file elife-73523-fig4-figsupp1-data1.zip › Raw blots/anti-LaminB1.tif]

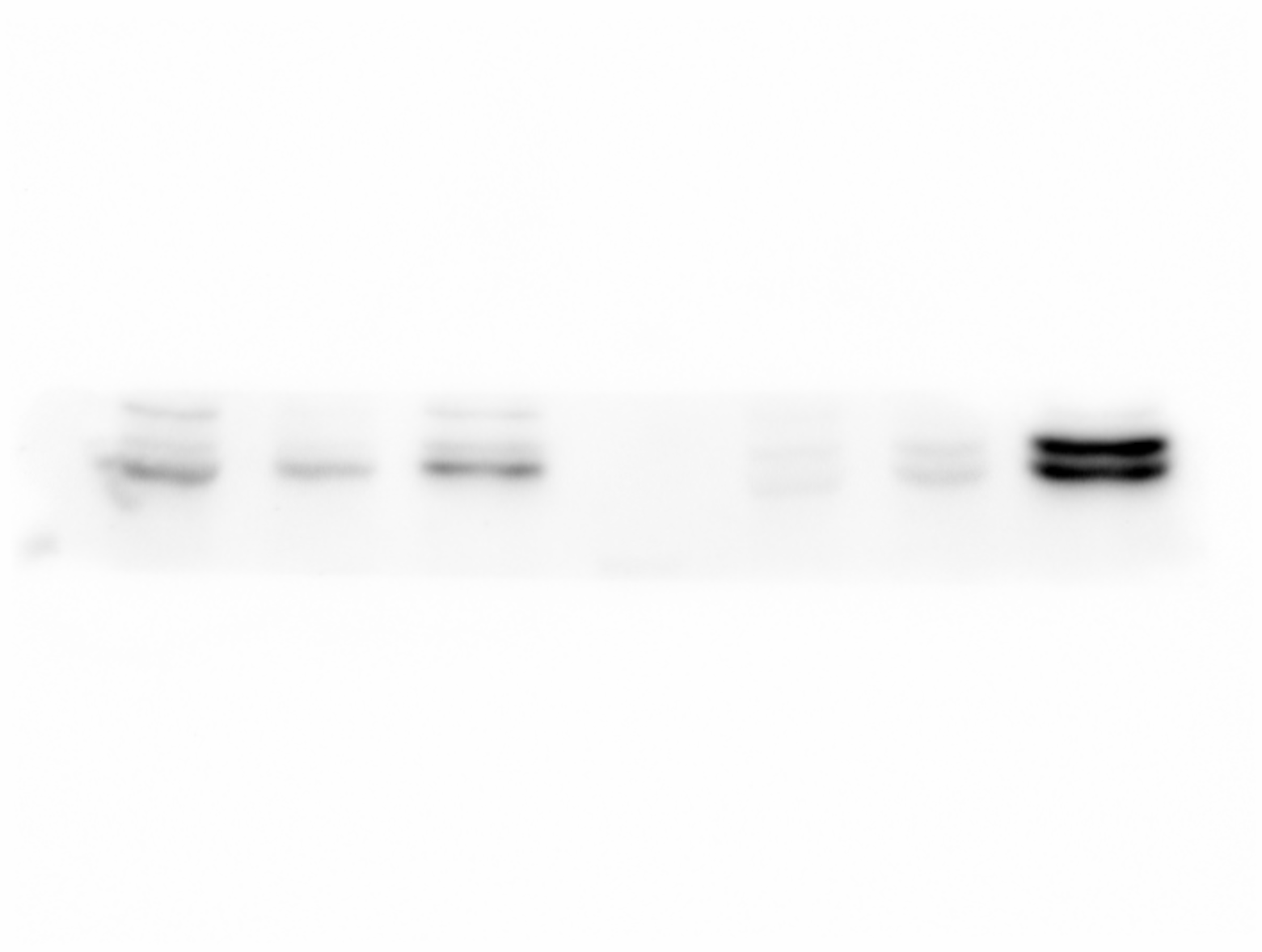

Supplement: Figure 4—figure supplement 1—source data 1. [file elife-73523-fig4-figsupp1-data1.zip › Raw blots/anti-SMARCB1.tif]

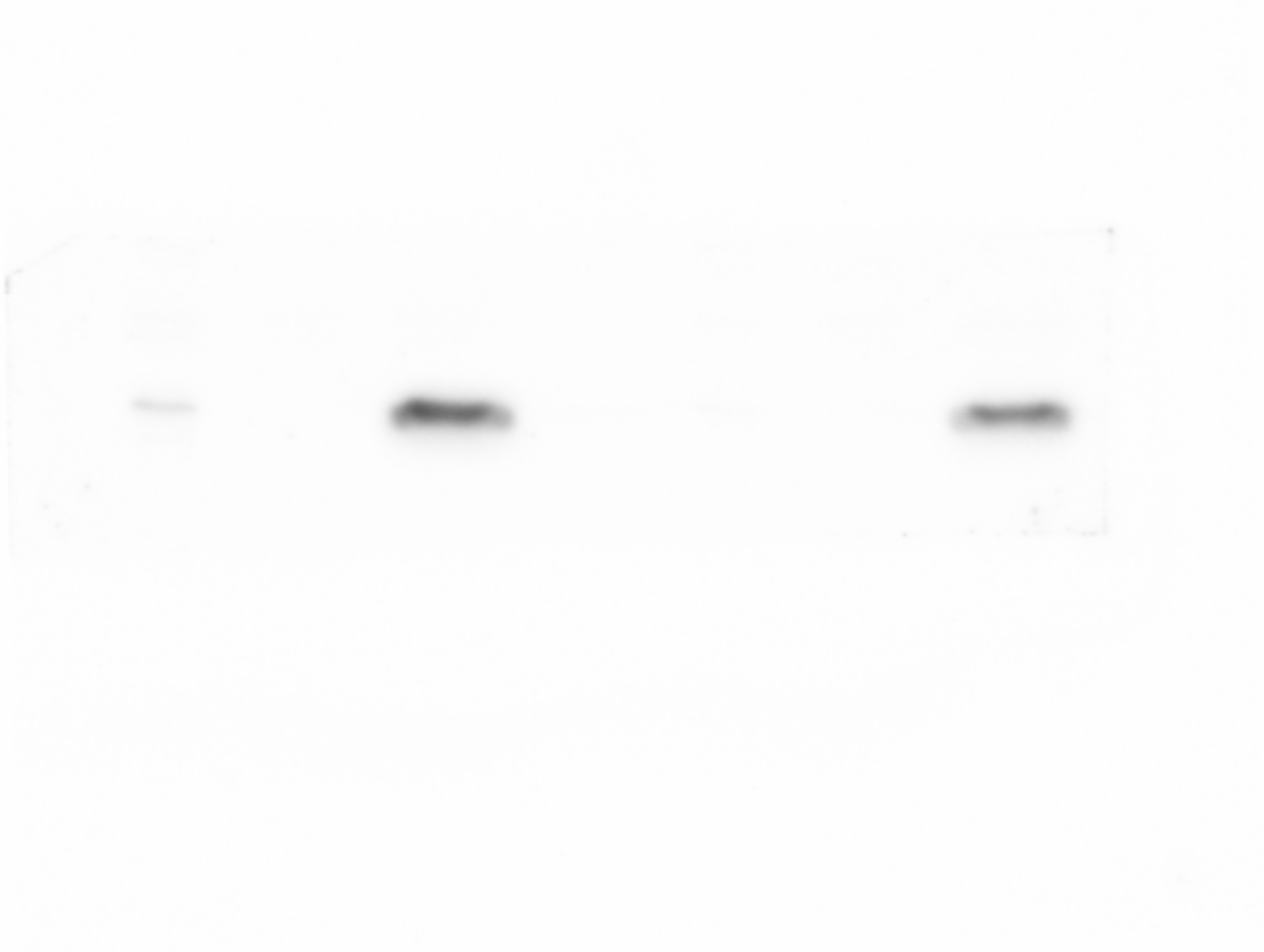

Supplement: Figure 4—figure supplement 1—source data 1. [file elife-73523-fig4-figsupp1-data1.zip › Raw blots/anti-Histone H3.tif]

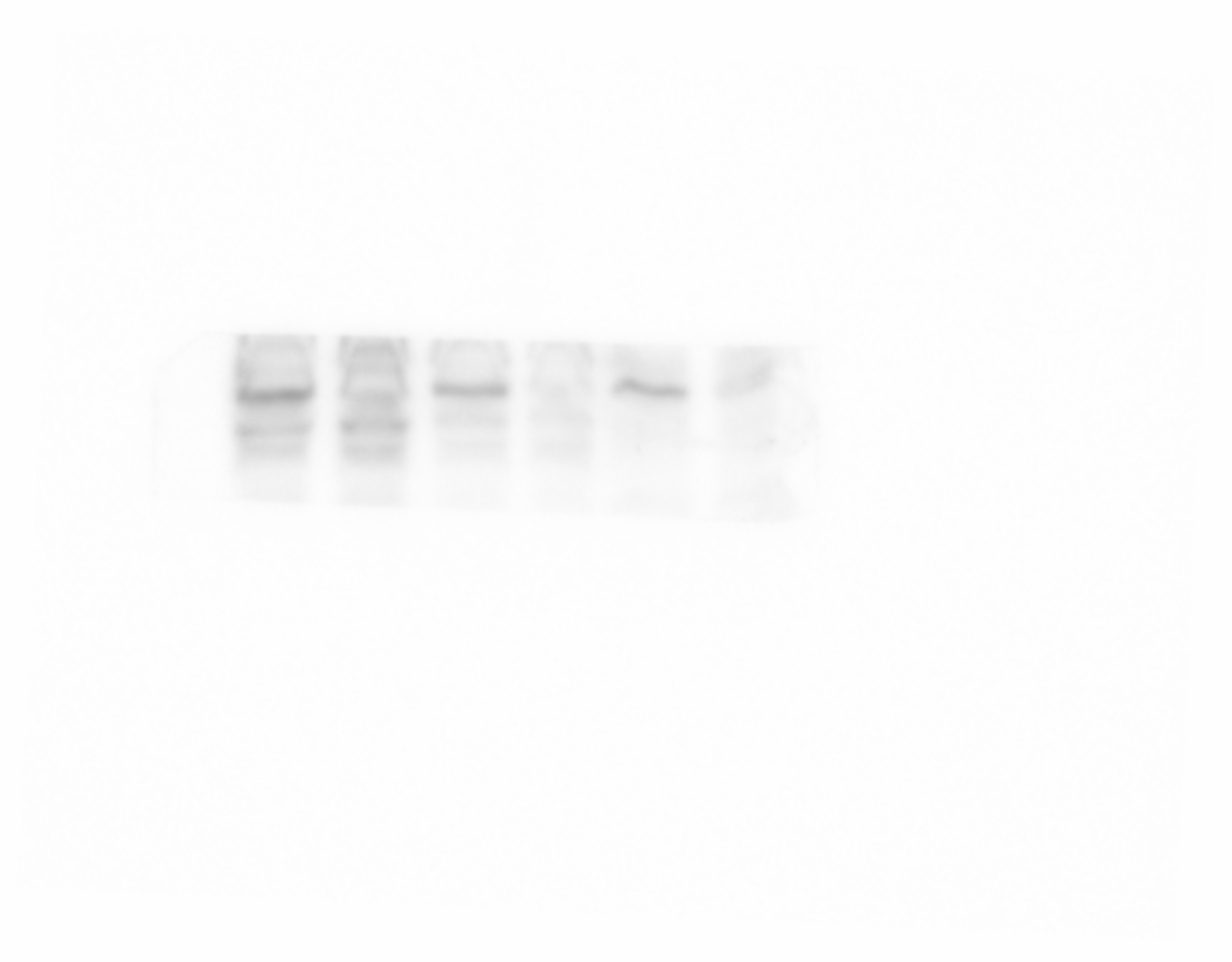

Supplement: Figure 4—figure supplement 1—source data 2. [file elife-73523-fig4-figsupp1-data2.zip › Raw blots/anti-IPMK.tif]

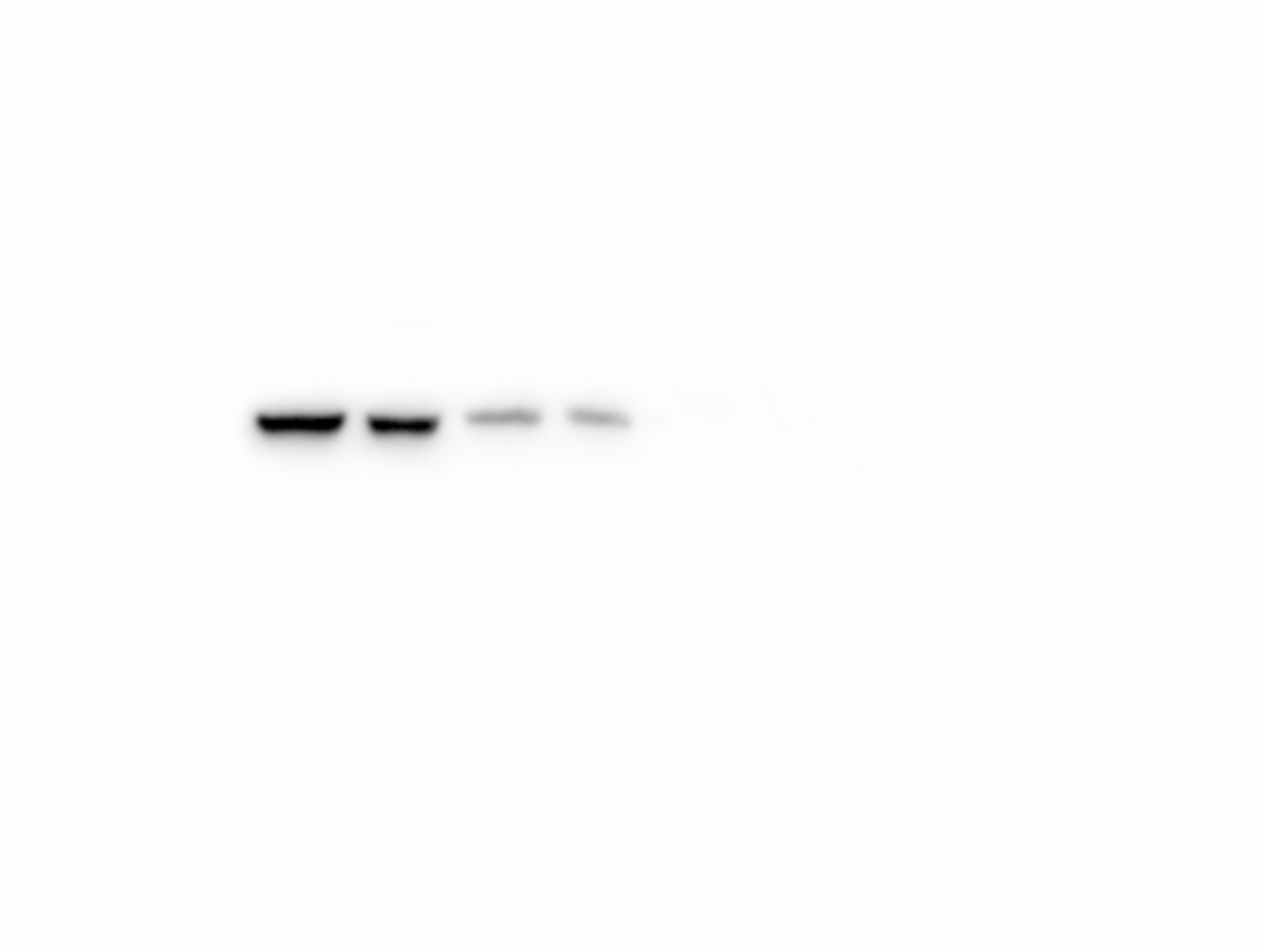

Supplement: Figure 4—figure supplement 1—source data 2. [file elife-73523-fig4-figsupp1-data2.zip › Raw blots/anti-alpha TUBULIN.tif]

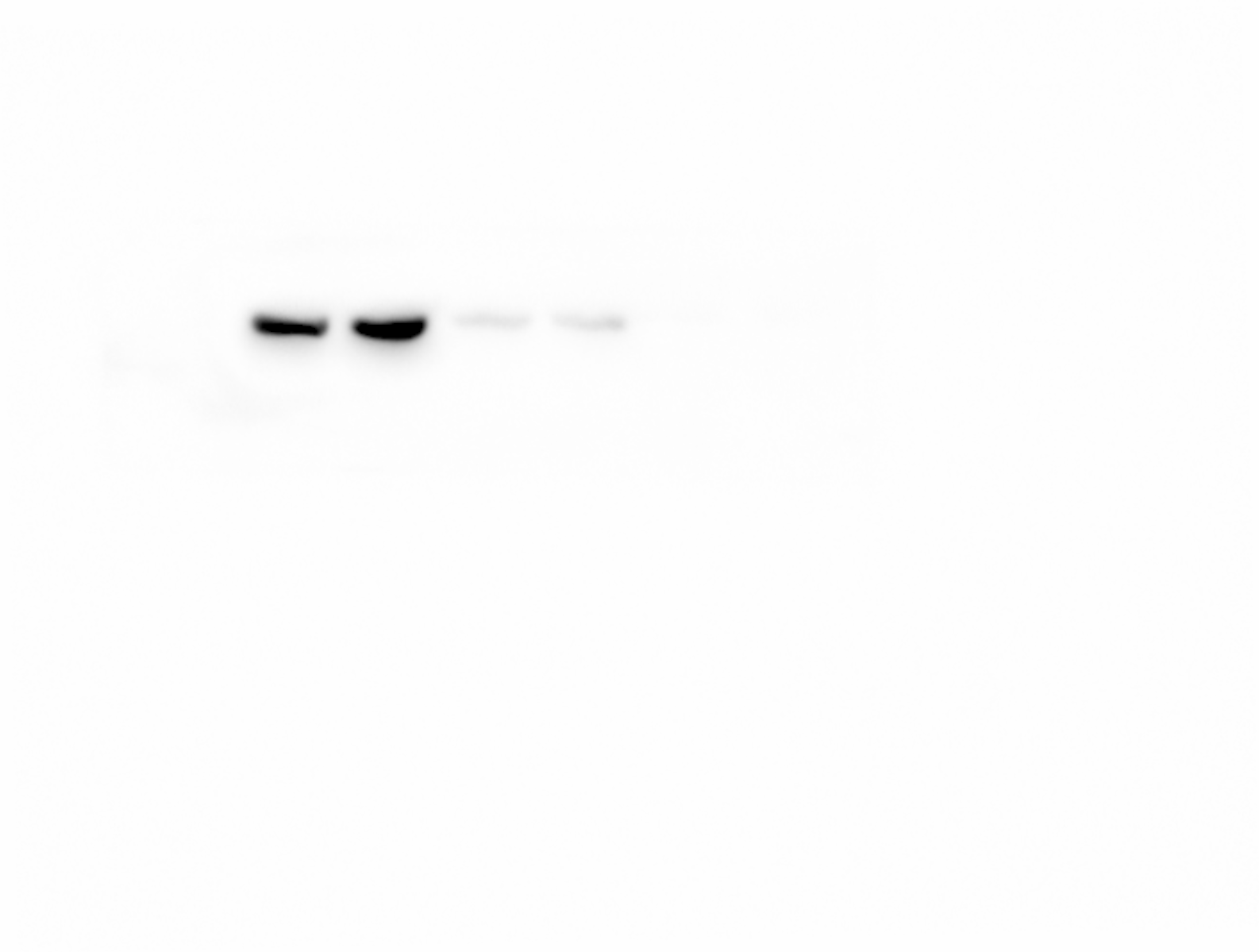

Supplement: Figure 4—figure supplement 1—source data 2. [file elife-73523-fig4-figsupp1-data2.zip › Raw blots/anti-GAPDH.tif]

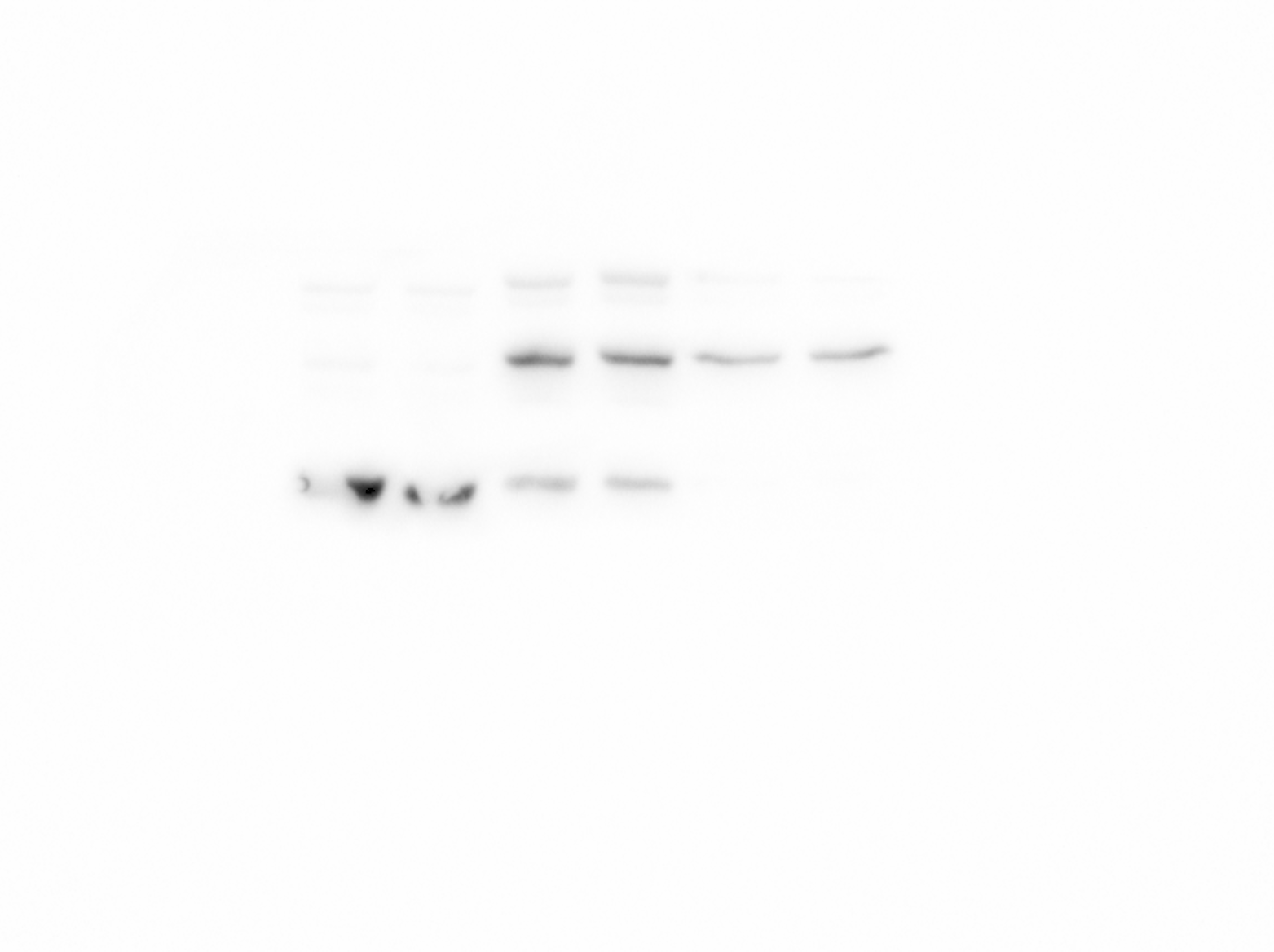

Supplement: Figure 4—figure supplement 1—source data 2. [file elife-73523-fig4-figsupp1-data2.zip › Raw blots/anti-LaminB.tif]

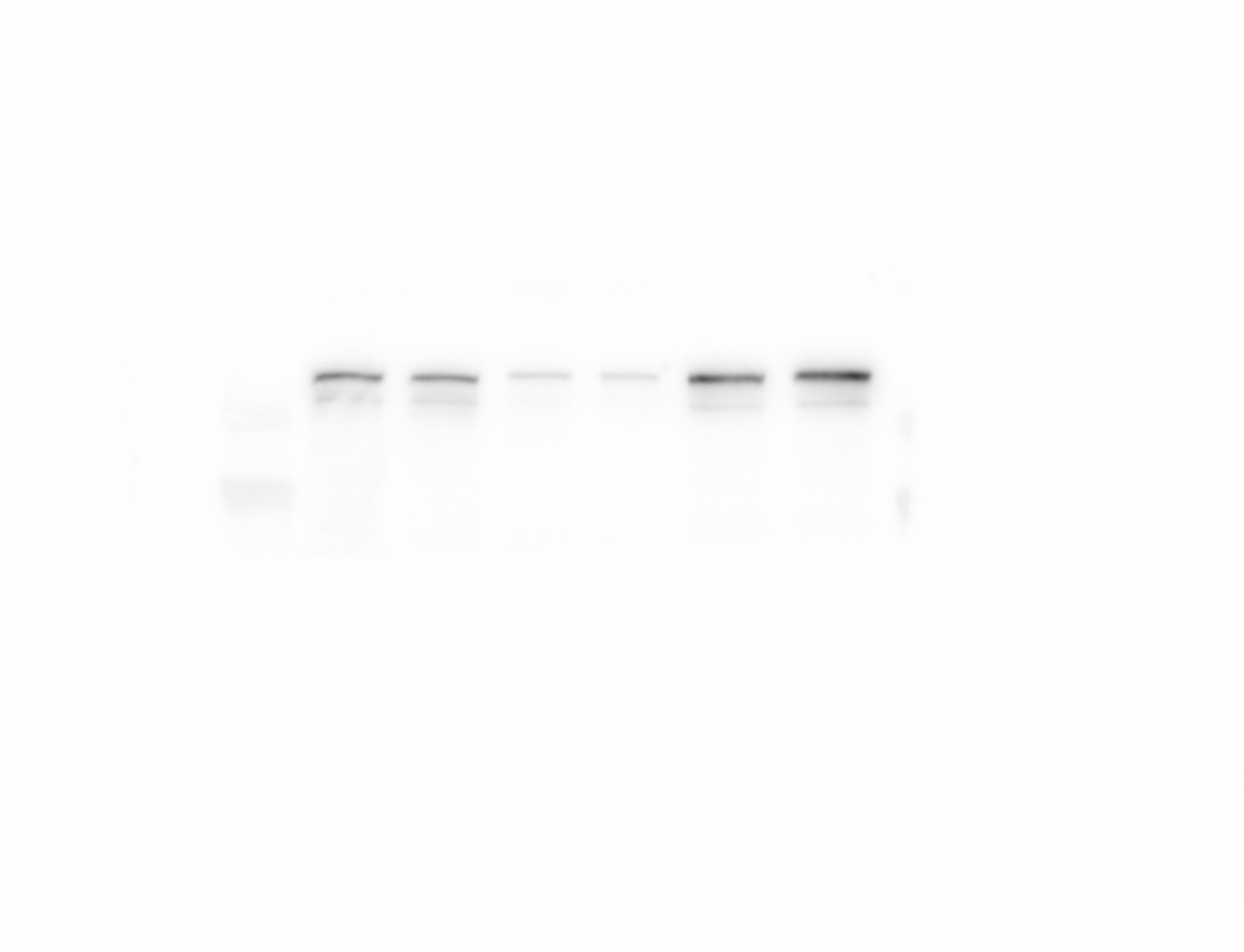

Supplement: Figure 4—figure supplement 1—source data 2. [file elife-73523-fig4-figsupp1-data2.zip › Raw blots/anti-BRG1.tif]

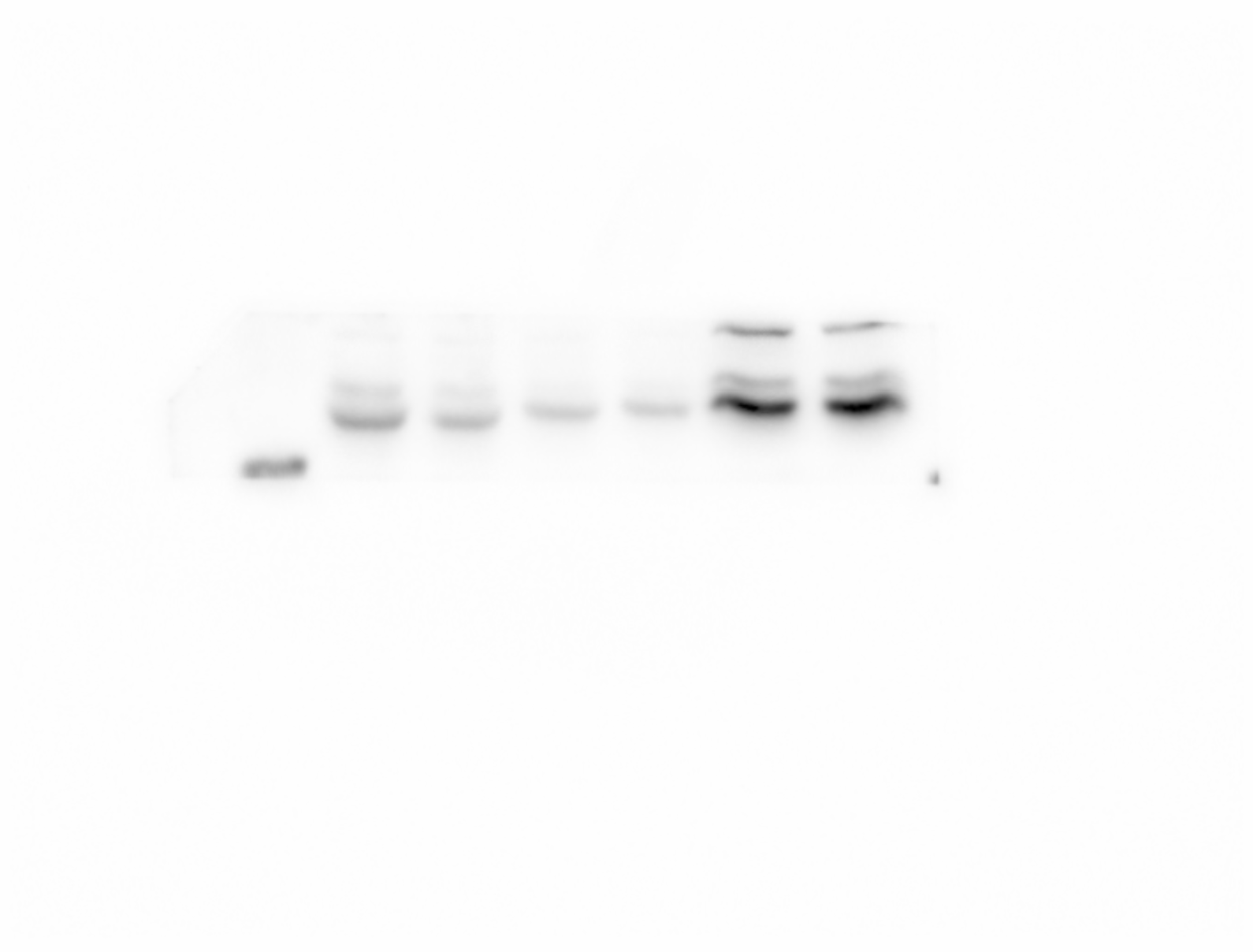

Supplement: Figure 4—figure supplement 1—source data 2. [file elife-73523-fig4-figsupp1-data2.zip › Raw blots/anti-SMARCB1.tif]

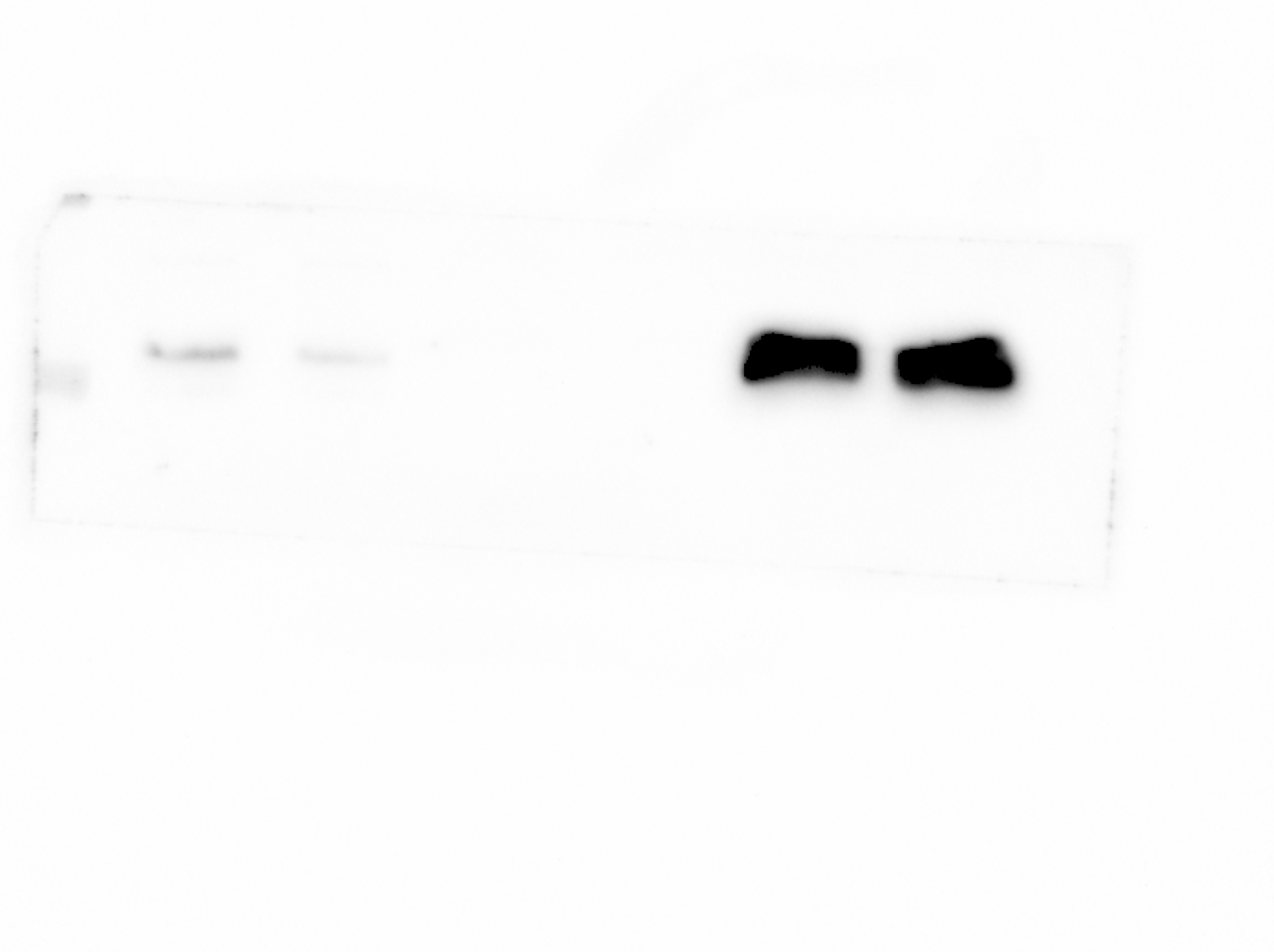

Supplement: Figure 4—figure supplement 1—source data 2. [file elife-73523-fig4-figsupp1-data2.zip › Raw blots/anti-Histone H3.tif]

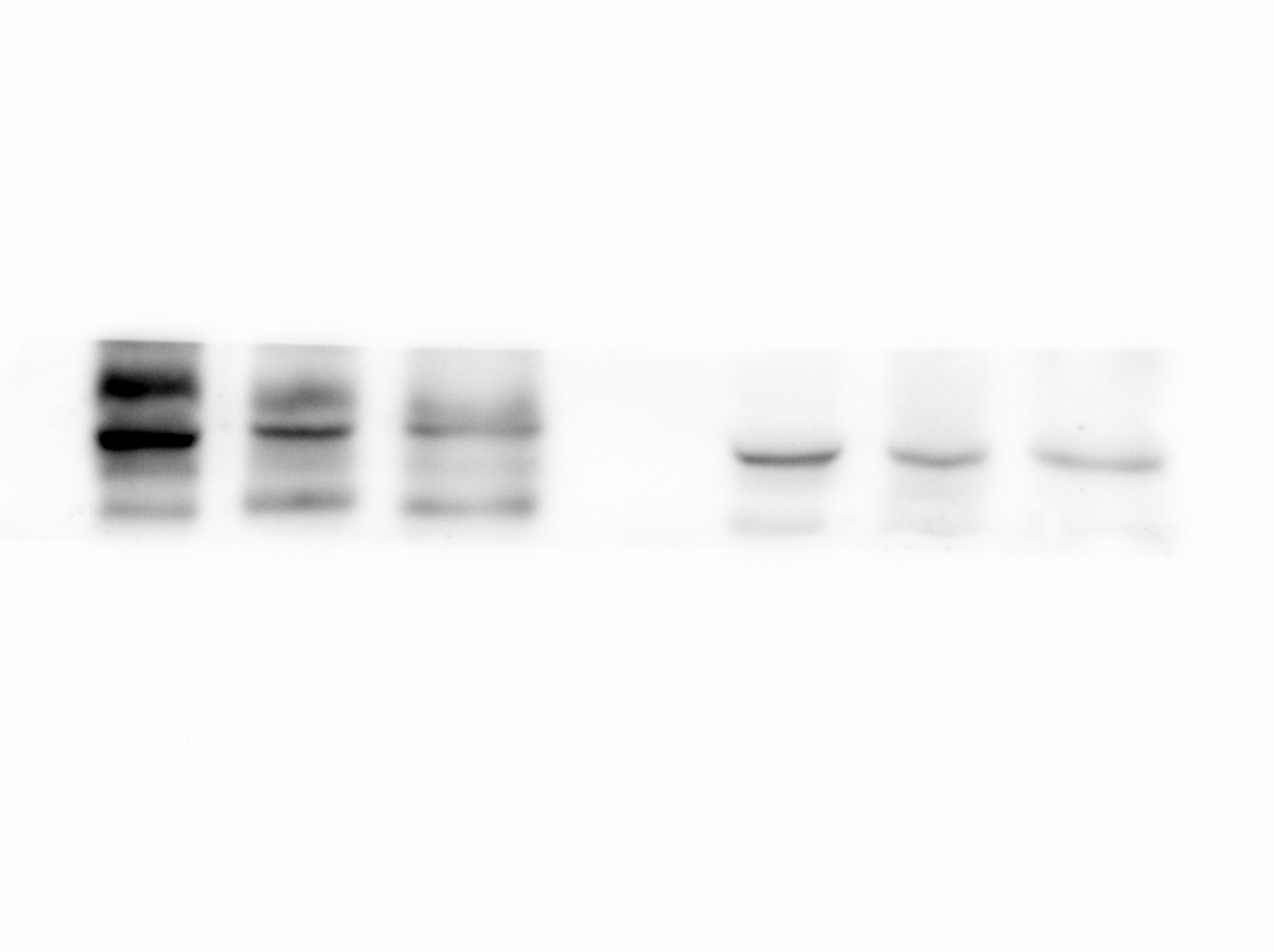

Supplement: Figure 4—figure supplement 1—source data 3. [file elife-73523-fig4-figsupp1-data3.zip › Raw blots/anti-IPMK.tif]

Cytoplasm  
Nucleoplasm  
Chromatin

IPMK

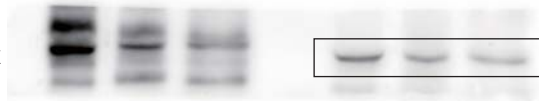

SMARCB1

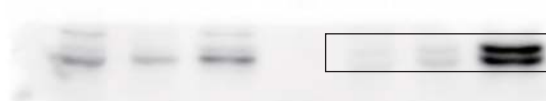

LaminB1

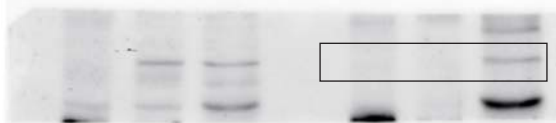

$\alpha$ -TUBULIN

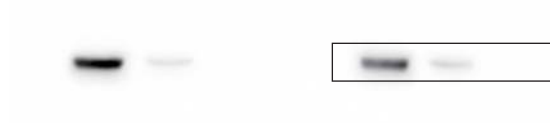

Histone H3

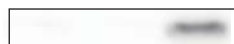

Supplement: Figure 4—figure supplement 1—source data 3. [file elife-73523-fig4-figsupp1-data3.zip › Labelled blots.pdf]

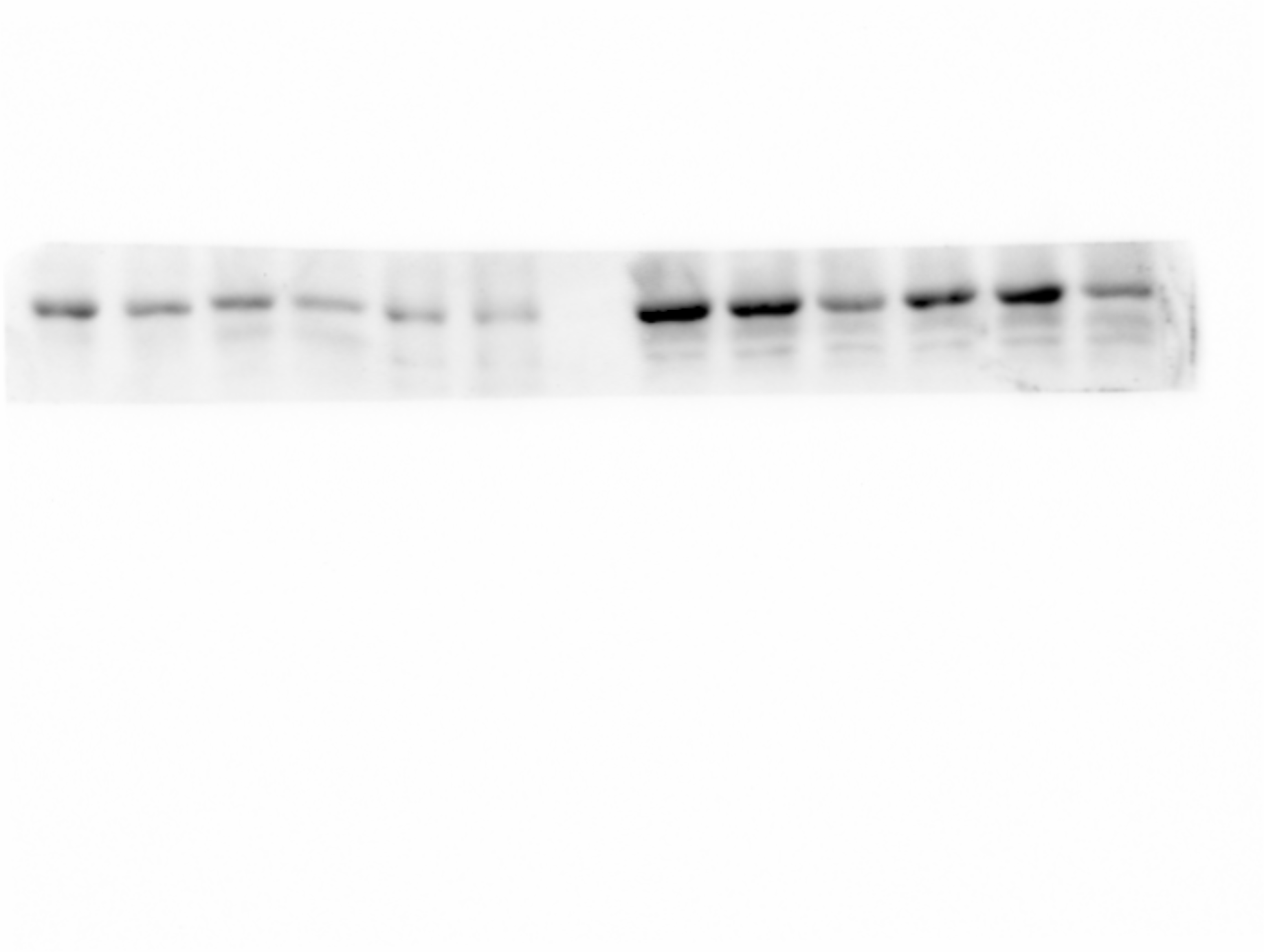

Supplement: Figure 4—figure supplement 1—source data 4. [file elife-73523-fig4-figsupp1-data4.zip › Raw blots/anti-IPMK.tif]

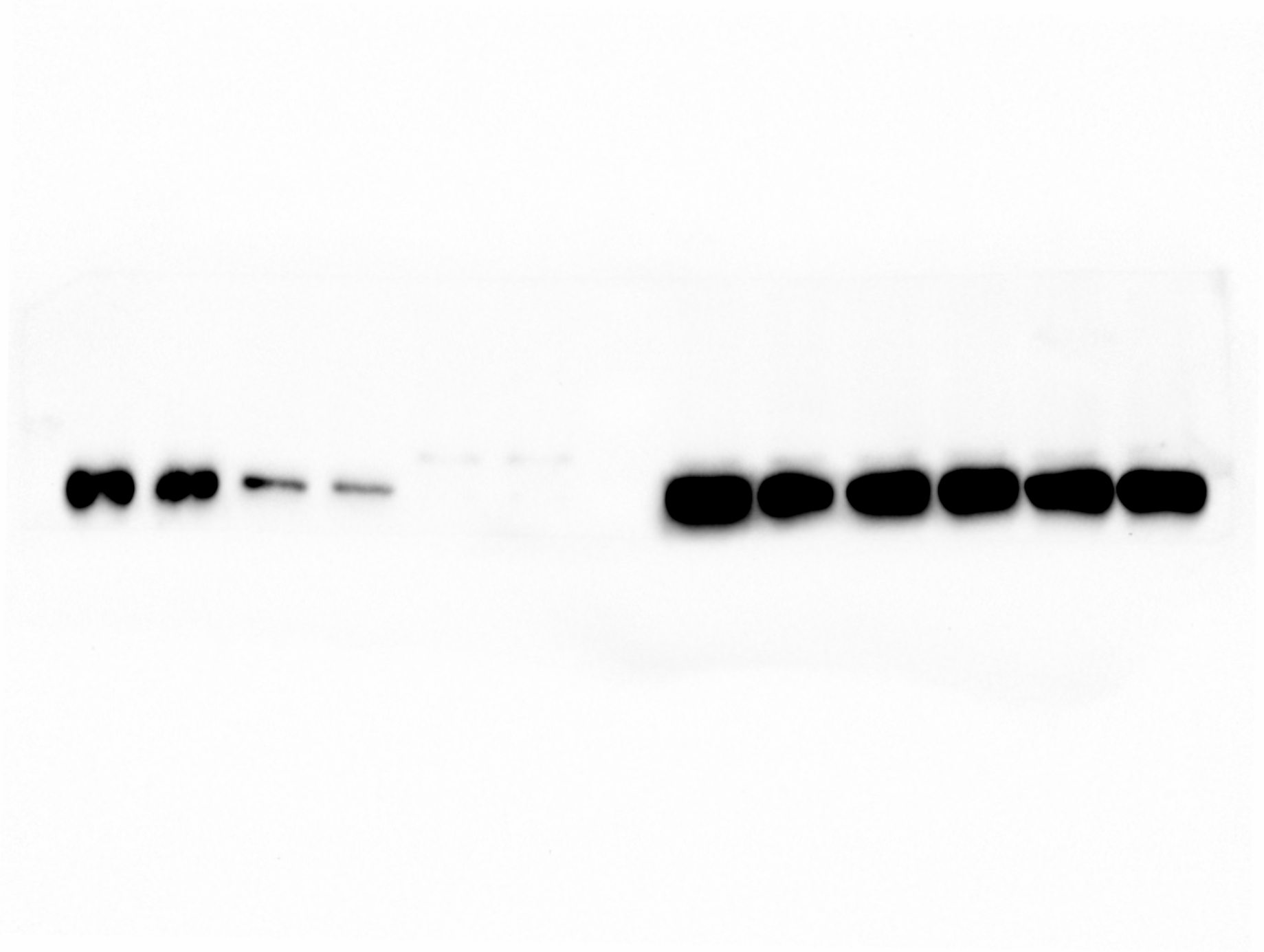

Supplement: Figure 4—figure supplement 1—source data 4. [file elife-73523-fig4-figsupp1-data4.zip › Raw blots/anti-alpha TUBULIN.tif]

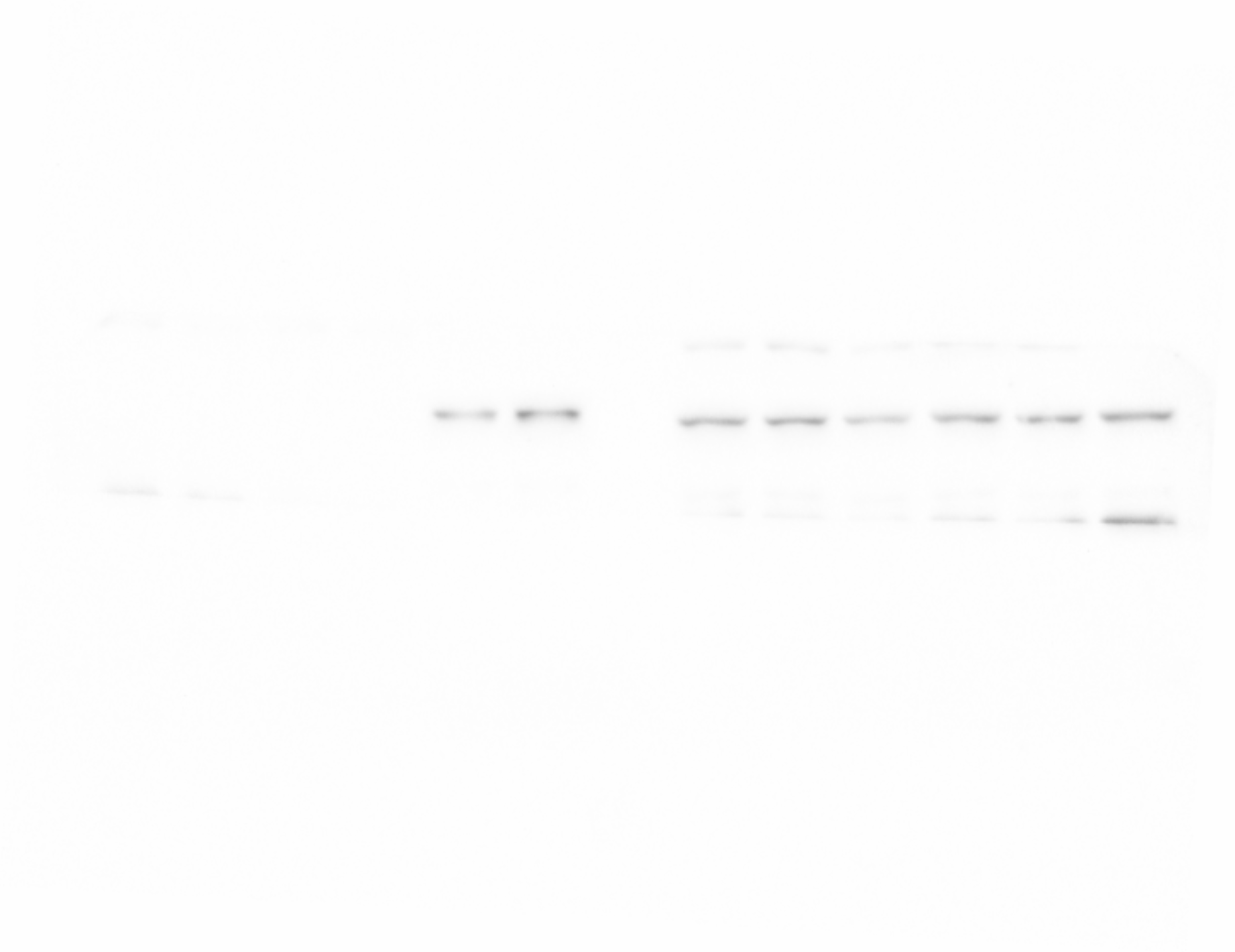

Supplement: Figure 4—figure supplement 1—source data 4. [file elife-73523-fig4-figsupp1-data4.zip › Raw blots/anti-LaminB1.tif]

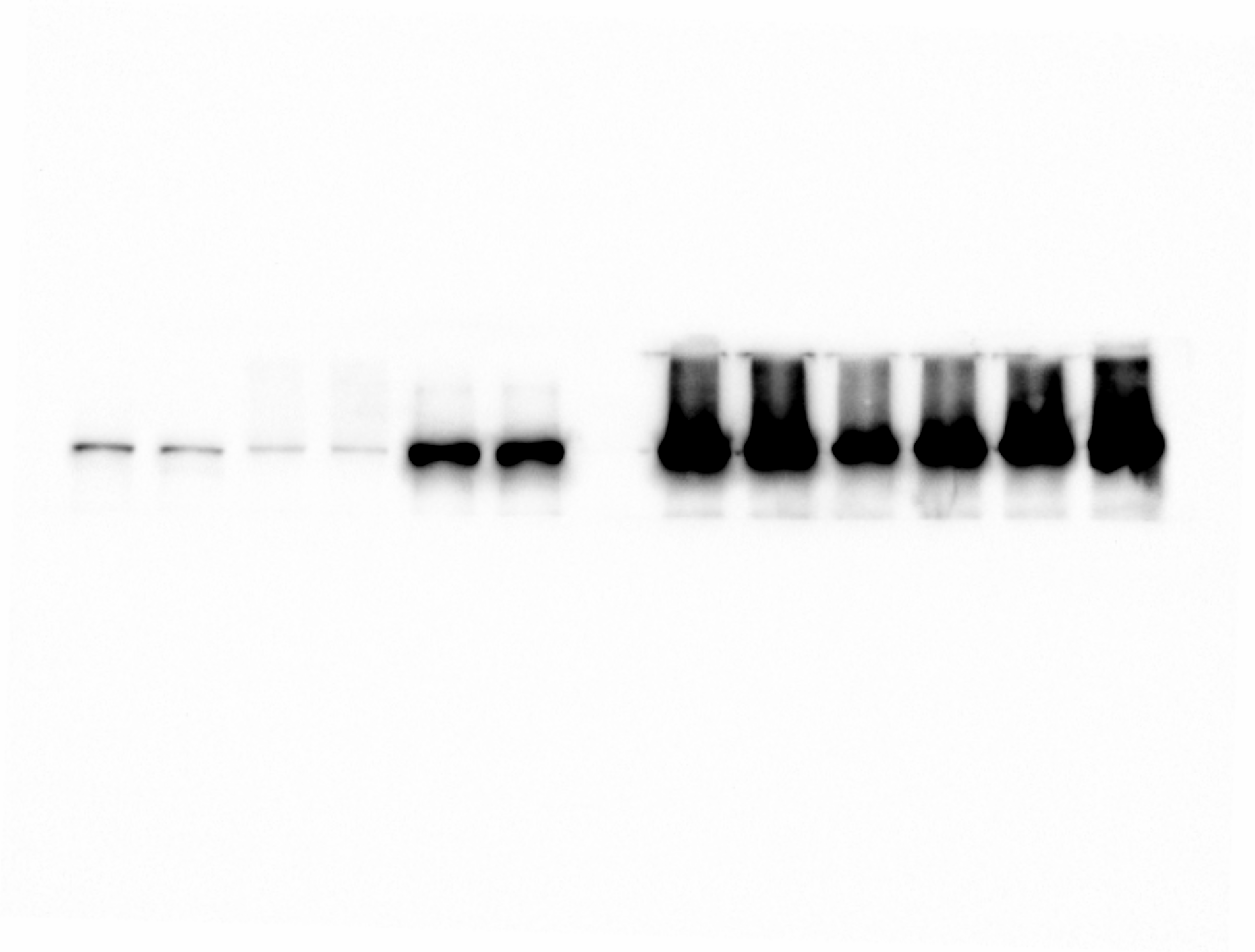

Supplement: Figure 4—figure supplement 1—source data 4. [file elife-73523-fig4-figsupp1-data4.zip › Raw blots/anti-BRG1.tif]

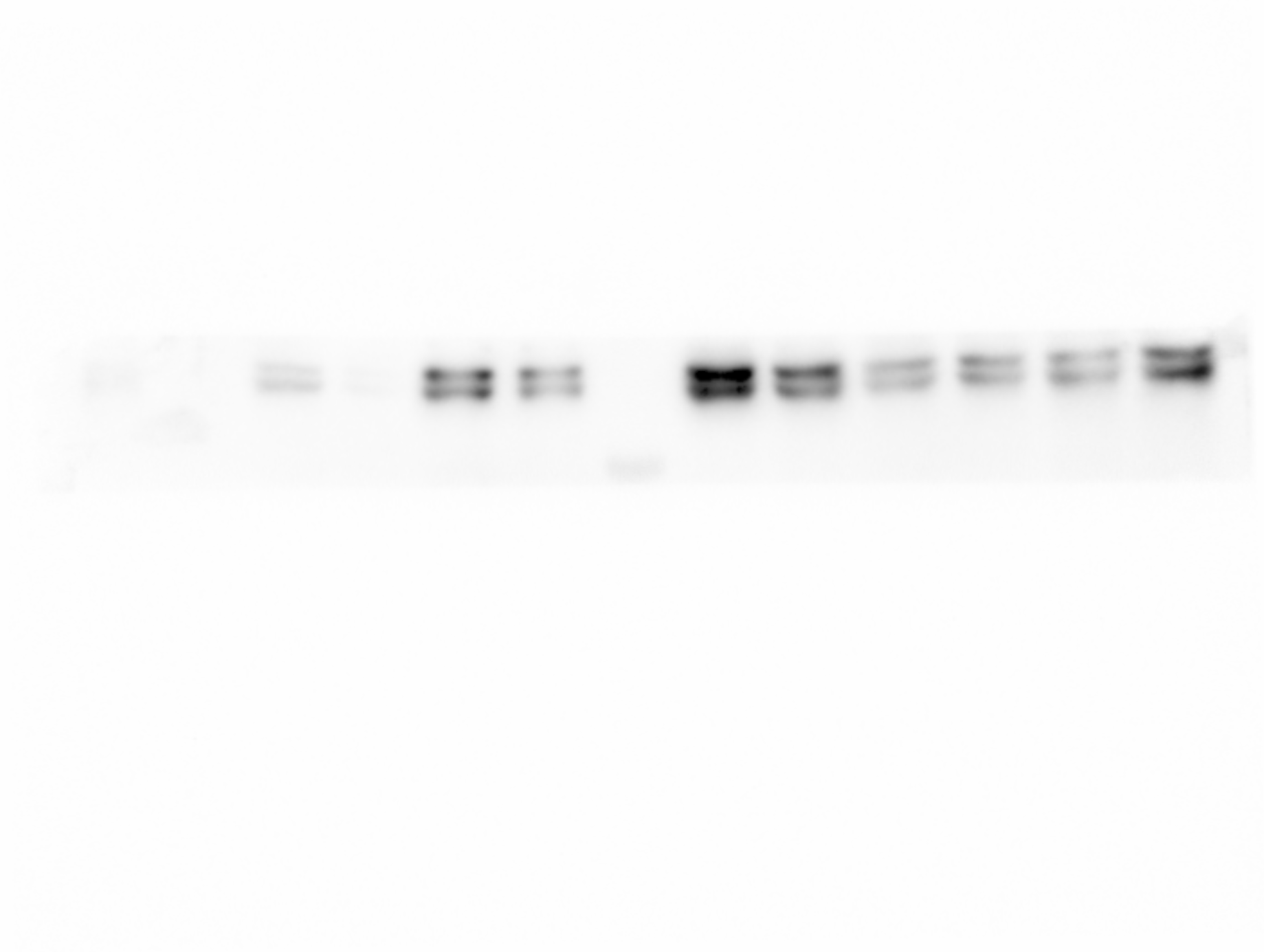

Supplement: Figure 4—figure supplement 1—source data 4. [file elife-73523-fig4-figsupp1-data4.zip › Raw blots/anti-SMARCB1.tif]

**A**

|  | Cytoplasm |           | Nucleoplasm |           | Chromatin |           |
|--|-----------|-----------|-------------|-----------|-----------|-----------|
|  | siEgfp    | siSmardb1 | siEgfp      | siSmardb1 | siEgfp    | siSmardb1 |

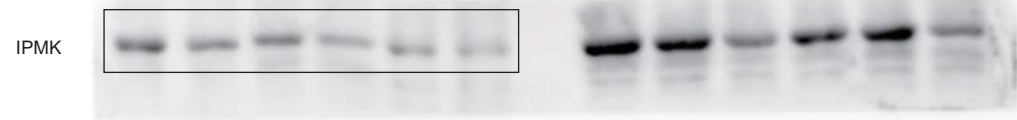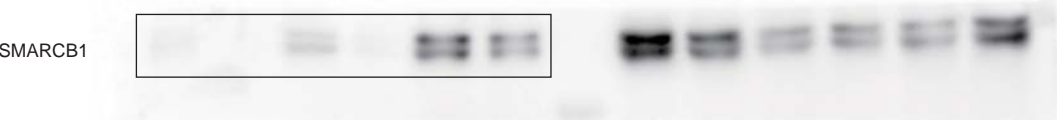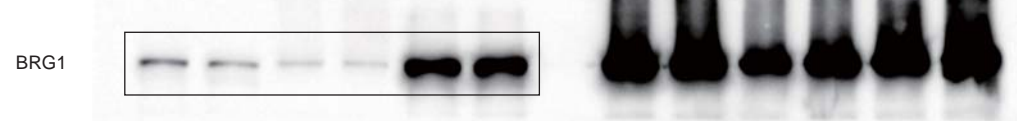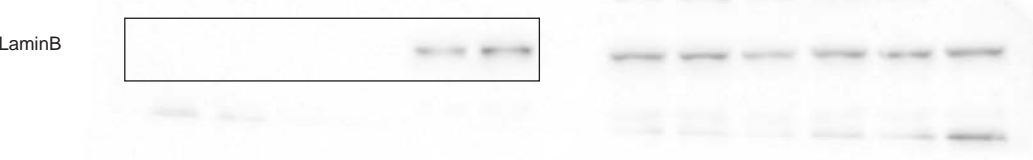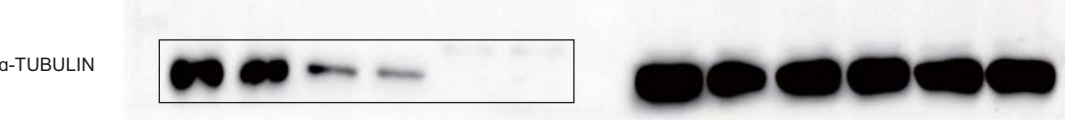

Supplement: Figure 4—figure supplement 1—source data 4. [file elife-73523-fig4-figsupp1-data4.zip › Labelled blots.pdf]

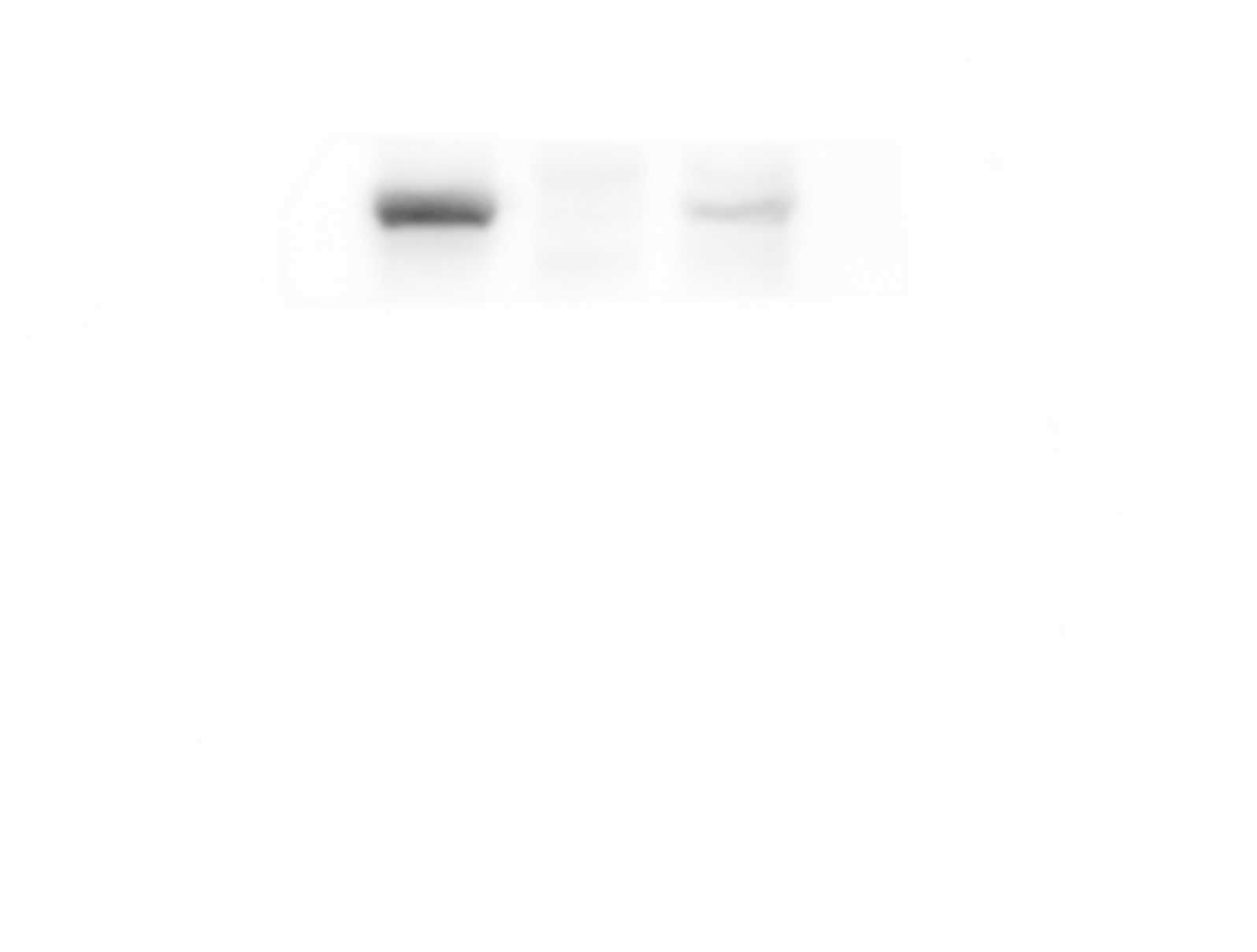

Supplement: Figure 4—figure supplement 2—source data 1. [file elife-73523-fig4-figsupp2-data1.zip › Raw blots/anti-IPMK.tif]

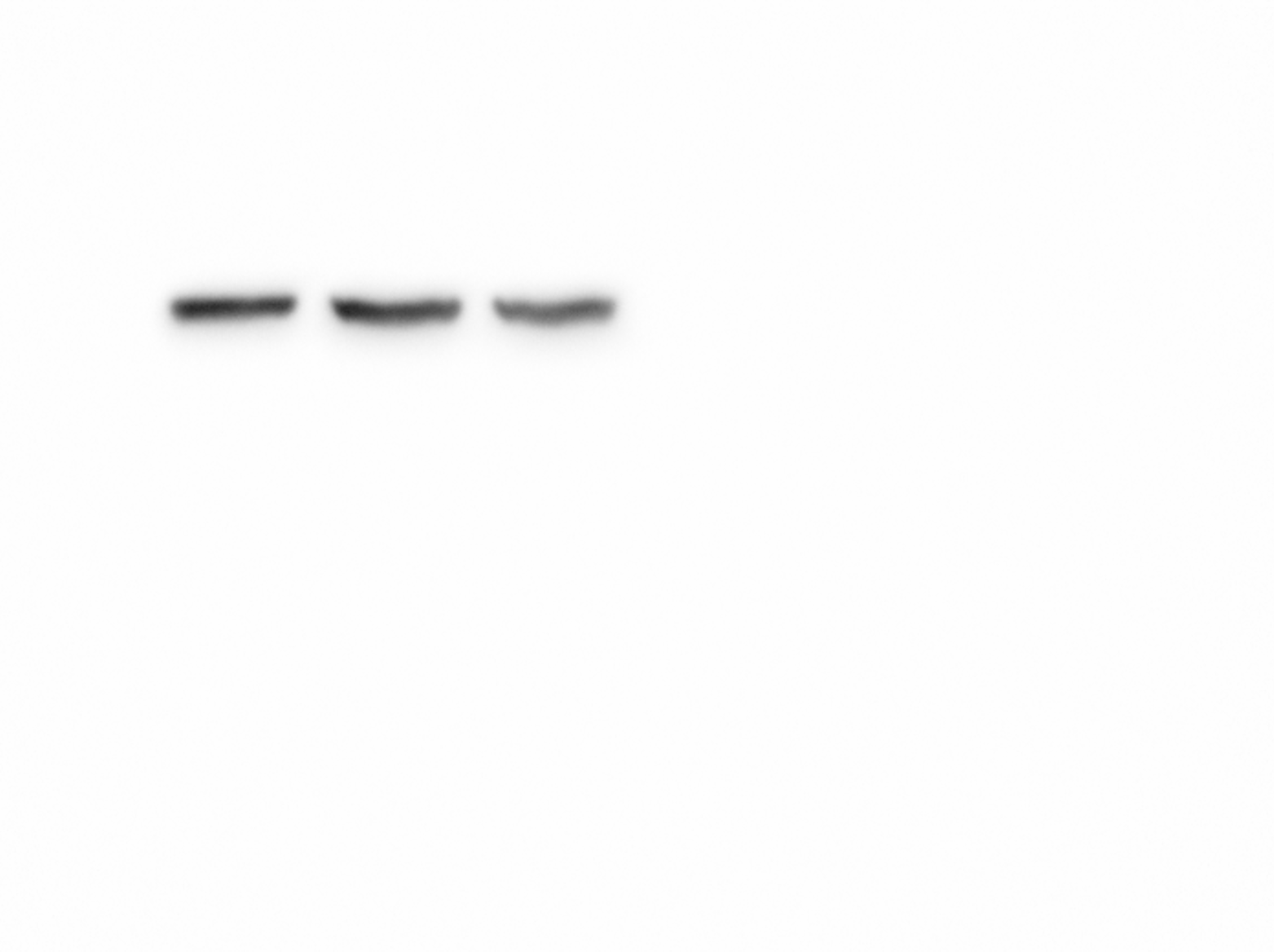

Supplement: Figure 4—figure supplement 2—source data 1. [file elife-73523-fig4-figsupp2-data1.zip › Raw blots/anti-alpha TUBULIN.tif]

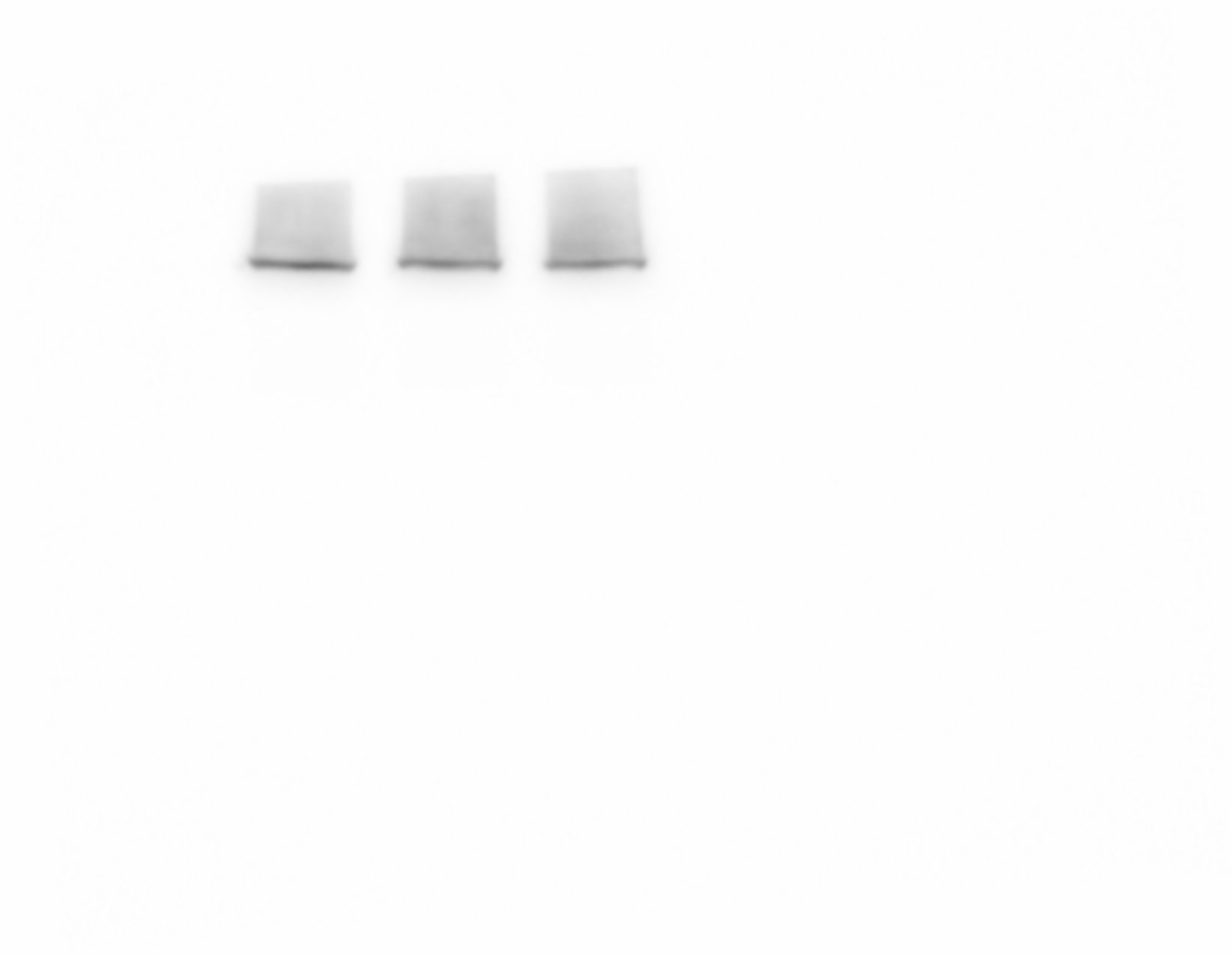

Supplement: Figure 4—figure supplement 2—source data 1. [file elife-73523-fig4-figsupp2-data1.zip › Raw blots/anti-BRG1.tif]

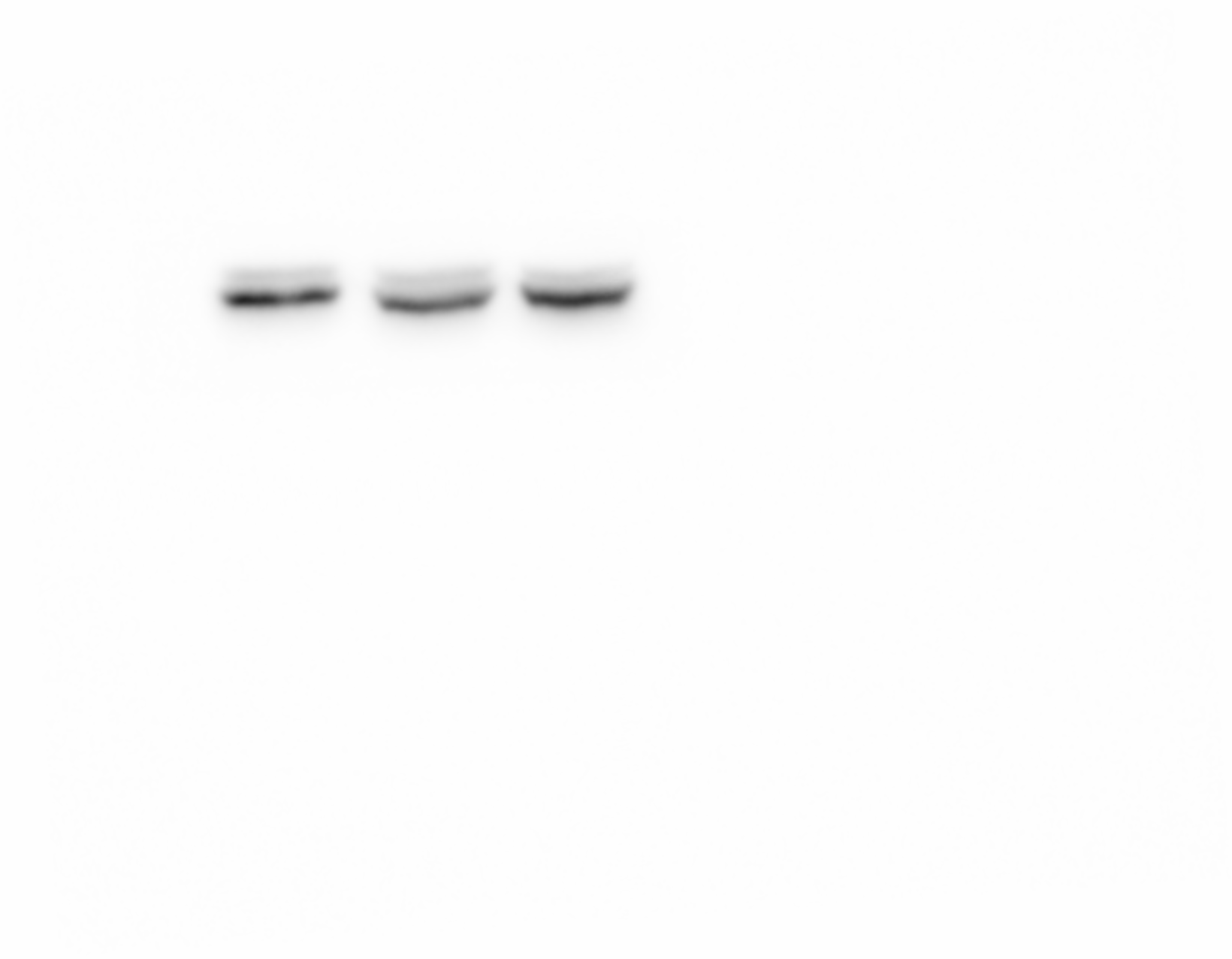

Supplement: Figure 4—figure supplement 2—source data 1. [file elife-73523-fig4-figsupp2-data1.zip › Raw blots/anti-SMARCB1.tif]

shNT shipmk1 shipmk2

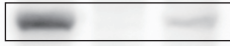

IPMK

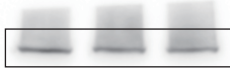

BRG1

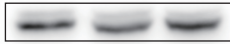

SMARCB1

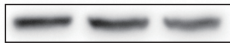

$\alpha$ -TUBULIN

Supplement: Figure 4—figure supplement 2—source data 1. [file elife-73523-fig4-figsupp2-data1.zip › Labelled blots.pdf]

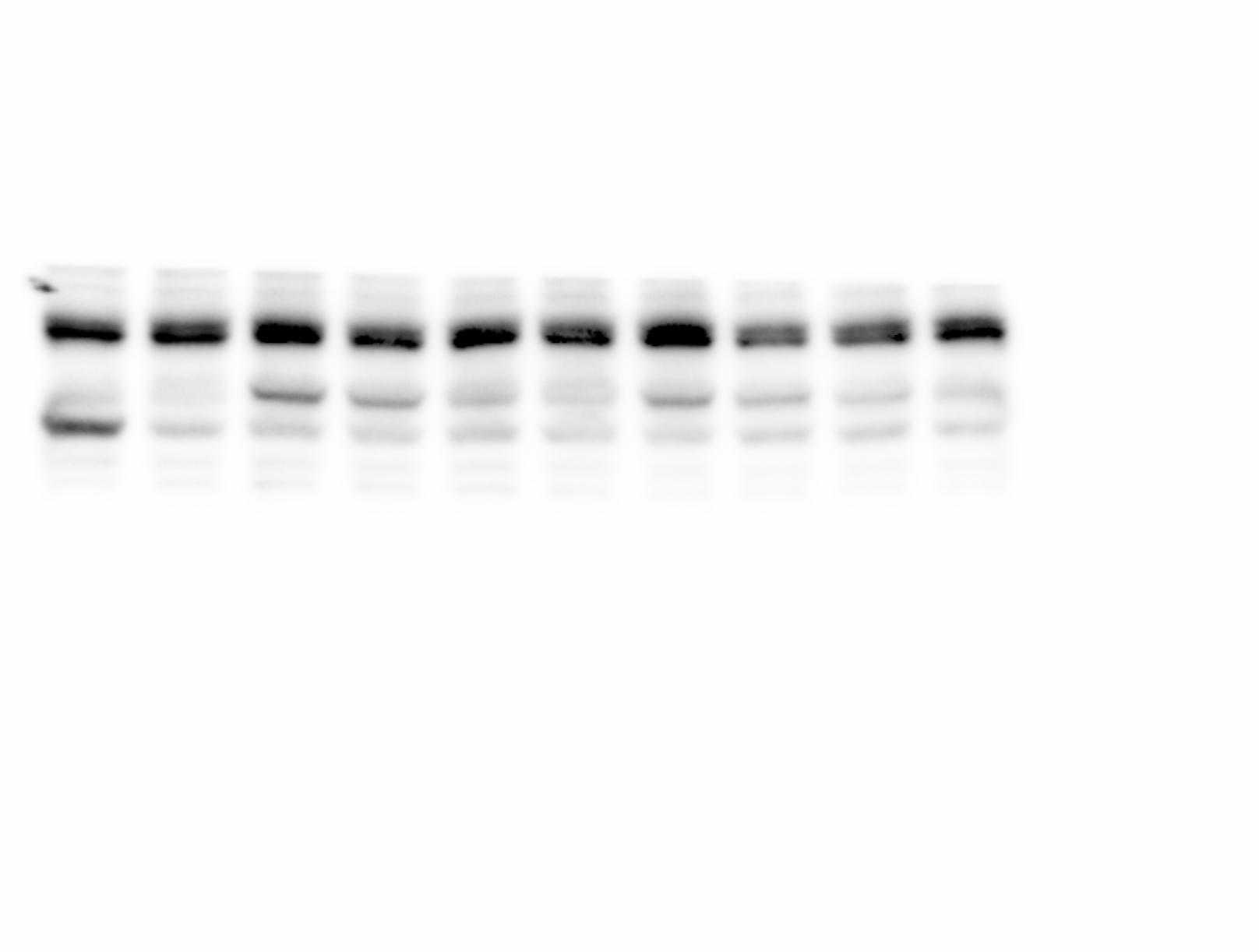

Supplement: Figure 4—figure supplement 2—source data 2. [file elife-73523-fig4-figsupp2-data2.zip › Raw blots/anti-IPMK.tif]

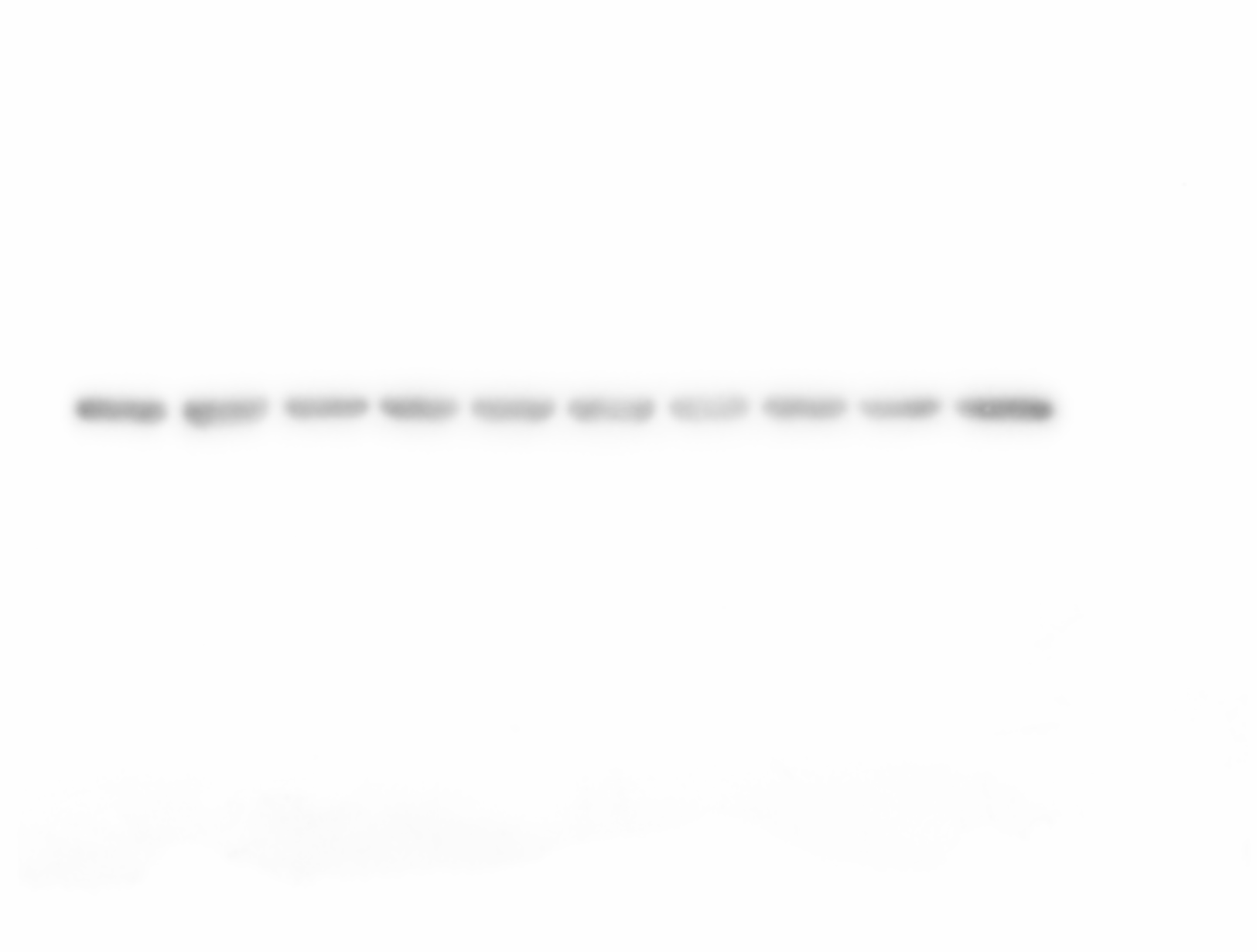

Supplement: Figure 4—figure supplement 2—source data 2. [file elife-73523-fig4-figsupp2-data2.zip › Raw blots/anti-GAPDH.tif]
