# Supplementary material for: Inositol polyphosphate multikinase physically binds to the SWI/SNF complex and modulates BRG1 occupancy in mouse embryonic stem cells
Source: eLife. 2022 May 12;11:e73523. doi: 10.7554/eLife.73523 (PMC9098221; doi:10.7554/eLife.73523)
Supplement: Figure 3—source data 2. [file elife-73523-fig3-data2.zip › Labelled blots.pdf]

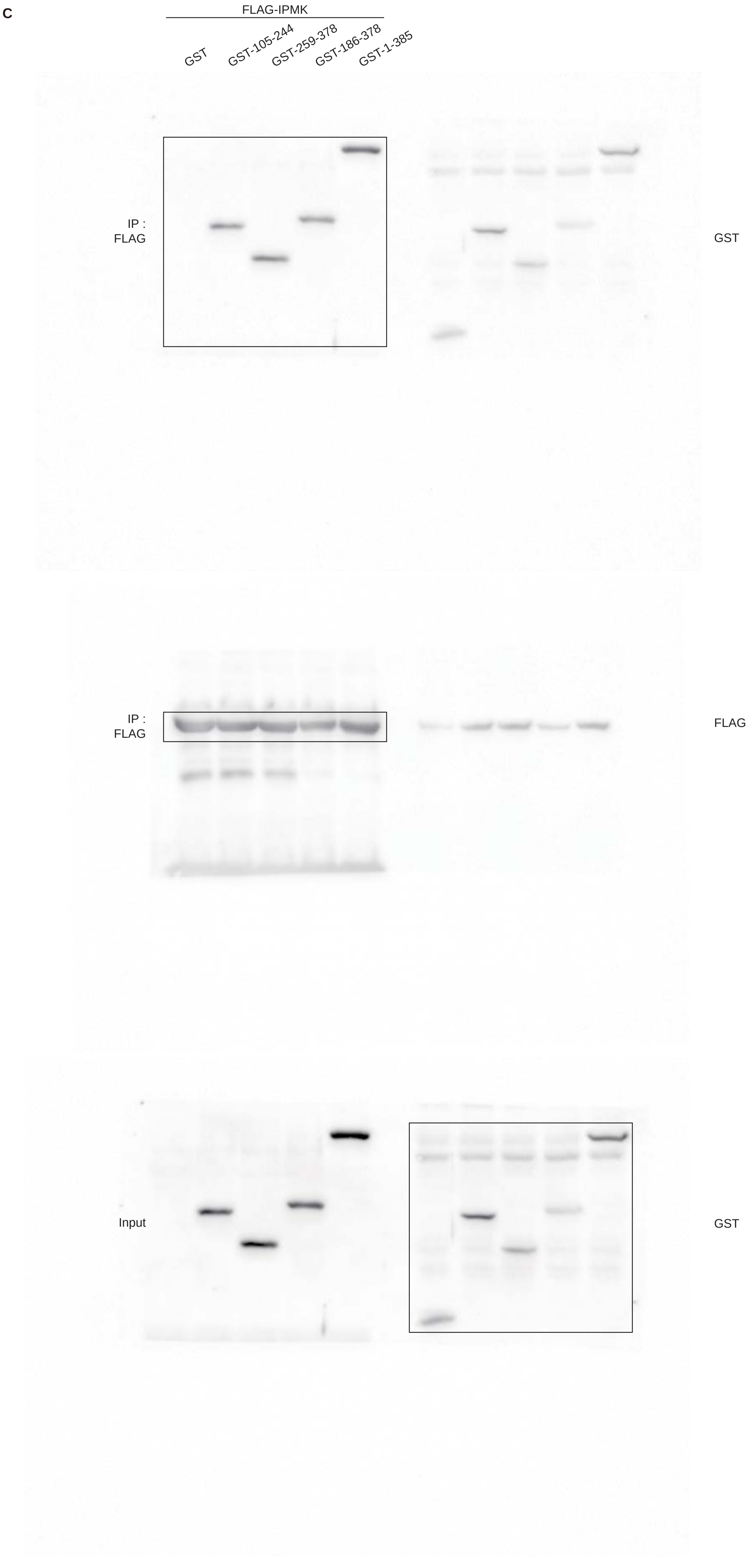
C
FLAG-IPMK
GST
GST-105-244
GST-259-378
GST-186-378
GST-1-385
IP :
FLAG
GST
IP :
FLAG
FLAG
Input
GST
