# Supplementary material for: Inositol polyphosphate multikinase physically binds to the SWI/SNF complex and modulates BRG1 occupancy in mouse embryonic stem cells
Source: eLife. 2022 May 12;11:e73523. doi: 10.7554/eLife.73523 (PMC9098221; doi:10.7554/eLife.73523)
Supplement: Figure 3—figure supplement 1—source data 2. [file elife-73523-fig3-figsupp1-data2.zip › Labelled blots.pdf]

B

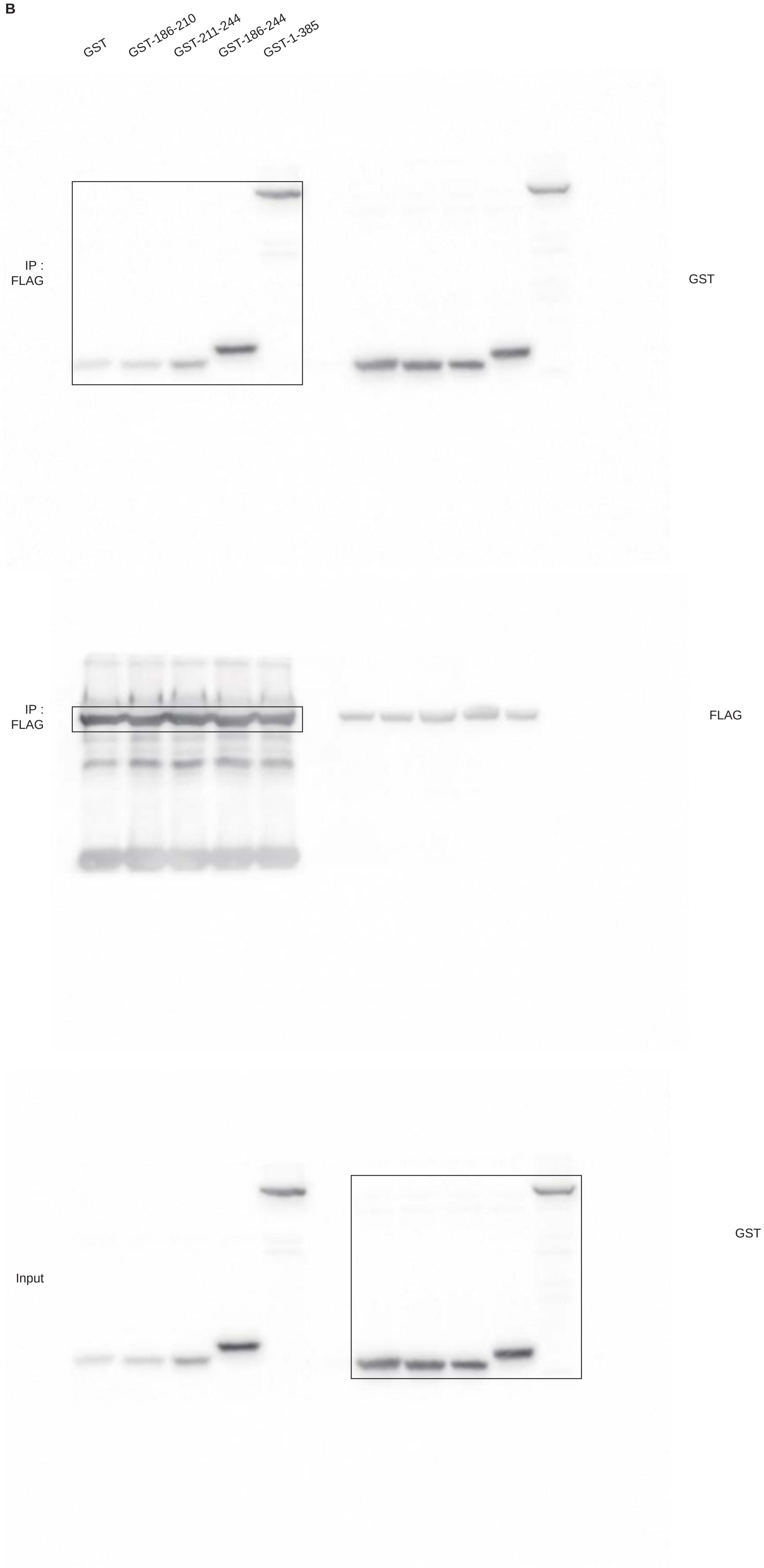
GST
GST-186-210
GST-211-244
GST-186-244
GST-1-385
IP : FLAG
GST
IP : FLAG
FLAG
Input
GST
