# Supplementary material for: Inositol polyphosphate multikinase physically binds to the SWI/SNF complex and modulates BRG1 occupancy in mouse embryonic stem cells
Source: eLife. 2022 May 12;11:e73523. doi: 10.7554/eLife.73523 (PMC9098221; doi:10.7554/eLife.73523)
Supplement: Figure 4—figure supplement 1—source data 1. [file elife-73523-fig4-figsupp1-data1.zip › Labelled blots.pdf]

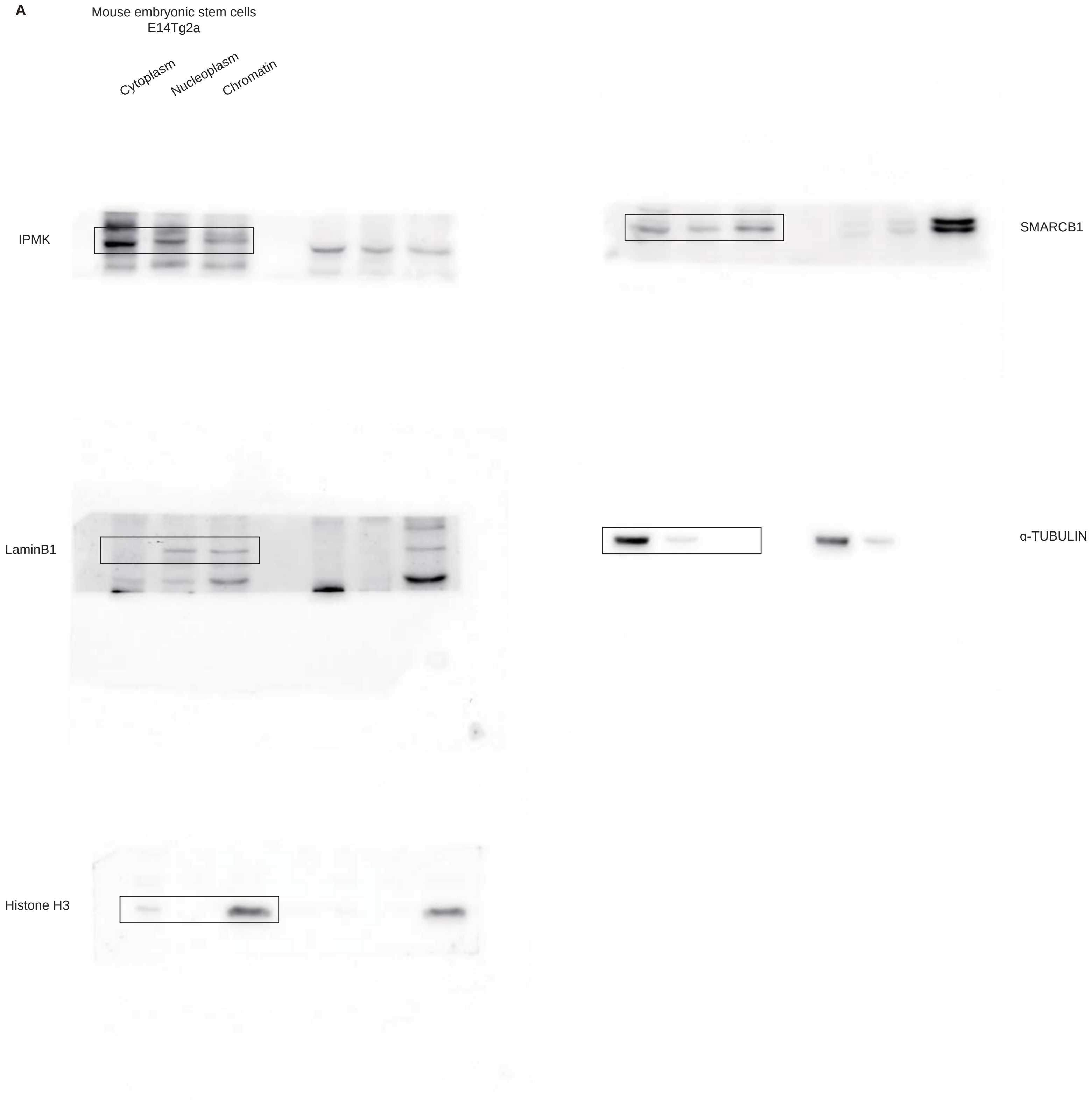
A
Mouse embryonic stem cells
E14Tg2a
Cytoplasm
Nucleoplasm
Chromatin
IPMK
SMARCB1
LaminB1
α-TUBULIN
Histone H3
